# π‐Hole Interactions Involving Nitro Aromatic Ligands in Protein Structures

**DOI:** 10.1002/chem.201903404

**Published:** 2019-09-17

**Authors:** Antonio Bauzá, Antonio Frontera, Tiddo Jonathan Mooibroek

**Affiliations:** ^1^ Department of Chemistry Universitat de les Illes Balears Crta. de Valldemossa km 7.5 07122 Palma (Baleares) Spain; ^2^ van ‘t Hoff Institute for Molecular Sciences Universiteit van Amsterdam Science Park 904 1098 XH Amsterdam The Netherlands

**Keywords:** density functional calculations, ligand–protein interactions, nitro compounds, PDB analysis, pi–hole interactions

## Abstract

Studying noncanonical intermolecular interactions between a ligand and a protein constitutes an emerging research field. Identifying synthetically accessible molecular fragments that can engage in intermolecular interactions is a key objective in this area. Here, it is shown that so‐called “π‐hole interactions” are present between the nitro moiety in nitro aromatic ligands and lone pairs within protein structures (water and protein carbonyls and sulfurs). Ample structural evidence was found in a PDB analysis and computations reveal interaction energies of about −5 kcal mol^−1^ for ligand–protein π‐hole interactions. Several examples are highlighted for which a π‐hole interaction is implicated in the superior binding affinity or inhibition of a nitro aromatic ligand versus a similar non‐nitro analogue. The discovery that π‐hole interactions with nitro aromatics are significant within protein structures parallels the finding that halogen bonds are biologically relevant. This has implications for the interpretation of ligand–protein complexation phenomena, for example, involving the more than 50 approved drugs that contain a nitro aromatic moiety.

## Graphical abstract

**Nitro aromatic ligands** have a positive electrostatic potential on the nitro group N atom (the so‐called π‐hole) that can interact with lone‐pair O/S electrons with energies of about −5 kcal mol^−1^. A thorough Protein Data Bank (PDB) analysis and scrutiny of relevant literature revealed the structural and functional significance of π–hole interactions of nitro aromatic ligands within proteins.

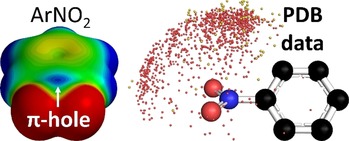

## Introduction

Noncovalent interactions largely determine how molecules interact with one another.^1^ σ‐Hole interactions,^2^ such as hydrogen bonding, figure prominently among identifiable interactions that bear biological significance.^3^ σ‐Hole interaction can also occur with other nonmetals,^4^ such as in the case of halogen bonding.^5^ The biological significance of halogen bonding has become increasingly clear since about 2004^6^ and systematic evaluations of the Protein Data Bank (PDB)^7^ revealed that halogen bonding is orthogonal to hydrogen bonding from a structural perspective.5a, 6, 8 Moreover, halogen bonding has been exploited in systematic drug discovery,^9^ used to control the conformational isomer of a four‐stranded DNA junction^10^ and to control the conformation of peptides.^11^

The impact of a novel type of interaction on molecular biology begs the question what other yet underexplored noncovalent interactions await a similar exploitation. In this context, π‐holes2c, 12 found on carbonyls^13^ and π‐acidic aromatics^14^ must be highlighted as known electron deficient moieties that interact favorably with electron rich partners.^15^ It has been disclosed that π‐holes are also found on nitro‐derivatives (R‐NO_2_).^16^ Nitrate anions can even act as π‐holes in crystal structures of proteins and small molecules when the negative charge is dampened by interactions such as hydrogen bonding^17^ or coordination to a metal.^18^ The gas‐phase complex between trimethylamine and nitroethane has been recently elucidated with rotational spectroscopy, revealing that the π‐hole bonding geometry prevails over competing arrangements involving C−H⋅⋅⋅O/N hydrogen bonding.^19^

**Figure 1:**
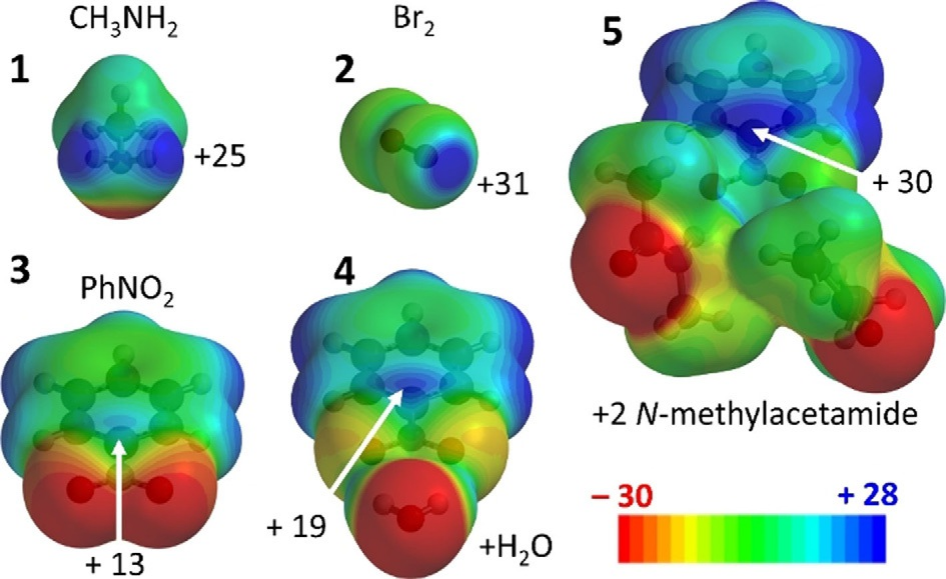
Electrostatic potential maps (MEPs) of model compounds. MEPs shown at isodensity surface of 0.002 a.u. of some σ‐holes (**1** and **2**) and π‐holes (**3**–**5**) with their electropositive potential maps in kcal mol^−1^ (MP2/aug‐cc‐pVDZ//B3LYP/6–31+G*, see also section 1.1. of the Supporting Information).

The potency of a σ‐ or π‐hole can easily be estimated by inspection of molecular electrostatic potential maps, according to which a more positive value is predictive of a stronger interaction. This potential is computed at +25 kcal mol^−1^ for methylamine and +31 kcal mol^−1^ for Br_2_, which are good hydrogen‐ and halogen‐bond donors, respectively (see Figure 1). The moderate potential of the π‐hole on nitrobenzene (**3**, +13 kcal mol^−1^) is readily amplified by hydrogen bonding to water (**4**, +19 kcal mol^−1^) or amides (**5**, +30 kcal mol^−1^).

The π‐hole bonding geometry of **3** or **4** with some O‐ or S‐bearing molecules (e.g., water and dimethylthioether) already indicate an energy contribution up to about −5 kcal mol^−1^ (see section 1.2. of the Supporting Information for details). Moreover, displacing the water molecule in a [**3**⋅⋅⋅OH_2_] π‐hole bonding geometry away from N hardly impacts the interaction energy (<11 %) below 0.25 Å displacement. Given these computational predictions, it was wondered if π‐hole interactions with nitro aromatic ligands currently might have structural and/or functional significance in proteins. This is particularly interesting because nitro aromatics are synthetically accessible and incorporated within many approved drugs,^20^ including the inexpensive and abundantly used antibiotic chloramphenicol,^21^ psychoactive drugs like the valium derivative clonazepam,^22^ the nonsteroidal antiandrogen flutamide^23^ used to treat prostate cancer, venetoclax^24^ used to treat chronic lymphocytic leukemia, dantrolene,^25^ which is the only clinically availably drug to treat malignant hypothermia, and nefidipine,^26^ which is used to manage angina.

The combined PDB study and computational analyses reported herein provide ample evidence to support the hypothesis that π‐hole interactions with nitro aromatic ligands in protein structures have structural and functional significance.

## Results

### Numerical PDB analysis

**Figure 2:**
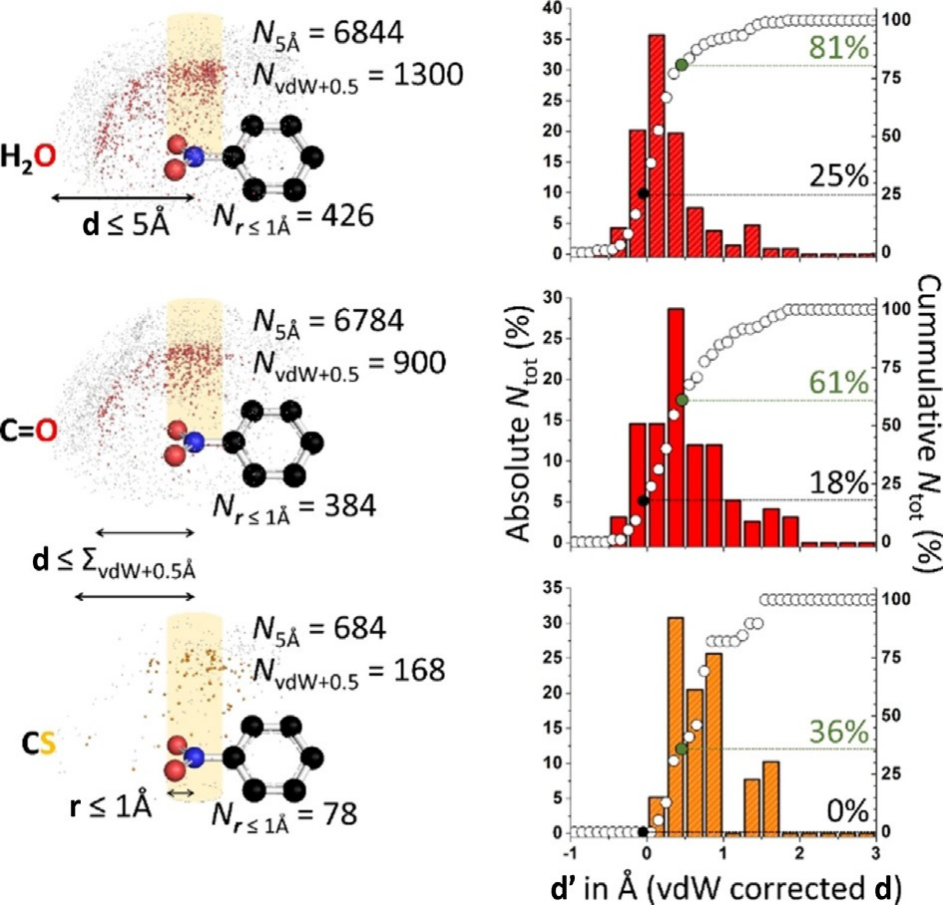
Summary of the PDB data for nitro arenes interacting with a water O atom (top), a carbonyl O atom (middle) or a carbon‐bound S atom (bottom). Left: distribution of data around the average model nitro arene (centered on the N atom); right: distribution of data with *r*≤1 Å plotted as a function of *d“. r*=the parallel displacement parameter away from N in the CNO_2_ plane; *d′*=the van der Waals corrected ^nitro^N⋅⋅⋅O/S distance *d* [i.e., *d*−vdW(N)−vdW(O/S)]. See the main text and section 2 of the Supporting Information for details.

An initial dataset was retrieved from the PDB for which the distance (*d* in Figure 2, top left) between the N atom of any of the >1500 nitro aromatic ligands and some interacting atom was 5 Å or less (see section 2 of the Supporting Information for full technical details). The interacting atom could be the O atom of a water molecule (6844 hits, top left in Figure 2), a carbonyl moiety (middle left, 6784 hits), or any carbon‐bound S atom (bottom left, 684 hits).

All these data are shown as small grey, red, or orange spheres in the left‐hand side of Figure 2 and are confined within a hemisphere with a radius of 5 Å radius (hence the label “*N*_5Å_” in Figure 2). This hemisphere is centered on the N atom of a model nitro aromatic, which is shown in ball‐and‐stick representation. The model nitro aromatic was reconstructed from the actual data, as is detailed in section 2.2 of the Supporting Information. Data that enveloped the central N atom at a distance *d*≤ the sum of the van der Waals (vdW) radii of N+O(or S)+0.5 Å (i.e., “*N*_vdW+0.5_” in Figure 2) are colored in red (for O) or orange (for S). These subsets contain significant amounts of data and are distributed mainly above (/below) the ‐NO_2_ moiety.

To probe the distribution of data directly above (/below) the N atom, the data characterized by a parallel displacement from the N atom in the nitro fragment of *r*≤1 Å (see the bottom left) was further scrutinized. The graphs shown on the right‐hand side of Figure 2 display the absolute (colored bars) and cumulative (white circles) percentages of data with *r*≤1 Å as a function of *d“*, which is the van der Waals corrected N⋅⋅⋅O(or S) distance *d* [i.e., *d*−vdW(N)−vdW(O/S)]. For the data involving water O atoms (*N*_***r***≤1Å_=426, top) a Gaussian‐like distribution of data is observed and 25 % of all hits are apparently involved in overlapping van der Waals shells (black circle and dotted line), which is a clear indicator for a bonding interaction. Loosening this rather strict criterion to data 0.5 Å longer than this benchmark (green circle and dotted line) even encompasses 81 % of the data. Hits involving carbonyl O atoms resulted in a wider distribution of data and less van der Waals overlap (18 %, *N*_***r***≤1Å_ = 384, middle right; these data might involve some π–π stacking). Still, 61 % of the data falls within the looser Σ_vdWaals_+0.5 Å criterion. For carbon bound sulfur atoms (bottom right) there were merely 78 data points. No van der Waals overlap was observed, yet 36 % of the data falls within the Σ_vdWaals_ +0.5 Å criterion. Further attempts to quantify the directionality of this interaction were inconclusive (see section 2.3 of the Supporting Information) due to the diverse steric effects of the nitro aromatic ligands and/or because there were insufficient data points.15b, 16a, 16b, 17

### Structural relevance

The above PDB analysis demonstrates that geometries consistent with π‐hole interactions involving nitro aromatics are abundant in protein structures. To assess whether existing literature contains evidence of the structural and/or functional significance of these interactions, all the data characterized by *r*≤1 Å and *d*≤Σ_vdWaals_+0.5 Å were inspected manually. These data were found in 110 unique protein structures, as is detailed in section 3.1 of the Supporting Information. Manual inspection of these structures and the articles in which they were published yielded a total of 28 cases in which π‐hole interactions appear to bear a structural and/or functional relevance. All these cases were analyzed as detailed in sections 3.2–3.9 of the Supporting Information and the most striking instances are highlighted below.

**Figure 3:**
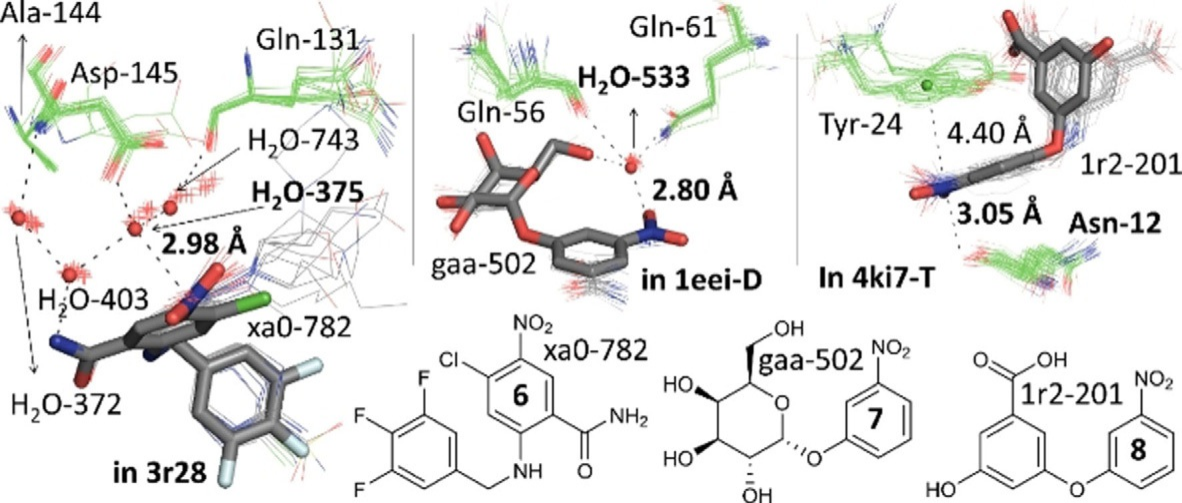
π‐Hole interactions with nitro aromatic ligands found within protein–ligand complexes. Left: Perspective view of 20 aligned nitro arene ligands (grey wires) and relevant residues (green wires) as found within 20 cyclin‐dependent kinases 2 structures. The ligand shown in capped grey sticks is ligand **6** found in PDB 3r28. The red spheres represent the center of mass of the waters 372, 375, 403 and 743. Middle: Perspective view of 35 aligned nitro arene ligands (grey wires) and relevant residues (green wires) as found within 9 cholera toxin B pentameric structures. The ligand shown in capped grey sticks is ligand **7** found in chain D of 1eei. The red sphere represents the center of mass of all water‐533 O‐atoms. Right: Perspective view of 38 aligned nitro arene ligands (grey wires) and relevant residues (green wires) as found within 2 type II dehydroquinase structures. The ligands shown in capped grey sticks is ligand **8** found in chain T of 4ki7. The green sphere represents the center of mass of all aromatic rings of the Tyr‐24 residues.

As is illustrated in Figure 3, three classes of structures were found in which the close proximity of a water ligand or protein amide O atom is highly conserved and indicative of a π‐hole interaction. As is shown in the left‐hand side of Figure 3, each of the various nitro aromatic ligands within 20 (*Homo sapiens*) cyclin‐dependent kinase 2 structures are located in the same binding pocket comprising Gln‐131, Ala‐144 and Asp‐145 (see also Figure S9, Supporting Information).^27^ One nitrate O atom is H‐bonded to H_2_O‐743 (not shown) which likely enhances the π‐hole potential (estimated at +14.3 kcal mol^−1^ for a water complex). Of the four water molecules that fill the void in between the ligand and residues, water‐375 is always in close proximity to the nitrate moiety. In structure PDB 3r28, the distance between this water molecule O atom (red sphere) and the nitrate N atom of ligand **6** (xa0‐788, capped sticks) is 2.98 Å (average=3.04 Å), which is 0.09 Å within the sum of the van der Waals radii of N and O (3.07 Å). The four close contact distances found with water‐375 suggest that water‐375 acts with both electropositive (H) and electronegative sites (the O lone‐pairs), where one interacting partner is the nitro group π‐hole.

Shown in the middle of Figure 3 is the alignment of 35 nitro aromatic ligands found in nine pentamers of (*Vibrio cholerae*) cholera toxin (CT) B^28^ that have two close contacts to water H_2_O‐553 (see also Figure S10, Supporting Information). One close contact is to the nitro group N atom with a N⋅⋅⋅O distance of 2.80 Å involving ligand **7** (gaa‐502) in chain D of structure 1eei. This is 0.27 Å below the van der Waals benchmark (N+O=3.07 Å). This water molecule is also bound by residues Gln‐56 and Gln‐61, thus displaying a total of four close contact distances. One of the nitro O atoms is H‐bonded to the amide NH of Gln‐56 (not shown), which will enhance the magnitude of the π‐hole (estimated at +26.3 kcal mol^−1^ for the water complex). Interestingly, it is known that CT is 100 times better inhibited by **7** (IC_50_=1700 μm) than regular d‐galactose.28e This nitro aromatic ligand has consequently been exploited as an anchoring platform to develop nonspanning bivalent CT inhibitors with IC_50_‐values up to 9 μm.28e In the best inhibitor the binding pocket shown in Figure 3 is preserved (shortest N⋅⋅⋅O distance=3.17 Å with ligand BV3 in chain X of structure 1rdp).28e

The 38 nitro aromatic ligands found within two (*Mycobacterium tuberculosis*) type II dehydroquinases are highlighted in the right‐hand side of Figure 3 (see also Figure S11, Supporting Information).^29^ In these examples, the nitro aromatic group is not H‐bonded to any residue, yet consistently sandwiched in between Tyr‐24 and the amidic O atom of Asn‐12. The shown N⋅⋅⋅O distance (3.05 Å) involving ligand **8** (1r2‐201) and Asn‐12 is found in chain T of structure 4ki7 (π‐hole estimated at +22.0 kcal mol^−1^). Such nitro aromatic ligands are potent inhibitors for type II dehydroquinase with up to 92 % inhibition and *K*_i_=20 μm.^29^

### Functional relevance

**Figure 4:**
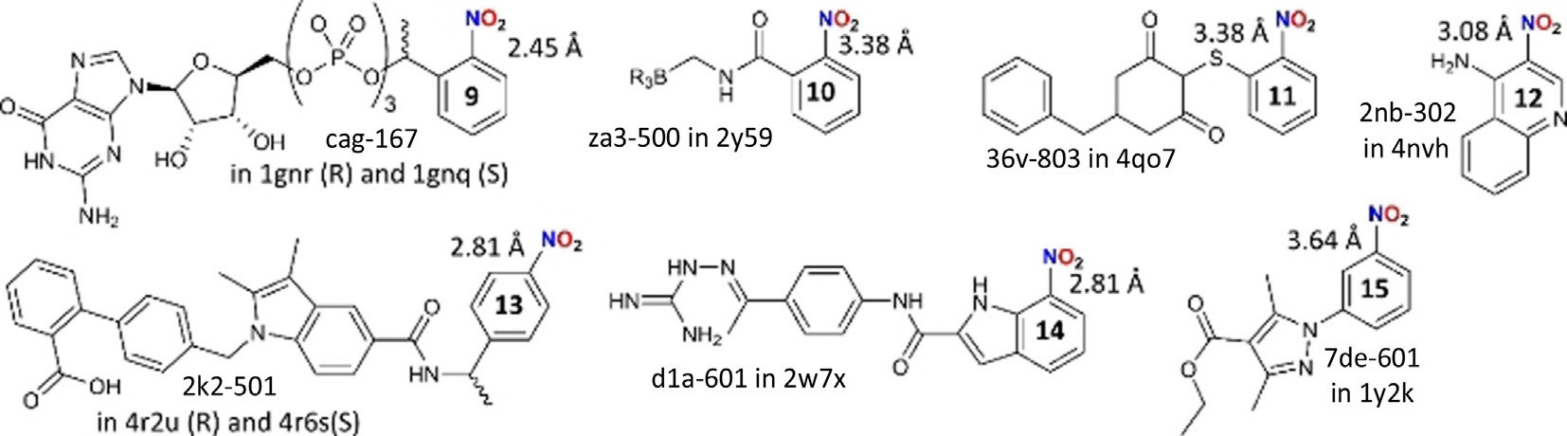
Overview of nitro aromatic ligands **9**–**15** found within the PDB that have their nitro group N atom in close proximity (as indicated) to a water O atom (**9**), a carbonyl O atom (**10**–**14**) or a residue *S* atom (**15**). These short distances could explain the functional significance of these π‐hole interactions, as detailed in the main text.

Besides these three classes of structures with highly preserved π‐hole interaction geometries, various examples were encountered for which the π‐hole interaction can be significant in the function of the protein. Shown in Figure 4 is an overview of the ligands involved in these cases and detailed structural information is given in section 3 of the Supporting Information.

Structures 1gnr and 1gnq are (*Homo sapiens*) oncogene product p21^H‐RAS^ guanine nucleotide binding proteins that have their catalytic domains occupied by enantiomerically pure R/S **9** (ligands cag‐167, see Figure 4).^30^ The binding pockets of both these protein structures are very similar as is detailed in Figure S12 (Supporting Information). However, in structure 1gnr (the *R* isomer) a water molecule (H_2_O‐523) is found in close contact to the nitro group (N⋅⋅⋅O=2.45 Å). This interaction may well contribute to the slower phosphate hydrolysis observed (half‐life of 1000 days versus 70 days for the *S* isomer in 1gnq) by stabilizing the water molecule involved in the hydrolysis reaction.^30^

Ligand **10** (za3‐500) is found within penicillin‐binding protein complex 2y59.^31^ In chain B, the nitro moiety is nearby the O atom of Thr‐413 (N⋅⋅⋅O=3.38 Å, see Figure S14, Supporting Information). Nitro aromatic **10** is by far the strongest binder of the ligands tested with *K*_i_=0.36 μm, 117 times more potent than the isostructural chloride analogue (*K*_i_=42 μm).^31^

Nitro aromatic **11** (36v‐803) is found in protein complex 4qo7, a (*Homo sapiens*) lactate dehydrogenase A.^32^ As is detailed in Figure S15 (Supporting Information), the carboxylate of Asp‐165 is located directly above the nitro group (N⋅⋅⋅O=3.38 Å in chain A). In a study testing 60 ligands as inhibitors, **11** was found to be 31 times more potent than the ‐Br analogue (LC_50_=1.7 vs. 53 μm).^32^

Compound **12** is found as ligand 2nb‐302 in (*Saccharomyces cerevisiae*) cytochrome c peroxidase structure 4nvh.^33^ Within the binding pocket (detailed in Figure S16, Supporting Information) the nitro N atom is merely 3.08 Å away from the O atom of Met‐228. This methionine residue is preserved in the nearly identical binding pockets of 4nvi (containing a ‐Br analogue) and 4nvg (containing an ethyl ester analogue). The affinity (*K*_d_) of **12** for the protein (7 μm) is 2.5 and 10 times higher than the isostructural ‐Br (18 μm) and ethyl ester (71 μm) analogues.^33^

The *S* (3k2‐501) and *R* (3k2‐501) enantiomers of **13** were found in (*Homo sapiens*) nuclear receptor peroxisome proliferator‐activated receptor gamma (PPAR*γ*) structures 4r6s and 4r2u, respectively.^34^ Interestingly, the *S* enantiomer binds 40 times stronger to PPAR*γ* (*K*_d_=2 nm) than the *R* enantiomer (*K*_d_=80 nm). A major difference in the binding pockets of these protein complexes is the orientation of an O‐atom of side‐chain amide of Gln‐286 relative to the nitro aromatic. As is detailed in Figure S17 (Supporting Information), this O atom is pointing towards the π‐hole for the stronger binding *S* isomer with a N⋅⋅⋅O distance of 2.81 Å, which is 0.26 Å below the van der Waals benchmark. This O atom is pointing away from the nitro aromatic in the weaker bound *R* isomer.

**Figure 5:**
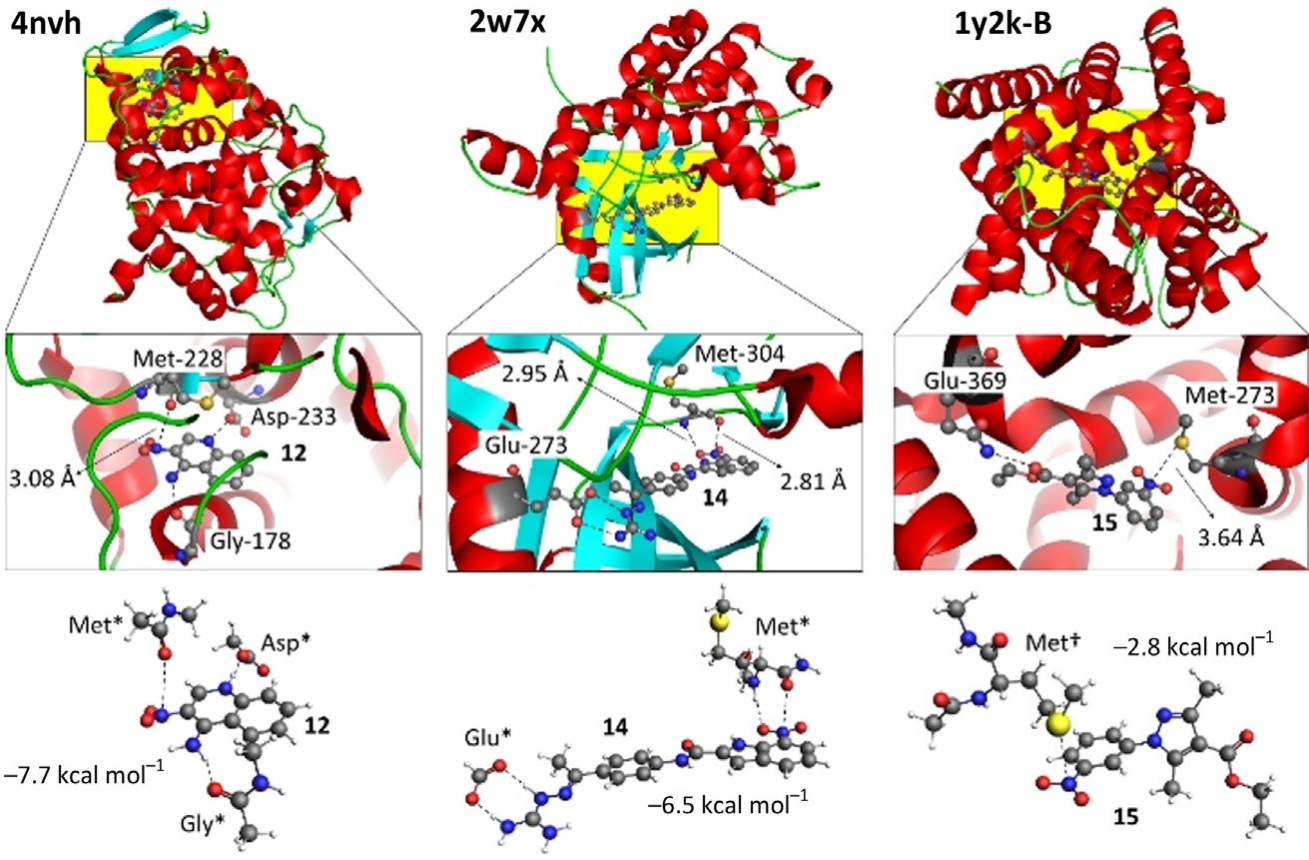
Examples of high level computational analysis (SCS‐MP2/def2‐TZVP) to estimate the energetic contribution of the π‐hole interactions found in structures 4nvh (left) 2w7x (middle) and 1y2k (right). The top picture shows the protein structure with helices in red, sheets in cyan and loops in green. The yellow area highlights the binding pockets as magnified in the middle. The bottom is a model of the binding pocket with optimized energy.

7‐Nitro‐1*H*‐indole derivative **14** (d1a‐601) was found in (*Homo sapiens*) checkpoint kinase 2 structure 2w7x (see also Figure 5), in which it displays a very short nitro N⋅⋅⋅O (Met‐304) distance of 2.81 Å (i.e., 0.26 Å below the van der Waals benchmark).^35^ As is detailed in Figure S18 (Supporting Information), this complex is part of a series of seven structures containing very similar ligands, four of which contain the 7‐nitro‐1*H*‐indole fragment.^35^, ^36^ The residues surrounding the ligands as well as their relative orientation is highly preserved throughout these seven structures. The short N⋅⋅⋅O distance is also preserved in the four structures involving a 7‐nitro‐1*H*‐indole ligand (average distance=2.87 Å) and this interaction actually is one of only two polar contacts with the ligands. Interestingly, **14** displays the shortest N⋅⋅⋅O contact distance and is also the best inhibitor tested with IC_50_=0.03 nm, which is at least 45 times more potent than ligands that lack the nitro group (e.g., IC_50_=1.36 nm for nxp‐600 in 2ycs, see Figure S18, Supporting Information).^35^, ^36^

Nitro aromatic ligand **15** (7de‐601) co‐crystallized with (*Homo sapiens*) phosphodiesterase (PDE) 4D (1y2k) and 4B (1y2j) where it is situated in nearly identical binding pockets (see Figure S22 in the Supporting Information for details).^37^ The nitro moiety is in close contact with the S atom of Met‐273 with N⋅⋅⋅S=3.64 Å (in chain B of 1y2k, see also Figure 5). Interestingly, **15** was found to be the most potent inhibitor for both PDE‐4D (IC_50_=21 nm) and PDE‐4B (IC_50_=33 nm). The IC_50_ values for the other 20 inhibitors tested are typically in the (higher) mm range.^37^

### Computational studies

To probe the energetic contribution of the likely π‐hole interactions involving **6**–**15**, model binding pockets were evaluated computationally (see sections 3.1 and 3.9 of the Supporting Information). Atomic coordinates of relevant C, N and O atoms were retrieved from the PDB, H atoms were added and their position optimized at the BP86^38^‐D3^39^/def2‐TZVP^40^ level of theory after which an energy calculation was conducted at the much higher SCS‐MP2^41^/def2‐TZVP^40^ level of theory (Δ*E* ^1^). The def2‐TZVP basis set was chosen due to the very low basis set superposition error.^40^ For comparison, interaction energies were also computed of isostructural nitroso arenes (i.e., in silico ‐NO_2_ to ‐NO mutation; Δ*E* ^2^), which are sterically similar but have a negligible π‐hole (+3.1 kcal mol^−1^, see Figure S8, Supporting Information). The differences in interacting energies (ΔΔ*E*=Δ*E* ^1^−Δ*E* ^2^) were always negative and as large as −4.4 kcal mol^−1^ for **13** in 4r6s, which is indicative of the energetic contribution of the nitro moiety π‐hole interaction.

Three examples were scrutinized further; **12** in structure 4nvh and **14** in structure 2w7x highlighted on the left and in the middle of Figure 5 serve as an example for a π‐hole interaction with an amidic carbonyl O‐atom. Compound **15** in 1y2k highlighted in the right‐hand side of Figure 5 involves a carbon‐bound S atom.

Inhibitor **12** is shown in Figure 5 (left), bound within the active site of cytochrome c peroxidase structure 4nvh by hydrogen bonding to Gly‐178 and Asp‐233 (see also Figure S16, Supporting Information). Additionally, the carbonyl O atom of Met‐228 is in close proximity of the ligand nitro moiety with ^nitro^N⋅⋅⋅O^amide^=3.08 Å. The interaction energy of a model for the nitroarene–amide interaction (bottom left in Figure 5) is −7.7 kcal mol^−1^. An energy decomposition analysis (see section 1.3. of the Supporting Information) indicates that electrostatics (53 %), orbital interactions (22 %) and dispersion (25 %) all contribute significantly to the binding energy. Mutating the amide C=O to a CH_2_ group resulted in an interaction energy of merely −2.1 kcal mol^−1^, which means that the π‐hole interaction can account for about −5.6 kcal mol^−1^. Interestingly, the bromide analogue of ligand **12** is found within the structure 4nvi. Both binding pockets are nearly identical to one another, as detailed in Figure S16‐2 (middle; Supporting Information). The binding energies for model binding pockets are −119.0 kcal mol^−1^ for the ‐NO_2_ ligand and −109.3 kcal mol^−1^for the ‐Br ligand (SCS‐MP2‐def2‐SVP, see section 1.4. of the Supporting Information). Mutating the NO_2_ ligand in silico to the ‐Br analogue while retaining the model binding pocket of 4nvh gave an energy penalty of +9.0 kcal mol^−1^. Conversely, in silico ‐Br→‐NO_2_ mutation of the ligand in 4nvi gave an energy gain of −5.5 kcal mol^−1^. These calculations are consistent with the observations that nitro aromatic **12** binds stronger to the protein (*K*_d_=7 μm) than its ‐Br analogue (*K*_d_=18 μm).^33^

Inhibitor **14** is anchored within the active site of checkpoint kinase 2 structure 2w7x by a strong salt bridge to Glu‐273 (Figure 5, middle). An additional polar interaction involving Met‐304 is a combined H‐bonding (N⋅⋅⋅O=2.95 Å) and π‐hole (N⋅⋅⋅O=2.81 Å) interaction. Curiously, no other H‐bonding interaction is established between the inhibitor and the active site, whereas both NHs of the inhibitor interact with water molecules (not shown). The interaction energy of the complex [**14**H^+^⋅⋅⋅^−^O_2_CH]⋅⋅⋅Met* (bottom center in Figure 5) is −6.5 kcal mol^−1^, which accounts for both the N−H⋅⋅⋅O H‐bond and the π‐hole interaction. An energy decomposition analysis (see section 1.3. of the Supporting Information) revealed that 51 % of the attractive forces are electrostatic in nature, with orbital interactions and dispersion accounting for 24 and 25 % respectively. Mutating the amide C=O involved in the π‐hole interaction, or the amide N−H involved in the hydrogen bond to a H atom (i.e. CH_2_ or CH) resulted in interaction energies of Δ*E* ^H‐bond^ = −2.9 kcal mol^−1^ (i.e., only the H‐bonding interaction) and Δ*E* ^π‐hole^=−4.4 kcal mol^−1^ (i.e., only the π‐hole interaction). This implies that the contribution of the π‐hole interaction is of similar magnitude as the H‐bond, and actually most significant at about −4.0 kcal mol^−1^. An energy decomposition analysis (see section 1.3. of the Supporting Information) of the two mutants revealed that dispersion contributes somewhat more in the π‐hole interaction (38 %^π‐hole^ vs. 24 %^H‐bond^), whereas the orbital contribution is larger in the H‐bonding interaction (13 %^π‐hole^ vs. 30 %^H‐bond^). The electrostatic component is about the same on both cases (49 % ^π‐hole^ vs. 46 %^H‐bond^).

Inhibitor **15** is found within the active site of phosphodiesterase structure 1y2k (Figure 5, right), in which it is H‐bonded to only one residue (Glu‐369). The nitro‐group is H‐bonded to a water molecule (not shown) and is in close proximity to the S atom of Met‐273. The interaction energy of the complex **15**⋅⋅⋅Met^†^ (bottom right in Figure 5) is −2.8 kcal mol^−1^. Addition of a water molecule H‐bonded to the nitro group increased the interaction energy to −4.0 kcal mol^−1^. An energy decomposition analysis (see section 1.3. of the Supporting Information) revealed that about 25 % of the attractive forces are electrostatic in nature, with orbital interactions and dispersion each accounting for about 15 and 60 %, respectively.

### Approved nitro aromatic drugs within the PDB

Considering that the evidences from structure data, literature studies, and our model computations all point towards the relevance of π‐hole interactions involving nitro aromatic ligands in protein structures, we assessed which approved drugs contain a nitro aromatic and looked for their occurrence in the PDB (see section 4 of the Supporting Information for details). In total, 54 such drugs were identified (see Table S5, Supporting Information) and nine of these drugs (or very similar molecules) were present as a ligand: chloramphenicol (and related ligands),^21^ nimodipine,^42^ hydroxy flutamide (the active metabolite of flutamide),^23^ nifursol‐like ligands,^43^ a venetoclax‐like ligand,^24^ tolcapone,^44^ nimesulide,^45^ nitroxoline,^46^ and oxaminiquine.^47^ Manual inspection of the 5 Å envelope surrounding the NO_2_ moiety revealed that in 32 of the 57 structures (56 %) there is at least one electron‐rich moiety near the nitro group (i.e., atoms with lone‐pair electrons and/or aromatic rings). These 32 structures included all of the nine abovementioned drugs—except for a finurisol‐like ligand—and are summarized in Figures S37–S41 (Supporting Information).

## Discussion

The numerical analysis of the data compiled in the PDB (Figure 2) shows clustering of data near the π‐hole region. These data and the examples highlighted in Figures 3–5 show that π‐hole interactions with nitro aromatic ligands are a common structural phenomenon within the PDB and might thus be functionally relevant. The lack of apparent van der Waals overlap involving carbon bound S vis‐à‐vis water and carbonyl O atoms is in line with the model computations involving dimethyl thioether (see section 1 of the Supporting Information). Moreover, it has been reported that close proximity of the S atom of methionine and the π‐system of histidine is conserved within at least 80 protein structures, without displaying van der Waals overlap.^48^ This interaction was furthermore shown to stabilize transition state 2 of phosphite dehydrogenase with 7.9 kcal mol^−1^.^48^ This suggests that for S atoms, overlap of van der Waals shells is not a strict prerequisite for the relevance of an interaction with that S atom. In light of this, it is even more remarkable that so many data were found for which water and carbonyl O atoms are involved in apparent van der Waals overlap with the nitro group N atom and presents experimental evidence for the significance of such π‐hole interactions within proteins.

Several examples were encountered in the literature for which a nitro aromatic ligand displayed a better inhibition or binding affinity compared to a very similar non‐nitro analogue. In all cases highlighted, the protein–ligand interaction includes a π‐hole interaction with the nitro aromatic ligand, as is evident from the crystal structures. This clearly suggests a functional relevance of these π‐hole interactions.

The computational data involving simple models of nitro arene ligands (**3** and **4**, Table S1, Supporting Information) predict that π‐hole interactions are about −5 kcal mol^−1^. These interaction energies are likely a conservative estimate, as nitro aromatics bound within a protein can bear a deeper π‐hole due to (charge assisted) H‐bonding, like in the bis‐amide model complex **5** (Figure 1). The interaction energies estimated with inhibitors **12**, **14** and **15** highlighted in Figure 5 indeed show that π‐hole interactions can contribute about 5 kcal mol^−1^ to the overall binding energy of a protein–ligand complex. This is twice as strong as the H‐bonding interaction between the α‐H of side chain amino acids with water (1.9–2.5 kcal mol^−1^)^49^ and of the same magnitude as a water dimer.^50^ Thus, the strength of these π‐hole interactions is in general in the order of a moderately strong hydrogen bond.^51^ It is worth pointing out that interactions of that magnitude are well known to play an important role in biological systems. For example, carbonyl–carbonyl π‐hole interactions can be a structural determinant in protein folding.13b C−H⋅⋅⋅π interactions are a well‐preserved structural feature in protein carbohydrate complexes^52^ and have even been successfully exploited as important design elements for making artificial carbohydrate receptors.^53^

The search for approved nitro aromatic drugs within the PDB revealed nine drugs within 57 PDB structures. In 32 (56 %) of these structures the nitro moiety is in closest proximity to an aromatic and/or a lone pair bearing atom of a residue. This, again, suggests that π‐hole interactions with nitro aromatics is a common structural feature. That these structural features were encountered with eight out of the nine approved drugs found as ligand in the PDB again hints to the functional significance of these π‐hole interactions.

## Conclusions

It is thus concluded that π‐hole interactions of nitro aromatic ligands within proteins are a force to be reckoned with. Explicit examples are provided for π‐hole interactions in biological systems. This discovery likens to the finding that halogen bonds are biologically significant. We thus expect that taking π‐hole interactions into account will aid future interpretations of the structure/function relationship involving nitro aromatics, including the more than 50 currently approved drugs that contain the nitro aromatic moiety. Finally, our finding may serve as an aid to discover/design new ligands as potent inhibitors of a target protein.

## Conflict of interest

The authors declare no conflict of interest.

## Supporting information

As a service to our authors and readers, this journal provides supporting information supplied by the authors. Such materials are peer reviewed and may be re‐organized for online delivery, but are not copy‐edited or typeset. Technical support issues arising from supporting information (other than missing files) should be addressed to the authors.

Supplementary
