# Supplementary material for: π‐Hole Interactions Involving Nitro Aromatic Ligands in Protein Structures
Source: Chemistry. 2019 Sep 17;25(58):13436–43. doi: 10.1002/chem.201903404 (PMC6856858; doi:10.1002/chem.201903404)
Supplement: Supplementary file 1 — Supplementary [file CHEM-25-13436-s001.pdf]

CHEMISTRY
A European Journal

Supporting Information

π-Hole Interactions Involving Nitro Aromatic Ligands in Protein Structures

Antonio Bauzá,[a] Antonio Frontera,*[a] and Tiddo Jonathan Mooibroek*[b]

chem_201903404_sm_miscellaneous_information.pdf

Contents

1. Details of calculations: 2
1.1. General computational methods for calculations in the main text: 2
1.1. Details of model calculations: 2
2. Details of PDB enquiries: 5
2.1. Query for retrieving data on aromatic nitro ligands in the PDB: 5
2.2. Method to derive Cartesian coordinates of XNO_2, the interacting atom and the $ArNO_2$ model. 6
2.3. Methods to probe directionality with 3D, 4D density, P(r) and N(d’) plots 7
3. Evaluation of close contact cases. 9
3.1. General approach and overview of data. 9
3.2. Examples where a π-hole interaction is highly preserved within similar structures. 12
3.3. Examples of likely functional relevance of π-hole interactions with H_2O 15
3.4. Examples of likely functional relevance of π-hole interactions with C=O. 17
3.5. Example of likely functional relevance of π-hole interaction with C–S. 26
3.6. π-hole interactions that seem at least structurally relevant with H_2O. 27
3.7. π-hole interactions that seem at least structurally relevant with C=O. 33
3.8. π-hole interaction that seem at least structurally relevant with C–S. 40
3.9. Cartesian coordinates of computed model nitro aromatics and relevant residues. 41
4. Overview of (approved) drugs containing a nitro aromatic. 109
References: 117

1. Details of calculations:

1.1. General computational methods for calculations in the main text:

Molecular electrostatic potential surfaces were rendered with Spartan 2018 (version 2.0.7) from geometry optimized structures with density functional theory at the DFT/B3LYP[1]/6-31+G*[2] level of theory. The MEP values indicated in Figure 1 were computed at the MP2[3]/aug-cc-pVDZ[4] level of theory using the B3LYP/6-31+G* geometries. All binding energies reported herein were computed with Turbomole 7.0 program and employing the spin-component scaled second-order Møller-Plesset method (SCS-MP2)[5] and using as the basis set default 2 with triple zeta valence quality polarization function (def2-TZVP), as this gives an accurate energy at reasonable computational cost and a very low basis set superposition error (BSSE).[6]

1.2. Computation of simple model adducts:

As is detailed in Table S1, high level computations were conducted to explore the binding potential of the π-hole on nitrobenzene (**3**) or its water adduct (**4**). The interacting partners chosen were: water, obviously abundant in proteins; methanol and dimethyl ether as models for sp^3 hybridized O-atoms such as in Serine and Threonine; dimethyl thioether as model for sulfur containing residues like Methionine; and formamide as a model for Asparagine, Glutamine and amides in general. For these calculations we employed modern Møller Plesset method (SCS-MP2)[5] and using as the basis set default 2 with triple zeta valence quality polarization function (def2-TZVP), as this gives an accurate energy at reasonable computational cost and a very low basis set superposition error (BSSE).[6]

The interacting energies range from –1.7 kcal/mol for [**3**···**water**] to –4.2 kcal/mol for [**4**···**formamide**] and the water adduct of nitrobenzene (**4**) consistently results in lower energy complexes. In all cases involving O as interacting atom, > 0.2 Å van der Waals overlap is observed between N and O. The longest N···O distance (***d***) computed is 2.863 Å for [**3**···formamide], which is still 0.207 Å within the sum of the van der Waals radii[7] of N and O (i.e. 1.55 + 1.52 = 3.07 Å). Only very limited overlap of ≤ 0.04 Å is observed with dimethylthioether (i.e. N + S = 1.55 + 1.80 = 3.35 Å).

In order to probe the influence of translating the water molecule in [**3**···**water**] (–1.7 kcal/mol) away from N, several additional computations were conducted. To avoid additional influences on the interaction energies apart from the π-hole interaction, the conformation of the water molecule was confined so that the H_2O lone pair is pointing to the π-hole and the H atoms are not directed toward the nitro or aromatic π-system. The interaction energies gathered in

Table S2 confirm that small displacement of the interacting atom from the π-hole has little influence on the interaction energy (maximum displacement 0.35 Å).

An 'atoms in molecules' analysis[8] of the complexes with dimethylether and formamide (see Figure S1) clearly revealed bond critical points and bond paths connecting nitrobenzene's N and the O-atom of dimethylether or formamide, thus confirming that these π–hole interactions are bonding in nature.

Table S1. Interaction energies with basis set superposition error correction (E in kcal/mol) and equilibrium N···O(or S) distances (***d*** in Å) of complexes with **3** and **4** at the SCS-MP2/def2-TZVP level of theory.

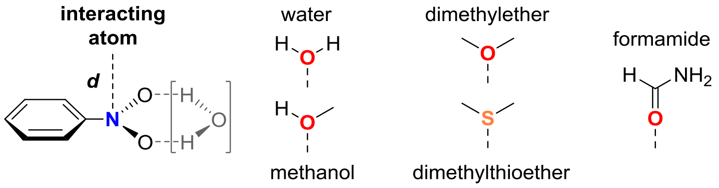

Host→	3 ($PhNO_2$)		4 ($PhNO_2 \cdots H_2O$)	
↓ Guest	E	*d*	E	*d*
water	−1.7	2.831	−6.4	2.851
methanol	−2.1	2.799	−3.0	2.789
dimethylether	−2.2	2.808	−2.7	2.763
dimethylthioether	−2.1	3.332	−2.3	3.307
formamide[a]	−4.2	2.863	−8.6	2.836

[a]The formamide is concurrently involved in N–H···O hydrogen bonding to the nitro group in **3** and the water molecule in **4**.

Table S2. Interaction energies (in kcal/mol) relative to the optimal geometry of [**3**···**water**] (–1.7 kcal/mol) at different points on the axes of the inset figure at the SCS-MP2/def2-TZVP level of theory.

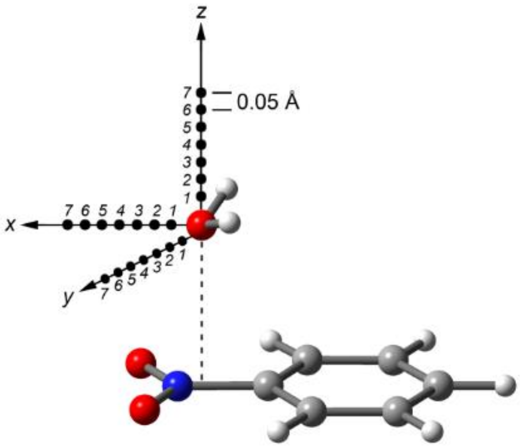

Translation: point #	Relative energy in direction: *x*	*y*	*z*
1 (0.05 Å)	<0.01 (< 0.4 %)	<0.01 (< 0.4 %)	<0.01 (< 0.4 %)
2 (0.10 Å)	<0.01 (< 0.4 %)	0.01 (0.6 %)	0.02 (1.2 %)
3 (0.15 Å)	0.01 (0.6 %)	0.03 (1.8 %)	0.06 (3.5 %)
4 (0.20 Å)	0.03 (1.8 %)	0.06 (3.5 %)	0.12 (7.1 %)
5 (0.25 Å)	0.06 (3.5 %)	0.10 (5.8 %)	0.19 (11.2 %)
6 (0.30 Å)	0.09 (5.3 %)	0.14 (8.2 %)	0.27 (15.8 %)
7 (0.35 Å)	0.13 (7.6 %)	0.20 (11.8 %)	0.35 (20.6 %)

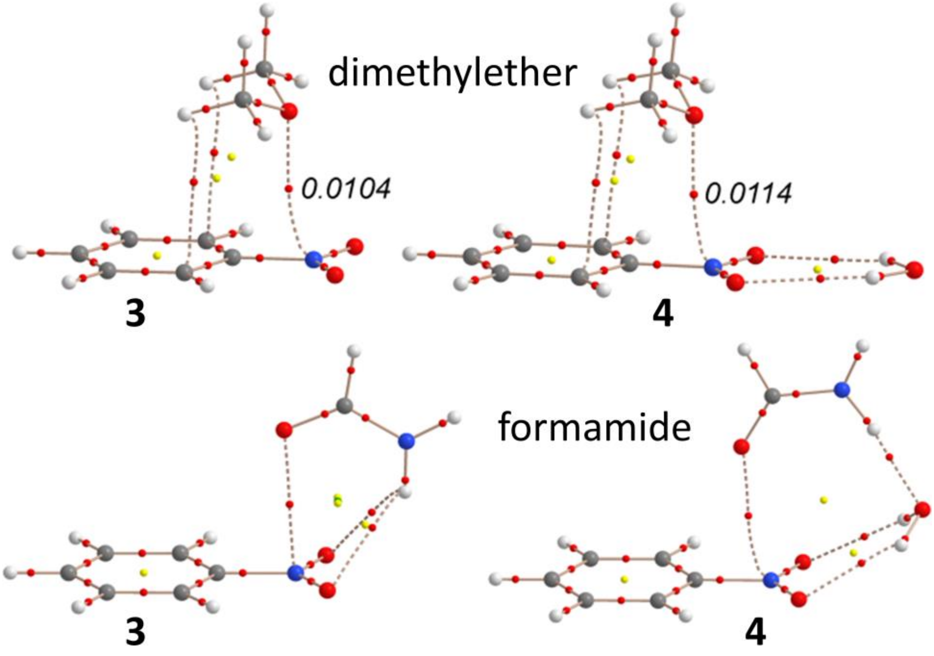

Figure S1. Geometries and distribution of critical points and bond paths of several π–hole complexes. The value of ρ(r) in a.u. is given for **3**/**4** in complex with dimethylether.

1.3. Energy decomposition and Atoms in Molecules Analyses of examples in the main text:

The energy decomposition (or fragment) analysis and the atoms in molecules analysis[8] were done with the Amsterdam Density Functional (ADF)[9] modelling suite at the B3LYP-D3/TZ2P level of theory (no frozen cores). Computed values in kcal·mol^{-1} for Pauli repulsion (PR), electrostatic attraction (EA), orbital interactions (OI) and dispersion (D): [4nvh]: +9.84 (PR), –10.71 (EA), –4.51 (OI), –5.00 (D), total = –10.37 kcal·mol^{-1}; [4nvh$^{\text{CO to CH2}}$]: +2.89 (PR), –1.42 (EA), –1.49 (OI), –4.13 (D), total = –4.15 kcal·mol^{-1}; [2w7x]: +12.75 (PR), –11.78 (EA), –5.43 (OI), –5.79 (D), total = –10.25 kcal·mol^{-1}; [2w7x$^{\text{only π-hole}}$]: +5.46 (PR), –5.81 (EA), –1.59 (OI), –4.52 (D), total = –6.46 kcal·mol^{-1}; [2w7x$^{\text{only H-bond}}$]: +7.91 (PR), –6.27 (EA), –4.03 (OI), –3.31 (D), total = –4.17 kcal·mol^{-1}; [1y2k]: +3.11 (PR), –1.54 (EA), –1.11 (OI), –4.63 (D), total = –4.17 kcal·mol^{-1}; [1y2k + H_2O]: +3.44 (PR), –2.40 (EA), –1.35 (OI), –5.36 (D), total = –5.67 kcal·mol^{-1}. An atoms-in-molecules analysis did not reveal a bond critical point in 4nvh and did in 2w7x (ρ(r) = 5.37 · 10^{-3} a.u.) and in 1y2k (ρ(r) = 10.0 · 10^{-3} a.u.).

1.4. Comparative computational evaluation of ligand-protein complexes in 4nvh and 4nvi:

All heavy atom coordinates were extracted from the PDB entry and the binding pocket was manually trimmed to include (simplified) residues that are close to the ligand. Water molecules shared by both binding pockets (i.e.: H_2O-428, 662, 664 and 723) were included. All hydrogen atoms were initially added with the automatic editor embedded in Mercury 3.10.1,[10] whereafter their positions were optimized with simple molecular mechanics. The resulting coordinates were used as input for calculations at the SCS-MP2/def2-SVP level of theory, whereby all except the H-atoms were set as frozen. The resulting structure was used to compute the binding energy by also running a single point calculation of the thus optimized ligand and pocket coordinates. For comparison purposes, an *in silico* mutant was also evaluated where the ligand's –Br was replaced by –NO_2 for 4NVI, or the ligand's –NO_2 group was changed to a –Br group for 4NVH. This procedure was repeated for both structures and omitting all water molecules from the pockets. See also Figure S16-2.

2. Details of PDB enquiries:

2.1. Query for retrieving data on aromatic nitro ligands in the PDB:

The PDB was inspected with the online Query Sketcher of Relibase version 3.2.1 on the 2nd of March 2016. An schematic overview of the query used is shown in Figure S2.

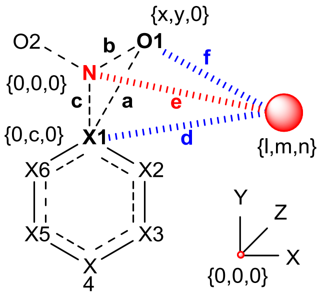

Figure S2. Illustration of the query used to extract (geometric) data from the PDB.

The aryl bonds of the central $ArNO_2$ unit were set to aromatic (solid and dashed black lines) and the other three bonds were specified as 'any type' (only dashed black lines). All covalent bond distances and selected triatomic angles as well as the O1-N-X1-X2 torsion angle were collected to reconstruct the average model used for accessing directionality (see below). The interatomic distance between the interacting atom (red sphere; O in O=C or OH_2 or S in SC) and the nitro's N-atom (**e**, highlighted in red in Figure S2) was set as ≤ 5 Å so that the data was confined within a 10 Å diameter sphere centred on N. The X_6-NO_2 central unit was marked as a ligand and the interacting atom(s) as part of a protein, or in the case of water the interacting O was specified as water. The numerical data for the amount of PDB structures (N_{PDB}) and the amount of individual hits (N_{hits}) that resulted from these queries are collected in the first two columns of Table S3 (the other columns involve narrower datasets, explained below).

Table S3. Numerical overview of search results.
Data of the column highlighted was scrutinized manually as detailed herein.

Interaction	N_{PDB}	N_{hits}	$N_{\|x\| \leq 2}$ [a]	$N_{r \leq 1}$	$N_{\leq vdW+0.5}$ [b]
N···O=C	940	6,784	3,374	384	234 (82.4%)
N···OH_2	735	6,844	3,250	426	344 (80.8%)
N···SCR[c]	160	684	434	78	28 (18.9%)

a) Data with a spherical segment of 5Å sphere and |x| ≤ 2 Å. b) and *r* ≤ 1 Å. Percentages relative to all the data with *r* ≤ 1 Å.
c) R = any atom but mostly C (methionine).

2.2. Method to derive Cartesian coordinates of XNO_2, the interacting atom and the $ArNO_2$ model.

The interatomic distances between the interacting atom (red sphere) and X2, O1 and O2, as well as the X1-O1 distance were also collected (set to ≤ 8 Å). The triangle formed by X1-N-O1 was chosen as the base, and the interacting atom as the tip of a tetrahedron (see Figure S2) so that Cartesian Coordinates {X,Y,Z} of all the atoms could be derived as follows: the N-atom was taken as the centre {0,0,0}, X1 as {0,c,0}, O1 as {x,y,0}, and the interacting atom at {**l**,**m**,**n**}. Distances **a**-**f** were measured, from which **y**, **x**, **m**, **l** and **n** can be derived using equations (1) - (5) respectively.

(1) $$y = \frac{a^2 + c^2 - b^2}{2c}$$

(2) $$x = \sqrt{a^2 - y^2}$$

(3) $$m = \frac{c^2 + e^2 - d^2}{2c}$$ (= X-value)

(4) $$l = \frac{a^2 + e^2 - f^2 - 2my}{2x}$$ (= Y-value)

(5) $$n = \sqrt{e^2 - m^2 - l^2}$$ (= Z-value)

Thus, the distance between the interacting atom and the plane defined by O1-N-X1 is **n**, i.e. the Z-value. With this and the N···interacting atom distance (**e**) the parallel displacement parameter (***r***) could be derived according to equation (6):

(6) $$r = \sqrt{e^2 - n^2}$$

With this procedure the sign of **n** (i.e. the Z-axis) is always positive, meaning that data in one half of the sphere were reflected to the other half of the sphere to obtain the data within a 5 Å high and 10 Å wide hemisphere.

To obtain all {X,Y,X} coordinates of the average model for the aromatic nitro central group, it was assumed that both the X_6 ring and the NO_2 group were planar, where the angle between these two planes is given by the O1-N-X1-X2 torsion angle. The averages of relevant distances and angles were then used together with the rules of sine and cosine to obtain the {X,Y,X} coordinates. The relative standard deviations of the parameters used were typically below 5%.

2.3. Methods to probe directionality with 3D, 4D density, P(r) and N(d') plots.

As the substituent on the aryl ring is left unspecified, we only scrutinized the data characterized by N···interacting atom ≤ 5Å and |x| ≤ 2 Å, meaning that the data is confined within half of a spherical segment centred on N and with base made dimensions of 4 x 10 Å and a 5 Å radius (see also the volume outlined in red in Figure S3).

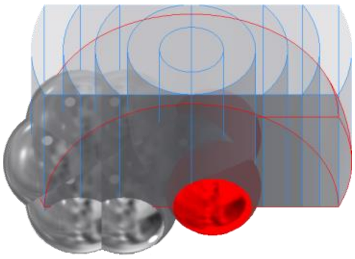

Figure S3. Illustration of the aromatic nitro model and the volumes used to access directionality. The volume outlines in red is a spherical segment centred on N with N···interacting atom ≤ 5Å and |x| ≤ 2 Å.

The amount of data within these bodies are collected in the third column of Table S3 ($N_{|x| \le 2}$) and the raw 3D data (with a simplified, flattened model $ArNO_2$) are shown in Figure S4.

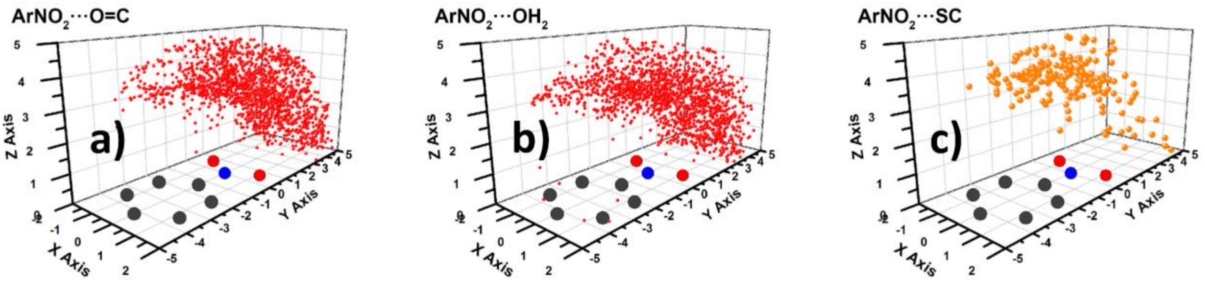

Figure S4. 3D plots of of the raw data extracted from the PDB characterized by N···interacting atom ≤ 5Å and |x| ≤ 2 Å for the interacting partner atoms: a) C=O Oxygen with 3,374 hits; b) H_2O Oxygen with 3,250 hits; and c) CSR Sulphur (R can be any atom) with 434 hits.

Four dimensional (4D) density plots were generated by first binning the data (using a custom build Excel spreadsheet, available on request) in 96 volumes {X [3 x $^{4}/_{3}$ Å], Y [8 x $^{10}/_{8}$ Å], Z [4 x $^{5}/_{4}$ Å]}. The percentage of the total that each volume contains was computed by dividing the number of data in a certain volume by the total amount of data. This density information was projected onto the centre of each volume using Orgin Pro 8. The size and colour of the spheres in the resulting plots are a visual representation of the density of data, whereby red and larger is denser, empty and small is less dense. These plots are shown in Figure S5.

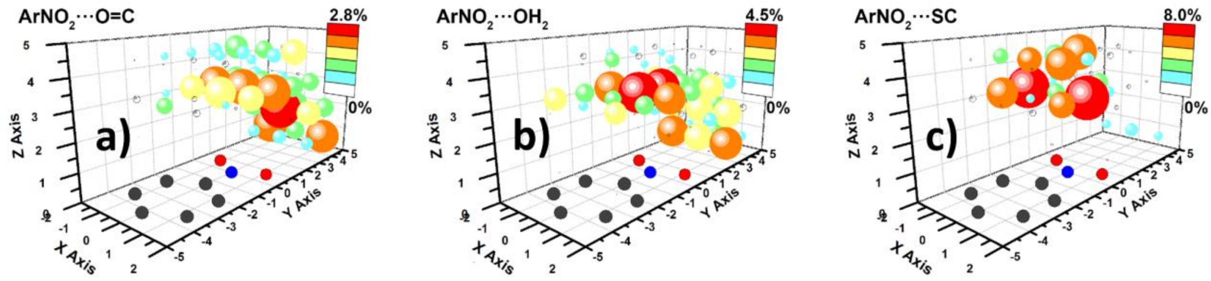

Figure S5. 4D density plots of the data extracted from the PDB characterized by N···interacting atom ≤ 5Å and |x| ≤ 2 Å for the interacting partner atoms: a) C=O Oxygen with 3,374 hits; b) H_2O Oxygen with 3,250 hits; and c) CSR Sulphur (R can be any atom) with 434 hits.

The {X,Y,Z} coordinates of the model, together with the standard van der Waals radii of C (1.70 Å), N (1.55 Å) and O (1.52 Å) were used to generate a model as a single body 'part' file (.ipt) using Autodesk Inventor® Professional 2016 (by using mm instead of Å). Similarly, a half spherical segment was created (radius 5 mm, width 4 mm and length 10 mm). 5 mm high half cylinders of increasing radius (up to 5 mm) were also generated and trimmed so that their width (X-axis) was ≤ 4 mm. All these bodies were collected in an assembly file (.iam), properly alighted, and the half spherical segments were mirrored along the X-axis; the result is illustrated in Figure S3 with (trimmed) half cylinders of 1, 2, 3, 4 and 5 mm radius. These (trimmed) half cylinders are representative of volumes characterized by ≤ ***r***.

Using the 'Analyse Interference' option in Autodesk Inventor® Professional 2016 the interfering volumes between the model and the (trimmed) half cylinders could be derived. The volume difference between two such interfering volumes of incremental ***r***-values, say $\boldsymbol{r}_a$ and $\boldsymbol{r}_b$, thus represent the volume that the model occupies in between two values of ***r***, i.e. V_{model}. Similarly, the interfering volume between the half spherical segment and the (trimmed) half cylinders could be derived as a function of ***r***, from which the volume in between two ***r***-values as found within the half spherical segment could be derived, i.e. $V_{no\ model}$. The actual free volume in between two ***r***-values that a 'host' can occupy, i.e. V^{r}_{free}, is thus given $V_{no\ model} - V_{model}$. The total freely accessible volume, V^{total}_{free}, is naturally given the volume of the half spherical segment minus the volume of the model in the half spherical segment. The random (or volume) distribution as a function of ***r***, i.e. D^{r}_{chance}, is thus given by:

(7) $$D^{r}_{chance} = \frac{V^{r}_{free}}{V^{total}_{free}}$$

The actual distribution of the data, D^{r}_{data}, is naturally given by:

(8) $$D^{r}_{data} = \frac{N^{r}}{N^{total}}$$

Thus, the change corrected distribution of data, $P(r)$ is given by:

(9) $$P(r) = \frac{D^{r}_{data}}{D^{r}_{chance}}$$

For an accidental distribution, P should be unity across all ***r***-values; a P-value greater than unity is thus evidence of positive clustering (suggesting a favourable interaction), while P-values smaller than unity reflect a depletion of data (suggesting an unfavourable interaction). These $P(r)$ plots are sown in Figure S6.

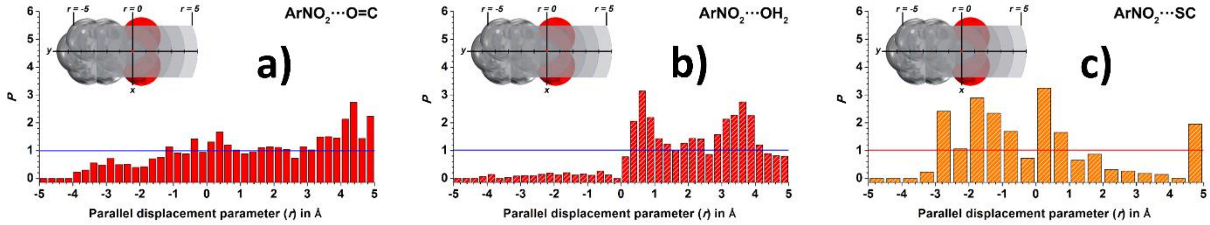

Figure S6. $P(r)$ plots of the data extracted from the PDB characterized by N···interacting atom ≤ 5Å and |x| ≤ 2 Å for the interacting partner atoms: a) C=O Oxygen with 3,374 hits; b) H_2O Oxygen with 3,250 hits; and c) CSR Sulphur (R can be any atom) with 434 hits. The inset figures are a guide to the eye and the straight lines at $P = 1$ indicate the distribution anticipated for a random scattering of the data.

The data further characterized by a parallel displacement parameter of ≤ 1 Å were further inspected to assess possible overlap of van der Waals shells. These data were plotted as percentages as a function of the van der Waals corrected N – interacting atom (O or S) distance (***d'***) in both absolute and cumulative fashion, as shown respectively as solid bars and empty circles in Figure S7 (See also Figure 2 in the main text).

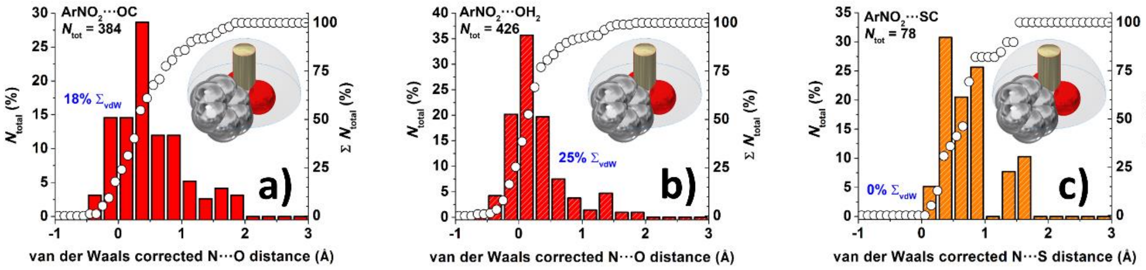

Figure S7. $N(d')$ plots where d'= van der Waals corrected N···interacting atom distance and N stands for the relative number of hits in absolute (solid bars) and cumulative (empty circles) fashion. These data were extracted from the PDB and characterized by N···interacting atom ≤ 5 Å, |x| ≤ 2 Å and r ≤ 1 Å, i.e. the gold region in the inset figures. The interacting partner atoms are: a) C=O Oxygen with 384 hits; b) H_2O Oxygen with 426 hits; and c) CSR Sulphur (R can be any atom) with 78 hits. The amount of data involved in apparent overlap of van der Waals shells is given in blue as '% Σ_{vdW}'.

3. Evaluation of close contact cases.

3.1. General approach and overview of data.

The four dimensional (4D) density plots and the directionality analyses were less clear than hoped for and likely underdetermined by the data. Hence, a further scrutiny was undertaken of the data. First the data was filtered for r ≤ 1.0 Å (column '$N_{r\leq1}$' in Table S3). Then the data was limited to hits with [^{NO2}N···X$^{water/CO/S}$] ≤ the sum of the van der Waals radii of N and O/S + 0.5 Å (column '$N_{\leq vdW+0.5}$' in Table S3, also highlighted in blue). These data contained a total of 170 unique hits, as is detailed in Table S4.

For all these hits the PDB structures and the articles in which they were published were collected and inspected manually. Instances where structural and/or functional relevance was suspected were processed further, leading to the cases highlighted in Figure S9 – Figure S36. The remaining structures were dismissed (although structural and/or functional significance cannot be ruled out).

In a typical case, a cartoon representation of the (aligned) protein structure(s) was generated and if a structure contained multiple chains these were aligned and cartoon representation generated. The (aligned) binding pockets of relevant ligands and/or selected residues around the nitro aromatic were also highlighted and geometric (distance) information is frequently given. When relevant, illustrations of comparative structures are given (sometimes not bearing a nitro aromatic ligand).

In all cases, a LigPlot+ (version 1.4)[11] plot was generated for the ligand involved in the π-hole contact and in case of multiple similar structures the one with the shortest contact distance was used. The Chemical structure(s) of the ligand(s) is also always given. Molecular Electrostatic Potential maps (MEPs) were computed with DFT at the BLYP[1] 6-31G*[2] level of theory as follows (using Spartan© 2014). The atomic coordinates of the nitro-bearing ligand was first lifted from the relevant PDB

structure and the structure was simplified and/or completed as necessary (e.g. by adding H-atoms). A DFT energy optimization was then performed ensuring that the measured atomic coordinates remained at their experimentally determined positions. A water molecule was then added in a co-planar hydrogen-bonding geometry with the nitro group to mimic the mimic the ligand being bound by the protein. The energy was again minimized (conserving the coordinates of measured atoms) and the MEPs of resulting structures are used in Figure S9 – Figure S36 with the indicated electropositive potentials of the nitro's π-hole in kcal/mol.

The contribution of the π-hole interactions to the total binding enthalpy for selected examples (Figure S9 – Figure S22) was estimated as follows. Cartesian coordinates of the ligand and relevant residues were first extracted from the PDB file. These structures were sculpted to generate a simple and reasonable model of the nitro aromatic and residues that might impact the π-hole interaction. All measured atoms were frozen, H-atoms were added where relevant and the positions of H-atoms were optimized at the BP86[1]-D3[12]/def2-TZVP[6] level of theory. The energy of resulting complexes was computed at the SCS-MP2[5]/def2-TZVP[6] level of theory (ΔE^1). The energy of an *in silico* mutation designed to eliminate only the π-hole interaction was computed as well (ΔE^2). The subtraction $\Delta E^1 - \Delta E^2$ then gives *a rough estimate* (e.g. the contribution of dispersion might also change) for the energetic contribution of the π-hole interaction (ΔΔE). In some cases the interacting residue was mutated and for all cases the nitro group was mutated to a nitroso moiety. As is illustrated in Figure S8, a nitroso aromatic is much like to a nitro aromatic in terms of sterics and electronics, yet it has no appreciable π-hole. Cartesian coordinates and ΔΔE values are given in section 3.9.

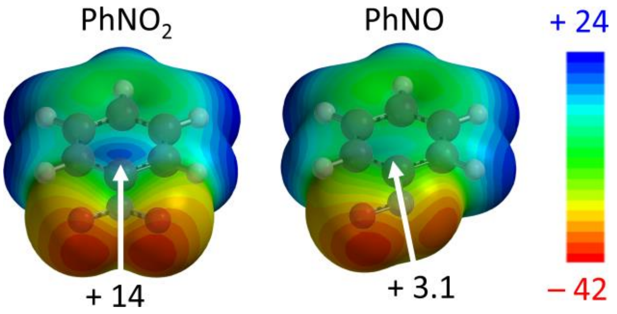

Figure S8. Molecular electrostatic potential maps of nitrobenzene (left) and nitrosobenzene (right) computed with density functional theory at the BLYP/6-31G* level of theory. The potential range from –42 (red) to +24 (blue) kcal/mol.

Table S4. List of unique hits found in the PDB with $[NO_2^{N}\cdots X^{CO/water/S}]$ distance of ≤ the sum of the van der Waals radii of N and O/S + 0.5 Å, and a parallel displacement (r) from the nitrate's N-atom of ≤ 1.0 Å (see also highlighted data in Table S3). The columns in table indicate the PDB structure ('PDB'), the nitro-bearing ligand ('Lig.') and residue ('Res.') involved in the hit, the chain ('Ch.') in which the unique hit was found, and the $[NO_2^{N}\cdots X^{water/CO/S}]$ distance ('*d* / Å') found.

PDB	Lig.	Res.	Ch.	*d* / Å	PDB	Lig.	Res.	Ch.	*d* / Å	PDB	Lig.	Res.	Ch.	*d* / Å
**Interacting atom is C=O Oxygen:**					4tki[13]	33E	1202	A	3.456	2oyq[14]	N5P	904	C	3.368
1d0c[15]	INE	765		3.190		33E	1202	D	3.407	2qp6[16]	MB1	800	A	3.271
	INE	766		3.136		33E	1203	B	3.504	2wbk[17]	M2F	1868	B	3.058
1d0o[15]	INE	765		3.235	4zbb[18]	GDN	301	C	3.513		M2F	1869	A	2.702
	INE	766		3.159	5del[19]	D59	603	A	3.193	3lm1[20]	LEC	134	C	3.400
1d0y[21]	ONP	999		3.016	**Interacting atom is H_2O Oxygen:**					3qwk[22]	X62	493	A	3.215
1e36[23]	TPY	280	B	3.051	1d1a[21]	DAE	999		3.390	3qx2[22]	X63	300	A	3.068
1e37[23]	TPY	280	B	3.029	1e4i[24]	NFG	3000	A	3.309	3qx4[22]	X4B	454	A	3.290
1e38[23]	TPY	280	B	3.075	1eei[25]	GAA	504		2.803	3qzh[22]	X69	839	A	2.954
1gaf[26]	NPE	1		3.365		GAA	505		3.269	3qzi[22]	X72	512	A	3.083
1lop[27]	XAPAX	0	B	3.392		GAA	506		2.886	3r1q[22]	X75	616	A	2.972
1m9r[28]	INE	902		3.422		GAA	507		3.333	3r28[22]	XA0	782	A	2.984
	INE	903		3.135	1gnr[29]	CAG	167		2.450	3r6x[22]	X84	778	A	3.085
1vbs[30]	AXPFX	2	B	3.258	1gvs[31]	TNF	500	A	3.122	3r71[22]	X86	800	A	3.050
2c5p[32]	CK7	1297	A	3.370	1jyv[33]	145	2001	B	3.311	3r73[22]	X87	920	A	3.062
2f9b[34]	N1H	246		3.388	1jqy[35]	A32	104	G	3.146	3r7e[22]	X88	919	A	3.247
2fhl[36]	BNI	502		2.971		A32	104	W	2.851	3r7i[22]	X9I	505	A	2.884
2w5u[37]	IC3	165	A	2.647		A32	104	X	2.776	3r7u[22]	X96	523	A	3.290
2w7x[38]	D1A	601	A	2.814		A32	104	X	2.758	3r7v[22]	Z02	490	A	3.332
2wev[32]	CK7	1298	C	3.326		A32	104	X	2.776	3r7y[22]	Z04	484	A	2.879
	CK7	1298	C	3.213		A32	104	X	2.758	3r83[22]	Z14	589	A	2.916
2xk9[39]	XK9	1511	A	2.832		A32	104	X	2.776	3r9n[40]	Z68	529	A	2.920
2y59[41]	ZA3	500	B	3.379		A32	104	X	2.758	3rai[22]	X85	923	A	3.108
	ZA3	500	C	3.467		A32	104	X	2.776	3rm6[22]	18Z	478	A	3.134
2ycq[39]	UPX	600	A	2.900		A32	104	X	2.758	3rpo[22]	24Z	778	A	3.029
2yir[42]	YIR	600	A	2.948		A32	104	Y	3.460	3vo0[43]	DNF	502	A	3.284
2zhn[44]	RPN	1168	A	3.054	1llr[45]	FNG	104	E	3.064		DNF	502	A	3.257
3cht[46]	4NB	502	A	3.021		FNG	104	F	3.060	4d43[47]	9W7	1258	D	3.157
3g4g[48]	D71	901	A	3.351		FNG	104	G	3.133		9W7	1259	H	3.370
	D71	902	B	3.450		FNG	104	H	3.105	4kjx[49]	XXH	305	B	3.342
	D71	904	D	3.352	1lt6[50]	GAA	104	F	2.868	4m81[51]	PNW	502	A	3.555
3g4i[48]	D71	901	A	3.351		GAA	104	M	3.424		PNW	502	A	3.555
3h0b[52]	B35	449	A	3.548		GAA	104	N	3.414		PNW	502	A	3.260
3m3o[53]	NPO	240	A	2.984	1pzi[54]	1DM	105		3.299		PNW	502	A	3.555
3mk0[55]	NPO	913	A	3.295		1DM	106		3.069		PNW	502	A	3.260
3mk1[55]	NPO	913	A	3.344		1DM	107		3.195		PNW	502	A	3.555
3qtx[40]	X43	299	A	3.373		1DM	108		3.118		PNW	502	A	3.260
3sgv[56]	2BJ	2001	A	3.028	1pzj[54]	J15	105		3.237	4mik[57]	JIL	301	B	2.613
3vpl[58]	DNX	1004	A	3.078		J15	106		3.176	4n8s[59]	XXH	302	B	2.878
4ek8[60]	16K	301	A	2.951	1rcv[61]	BV1	105		3.241	4nfl[62]	2JW	303	A	3.548
4itz[63]	NIT	0	C	3.236	1rd9[61]	BV2	105		3.144	4o4r[64]	20V	601	C	3.430
4ki7[65]	1R2	201	B	3.464		BV2	107		3.345	4rv8[66]	I13	501	A	3.250
	1R2	201	C	3.330		BV2	108		3.134	4s0x[67]	XXH	306	B	3.375
	1R2	201	H	3.493	1rdp[61]	BV3	104		3.160	4tpw[68]	33R	302	A	3.530
4kiw[65]	KIW	201	H	3.268		BV3	105		3.298	4woh[69]	4NP	201	A	3.197
	KIW	201	J	3.371		BV3	106		3.137	4zbb[18]	GDN	301	A	3.215
	KIW	500	A	3.350		BV3	107		3.287		GDN	301	C	2.973
4lq3[70]	20V	601	A	2.857		BV3	108		3.259		GDN	301	D	2.850
4ndz[71]	NPO	2004	D	2.921	1rf2[61]	BV4	104		3.197	**Interacting atom is RSC Sulfur:**				
4nvh[72]	2NB	302	B	3.084		BV4	106		3.188	1y2j[73]	7DE	102		3.695
4oad[74]	CLM	206	A	3.479		BV4	107		3.315	1y2k[73]	7DE	601		3.638
4oae[74]	CLM	208	A	3.467		BV4	108		3.223		7DE	602		3.658
4oxy[75]	1TN	302	A	3.564	1uyq[76]	NFG	3000	A	3.387	2cl6[77]	CAG	167	X	3.494
4qo7[78]	36V	803	A	3.376	1y2k[73]	7DE	601		3.417	2zvp[79]	NPO	1202	X	3.731
4qs5[80]	1DF	402	A	3.544		7DE	602		3.417	2zyv[81]	NPO	1602	X	3.762
4qs6[82]	1DF	404	A	3.375	1zwp[83]	NIM	401		2.884	2zyw[81]	NPO	602	X	3.679
4r6s[84]	3K2	501	B	2.806	2hj4[85]	PNZ	1202	D	3.144	3f1o[86]	2XY	1	A	3.618
						PNZ	1204	H	3.498	3h7w[87]	18	1	A	3.517

3.2. Examples where a π-hole interaction is highly preserved within similar structures.

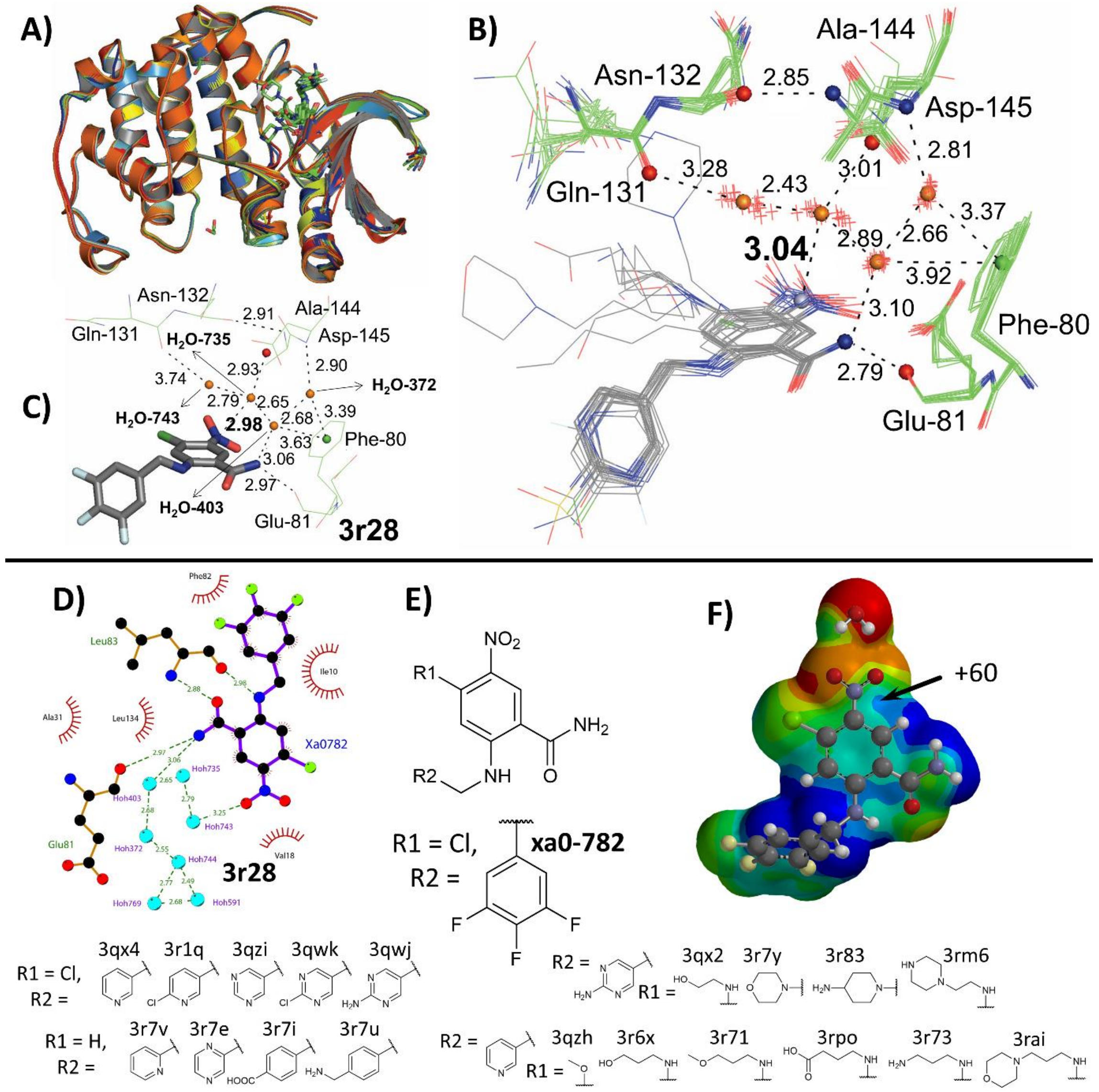

Figure S9. Illustrations of possible Ar-NO_2 π-hole interactions involving the 20 protein structures 3r28, 3qx4, 3r1q, 3qzi, 3qwk, 3qwj, 3r7v, 3r7e, 3r7i, 3r7u, 3qx2, 3r7y, 3r83, 3rm6, 3qzh, 3r6x, 3r71, 3rpo, 3rai and 3r73.[22] **A)** Cartoon representation of aligned protein structures with the ligands in capped-sticks mode; **B)** Capped stick representation of aligned ligands (carbon backbone in grey) with nearby water molecules and the residues in close contact with these water molecules. The small solid spheres represent average atomic coordinates ('dummy atoms') and the distances shown are measured between these average positions. One group of water molecules is consistently found near the π-hole region of the nitro aromatic with an average $^{water}O\cdots N^{NO2}$ distance of 3.07 Å, which is 0.05 Å within the sum of the van der Waals radii of N+O (1.55 + 1.52 = 3.07 Å) and thus indicative of a π-hole interaction; **C)** Partial binding pocket of ligand xa0-782 (see also E) as found within 3r28. The same residues and water molecules as in B are shown. The $^{water\text{-}735}O\cdots N^{NO2}$ distance of 2.98 Å is 0.09 Å within the sum of the van der Waals radii of N+O (1.55 + 1.52 = 3.07 Å) and thus indicative of a π-hole interaction; **D)** LigPlus plot of ligand xa0-782 in 3r28. Polar contacts are shown as green striped lines with bonding distance. The polar residues are shown as balls and sticks and the apolar residues as red eye-lashes; **E)** Schematic drawing of the chemical structures of the ligands, with ligand xa0-782 highlighted (used as example in C and D); **F)** The molecular electrostatic potential map of xa0-782 (atomic coordinates extracted from 3r28) with the nitro group hydrogen bonded to a water molecule to mimic the ligand being bound by the protein. The colour code spans from +150 (blue) to –150 kJ/mol (red) in eight bands. All distances are in Angstroms (Å). **NB:** These structures are part of a series of 38 crystal structures of cyclin-dependent kinase 2 in complex with small molecule inhibitors. No binding data are available, as all these structures were only published in the PDB. Of these 38 structures, 25 structures had a ligand with the general structure as shown in E, and the 20 structures used here had a very similar binding pocket.[22]). See also Figure 3 (left) in the main text.

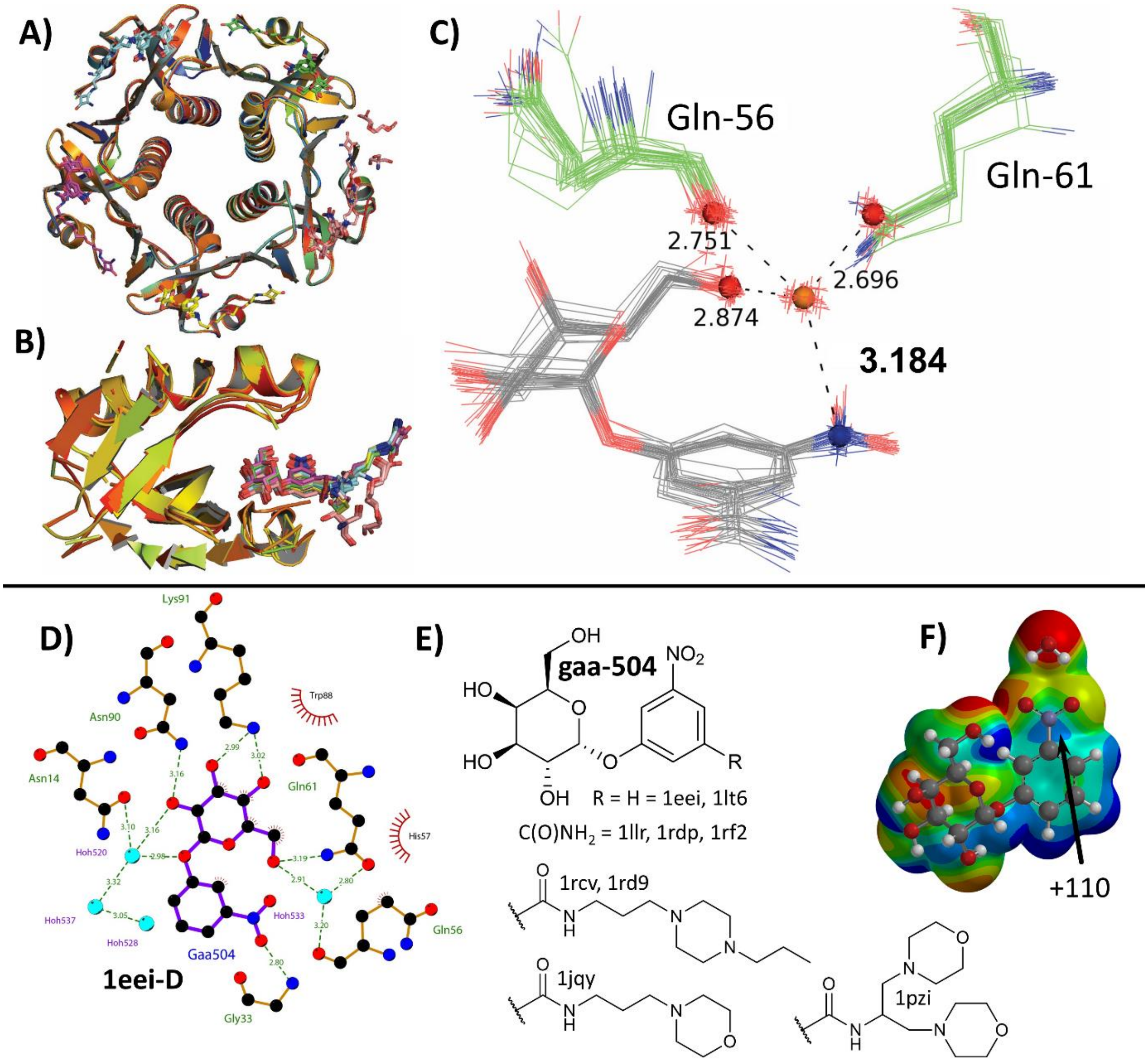

Figure S10. Illustrations of possible Ar-NO$_2$ π-hole interactions as found within protein structures 1eei,[25] 1lt6,[50] 1llr,[45] 1rdp,[61] 1rf2,[61] 1rcv,[61] 1rd9,[61] 1jqy[35] and 1pzi.[54] **A)** Cartoon representation of all twelve aligned pentameric protein structures (1lt6 contains two pentamers and 1jqy contains three pentamers); **B)** Cartoon representation of all 60 protein chains aligned; **C)** Out of the 60 binding pockets (aligned in B), 45 contained a water molecule and out of those 35 had a similarly oriented ligand. In these structures, the water molecule was encapsulated by two glutamine residues (56 and 61) and the ligand (H-bonding and π-bonding interactions). These structures were overlaid, illustrating that this water binding mode is highly preserved. The distances shown are averages, calculated by creating the average dummy atoms (shown as small spheres) of the atoms involved in the interactions. Note that the average $^{water}O\cdots N^{NO2}$ distance of 3.184 Å is very close to the sum of the van der Waals radii of N+O (1.55 + 1.52 = 3.07 Å) and in some structures this distance is significantly below this benchmark (e.g. 2.803 Å in chain D of 1eei). **D)** LigPlus plot of ligand gaa-504 in its binding pocket within chain D of 1eei. Polar contacts are shown as green striped lines with bonding distance. The polar residues are shown as balls and sticks and the apolar residues as red eye-lashes; **E)** Schematic drawing of the chemical structures of the ligands such as gaa-504 within the structures. **F)** The molecular electrostatic potential map of gaa-504 (atomic coordinates extracted from the PDB) with the nitro group hydrogen bonded to a water molecule to mimic the ligand being bound by the protein. The colour code spans from +150 (blue) to –150 kJ/mol (red) in eight bands. All distances are in Angstroms (Å). See also Figure 3 (middle) in the main text.

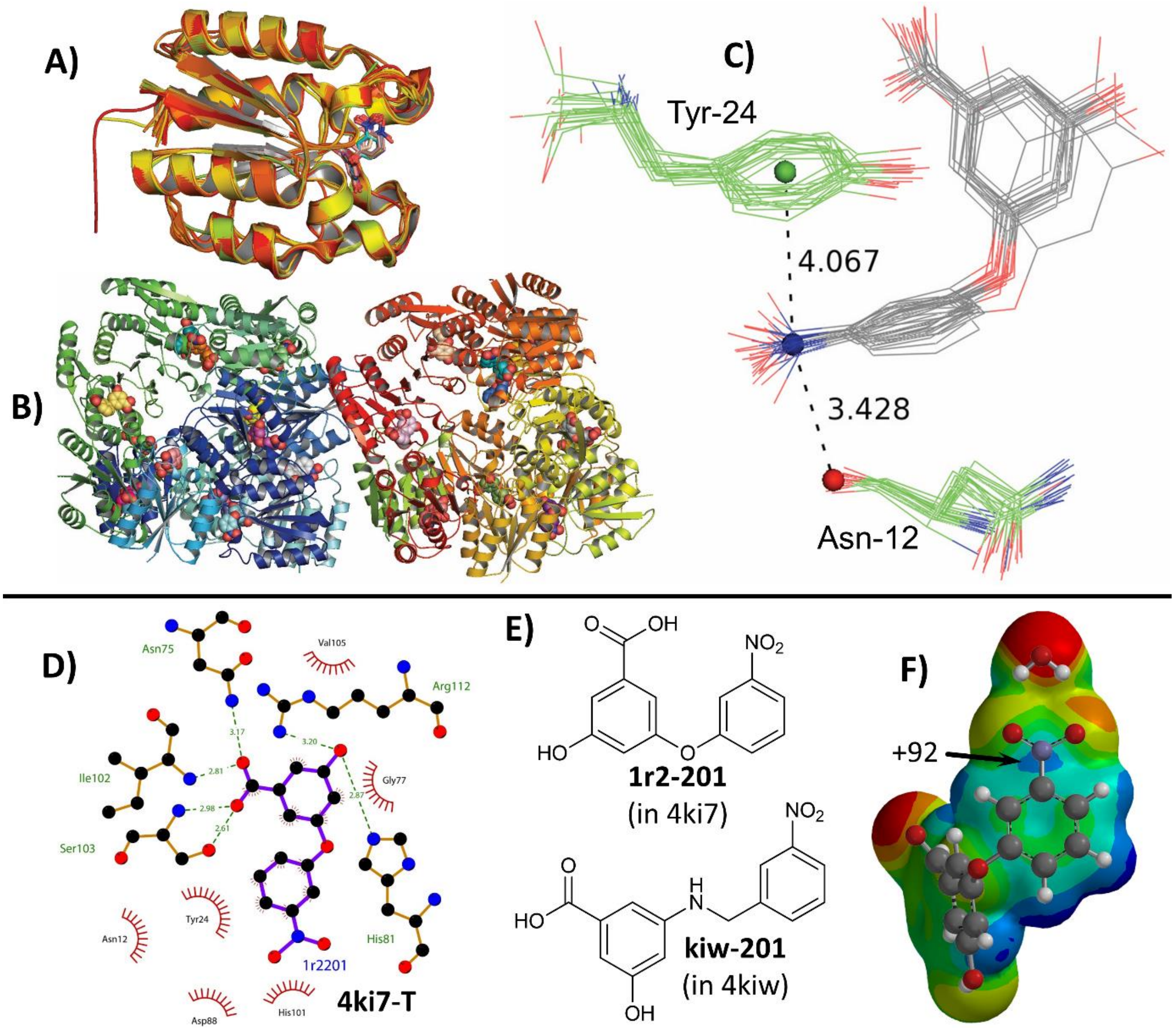

Figure S11. Illustrations of possible Ar-NO_2 π-hole interactions as found within protein structures 4ki7 and 4kiw.[65] **A)** Cartoon representation of all 48 chains aligned (24 in each structure); **B)** Cartoon representation of aligned structures; **C)** In 4ki7 there are 24 chains within which are 20 similarly aligned isostructural ligands (1r2-201, see also E). The nitroarene group of this ligand is sandwiched in between tyrosine (24) and asparagine (12) residues and the distances displayed are averages calculated by creating the average dummy atoms (shown as small spheres) of the atoms involved in the interactions. The shortest $^{Asn\text{-}12}O\cdots N^{NO2}$ distance is 3.051 Å involving the ligand in chain T. The same binding mode is observed for ligand kiw-201 in (see also E) in 18 binding pockets of the 24 isostructural chains in 4kiw with average $^{Tyr\text{-}24}$centroid$\cdots N^{NO2}$ and $^{Asn\text{-}12}O\cdots N^{NO2}$ distances of 4.652 and 3.586 Å respectively and the shortest $^{Asn\text{-}12}O\cdots N^{NO2}$ distances is 3.161 Å involving the ligand in chain V (not shown). **D)** LigPlus plot of ligand 1r2-201 in its binding pocket within chain T 4ki7. Polar contacts are shown as green striped lines with bonding distance. The polar residues are shown as balls and sticks and the apolar residues as red eye-lashes; **E)** Schematic drawing of the chemical structures of ligands 1r2-201 and kiw-201. **F)** The molecular electrostatic potential map of 1r2-201 (atomic coordinates extracted from the PDB) with the nitro group hydrogen bonded to a water molecule to mimic the ligand being bound by the protein. The colour code spans from +150 (blue) to –150 kJ/mol (red) in eight bands. All distances are in Angstroms (Å). See also Figure 3 (right) in the main text. **NB:** in the original paper it is reported that –NO_2 bearing ligands are two times better type II dehydroquinase (from *Mycobacterium tuberculosis*) inhibitors than ligands bearing an –NH_2 functionality (see ligand 41a *vs* 41b and ligand 44a *vs* 44b in Table 1).[65]

3.3. Examples of likely functional relevance of π-hole interactions with H_2O.

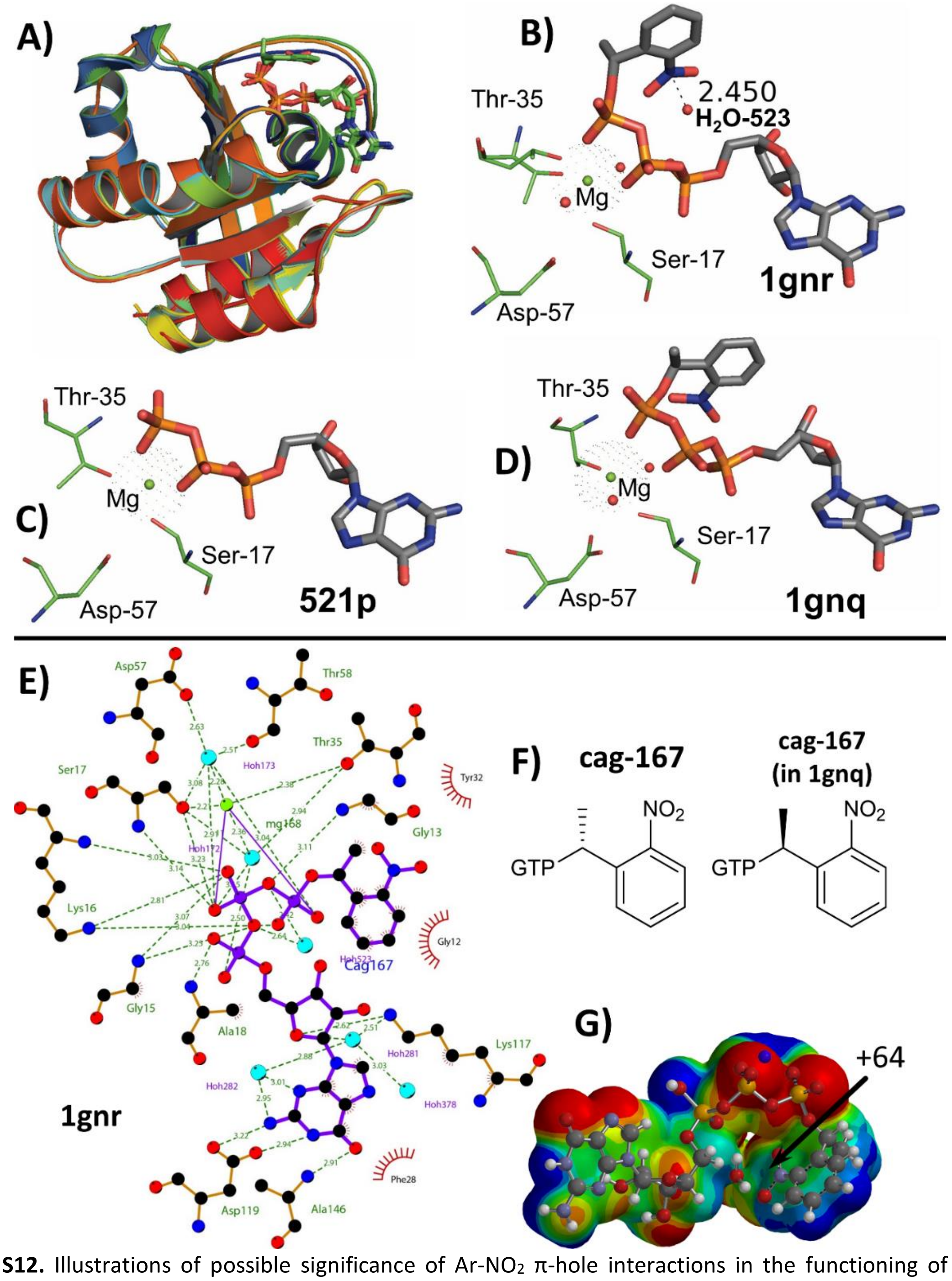

Figure S12. Illustrations of possible significance of Ar-NO_2 π-hole interactions in the functioning of protein structure 1grn.[29] **A)** Cartoon representation of overlaid protein structures of oncogene product p21$^{\text{H-ras}}$ in complex with GTP ligands. **B**–**D**) illustrations of these three different GTP ligands and the adjacent magnesium binding pocket. Note that three ligands in 1grn (B) and 1gnq (D) are isomers and that 521p (C) is regular GTP. In structure 1gnr (B) there is a water molecule that seems encapsulated by the ligand with a very short ^{water}O···N$^{\text{NO2}}$ distance of 2.450 Å, well within the sum of the van der Waals radii of N+O (1.55 + 1.52 = 3.07 Å). **E)** LigPlus plot of ligand cag-167 in its binding pocket of 1grn. Polar contacts are shown as green striped lines with bonding distance. The polar residues are shown as balls and sticks and the apolar residues as red eye-lashes; **F)** Schematic drawing of the chemical structures of the isomeric ligands cag-167 in 1grn and 1gnq. **G)** The molecular electrostatic potential map of cag-167 in 1grn (atomic coordinates extracted from the PDB) with the nitro group hydrogen bonded to a water molecule to mimic the ligand being bound by the protein. The colour code spans from +150 (blue) to –125 kJ/mol (red) in eight bands. All distances are in Angstroms (Å). See also Figure 4 and the main text.

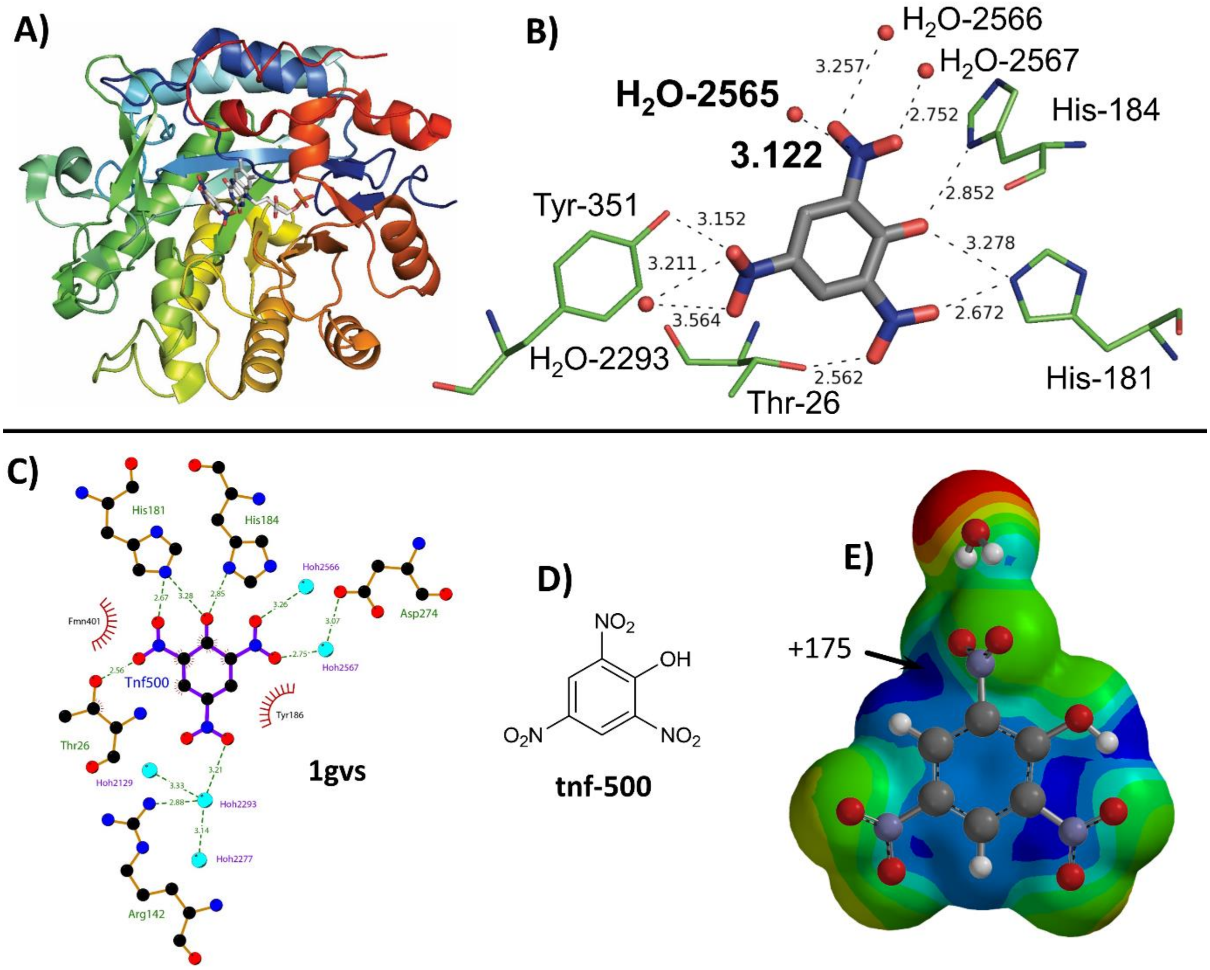

Figure S13. Illustrations of possible relevance of Ar-NO_2 π-hole interactions involving protein structure 1gvs.[31] **A)** Cartoon representation of the entire protein structure; **B)** Capped stick representation of the ligand tnf-500 (in grey, see also D) surrounded by the residues in the binding pocket that are H-bonded to the ligand (in green). One water molecule (H_2O-2565) in close to one of the ligands N-atoms, possibly held in place by combined π-bonding (to –NO_2) and H-bonding (to the orto C–H). **C)** LigPlus plot of ligand tnf-500 in its binding pocket in 1gvs. Polar contacts are shown as green striped lines with bonding distance. The polar residues are shown as balls and sticks and the apolar residues as red eye-lashes; **D)** Schematic drawing of the chemical structures of ligands tnf-500. **E)** The molecular electrostatic potential map of tnf-500 (atomic coordinates extracted from the PDB) with the nitro group hydrogen bonded to a water molecule to mimic the ligand being bound by the protein. The colour code spans from +125 (blue) to –125 kJ/mol (red) in eight bands. All distances are in Angstroms (Å).

3.4. Examples of likely functional relevance of π-hole interactions with C=O.

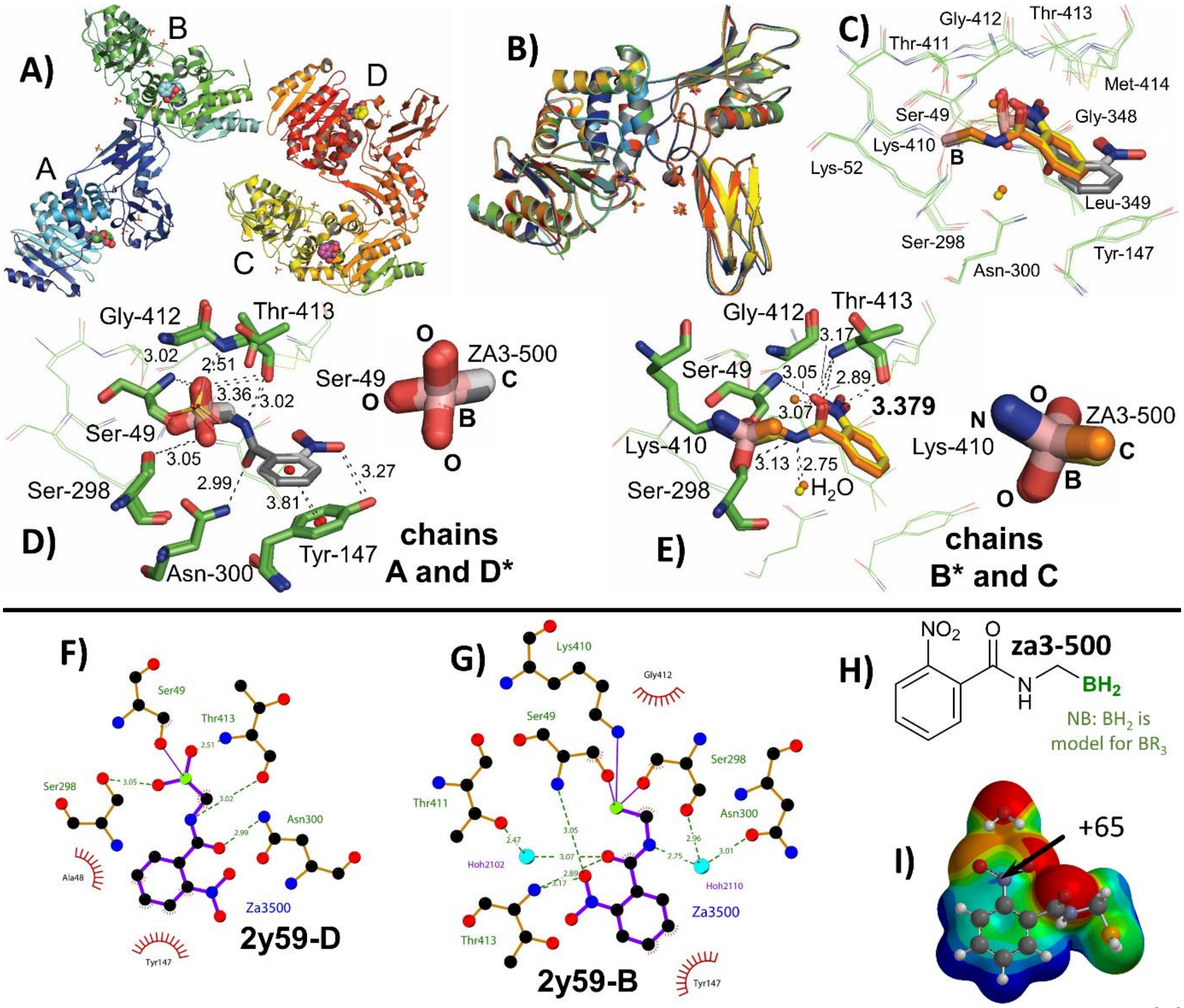

Figure S14. Illustrations of possible relevance of Ar-NO_2 π-hole interactions involving protein structure 2y59.[41] **A)** Cartoon representation of the entire protein structure; **B)** Cartoon representation of aligned chains A–D; **C)** Capped stick representation of aligned binding pockets of chains A–D (4 Å residues around the ligands) and their ligands (za3-500, see also H), showing that the binding pocket is highly preserved throughout the series; **D)** Capped stick representation of aligned binding pockets (4 Å residues around the ligands) of chains A and D wherein the ligand is covalently bound to Serine-49 via the ligands boronate (CBO_3). The residues involved in polar contacts are shown as thicker sticks, as is tyrosine-147 which is π-π stacking with the ligand's nitroaryl moiety. The distances shown are those found in chain D (hence the asterisk in the figure); **E)** Capped stick representation of aligned binding pockets (4 Å residues around the ligands) of chains B and C wherein the ligand is covalently bound to Serine-49, Serine-298 and Lysene-410 via the ligands boronate ($CBNO_2$). The residues involved in polar contacts are shown as thicker sticks and the distances shown are those found in chain B (hence the asterisk in the figure). Threonine-413 is H-bonded to the nitro moiety (NH···O) and its carbonyl-O is in close proximity of the ligands nitro π-hole region with $^{Thr\text{-}413}O\cdots N^{NO2}$ distance of 3.379 Å; **F)** and **G)** LigPlus plot of ligand za3-500 in chains D and B of 2y59 respectively. Polar contacts are shown as green striped lines with bonding distance. The polar residues are shown as balls and sticks and the apolar residues as red eye-lashes; **H)** Schematic drawing of the chemical structures of ligands za3-500, wherein the boranate is generalised as unbound –BH_2; **I)** The molecular electrostatic potential map of za3-500 (atomic coordinates extracted from chain B of 2y59 and the boronate simplified as neutral –BH_2) with the nitro group hydrogen bonded to a water molecule to mimic the ligand being bound by the protein. The colour code spans from +125 (blue) to –125 kJ/mol (red) in eight bands. All distances are in Angstroms (Å). See also Figure 4 and the main text.

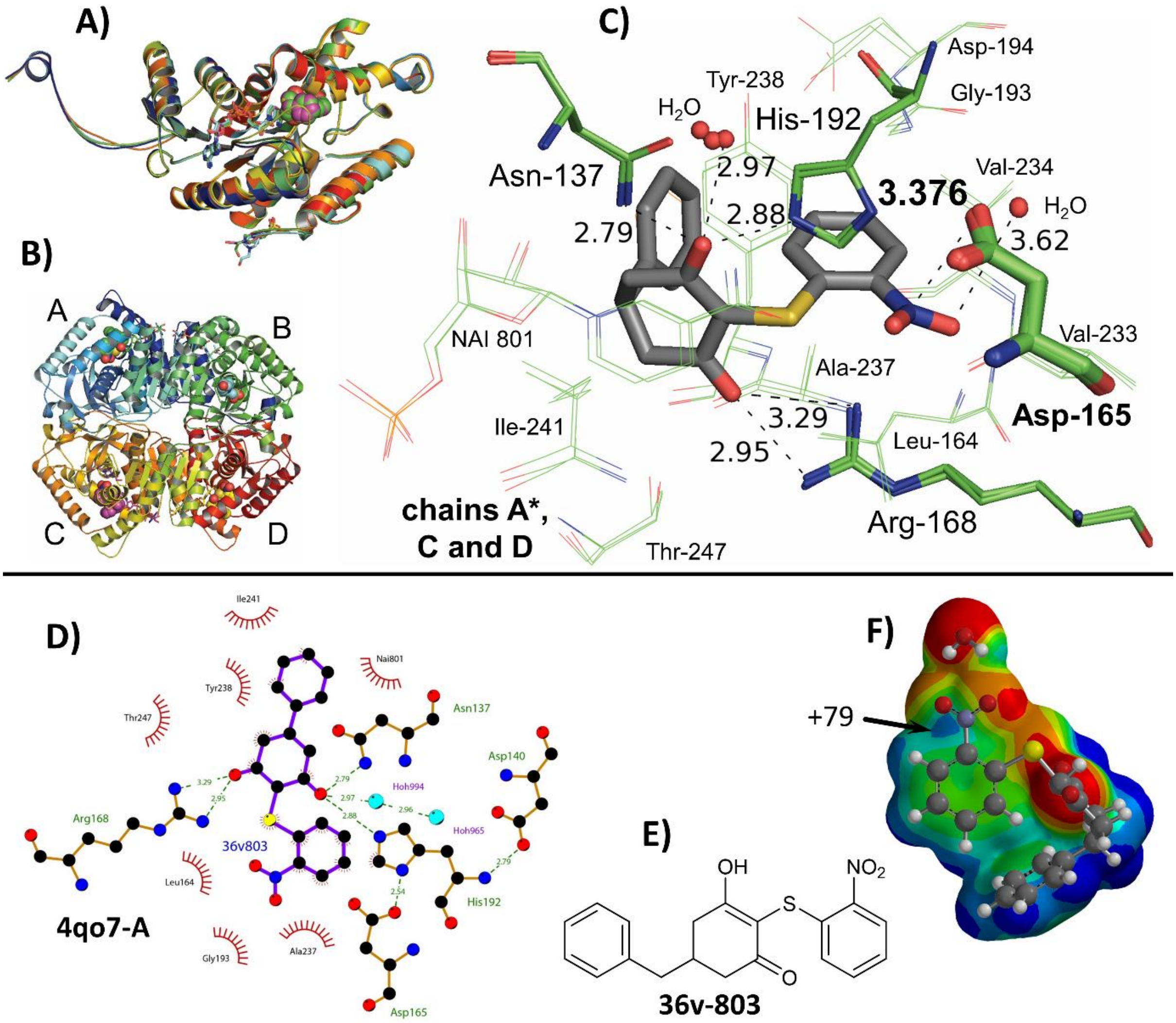

Figure S15. Illustrations of possible relevance of Ar-NO₂ π-hole interactions involving protein structure 4qo7.[78] **A)** Cartoon representation of aligned chains A–D; **B)** Cartoon representation of the entire protein structure; **C)** Capped stick representation of aligned binding pockets of chains A, C and D (4 Å residues around the ligands; chain B is empty) and their ligands (36v-803, see also E), showing that the binding pocket is highly preserved throughout the chains. The residues involved in polar contacts are shown as thicker sticks and the distances shown concern chain A (hence the asterisk in the figure). Asparganine-165, shown as the thickest sticks, is in close proximity to the ligands nitro π-hole region with an $^{Asp\text{-}165}O\cdots N^{NO2}$ distance of 3.376 Å; **D)** LigPlus plot of ligand 36v-803 in chains A 4q07. Polar contacts are shown as green striped lines with bonding distances. The polar residues are shown as balls and sticks and the apolar residues as red eye-lashes; **E)** Schematic drawing of the chemical structures of ligands 36v-803; **F)** The molecular electrostatic potential map of 36v-803 (atomic coordinates extracted from chain A of 4qo7 with the nitro group hydrogen bonded to a water molecule to mimic the ligand being bound by the protein. The colour code spans from +125 (blue) to –125 kJ/mol (red) in eight bands. All distances are in Angstroms (Å). See also Figure 4 and the main text.

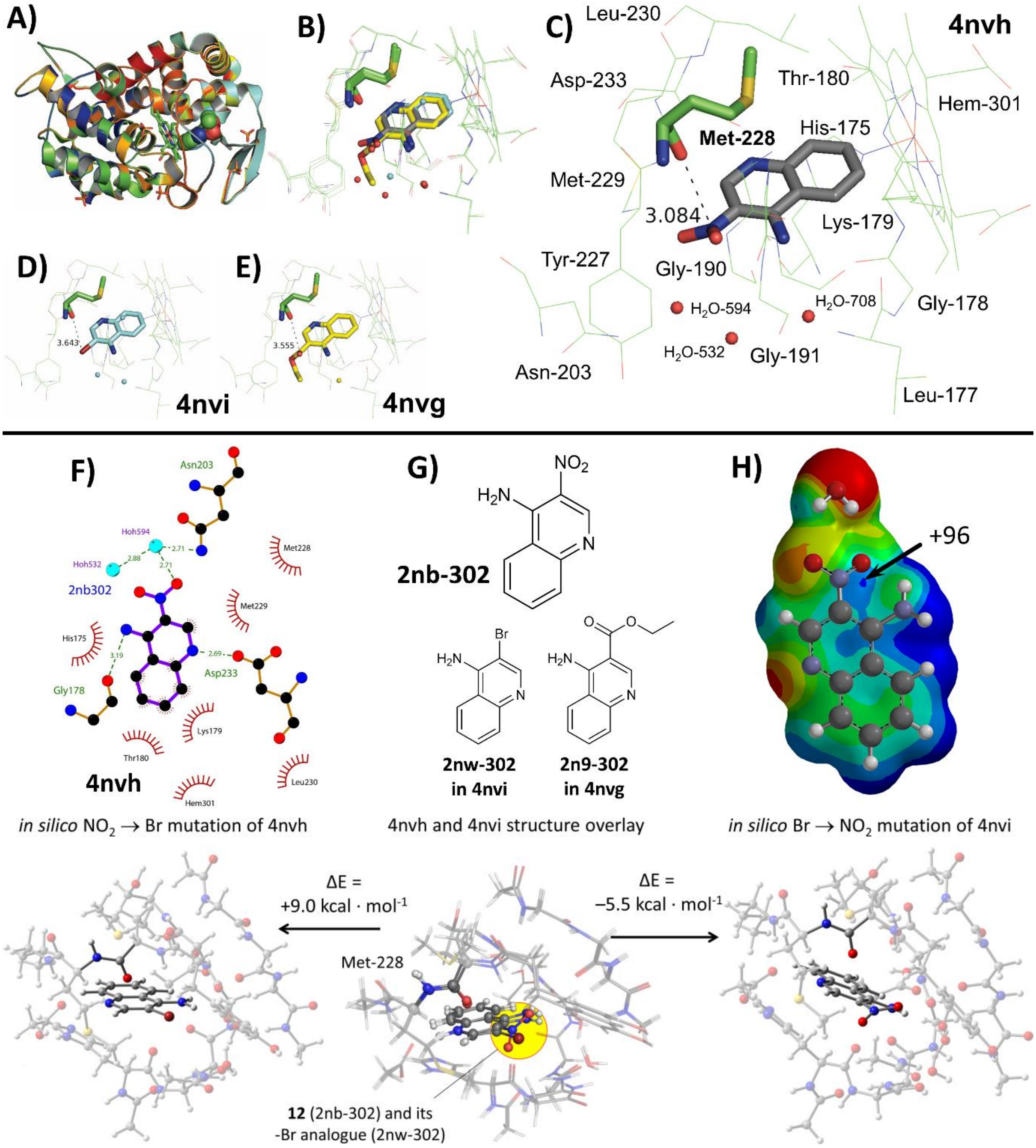

Figure S16-1. Illustrations of possible relevance of Ar-NO_2 π-hole interactions involving protein structure 4nvh compared to structures 4nvi and 4nvg.[72] **A)** Cartoon representation of the entire protein structures aligned with one another; **B)** Capped stick representation of aligned binding pockets (4 Å residues around the ligands) and their ligands (2nb-302, 2nw-302 and 2n9-302, see also G), showing that the binding pocket is highly preserved throughout the series. The Methionine residues close to the nitrate/bromo/ester moieties is shown as thicker sticks; **C)** The binding pocket of 2nb-302 in 4nvh (4 Å residues around the ligands) with a short $^{Met\text{-}228}O\cdots N^{NO2}$ distance of 3.084 Å (nearly within the sum of the van der Waals radii of N+O (1.55 + 1.52 = 3.07 Å)); **D)** and **E)** are idem as C), but for 2nw-302 in 4nvi and 2n9-302 in 4nvg. The $^{Met\text{-}228}O\cdots Br$ (3.643 Å) and $^{Met\text{-}228}O\cdots C^{ester}$ (3.555 Å) distance are significantly longer than the $^{Met\text{-}228}O\cdots N^{NO2}$ distance highlighted in C); **F)** LigPlus plot of ligand 2nb-302 in its binding pocket in 4nvh. Polar contacts are shown as green striped lines with bonding distance. The polar residues are shown as balls and sticks and the apolar residues as red eye-lashes; **G)** Schematic drawing of the chemical structures of ligands 2nb-302, 2nw-302 and 2n9-302; **H)** The molecular electrostatic potential map of 2nb-302 (atomic coordinates extracted from the PDB) with the nitro group hydrogen bonded to a water molecule to mimic the ligand being bound by the protein. The colour code spans from +125 (blue) to –150 kJ/mol (red) in eight bands. All distances are in Angstroms (Å). See also Figures 4 and 5 (left) in the main text.

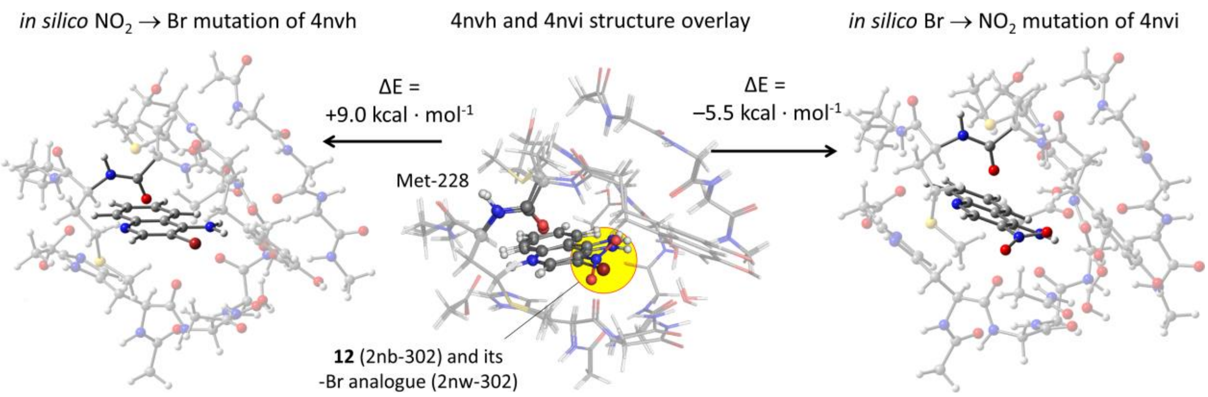

Figure S16-2. Computational evaluation (SCS-MP2/def2-SVP) of model (truncated) binding pockets of ligand **12** in structure 4nvh and its -Br analogue in structure 4nvi. Centre: structure overlay of model binding pockets. Four water molecules that were present in both binding pockets are included as well (H_2O-428, 662, 664 and 723). Left: model where the nitro group in 4nvh has been replaced by a bromine group. Right: model where the bromide group in 4nvi has been replaced by a nitro moiety. The energies are relative to the central models. When omitting the water molecules, the energy differences are +12.3 kcal·mol^{-1} for 4NVH and –10.9 kcal·mol^{-1}. See note [b] in the manuscript for computational details and see section 3.9 for the atomic coordinates of all eight computed binding pockets (4nvh, 4nvh$^{\rightarrow Br}$, 4nvi, 4nvi$^{\rightarrow NO_2}$, and their non-hydrates analogues).

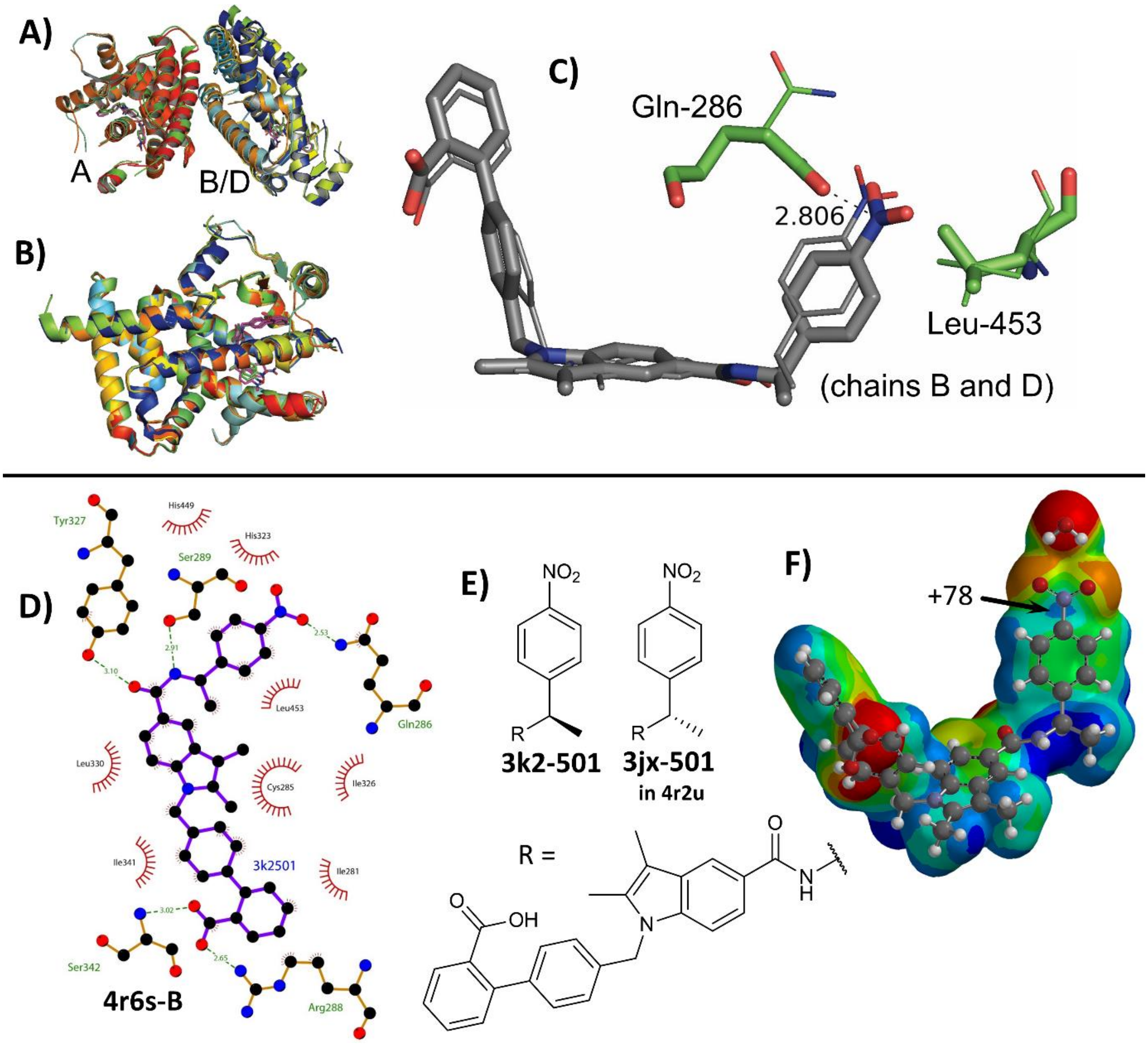

Figure S17. Illustrations of possible functionality of Ar-NO_2 π-hole interactions as found within protein structures 4r6s and 4r2u.[84] **A)** Cartoon representation of both structures aligned (4r6s has two chains labelled A and B, while 4r2u has two chains labelled A and D); **B)** Cartoon representation of all four chains aligned; **C)** The stereoisomeric ligands (see also E) within chain B of 4r6s (thick sticks) and chain D of 4r2u (narrow sticks) which have a glutamine (286) and leucine (453) residue in close proximity of their nitro-moiety. For the isomer in structure 4r6s a very close $^{Gln\text{-}286}O\cdots N^{NO2}$ distance of 2.806 Å is observed, which is well within the sum of the van der Waals radii of N+O (1.55 + 1.52 = 3.07 Å). This is not the case in 4r2u, where the glutamine residue is actually facing away from the Ar-NO_2 moiety. **D)** LigPlus plot of ligand 3k2-501 in its binding pocket within chain B of 4r6s. Polar contacts are shown as green striped lines with bonding distance. The polar residues are shown as balls and sticks and the apolar residues as red eye-lashes; **E)** Schematic drawing of the chemical structures of ligands 3k2-501 and 3jx-501. **F)** The molecular electrostatic potential map of 3k2-501 (atomic coordinates extracted from the PDB) with the nitro group hydrogen bonded to a water molecule to mimic the ligand being bound by the protein. The colour code spans from +125 (blue) to –125 kJ/mol (red) in eight bands. All distances are in Angstroms (Å). See also Figure 4 and the main text.

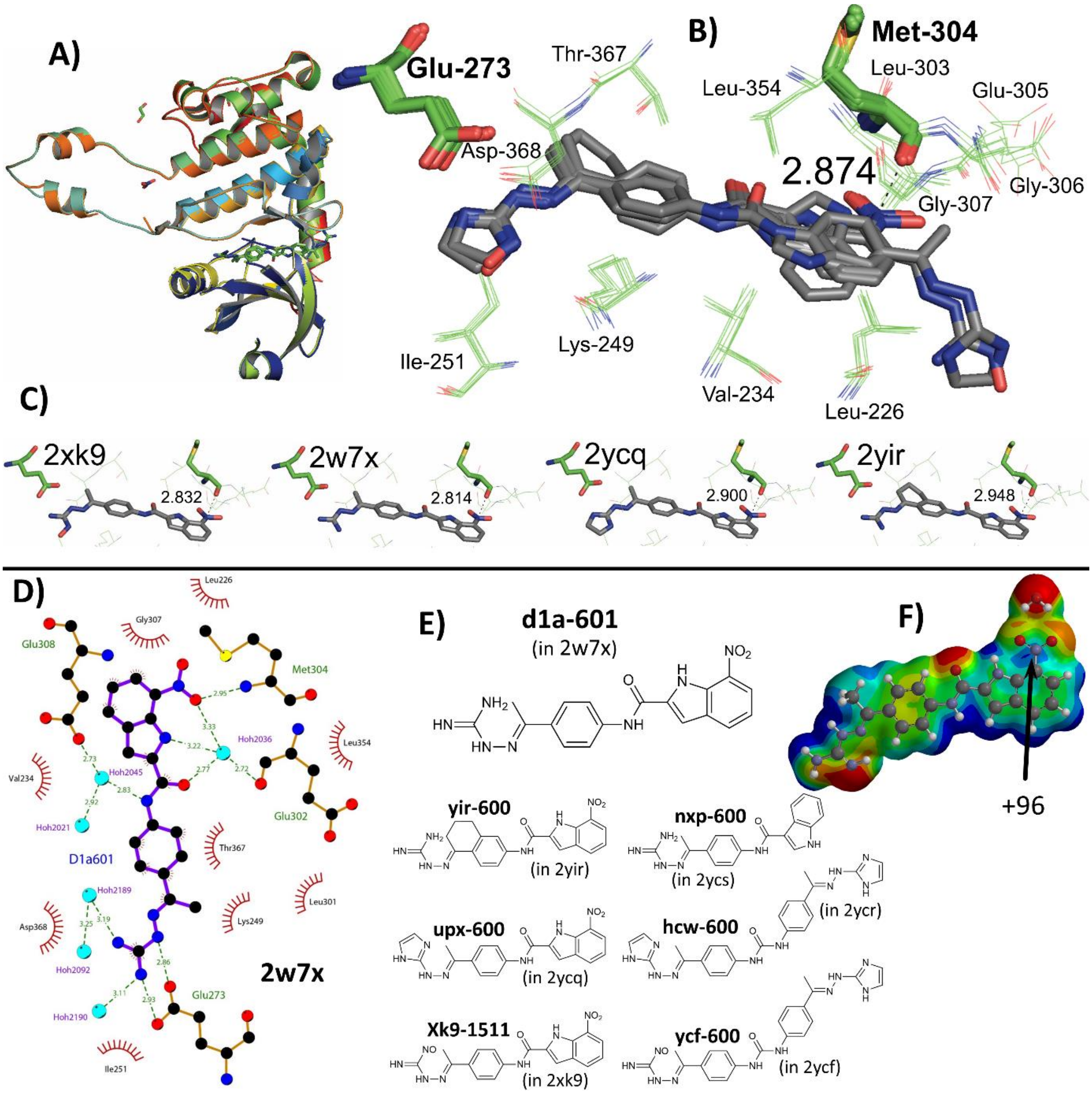

Figure S18. Illustrations of possible relevance of Ar-NO_2 π-hole interactions involving protein structures 2w7x,[38] 2yir,[42] 2ycq,[39] 2xk9[39] and (in which the ligands bear the –NO_2 functionality), and 2ycr, 2ycs and 2ycf[39] (in which the ligands do not bear the –NO_2 functionality).[38-39], [42] **A)** Cartoon representation of aligned structures; **B)** Capped stick representation of aligned binding pockets (4 Å residues around the ligands) and their ligands, showing that the binding pocket is highly preserved throughout the series. The residues involved in polar contacts are shown as thick sticks: glutamic acid 273 is involved in classical H-bonding and methionine 304 is concurrently involved in NH···O^{NO2} hydrogen bonding and $^{Met\text{-}304}O\cdots N^{NO2}$ π-hole bonding with an average distance of 2.874 Å (of four ligands), which is well within the sum of the van der Waals radii of N+O (1.55 + 1.52 = 3.07 Å); **C)** The four individual structures and their ligands and π-hole bonding distances; **D)** LigPlus plot of ligand d1a-601 in its binding pocket in 2w7x. Polar contacts are shown as green striped lines with bonding distance. The polar residues are shown as balls and sticks and the apolar residues as red eye-lashes; **E)** Schematic drawing of the chemical structures of the ligands bearing a nitro group (2w7x, 2yir, 2ycq, 2xk9) and those not bearing a nitro group (2ycs, 2ycr, 2ycf). **F)** The molecular electrostatic potential map of d1a-601 (atomic coordinates extracted from the PDB) with the nitro group hydrogen bonded to a water molecule to mimic the ligand being bound by the protein. The colour code spans from +125 (blue) to –125 kJ/mol (red) in eight bands. All distances are in Angstroms (Å). See also Figure 4 and Figure 5 (middle) and the main text.

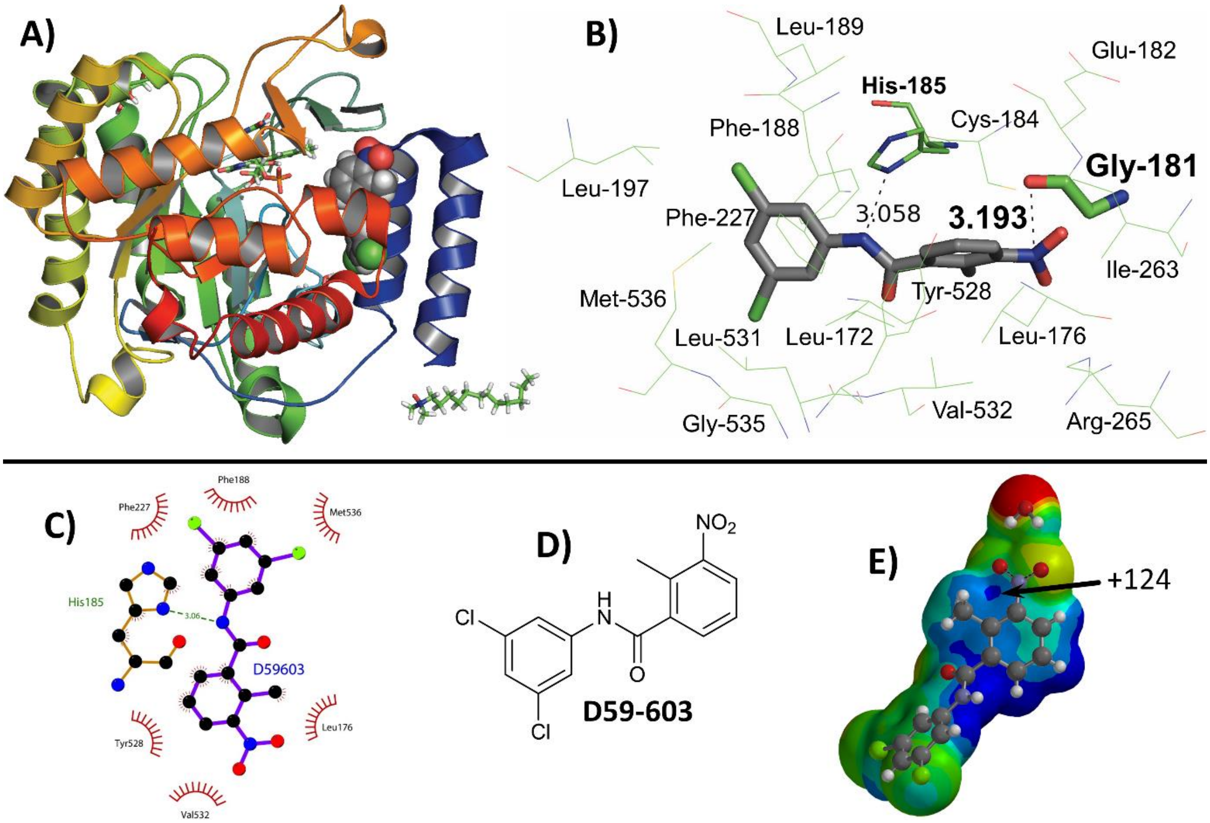

Figure S19. Illustrations of possible relevance of Ar-NO_2 π-hole interactions involving protein structure 5del.[19] **A)** Cartoon representation of the entire protein structure with molecular structures of the ligands (ligand d59-603 in grey space filling mode); **B)** Capped stick representation of the binding pocket of ligand d59-603 (grey carbon skeleton, 4 Å residues around the ligand are displayed). The residues involved in polar contacts are shown as thicker sticks: glycine-181 (thickest sticks) is in close proximity of the nitro group with a $^{Gly\text{-}181}O\cdots N^{NO2}$ distance of 3.193 Å; **C)** LigPlus plot of ligand d59-603 in its binding pocket in 5del. Polar contact is shown as green striped line with bonding distance. The polar residue is shown as balls and sticks and the apolar residues as red eye-lashes; **D)** Schematic drawing of the chemical structure of ligand d59-603; **E)** The molecular electrostatic potential map of d59-603 (atomic coordinates extracted from the PDB) with the nitro group hydrogen bonded to a water molecule to mimic the ligand being bound by the protein. The colour code spans from +150 (blue) to –150 kJ/mol (red) in eight bands. All distances are in Angstroms (Å).

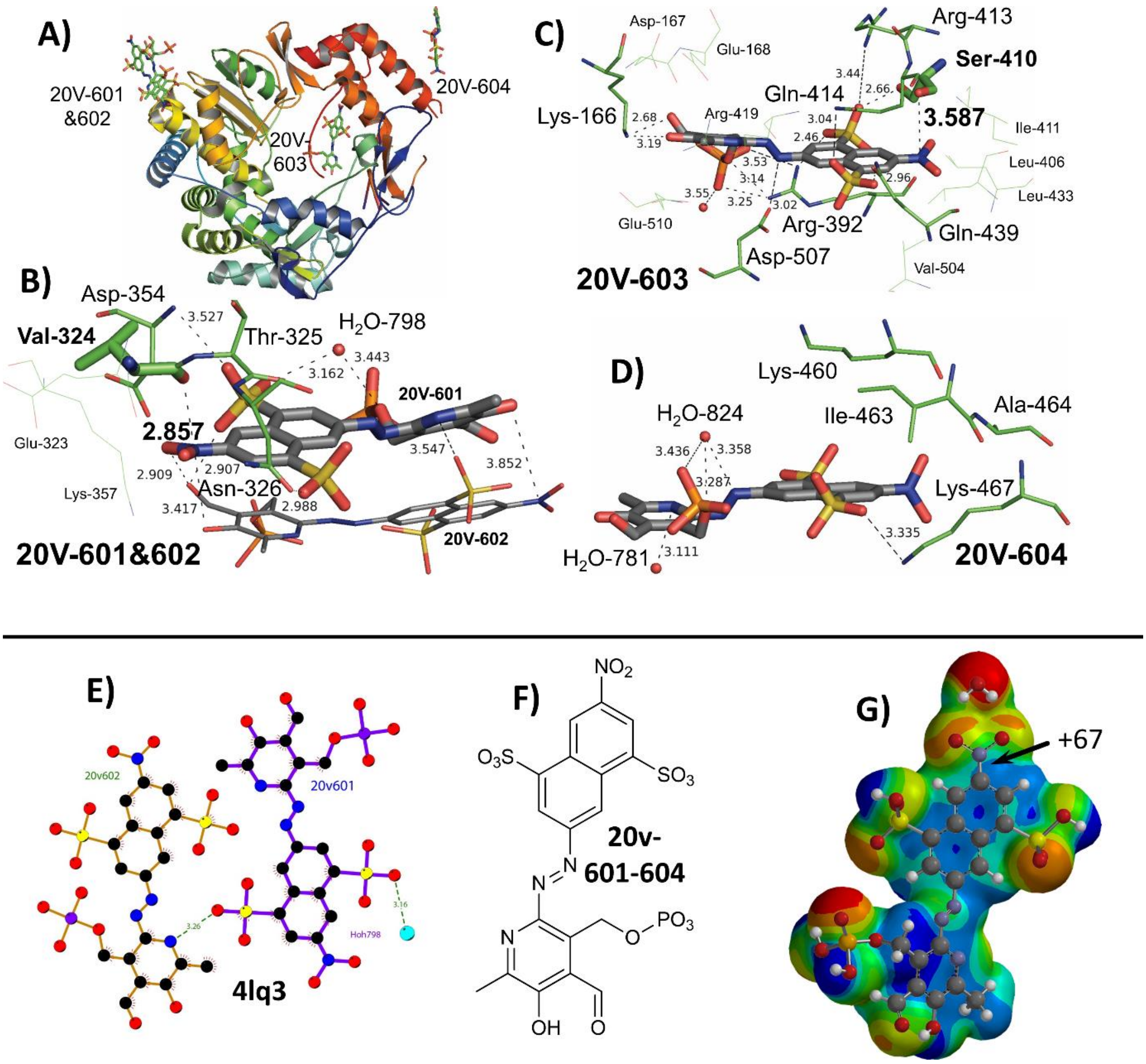

Figure S20. Illustrations of possible Ar-NO_2 π-hole interaction in structure 4lq3.[70] **A)** Cartoon representation of the entire protein structure indicating the ligations of the (isostructural) ligands; **B-D)** Capped stick representation of the ligands 20V-601/602 (B), 20V-603 (C) and 20V-604 (D) in their respective binding pockets where the polar contacts have been marked with dashed lines and distance information (ligand carbon skeleton depicted in thick grey sticks). In the case of 20V-601 there appears to be a π-hole contact with $^{\text{Val-324}}\text{O}\cdots\text{N}^{\text{NO2}}$ distance of 2.857 Å, which is 0.213 Å shorter than the sum of the van der Waals radii of N+O (1.55 + 1.52 = 3.07 Å). This interaction represents *the only* (polar) contact of the ligand with the protein. The ligand is also involved in π-π stacking interactions with ligand 20V-602. Ligand 20V-603 (C) is held in plave by several polar contacts and a relatively close $^{\text{Ser-410}}\text{O}\cdots\text{N}^{\text{NO2}}$ distance of 3.587 Å. Ligand 20V-604 (D) is located on the protein's periphery and apparently lacks π-hole type interactions with the nitro moiety; **E)** LigPlus plot of ligand 20V-601 in its apparent binding pocket, which –according to the LigPlot standards– involves only ligand 20V-602 and a water molecule. Polar contacts are shown as green striped lines with bonding distance; **F)** Schematic drawing of the chemical structures of ligands 20V-601-604 (the protonation states are unknown); **G)** The molecular electrostatic potential map of 20V-601 (atomic coordinates extracted from the PDB) with the nitro group hydrogen bonded to a water molecule to mimic the ligand being bound by the protein. The colour code spans from +150 (blue) to –150 kJ/mol (red) in eight bands. All distances are in Angstroms (Å).

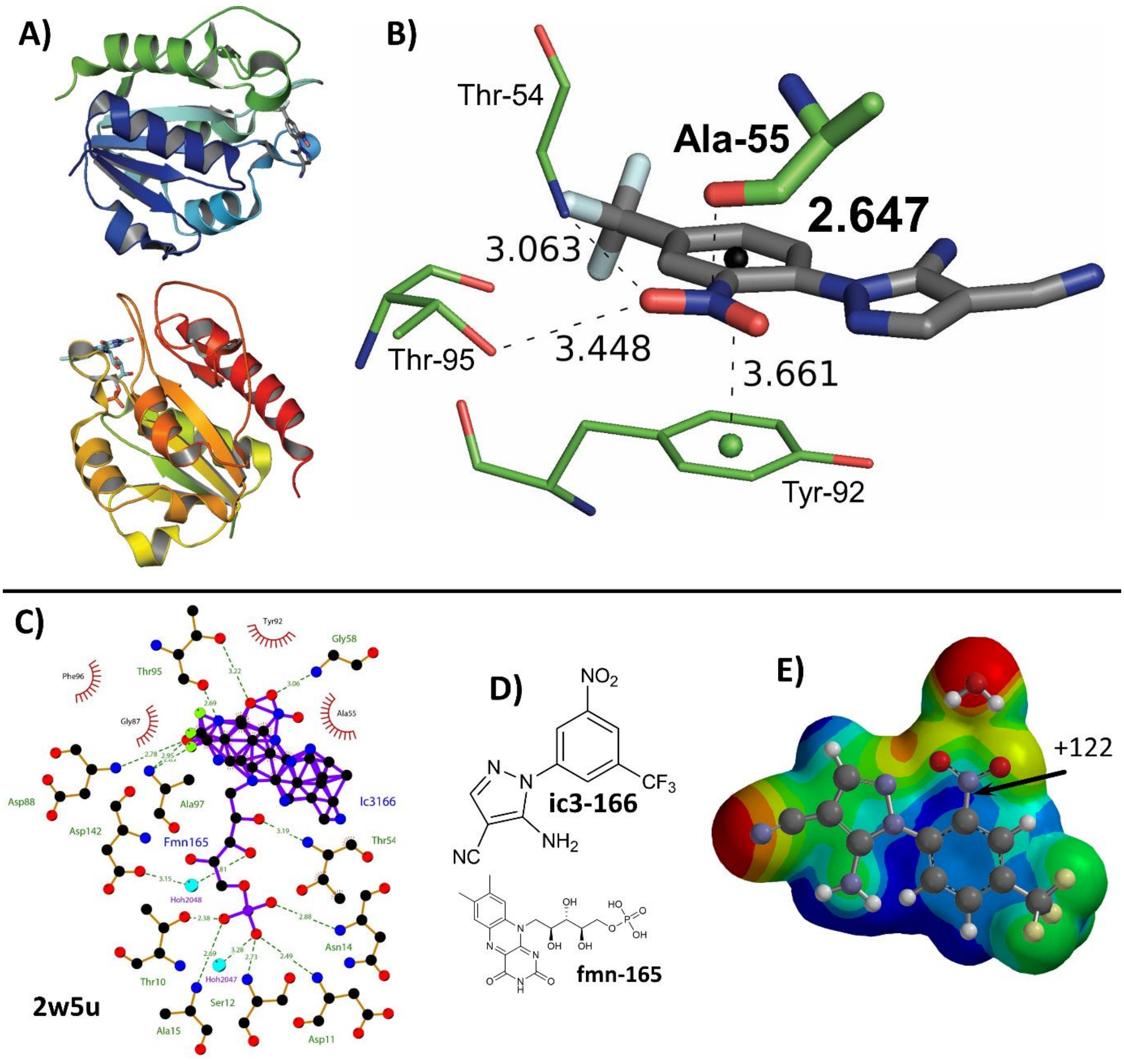

Figure S21. Illustrations of possible Ar-NO_2 π-hole interaction in structure 2w5u.[37] **A)** Cartoon representation of the protein structure; **B)** Capped stick representation of the ligand ic3-166 (in grey, see also D) surrounded by the residues in the binding pocket that surround the ligands nitro group (in green). In particular the nitro moiety is sandwiched in between alanine 55 and tyrosine 92, both of which (especially Ala-55) might very well be interacting with the ligands π-hole. Indeed, the $^{Ala\text{-}55}O{\cdots}N^{NO2}$ distance of 2.647 Å is 0.423 Å shorter than the sum of the van der Waals radii of N+O (1.55 + 1.52 = 3.07 Å); **C)** LigPlus plot of ligand ics-166 (overlapping with ligand fmn-165) in its binding pocket in 2w5u. Polar contacts are shown as green striped lines with bonding distance. The polar residues are shown as balls and sticks and the apolar residues as red eye-lashes; **D)** Schematic drawing of the chemical structures of the ligands ic3-166 and fmn-15; **E)** The molecular electrostatic potential map of ic3-166 (atomic coordinates extracted from the PDB) with the nitro group hydrogen bonded to a water molecule to mimic the ligand being bound by the protein. The colour code spans from +150 (blue) to –150 kJ/mol (red) in eight bands. All distances are in Angstroms (Å).

3.5. Example of likely functional relevance of π-hole interaction with C–S.

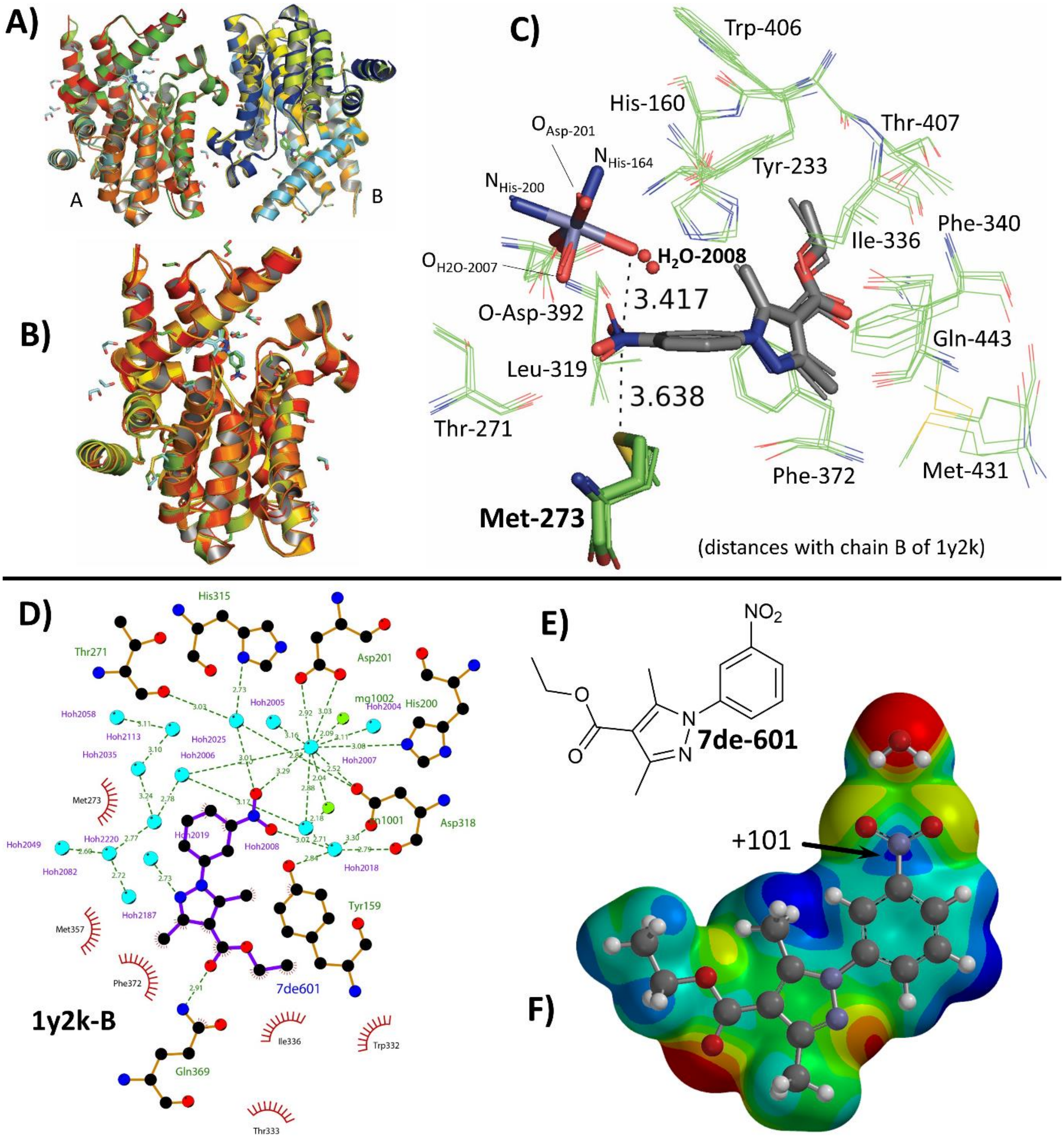

Figure S22. Illustrations of possible Ar-NO_2 π-hole interactions as found within protein structures 1y2k and 1y2j.[73] **A)** Cartoon representation of the aligned protein structfures; **B)** Cartoon representation of all chains A and B aligned; **C)** Stick representation of aligned Ar-NO_2 bearing ligands with selected residues at ≤ 4 Å from the ligand, illustrating that the binding pocket is highly preserved and that the nitrate fragment is sandwiched in between two electron rich atoms; the O-atom of Mg-bound H_2O-2008 and the S-atoms of Met-273. The distances are those found within chain B of 1y2k; **D)** LigPlus plot of ligand 7de-601 in its binding pocket within chain B of 1y2k. Polar contacts are shown as green striped lines with bonding distance. The polar residues are shown as balls and sticks and the apolar residues as red eye-lashes; **E)** Schematic drawing of ligand 7de-601's chemical structure; **F)** The molecular electrostatic potential map of 7de-601 (atomic coordinates extracted from the PDB) with the nitro group hydrogen bonded to a water molecule to mimic the ligand being bound by the protein. The colour code spans from +125 (blue) to –125 kJ/mol (red) in eight bands. All distances are in Angstroms (Å). See also Figure 4 and Figure 5 (right) and the main text.

3.6. π-hole interactions that seem at least structurally relevant with H_2O.

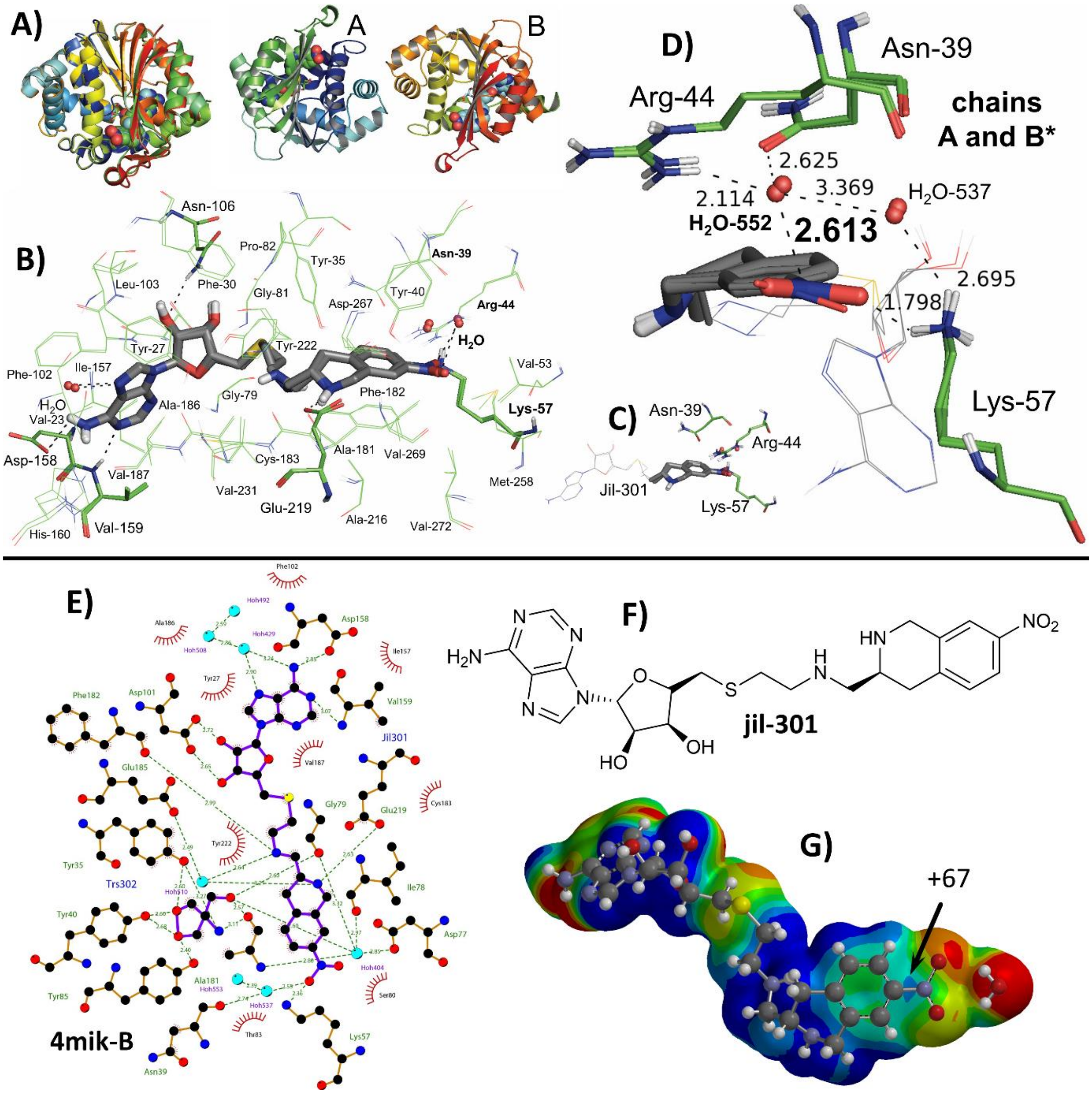

Figure S23. Illustrations of possible Ar-NO_2 π-hole interactions involving protein structure 4mik.[57] **A)** Cartoon representations of protein structure 4mik with chains A and B aligned (left) and separate (right) and the ligands in space filling mode; **B)** Capped stick representation of the aligned binding pockets (4 Å residues around the ligand) of ligand jil-301 (shown as thickest sticks with a grey C-backbone, see also F) as found in chains A and B. The residues involved in polar contacts are shown as thinker sticks; **C)** Selected part from the binding pocket of jil-301 highlighting the nitro aromatic moiety (thicker grey sticks) and the residues surrounding it. This view has the same perspective as in B and the same residues as in D; **D)** Partial aligned binding pockets of jil-301 (same residues as in C but from a different perspective) as found in chains A and B of 4mik. The distances shown are taken from chain B (hence the asterisk in the figure). Water molecule H_2O-552 has polar contacts with residues Arganine-44 and Asparganine-39, and water molecule H_2O-537. Concurrently, the H_2O-552 O-atom is in close proximity to the π-hole of ligand jil-301 (grey) with a $^{water\text{-}552}O{\cdots}N^{NO2}$ distance of 2.613 Å, which is 0.457 Å within the sum of the van der Waals radii of N+O (1.55 + 1.52 = 3.07 Å) and thus indicative of a π-hole interaction; **E)** LigPlus plot of ligand jil-301 in chain B of 4mik. Polar contacts are shown as green striped lines with bonding distance. The polar residues are shown as balls and sticks and the apolar residues as red eye-lashes; **F)** Schematic drawing of the chemical structures of ligand jil-301; **G)** The molecular electrostatic potential map of jil-301 (atomic coordinates extracted from chain B of 4mik) with the nitro group hydrogen bonded to a water molecule to mimic the ligand being bound by the protein. The colour code spans from +125 (blue) to –125 kJ/mol (red) in eight bands. All distances are in Angstroms (Å).

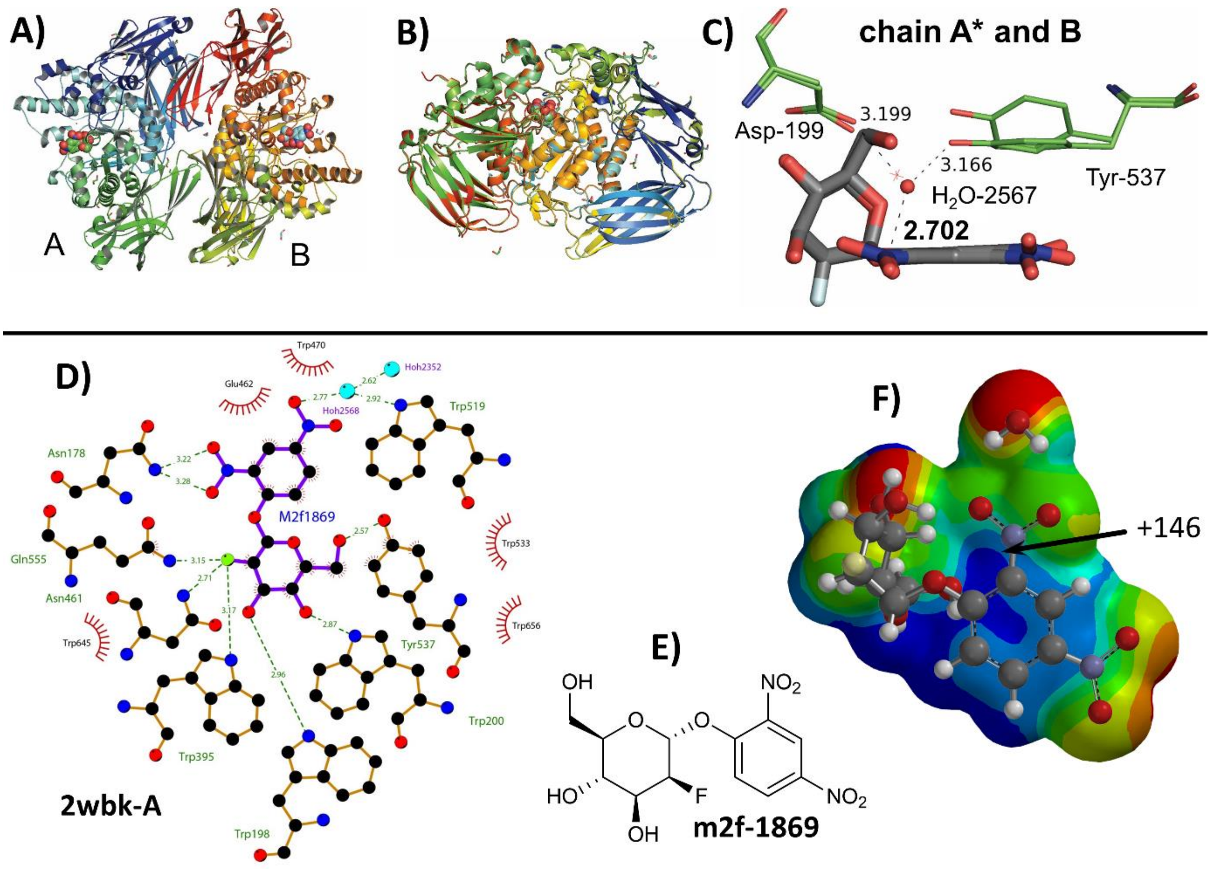

Figure S24. Illustrations of possible Ar-NO_2 π-hole interactions involving protein structure 2wbk.[17] **A)** Cartoon representation of chains A and B in protein 2wbk with ligands m2f-1869 (see also E) in space-filling mode; **B)** Cartoon representation of aligned chains A and B of protein 2wbk; **C)** Capped stick representation of the binding pockets of water molecule H_2O-2567, revealing polar contacts with residues Asparagine-199 and Tyrosine-537 (green). The water O-atom is also close proximity to the π-hole of ligand m2f-1869 (grey) with a $^{water\text{-}2567}O\cdots N^{NO2}$ distance of 2.702 Å (in chain A, hence the asterisk in the figure), which is 0.368 Å within the sum of the van der Waals radii of N+O (1.55 + 1.52 = 3.07 Å) and thus indicative of a π-hole interaction; **D)** LigPlus plot of ligand m2f-1869 in chain A of 2wbk. Note that the nitrate moiety possibly involved in the π-hole interaction (*ortho* relative to the carbohydrate part) is H-bonded to Asn-178, but –according to current conventional criteria– not bound to H_2O-2567. Polar contacts are shown as green striped lines with bonding distance. The polar residues are shown as balls and sticks and the apolar residues as red eye-lashes; **E)** Schematic drawing of the chemical structure of ligand m2f-1869; **F)** The molecular electrostatic potential map of m2f-1869 (atomic coordinates extracted from chain A of 2wbk) with the nitro group hydrogen bonded to a water molecule to mimic the ligand being bound by the protein. The colour code spans from +175 (blue) to –175 kJ/mol (red) in eight bands. All distances are in Angstroms (Å).

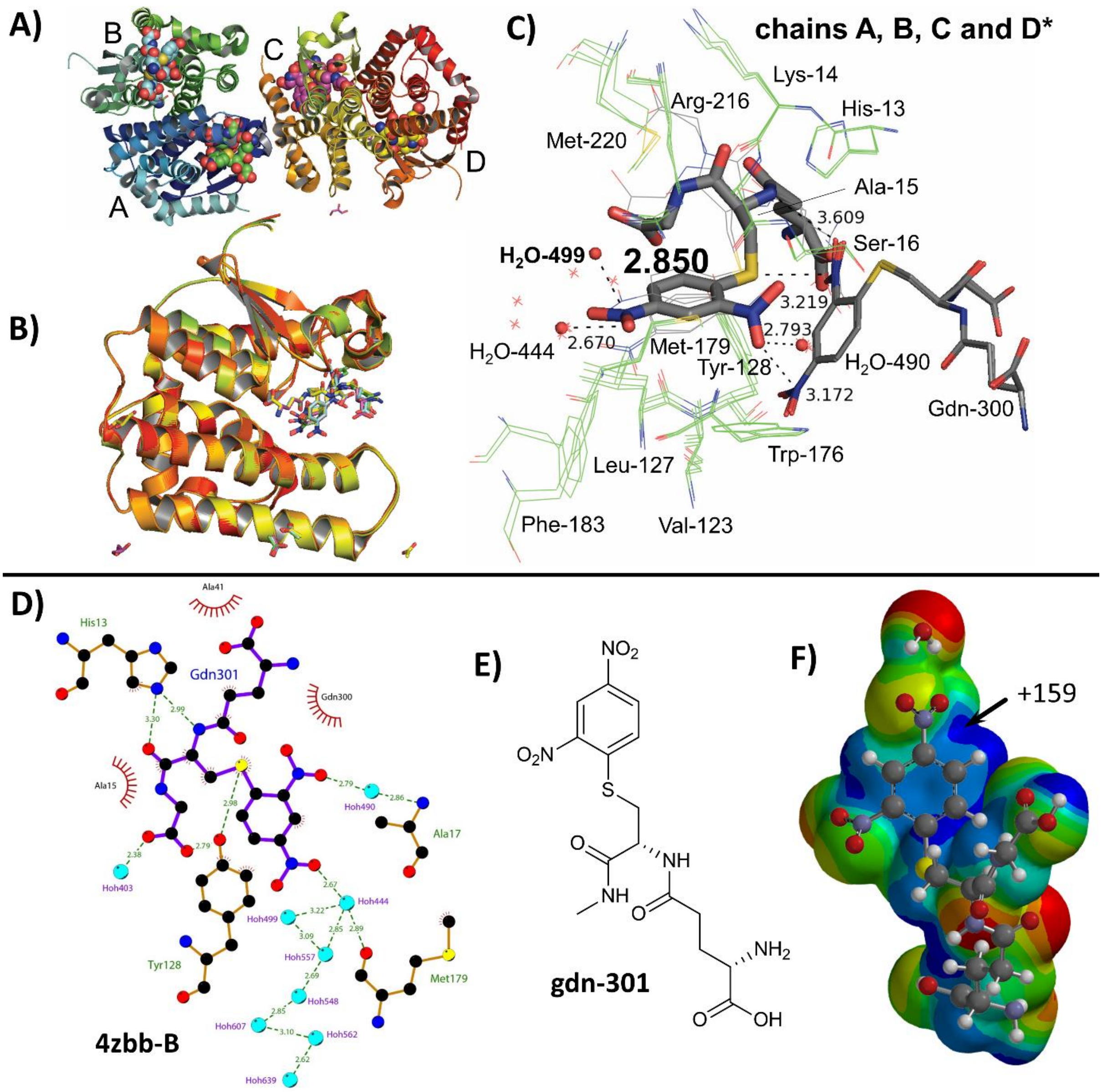

Figure S25. Illustrations of possible Ar-NO_2 π-hole interactions involving protein structure 4zbb.[18] **A)** Cartoon representation of protein structures 4zbb with the ligands in space filling mode; **B)** Cartoon representation of aligned chains A-D from protein structure 4zbb with the ligands in capped sticks mode; **C)** Capped stick representation of the binding pocket (4 Å residues around the ligand) of ligand gdn-301 (shown as thickest sticks with a grey C-backbone, see also E), revealing that the binding mode is well-preserved throughout chains A-D. The distances shown are taken from chain D (hence the asterisk in the figure). Water molecule 499 is located very near the π-hole interaction region of one of the ligand's nitro moieties with a $^{water\text{-}499}O\cdots N^{NO2}$ distance of 2.850 Å, which is 0.220 Å within the sum of the van der Waals radii of N+O (1.55 + 1.52 = 3.07 Å). Ligand gdn-301 is also interacting with ligand gdn-300 (medium thickness, grey C-backbone), possibly by two π-hole interactions involving both nitro moieties of gdn-300 as electron acceptors. The electron donors are then the S atom in gdn-301 and the nitro-group ortho to that S-atom. These distances are, respectively: $^{gdn\text{-}301}S\cdots N^{NO2@gdn\text{-}300}$ = 3.219 Å (0.131 Å shorter than the v/d Waals radii of S and N; 1.80 + 1.55 = 3.35 Å) and $^{gdn\text{-}301}O\cdots N^{NO2@gdn\text{-}300}$ = 3.172 Å (0.01 Å longer than the v/d Waals radii of O and N; 1.52 + 1.55 = 3.07 Å) and thus indicative of a π-hole interaction; **D)** LigPlus plot of ligand gdn-301 in chain B of 4zbb. Polar contacts are shown as green striped lines with bonding distance. The polar residues are shown as balls and sticks and the apolar residues as red eye-lashes; **E)** Schematic drawing of the chemical structures of ligand gdn-301; **F)** The molecular electrostatic potential maps of gdn-301 (atomic coordinates extracted from chain B of 4zbb) with the nitro group hydrogen bonded to a water molecule to mimic the ligand being bound by the protein. The colour code spans from +175 (blue) to –175 kJ/mol (red) in eight bands. All distances are in Angstroms (Å).

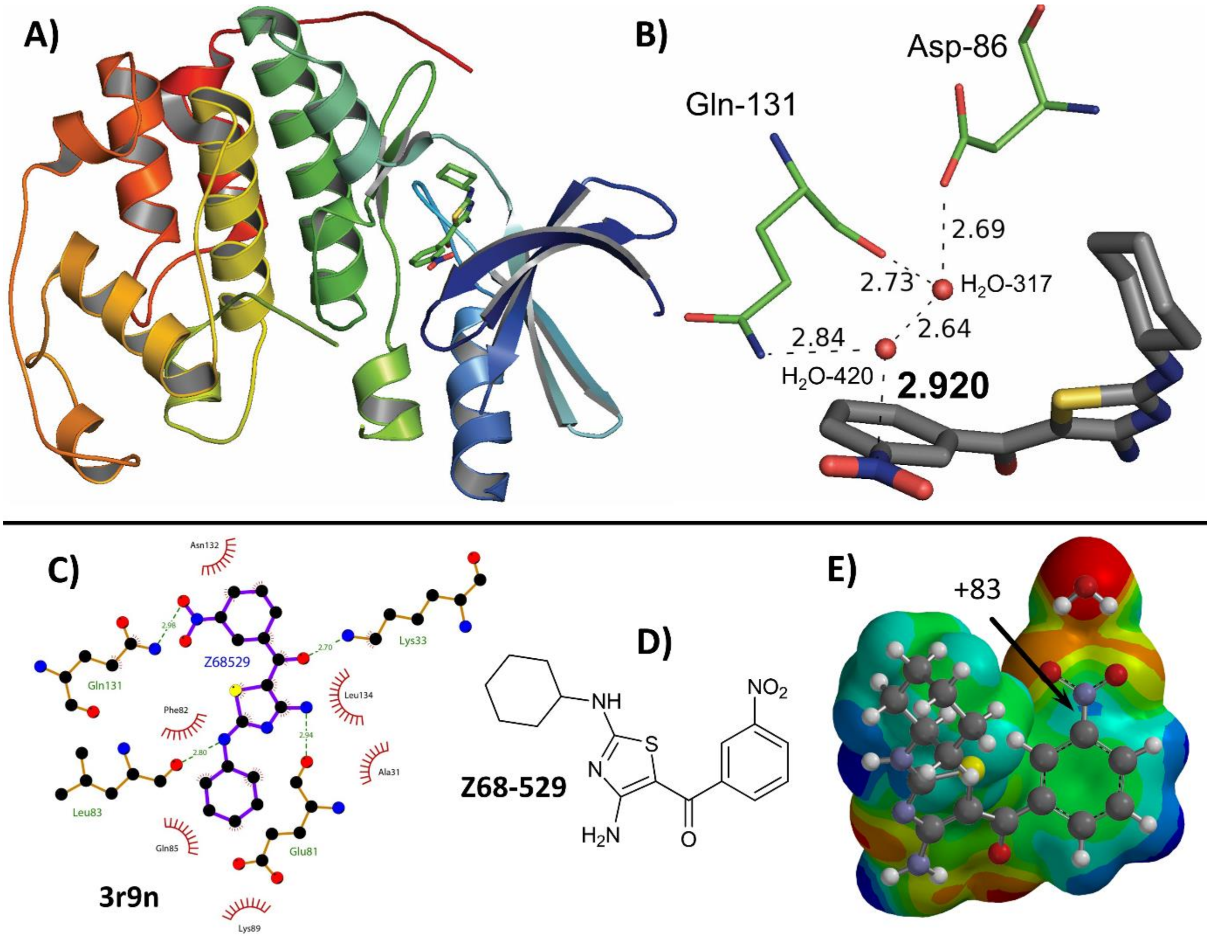

Figure S26. Illustrations of possible Ar-NO_2 π-hole interactions involving protein structure 3r9n.[40] **A)** Cartoon representation of protein 3r9n with ligand z68-529 (see also D) in capped stick mode; **B)** Capped stick representation of the binding pocket of water molecule H_2O-420, revealing polar contacts with residue Glutamine-131 and water-317 (which is also bound to Glutamine-131 and Asparganine-68). The water O-atom is also in close proximity to the π-hole of ligand z68-529 (grey) with a $^{water\text{-}420}O\cdots N^{NO2}$ distance of 2.920 Å, which is 0.150 Å within the sum of the van der Waals radii of N+O (1.55 + 1.52 = 3.07 Å) and thus indicative of a π-hole interaction; **C)** LigPlus plot of ligand z68-529 in 3r9n. Note that the nitrate moiety possibly involved in the π-hole interaction is H-bonded to Gln-131, but –according to current conventional criteria– not bound to H_2O-420. Polar contacts are shown as green striped lines with bonding distance. The polar residues are shown as balls and sticks and the apolar residues as red eye-lashes; **E)** Schematic drawing of the chemical structure of ligand z68-529; **I)** The molecular electrostatic potential map of z68-529 (atomic coordinates extracted from 3r9n) with the nitro group hydrogen bonded to a water molecule to mimic the ligand being bound by the protein. The colour code spans from +150 (blue) to –150 kJ/mol (red) in eight bands. All distances are in Angstroms (Å).

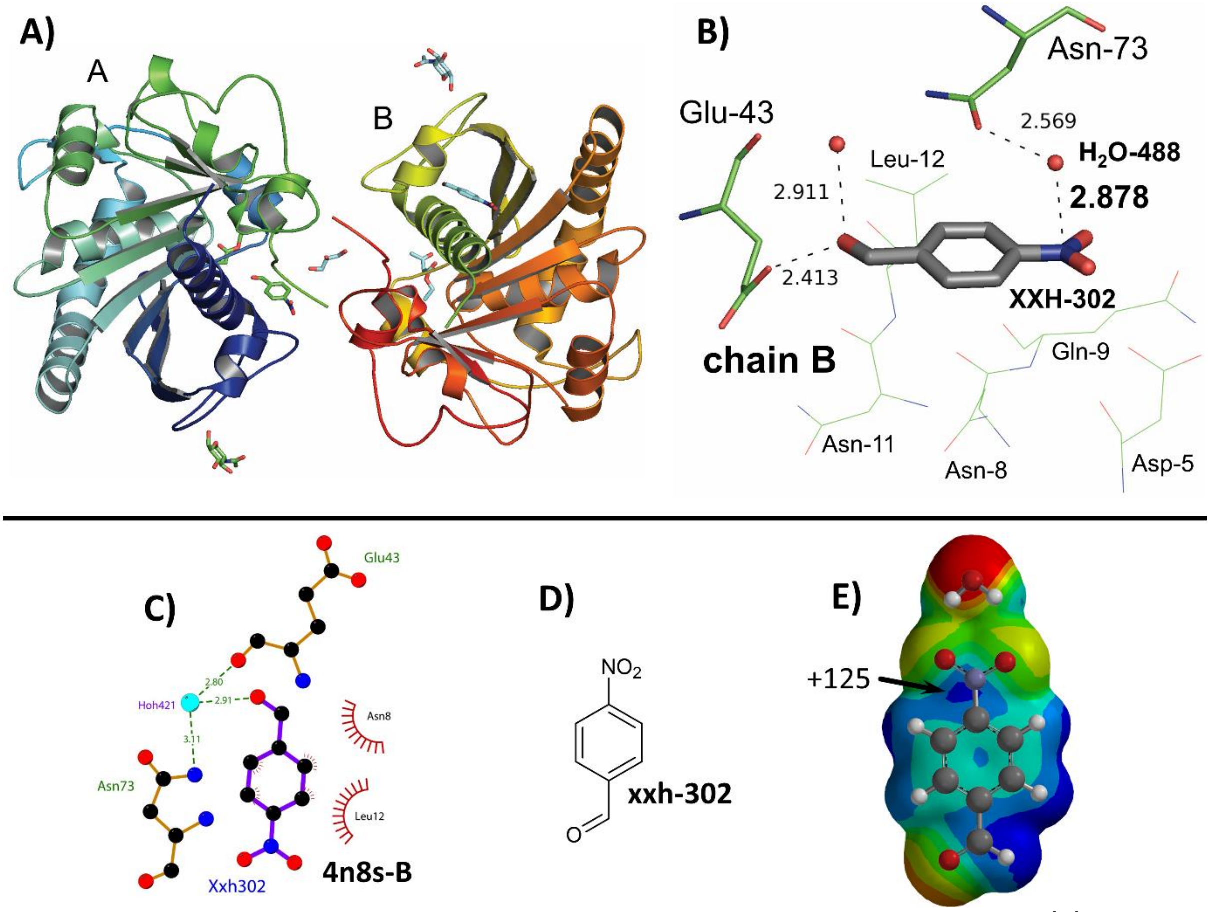

Figure S27. Illustrations of possible Ar-NO_2 π-hole interactions involving protein structure 4n8s.[59] **A)** Cartoon representation of protein structure 1zwp with the ligands in capped-sticks mode and the two chains (A and B) labelled; **B)** Capped stick representation of the binding pocket (4 Å residues around the ligand) of ligand xxh-302 in chain B (shown as thickest sticks with a grey C-backbone, see also D). The residues involved in polar contacts are shown as thinker sticks. Water molecule H_2O-488 has a polar contact with residue Asparagine-73 and its O-atom is in close proximity to the π-hole of ligand xxh-302 (grey) with a $^{water\text{-}466}O{\cdots}N^{NO2}$ distance of 2.878 Å, which is 0.192 Å within the sum of the van der Waals radii of N+O (1.55 + 1.52 = 3.07 Å) and thus indicative of a π-hole interaction; **C)** LigPlus plot of ligand xxh-302 in chain B of 4n8s. Polar contacts are shown as green striped lines with bonding distance. The polar residues are shown as balls and sticks and the apolar residues as red eye-lashes; **D)** Schematic drawing of the chemical structures of ligand xxh-302; **E)** The molecular electrostatic potential map of xxh-302 (atomic coordinates extracted from chain B in 4n8s) with the nitro group hydrogen bonded to a water molecule to mimic the ligand being bound by the protein. The colour code spans from +150 (blue) to –150 kJ/mol (red) in eight bands. All distances are in Angstroms (Å).

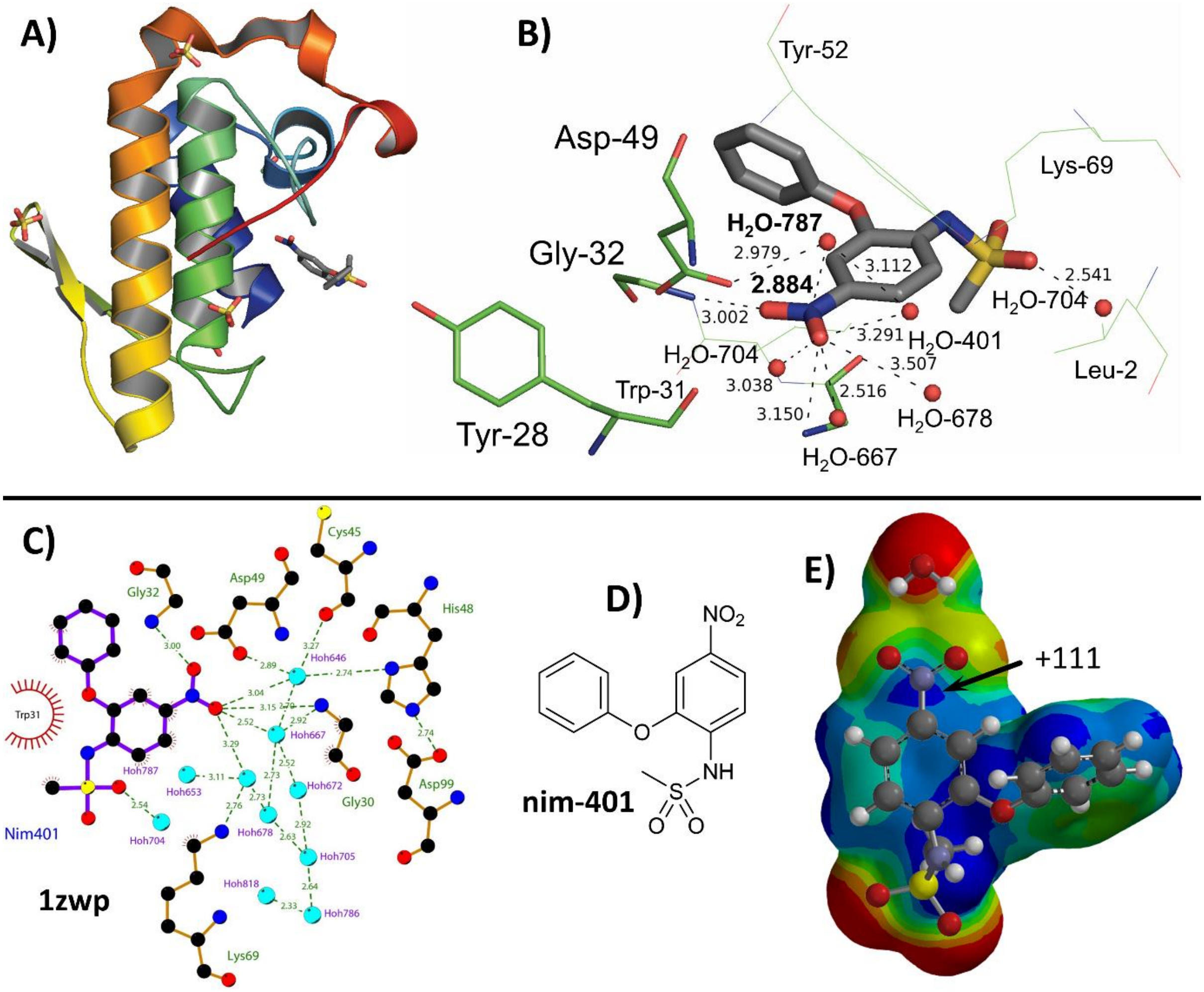

Figure S28. Illustrations of possible Ar-NO_2 π-hole interactions involving protein structure 1zwp.[83] **A)** Cartoon representation of protein structure 1zwp with the ligands in capped-sticks mode; **B)** Capped stick representation of the binding pocket (4 Å residues around the ligand) of ligand nim-401 (shown as thickest sticks with a grey C-backbone, see also D). The residues involved in polar contacts are shown as thinker sticks. Water molecule H_2O-787 has polar contacts with residue Asparagine-49 and water molecule H_2O-401. Concurrently, the H_2O-787 O-atom is in close proximity to the π-hole of ligand nim-401 (grey) with a $^{water\text{-}787}O\cdots N^{NO2}$ distance of 2.884 Å, which is 0.186 Å within the sum of the van der Waals radii of N+O (1.55 + 1.52 = 3.07 Å) and thus indicative of a π-hole interaction; **C)** LigPlus plot of ligand nim-401 in 1zwp. Polar contacts are shown as green striped lines with bonding distance. The polar residues are shown as balls and sticks and the apolar residues as red eye-lashes; **D)** Schematic drawing of the chemical structures of ligand nim-401; **E)** The molecular electrostatic potential map of nim-401 (atomic coordinates extracted from 1zwp) with the nitro group hydrogen bonded to a water molecule to mimic the ligand being bound by the protein. The colour code spans from +125 (blue) to –125 kJ/mol (red) in eight bands. All distances are in Angstroms (Å).

3.7. π-hole interactions that seem at least structurally relevant with C=O.

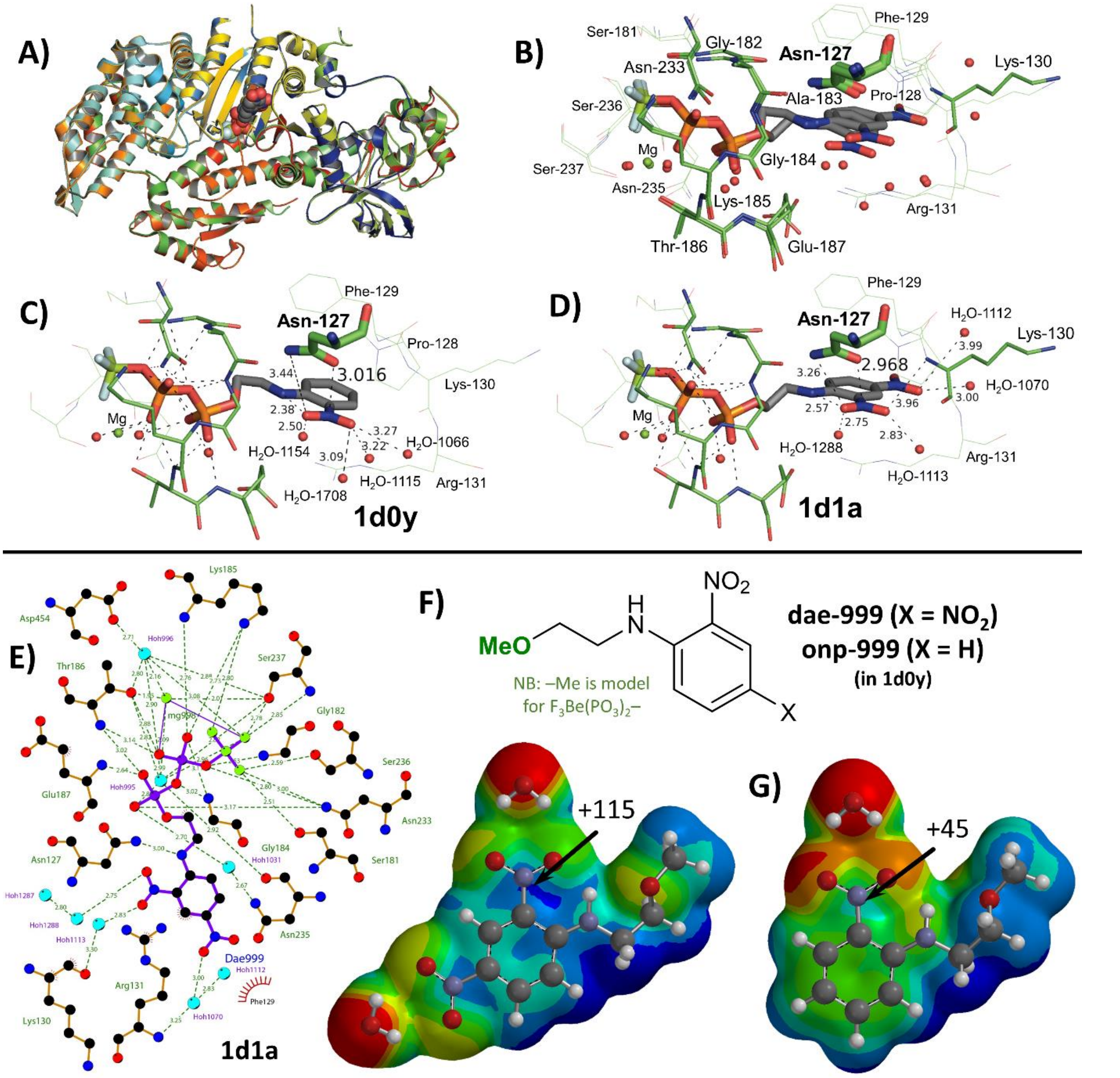

Figure S29. Illustrations of possible Ar-NO₂ π-hole interactions involving protein structures 1d0y and 1d1a.[21] **A)** Cartoon representation of the aligned protein structures; **B)** Capped stick representation of the aligned ligands (grey C-backbone) in their binding pockets (4 Å residues around the ligands). The residues involved in polar interactions are shown as thicker sticks and asparagine-127, which is near the ligands π-hole region, is shown as the thickest sticks. **C)** and **D)** show the individual binding pockets (4 Å residues around the ligands) of 1d0y and 1d1a respectively. The $^{Asn\text{-}127}O{\cdots}N^{NO2}$ distances of 3.016 Å (in 1doy) and 2.968 Å (in 1d1a) are below the van der Waals radii of N+O (1.55 + 1.52 = 3.07 Å) and thus indicative of a π-hole interaction; **E)** LigPlus plot of ligand dae999 in 1d1a. Polar contacts are shown as green striped lines with bonding distance. The polar residues are shown as balls and sticks and the apolar residues as red eye-lashes; **F)** Schematic drawing of the chemical structures of ligands dae-999 (found in 1d1a) and onp-999 (found in 1d0y); **G)** The molecular electrostatic potential maps of dae-999 (left, extracted from 1d1a) and onp-999 (extracted from 1d0y) with the nitro group hydrogen bonded to a water molecule to mimic the ligand being bound by the protein. The colour code spans from +150 (blue) to –150 kJ/mol (red) in eight bands for dae-999 (left, 1d1a) and idem but from +125 to –125 for onp-999 (right, 1d0y). The F_3Be-terminating phosphate chain has been simplified to circumvent the issue of charge of the phosphate groups (which is unknown). All distances are in Angstroms (Å). **NB:** Out of six ATP-analogues examined, the two highlighted above (named oNPhAE (onp-999) and op-NPhAE (dae-999) in the paper) are the only two that have a similar active section and shortening velocity as ATP (see Table V in the article). The authors attribute this to hydrogen bonding patterns, but the interaction with Asn-127 might contribute to this as well. Indeed, the authors noted that: 'there is a positive correlation between the distance of the bridging nitrogen from Asn127 and the ability to sustain tension and generate movement.'.[21]

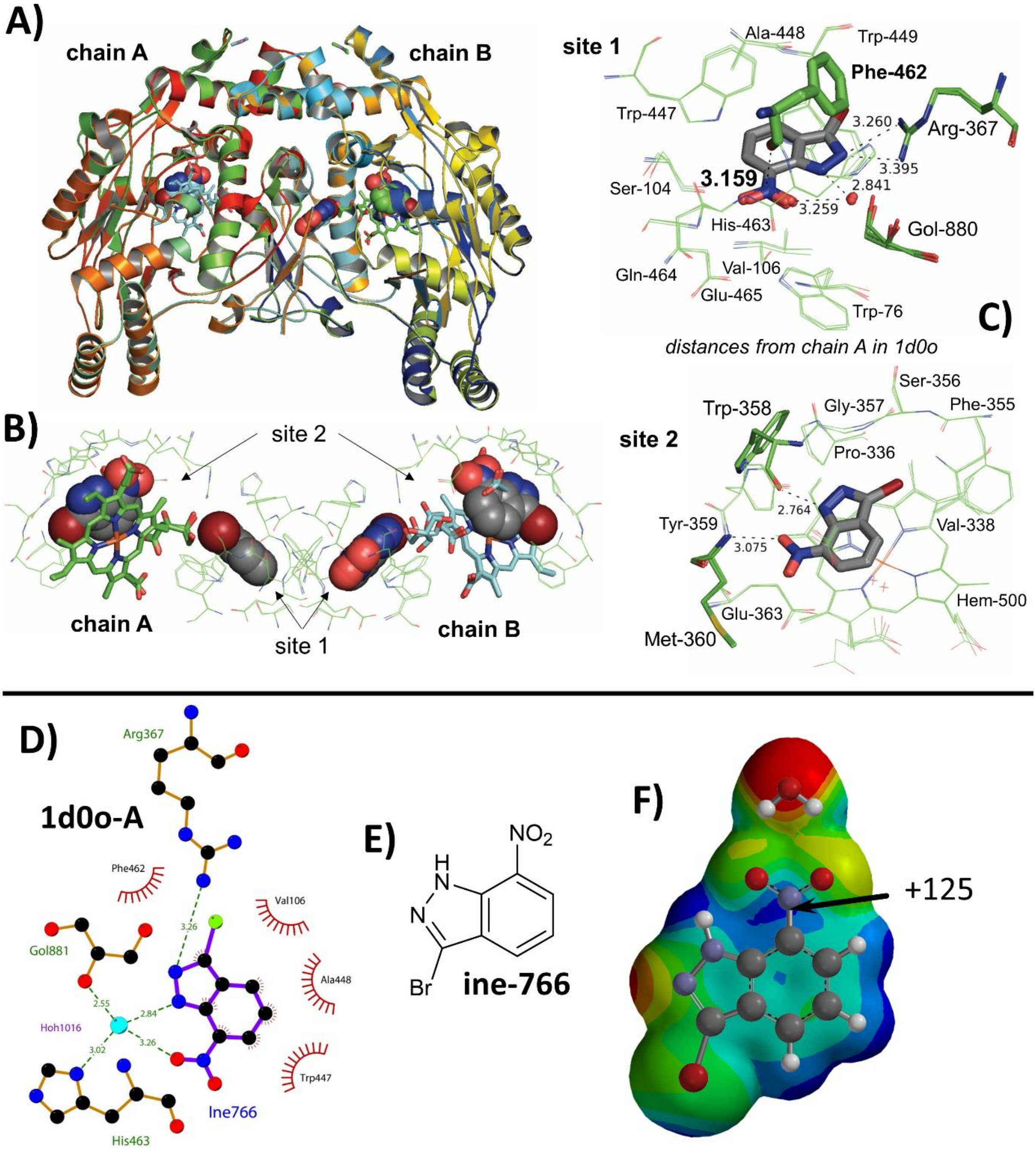

Figure S30. Illustrations of possible Ar-NO_2 π-hole interactions involving protein structures 1d0c and 1d0o.[15] **A)** Cartoon representation of the aligned protein structures with the ligands in space filling mode; **B)** Capped stick representation of the aligned binding pockets (4 Å residues around the ligands) wherein the heme moieties are shown as thicker sticks and the two different binding sites are indicated; **C)** close-up of the two binding sites (4 Å residues around the ligands) wherein the polar contacts are shown as thicker sticks and the ligands C-backbone is in grey. Within sites 1 there is a short $^{Phe\text{-}462}O\cdots N^{NO2}$ distance (3.159 Å in 1d0o), while no such interactions are present in sites 2; **D)** LigPlus plot of ligand ine-766 in chain A of 1d0o (site 1). Polar contacts are shown as green striped lines with bonding distance. The polar residues are shown as balls and sticks and the apolar residues as red eye-lashes; **E)** Schematic drawing of the chemical structures of ligand ine-766; **F)** The molecular electrostatic potential maps of ine-766 (atomic coordinates extracted from 1d0o) with the nitro group hydrogen bonded to a water molecule to mimic the ligand being bound by the protein. The colour code spans from +150 (blue) to –150 kJ/mol (red) in eight bands. All distances are in Angstroms (Å).

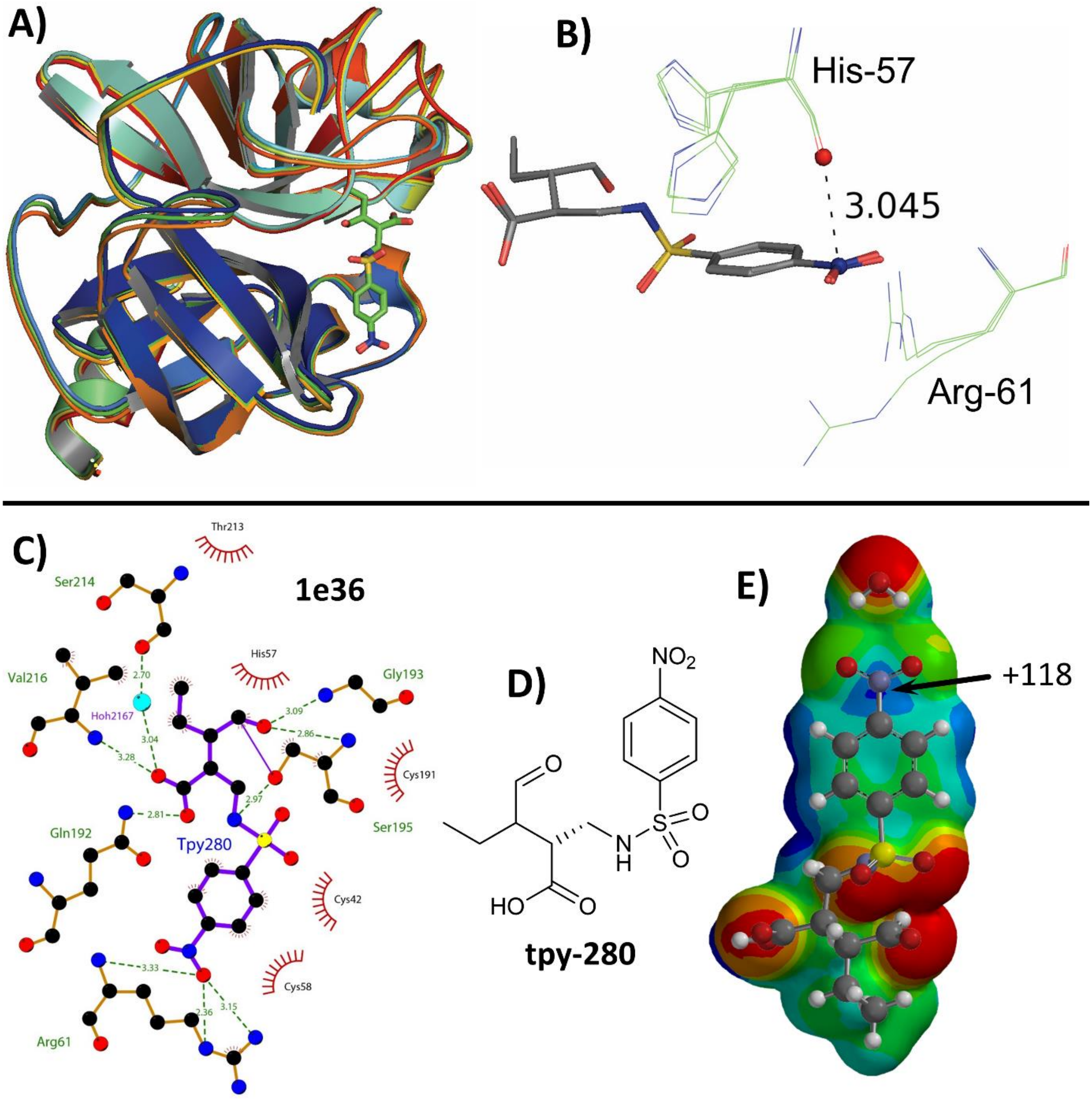

Figure S31. Illustrations of possible Ar-NO_2 π-hole interactions as found within protein structures 1e36, 1e37 and 1e38.[23] **A)** Cartoon representation of aligned structures; **B)** Capped sticks representation of the three isostructural ligands aligned with the two residues closest to the nitro group, showing that the binding mode of the -NO_2 group is highly preserved throughout these three structures. It seems that the –NO_2 is π-hole bonded to a carbonyl O-atom of histidine 57 with an average $^{His\text{-}57}O\cdots N^{NO2}$ distance of 3.045 Å, which is within the sum of the van der Waals radii of N+O (1.55 + 1.52 = 3.07 Å). The –NO_2 moiety is also H-bonded to arginine 61 (see also C). **C)** LigPlus plot of ligand tpy-280 in its binding pocket in 1e36. Polar contacts are shown as green striped lines with bonding distance. The polar residues are shown as balls and sticks and the apolar residues as red eye-lashes; **D)** Schematic drawing of the chemical structures of ligand tpy-280. **E)** The molecular electrostatic potential map of tpy-280 (atomic coordinates extracted from the PDB) with the nitro group hydrogen bonded to a water molecule to mimic the ligand being bound by the protein. The colour code spans from +150 (blue) to –150 kJ/mol (red) in eight bands. All distances are in Angstroms (Å). **NB:** It is noteworthy that these –essentially isostructural– complexes were obtained from very different conditions; 1e36 was obtained from a solution at pH 5 and 1e37 (1 minute soaking) / 1e38 (2 minutes soaking) from a solution at pH 9 (see Table 1 in the paper).[23]

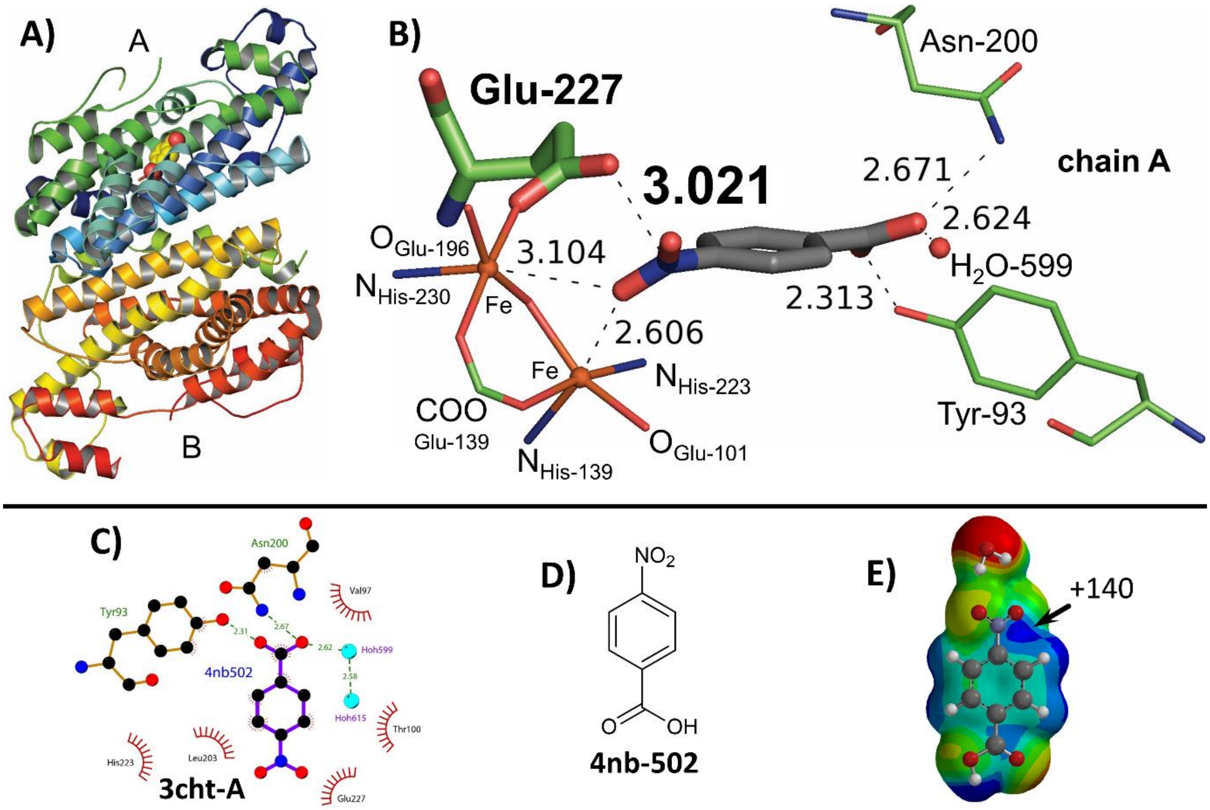

Figure S32. Illustrations of possible relevance of Ar-NO_2 π-hole interaction in structure 3cht.[46] **A)** Cartoon representation of the entire protein structure; **B)** Capped stick representation of the ligand 4nb-502 (in grey, see also D) surrounded by the residues in the binding pocket that seem to be involved in polar contacts with an apparent π-hole contact with $^{Glu\text{-}227}O\cdots N^{NO2}$ of 3.021 Å, somewhat shorter than the sum of the van der Waals radii of N+O (1.55 + 1.52 = 3.07 Å); **C)** LigPlus plot of ligand 4nb-502 in its binding pocket in 3cht (chain A). Polar contacts are shown as green striped lines with bonding distance. The polar residues are shown as balls and sticks and the apolar residues as red eye-lashes; **D)** Schematic drawing of the chemical structures of ligand 4nb-502; **E)** The molecular electrostatic potential map of 4nb-502 (atomic coordinates extracted from the PDB) with the nitro group hydrogen bonded to a water molecule to mimic the ligand being bound by the protein. The colour code spans from +150 (blue) to –150 kJ/mol (red) in eight bands. All distances are in Angstroms (Å).

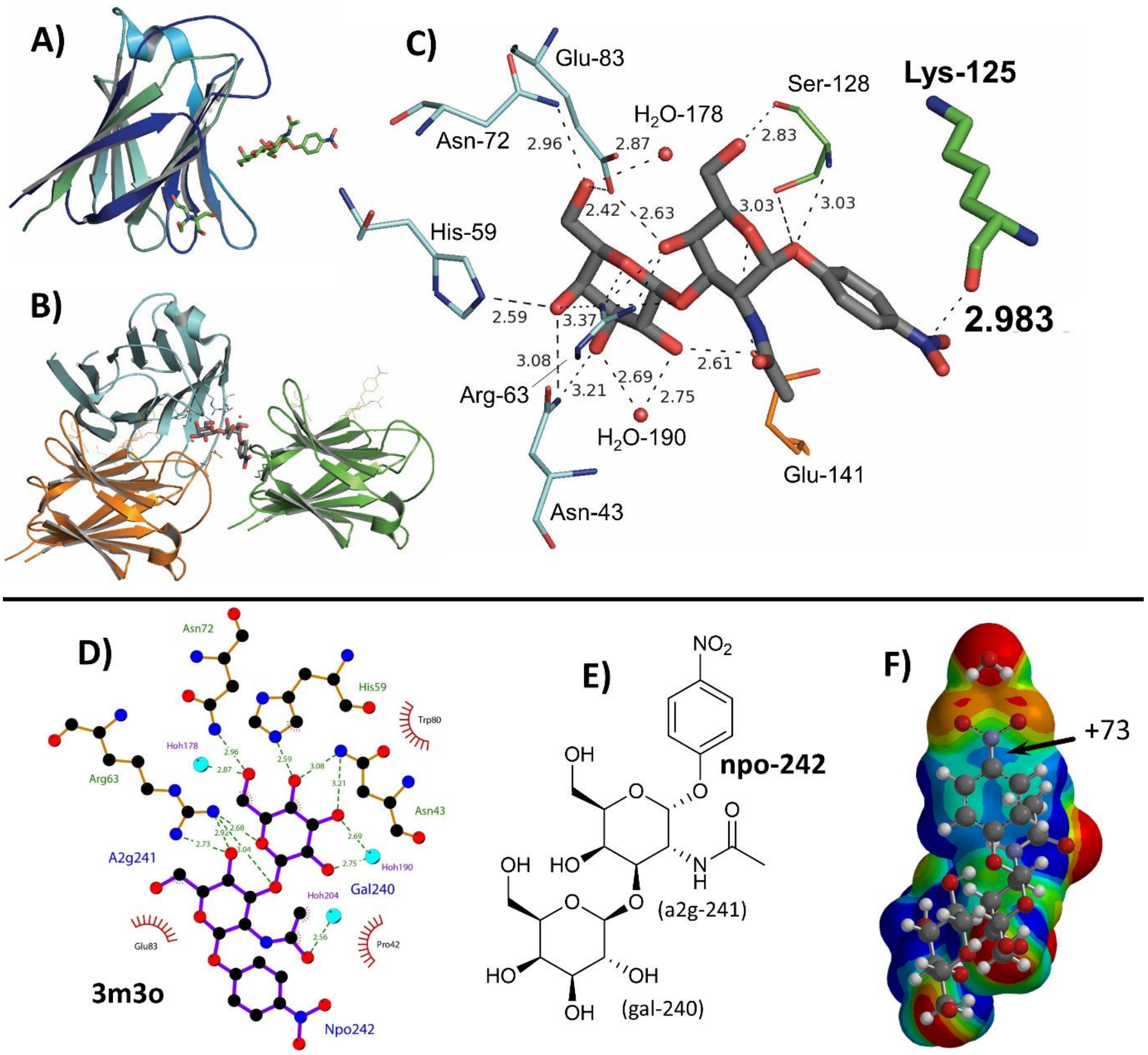

Figure S33. Illustrations of possible Ar-NO_2 π-hole interactions involving protein structure 3m3o.[53] **A)** Cartoon representation of the protein structure with its ligands; **B)** Cartoon representation two protein structures (generated by symmetry and colour coded orange and green) that all surround the ligand npo-0242 (and attached sugar residues, see also E); **C)** Capped stick representation of the binding residues to ligand npo-0242 in 3m3o (4 Å residues around the ligands). The ligand's carbon backbone is represented in grey and the interacting residues are colour coded to identify the (symmetry related) protein chain they belong to (blue, orange, green). The carbonyl O-atom of Lysine-125 belonging to the chain colour coded green is in close proximity to the ligands π-hole region with a $^{Lys\text{-}125}O\cdots N^{NO2}$ = 2.983 Å, which is 0.087 Å below the van der Waals radii of N+O (1.55 + 1.52 = 3.07 Å) and thus indicative of a π-hole interaction; **D)** LigPlus plot of ligand npo-242 in 3m3o. Polar contacts are shown as green striped lines with bonding distance. The polar residues are shown as balls and sticks and the apolar residues as red eye-lashes; **E)** Schematic drawing of the chemical structures of ligand npo-242 (and a2g-241 and gal-240, which are covalently attached to each other); **F)** The molecular electrostatic potential map of npo-242 and attached carbohydrate residues (atomic coordinates extracted from 3m3o) with the nitro group hydrogen bonded to a water molecule to mimic the ligand being bound by the protein. The colour code spans from +125 (blue) to –125 kJ/mol (red) in eight bands. All distances are in Angstroms (Å).

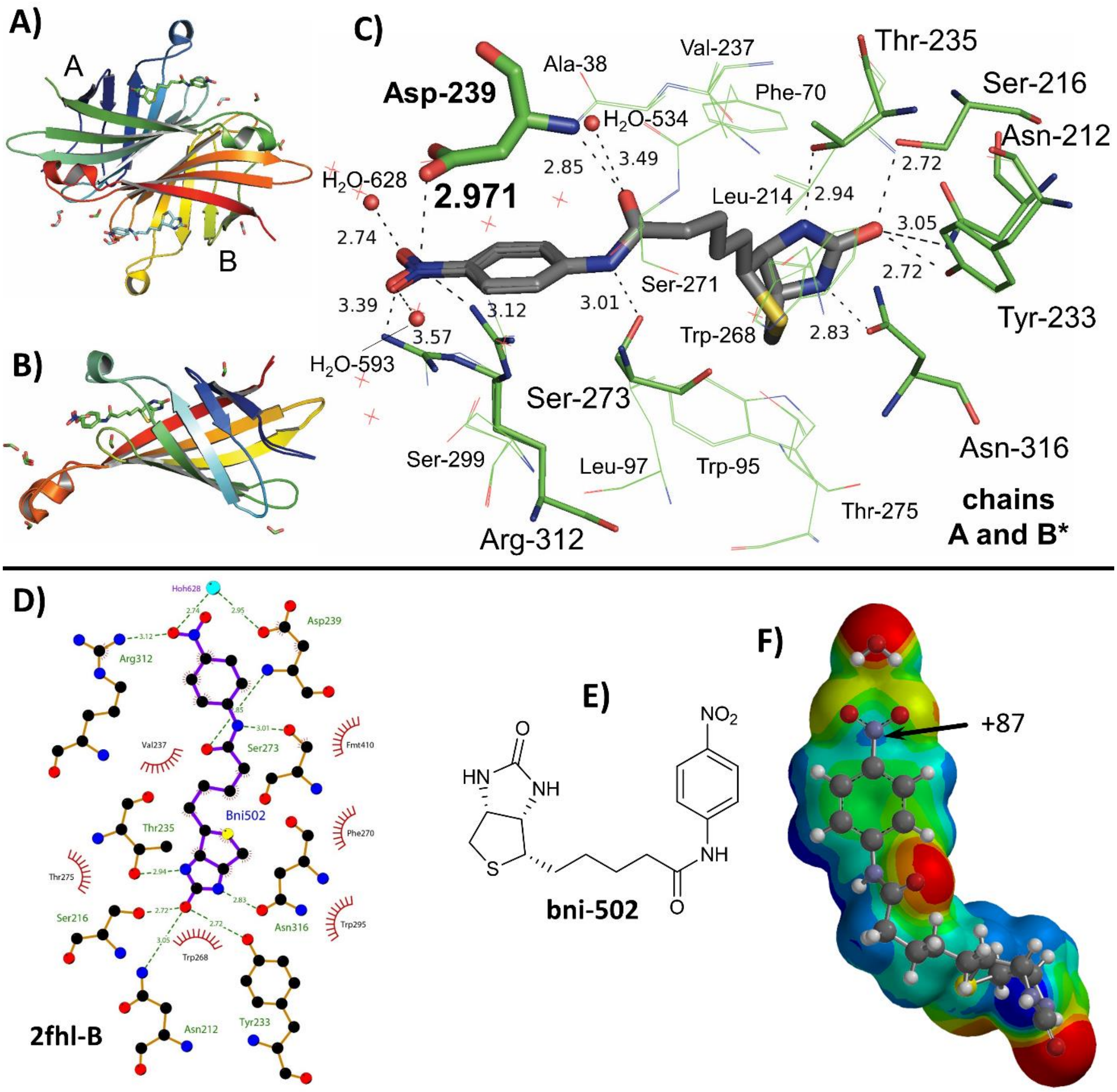

Figure S34. Illustrations of possible Ar-NO2 π-hole interactions involving protein structure 2fhl.[36] **A)** Cartoon representation of protein structure 2fhl. with the ligands in space filling mode; **B)** Cartoon representation of aligned chains A and B of structure 2fhl with the ligands in capped sticks mode; **C)** Capped stick representation of the aligned binding pockets (4 Å residues around the ligand) of ligand bni-502 (shown as thickest sticks with a grey C-backbone, see also E) as found in chains A and B. The residues involved in polar contacts are shown as thinker sticks and the distances shown are taken from chain B (hence the asterisk in the figure). An O-atom of Asparganine-239 (shown as thickest capped sticks) is located very near the π-hole interaction region of the ligand's nitro moieties with a $^{Asp\text{-}239}O\cdots N^{NO2}$ distance of 2.971 Å, which is 0.099 Å within the sum of the van der Waals radii of N+O (1.55 + 1.52 = 3.07 Å) and thus indicative of a π-hole interaction; **D)** LigPlus plot of ligand bni-502 in chain B of 2hfl. Polar contacts are shown as green striped lines with bonding distance. The polar residues are shown as balls and sticks and the apolar residues as red eye-lashes; **E)** Schematic drawing of the chemical structures of ligand bni-502; **F)** The molecular electrostatic potential map of bni-502 (atomic coordinates extracted from chain B of 2fhl) with the nitro group hydrogen bonded to a water molecule to mimic the ligand being bound by the protein. The colour code spans from +150 (blue) to –150 kJ/mol (red) in eight bands. All distances are in Angstroms (Å).

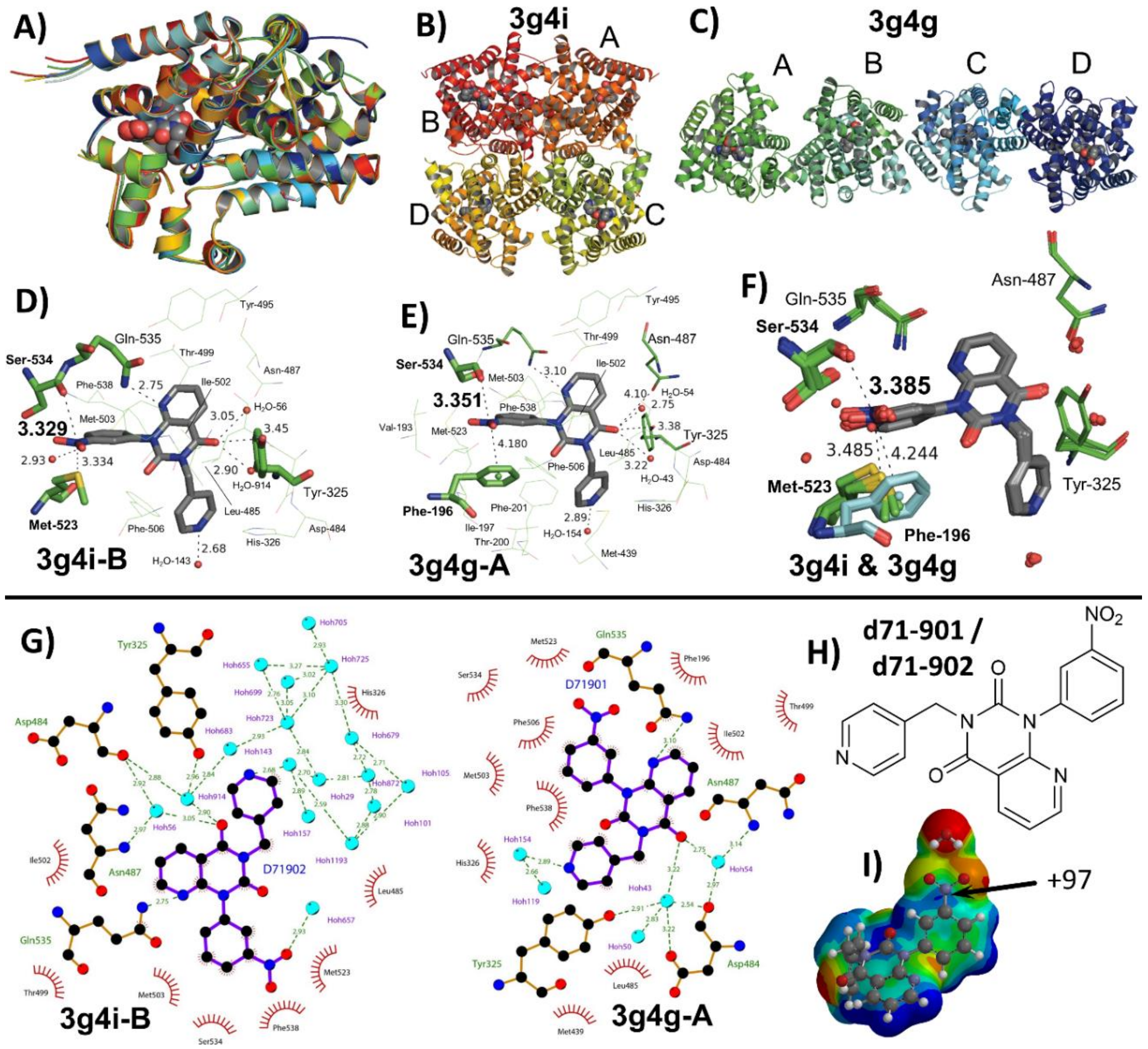

Figure S35. Illustrations of possible Ar-NO_2 π-hole interactions involving protein structures 1d0c and 1d0o.[48] **A)** Cartoon representation of aligned chains A-D from protein structures 3g4i and 3g4g with the ligands in space filling mode; **B)** Cartoon representation of protein structure 3g4i with the ligands in space filling mode and the four chains labelled; **C)** Cartoon representation of protein structure 3g4g with the ligands in space filling mode and the four chains labelled; **D)** Capped stick representation of the binding pocket of ligand d71-902 (see also H) in chain B of 3g4i (4 Å residues around the ligand) wherein the polar contacts are shown as thicker sticks and the ligands C-backbone is in grey. Note that the nitro-group is sandwiched in between the S-atom of methionine-523 ($^{\text{Met-523}}$S···N^{NO2} = 3.334 Å) and an O-atom of a Serine-534 ($^{\text{Ser-534}}$O···N^{NO2} = 3.329 Å); **E)** Capped stick representation of the binding pocket of ligand d71-901 (see also H) in chain A of 3g4g (4 Å residues around the ligand) wherein the polar contacts are shown as thicker sticks and the ligands C-backbone is in grey. Note that the nitro-group is sandwiched in between the aromatic ring of phenylalanine-196 ($^{\text{Phe-196}}$centroid···N^{NO2} = 4.180 Å) and an O-atom of a Serine-534 ($^{\text{Ser-534}}$O···N^{NO2} = 3.351 Å); **F)** Capped stick representation of aligned binding pockets (4 Å residues around the ligands (C-backbone in grey)) of ligands d71-901/d71-902 found in chains A-D in both 3g4i and 3g4g. Only the residues involved in polar contacts are shown and residues that sandwich the nitro group are shown as extra thick sticks. This figure shows that the binding sites are highly preserved and that the O-atom of Serine-534 is always near the nitro moiety with an average $^{\text{Ser-534}}$O···N^{NO2} distance of 3.385 Å. Similarly for the Methionine-523 residues in 3g4i (average $^{\text{Met-523}}$S···N^{NO2} = 3.485 Å) and the Phenylalanine-196 residues in 3g4g (average $^{\text{Phe-196}}$centroid···N^{NO2} = 4.244 Å); **G)** LigPlus plot of ligand d71-902 in chain B of 3g4i (left) and d71-901 in chain A of 3g4g (right). Polar contacts are shown as green striped lines with bonding distance. The polar residues are shown as balls and sticks and the apolar residues as red eye-lashes; **H)** Schematic drawing of the chemical structures of ligands d71-901/d71-902; **I)** The molecular electrostatic potential maps of d71-902 (atomic coordinates extracted from chain B of 3g4i) with the nitro group hydrogen bonded to a water molecule to mimic the ligand being bound by the protein. The colour code spans from +125 (blue) to –125 kJ/mol (red) in eight bands. All distances are in Angstroms (Å).

3.8. π-hole interaction that seem at least structurally relevant with C–S.

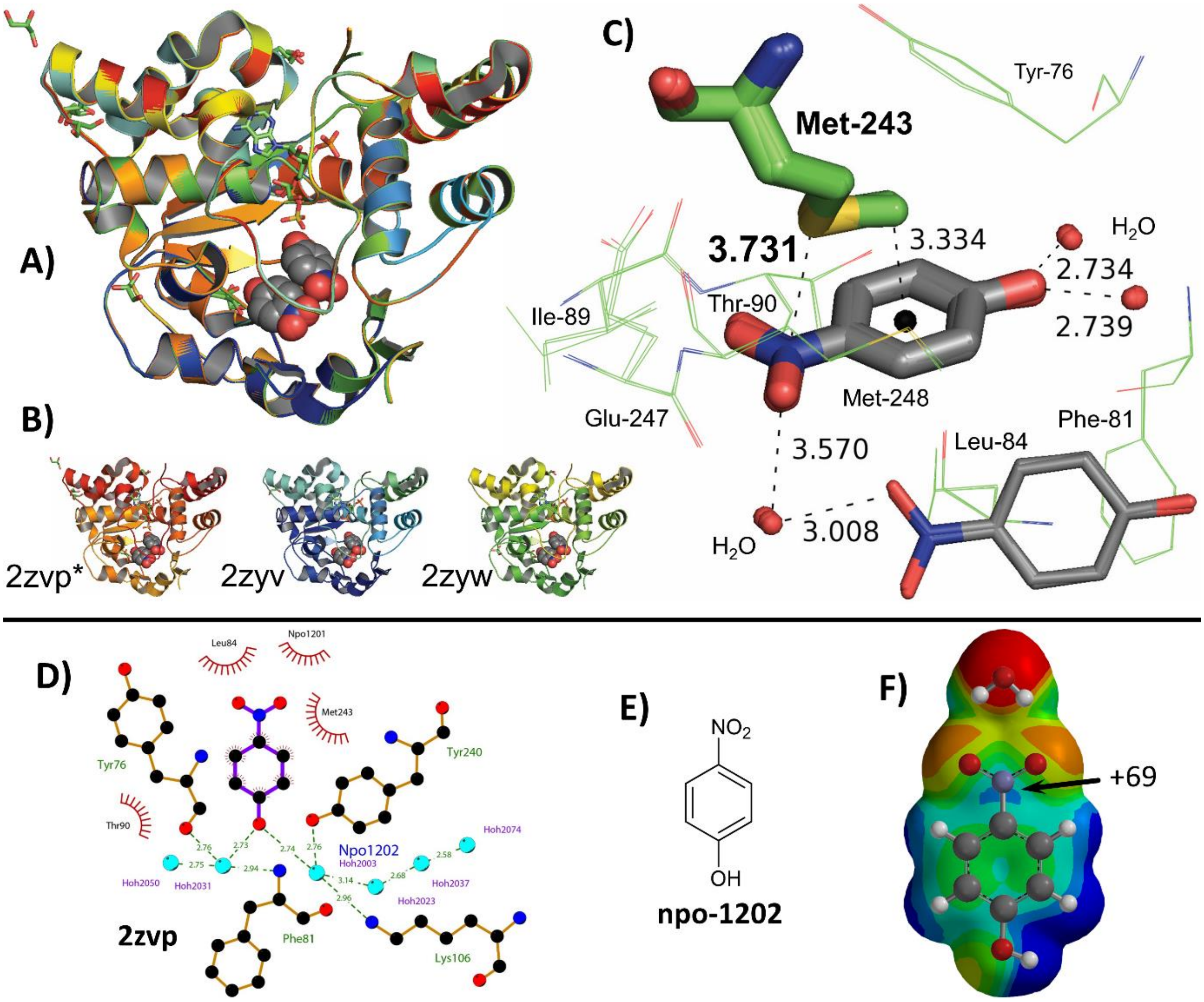

Figure S36. Illustrations of possible Ar-NO₂ π-hole interactions involving protein structures 2zvp,[79] 2zyv[81] and 2zyw.[81] **A)** Cartoon representation of the aligned protein structures; **B)** Cartoon representation of the individual protein structures (the asterisk denotes that the distances in C) are taken from structure 2zvp); **C)** Capped stick representation of aligned binding pockets in 2zvp, 2zyv and 2zyw (4 Å residues around the ligands; distances with 2zvp) and their ligands (npo, see also E), showing that the binding pocket is highly preserved throughout these three structures. One central npo ligand (npo-1202) is H-bonded to water molecules on one side (OH), while the $–NO_2$ moiety seems sandwiched in between a water molecule ('bottom', $^{H2O}O\cdots N^{NO2}$ = 3.570 Å) and a methionine ('top') that has a S-atom near the nitro's π-hole region ($^{Met\text{-}243}S\cdots N^{NO2}$ = 3.731 Å) and its methyl might have a CH-π interaction with npo's aryl ring ($^{Met\text{-}243}C\cdots centroid^{NPO}$ = 3.334 Å); **D)** LigPlus plot of ligand npo-1202 in 2zvp. Polar contacts are shown as green striped lines with bonding distance. The polar residues are shown as balls and sticks and the apolar residues as red eye-lashes; **E)** Schematic drawing of the chemical structures of ligand npo-1202; **F)** The molecular electrostatic potential map of npo-1202 (atomic coordinates extracted from 2zvp) with the nitro group hydrogen bonded to a water molecule to mimic the ligand being bound by the protein. The colour code spans from +125 (blue) to –125 kJ/mol (red) in eight bands. All distances are in Angstroms (Å).

3.9. Cartesian coordinates of computed model nitro aromatics and relevant residues.

3r28 (ΔΔE = –2.1 kcal/mol *vs* Ar-NO)

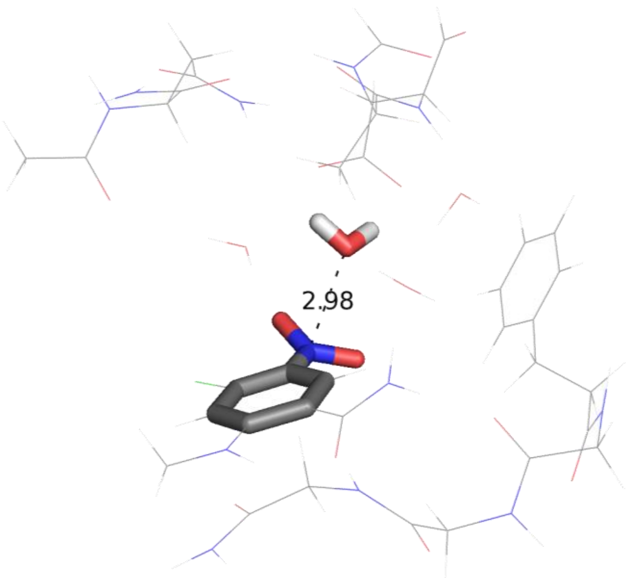

C	-3.87007800	-4.55100000	-4.64307090
C	-2.67007800	-5.40900000	-4.99707090
O	-2.42207800	-6.48600000	-4.41707090
C	-3.36207800	-3.55400000	-3.59907090
C	-4.34507800	-2.50500000	-3.22207090
C	-4.64207800	-1.46800000	-4.09607090
C	-4.93107800	-2.51600000	-1.96107090
C	-5.51407800	-0.44200000	-3.71007090
C	-5.80007800	-1.49800000	-1.57007090
C	-6.08707800	-0.46000000	-2.44907090
N	-1.87707800	-4.94600000	-5.95807090
C	-0.64907800	-5.65900000	-6.31207090
C	0.17292200	-5.58000000	-5.02107090
O	0.16092200	-4.54800000	-4.33107090
C	5.03492200	-5.00500000	-2.53807090
C	5.45292200	-5.33300000	-1.11107090
O	4.73692200	-5.13200000	-0.12407090
C	3.58892200	4.10400000	5.25692910
C	2.82692200	3.77800000	3.97792910
O	2.62792200	2.60700000	3.66792910
N	2.40192200	4.80600000	3.24292910
C	1.65892200	4.60000000	2.00392910
C	2.53392200	4.56500000	0.75392910
O	2.03892200	4.54000000	-0.38307090
C	0.59192200	5.68300000	1.81792910
C	-0.55607800	5.53400000	2.78792910
O	-0.52807800	6.04300000	3.92692910
C	-0.42507800	3.07200000	-2.57407090
C	-1.61907800	3.73500000	-1.89107090
O	-1.37007800	4.67800000	-1.09007090
N	-2.82507800	3.29100000	-2.24407090
C	-4.07307800	3.81800000	-1.67407090
C	-4.67207800	5.09100000	-2.27507090
O	-5.87007800	5.35200000	-2.23407090
C	-3.93107800	4.00600000	-0.16407090
C	-4.16707800	2.73900000	0.68292910
O	-3.69507800	2.77800000	1.85992910
Cl	-0.57307800	-2.15700000	5.04492910
N	2.68192200	-3.97300000	1.60492910
N	-2.41207800	-2.02400000	2.54492910
C	-0.84907800	-2.89800000	1.02492910
C	0.45992200	-3.41100000	0.74492910
C	1.42192200	-3.50400000	1.81392910
C	1.03992200	-3.09800000	3.12592910
C	-0.24407800	-2.60100000	3.40792910
C	-1.22107800	-2.48400000	2.34792910
C	0.76892200	-3.80700000	-0.54607090
C	3.71992200	-4.04500000	2.62692910

O	1.66692200	-4.69000000	-0.76807090
O	-3.43507800	-2.48200000	1.84992910
O	-2.63107800	-1.05000000	3.41292910
H	1.74227410	-3.18611490	3.94996950
H	-1.60642610	-2.81466150	0.25420630
N	0.09340100	-3.26677670	-1.58672130
H	0.25755930	-3.65586350	-2.51865220
H	-0.54147290	-2.46591400	-1.48577270
H	2.86036380	-4.39577320	0.68639430
H	4.62229720	-4.43099060	2.14487000
H	3.93293710	-3.05057400	3.05093740
H	5.62931120	-5.53922060	-3.29180350
H	-3.93989880	5.80300780	-2.73694190
H	-4.83288360	3.02755100	-1.80034090
H	-2.98413200	4.47905210	0.07881460
H	-4.71359770	4.70683790	0.17865810
H	-0.72790730	2.60288770	-3.52008750
C	0.21092200	2.00600000	-1.63907090
H	1.04097610	1.50691630	-2.15538130
H	-0.53107110	1.25530570	-1.33983110
H	0.59288160	2.49218190	-0.73268360
H	-0.87321700	-6.69118210	-6.61263420
H	-3.04037420	-4.11829680	-2.71075350
H	-2.44861740	-3.07075890	-3.98595550
H	-4.27045490	-4.02325010	-5.52252330
H	-4.15487260	-1.42276800	-5.07237280
H	-5.71722850	0.38227860	-4.39582600
H	-4.68585880	-3.31454780	-1.25649890
H	-6.20782550	-1.48624410	-0.55964290
H	-6.71973240	0.36447240	-2.11958420
N	-1.54307800	4.72300000	2.30992910
H	-1.50232450	4.37007400	1.35658030
H	-2.35217370	4.41322210	2.84462680
H	4.36053520	3.32197950	5.33390670
H	1.17752550	3.61105540	2.06068750
H	2.47351850	5.75287730	3.60433560
H	1.04576870	6.67708890	1.95620430
H	0.24017480	5.61166300	0.77756040
N	6.72122570	-5.82375830	-0.98936540
H	7.26306460	-6.10966890	-1.79547650
N	3.63518970	-5.37218920	-2.74086530
H	2.93971070	-5.02269980	-2.05528560
C	3.23892200	-6.31200000	-3.64707090
O	3.98692200	-6.84400000	-4.48407090
N	0.97092200	-6.65400000	-4.73107090
H	0.92473080	-7.47381860	-5.32466820
C	1.74992200	-6.73600000	-3.48907090
H	1.76082950	-7.78327650	-3.15701210
H	1.24768200	-6.12982810	-2.72388630
H	-2.85014660	2.35855930	-2.69871470
H	7.05919300	-6.06346820	-0.06314250

H	-2.01687330	-4.01174760	-6.32521560
N	0.56292200	4.10400000	-2.91807090
H	0.93028540	4.63966110	-2.13163710
C	1.08092200	4.28900000	-4.16507090
H	1.84735790	5.10075850	-4.16276650
O	0.77692200	3.67400000	-5.19707090
H	-1.02867010	0.45454250	2.70516920
O	-0.32407800	0.87500000	2.16992910
H	0.27980560	1.28398140	2.81393570
H	-2.16675240	-0.47404710	-0.43496730
O	-1.96907800	-1.04100000	-1.28707090
H	-2.82048680	-1.50132640	-1.43047810
H	-3.65183550	0.72614760	0.64726030
O	-2.71407800	0.38800000	0.81492910
H	-2.23690360	1.16137700	1.16492730
H	-2.32018050	0.26285720	-2.34726120
O	-2.82207800	0.92000000	-2.90807090
H	-3.74710840	0.63346230	-2.78676570
O	-4.78907800	1.75900000	0.12592910
H	-0.13476230	-5.12252210	-7.11791370
H	-4.65443370	-5.19502680	-4.22840550
H	5.17646350	-3.92091930	-2.68592300
H	3.43740680	-4.72339930	3.45088260
N	3.89092200	4.58700000	0.94692910
H	4.33235840	4.55509050	1.86435060
H	4.47152950	4.49681790	0.12114590
H	4.07693285	5.05619869	5.24898809
H	2.91717177	4.04362575	6.08759545

1eei (ΔΔE = –1.5 kcal/mol *vs* Ar-NO; –3.5 kcal/mol *vs* Ar-H)

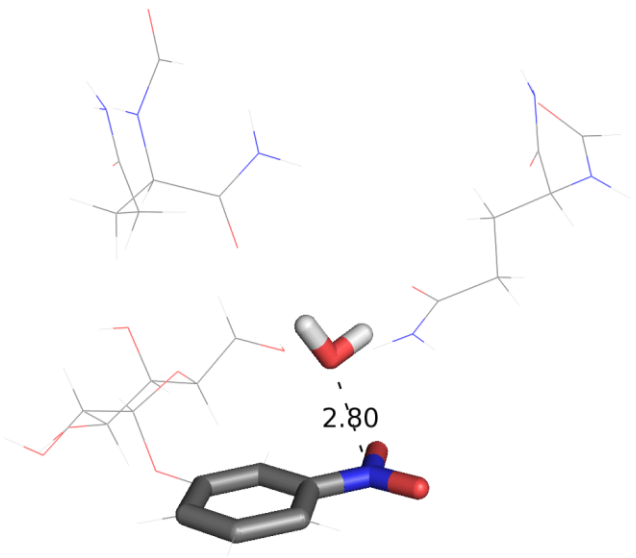

C	-0.3887176	-3.4409647	2.0023294
C	0.2352824	-2.0689647	1.8833294
O	0.6172824	-1.6329647	0.8023294
C	-1.7197176	-3.4859647	1.2113294
C	-2.6887176	-2.3169647	1.4113294
C	-3.6327176	-2.5099647	2.5763294
O	-4.7537176	-2.0109647	2.5463294
C	2.3962824	5.7980353	3.3293294
C	1.3972824	6.1290353	4.4173294
O	0.4142824	6.8410353	4.1613294
C	1.8652824	4.6190353	2.5343294
C	2.4162824	4.5050353	1.1283294
C	1.8052824	3.3390353	0.3673294
O	2.1882824	2.1920353	0.5693294
C	-1.1367176	-2.6079647	-3.1686706
N	2.6562824	1.4110353	-3.7586706
O	-0.7507176	-2.0099647	-4.4206706
C	-2.5707176	-3.0679647	-3.3686706
C	-3.4677176	-1.8329647	-3.6666706
C	-3.3447176	-0.8689647	-2.5006706
C	-1.9007176	-0.4989647	-2.3806706
O	-1.0407176	-1.6369647	-2.1306706
C	-1.8147176	0.3390353	-1.1786706
C	0.6212824	-1.6069647	-4.5386706
O	3.8372824	1.8150353	-3.8226706
C	0.9442824	-0.3449647	-4.1106706
O	1.8012824	2.1950353	-3.2686706
C	2.2762824	0.0740353	-4.2156706
C	3.2372824	-0.8219647	-4.7666706
C	2.8582824	-2.1049647	-5.1966706
C	1.5202824	-2.5109647	-5.0826706
H	0.2230408	0.3242148	-3.6527499
H	1.1737899	-3.4930958	-5.4006309
H	3.6099740	-2.7717834	-5.6177099
H	4.2715202	-0.4875771	-4.8373565
H	-0.4562371	-3.4295320	-2.8918483
H	-2.9183819	-3.5605507	-2.4465296
H	-3.0148163	-1.3434999	-4.5497522
H	-3.9118255	0.0521011	-2.7446294
H	-1.5982279	0.0599205	-3.2742747
H	-2.5286500	1.1743625	-1.2044620
H	-1.9612161	-0.2373540	-0.2560535
O	-2.5507176	-3.9369647	-4.4786706
H	-2.8787640	-4.8091521	-4.2092631
O	-4.8257176	-2.0969647	-3.9396706
H	-4.8539637	-2.7892802	-4.6250498
O	-3.8217176	-1.4529647	-1.3096706
H	-4.7499542	-1.7048113	-1.4663473
O	-0.5157176	0.8720353	-1.3086706
H	0.1901592	0.2057687	-1.1584796

H	2.2468896	5.4515956	0.5872823
H	3.5020049	4.3298808	1.1537261
H	2.0567488	3.6899580	3.0913193
H	0.7716756	4.7385573	2.4813565
H	2.3864739	6.6887245	2.6838193
H	0.3030664	-4.0548725	1.4063973
H	-1.4123023	-3.4624458	0.1548607
H	-2.2075439	-4.4580756	1.3779307
H	-2.1359763	-1.3772117	1.5680937
H	-3.3047543	-2.1655564	0.5234418
N	1.6112824	5.5590353	5.6483294
H	0.8700238	5.6706493	6.3324712
H	2.2788341	4.7869382	5.7411364
N	0.3522824	-1.2729647	3.0043294
H	0.8176192	-0.3798445	2.8687764
H	0.1530836	-1.5779590	3.9467214
N	0.7462824	3.6040353	-0.4566706
H	0.3276141	2.8091882	-0.9543893
H	0.5240640	4.5472276	-0.7482211
N	-3.2427176	-3.2869647	3.6663294
H	-4.0001824	-3.4960226	4.3112705
H	-2.5444319	-4.0139939	3.5484414
N	3.8252824	5.6400353	3.7293294
H	4.4765983	6.3147093	3.3410696
C	4.3802824	4.6380353	4.4763294
H	5.4887362	4.7164634	4.5156972
O	3.7632824	3.7390353	5.0683294
N	-0.3267176	-4.1389647	3.3333294
H	0.1668507	-5.0297823	3.2939552
C	-0.4637176	-3.7219647	4.6533294
H	-0.9850257	-2.7500414	4.7770423
O	-0.0957176	-4.4079647	5.6063294
H	2.2462925	-0.6530805	-0.7019439
O	2.2762824	0.1120353	-1.3036706
H	2.2788192	0.8862721	-0.6855152

4ki7 (ΔΔE = –1.5 kcal/mol *vs* Ar-NO)

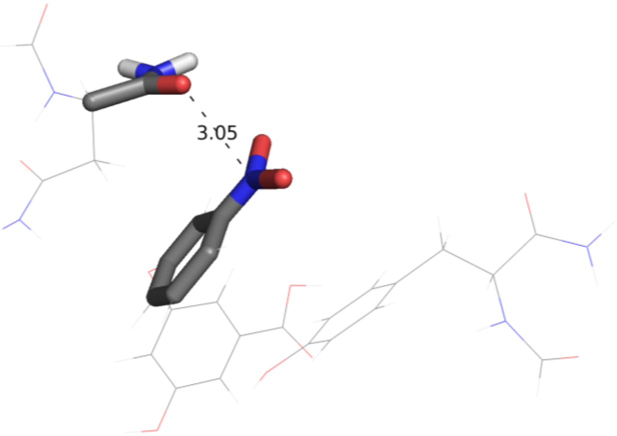

C	-4.73865790	0.94032890	3.37202630
C	-3.75865790	2.06232890	3.13402630
O	-2.66065790	2.04532890	3.70202630
C	-4.14365790	-0.32867110	2.76502630
C	-4.91965790	-1.54867110	3.13902630
O	-5.12165790	-1.80967110	4.33302630
C	6.01534210	1.76332890	-0.14897370
C	6.69834210	3.05832890	0.31902630
O	6.05534210	4.10732890	0.39302630
C	4.64634210	1.65332890	0.50902630
C	3.77234210	0.54932890	-0.02397370
C	3.02234210	0.73332890	-1.18297370
C	3.66534210	-0.67067110	0.64802630
C	2.20134210	-0.27067110	-1.67497370
C	2.84634210	-1.68867110	0.16902630
C	2.11234210	-1.48667110	-0.99697370
C	-1.09665790	-2.38967110	1.18302630
C	-1.90465790	-2.17367110	0.06002630
C	-0.57065790	-1.30167110	1.85302630
C	-2.48165790	-2.39267110	-3.47797370
C	-0.78465790	-1.72967110	-5.09497370
C	-1.34365790	-0.27667110	-3.21797370
C	-1.67665790	0.19132890	0.24802630
C	0.33734210	0.38332890	-4.83797370
C	-1.72365790	-2.66167110	-4.65497370
C	-2.21865790	-0.90867110	-0.42797370
C	-2.29965790	-1.18867110	-2.72697370
C	-0.60265790	-0.53467110	-4.38397370
C	-0.86165790	-0.02467110	1.38502630
N	-0.30965790	0.96532890	2.08402630
O	0.71834210	0.62032890	3.22402630
O	1.45034210	-0.03567110	-5.25797370
O	-0.65665790	2.26332890	1.77002630
O	-3.01365790	-0.84767110	-1.54997370
H	0.05928070	-1.39179600	2.73323720
H	-0.89975950	-3.40535490	1.52487420
H	-2.30397110	-3.03362250	-0.48265680
H	-1.86930090	1.21231750	-0.07520870
H	-3.22443360	-3.11919900	-3.13676020
H	-0.19375890	-1.92752490	-5.98764450
H	-1.18449640	0.65977320	-2.68364680
H	2.76411310	-2.63225910	0.71445080
H	4.19298070	-0.81600670	1.59414060
H	3.08102720	1.68457200	-1.71600200
H	1.63513450	-0.14219620	-2.59507780
O	1.27434210	-2.45467110	-1.53197370
H	1.29169960	-3.23001890	-0.94430670
O	-1.88965790	-3.81367110	-5.38697370
H	-2.58772210	-4.35553950	-4.97875090
H	4.79180830	1.52883220	1.59421360

H	4.15804950	2.62824920	0.38058740
H	5.89320970	1.88402030	-1.24070580
H	-4.81149690	0.81193160	4.46226870
H	-4.04053820	-0.20397220	1.67455930
H	-3.13082380	-0.43425380	3.17895360
N	6.75334210	0.48932890	0.07902630
H	6.18849310	-0.26102290	0.47194120
N	8.04234210	2.98532890	0.62002630
H	8.48081890	3.89094430	0.76670130
H	8.59828300	2.27599120	0.13270480
C	7.91234210	0.08432890	-0.52397370
H	8.10372890	-0.99989690	-0.36456190
O	0.03134210	1.71232890	-4.72497370
H	0.83545610	2.18911910	-5.02059080
N	-5.44565790	-2.30567110	2.09902630
H	-5.85594170	-3.20016460	2.35186960
H	-5.06501770	-2.21052160	1.16299660
N	-4.09565790	3.00932890	2.19102630
H	-5.06964950	3.13088850	1.93613250
H	-3.47354390	3.80882740	2.12490290
N	-6.10365790	1.17432890	2.83802630
H	-6.47906060	0.49173950	2.18301930
C	-7.01965790	1.94232890	3.53602630
H	-8.04951930	1.84430290	3.12220480
O	-6.73565790	2.68032890	4.47602630
O	8.70634210	0.81032890	-1.13997370

1gnr (ΔΔE = –2.6 kcal/mol *vs* Ar-NO)

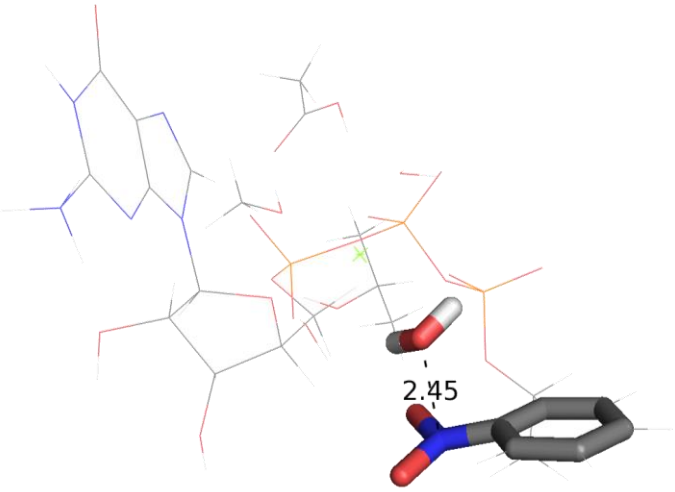

C	-1.4867704	-2.1826189	-3.4806130
O	-1.2767704	-3.4826189	-2.9866130
H	-1.2758508	-4.1524590	-3.7316471
H	-1.0169048	-2.0645680	-4.4701013
H	-2.5640730	-1.9700918	-3.5641991
C	-2.0427704	-7.5496189	0.0883870
C	-1.3677704	-6.3786189	-0.6446130
C	-1.0387704	-6.7686189	-2.0716130
O	-2.2277704	-5.2316189	-0.6396130
H	-2.7703066	-5.1957892	-1.4494183
H	-1.9596281	-7.0305269	-2.6135701
H	-0.5511917	-5.9677010	-2.6284558
H	-0.3732051	-7.6427139	-2.0964194
H	-0.4620843	-6.0940559	-0.0696464
H	-2.9431418	-7.8609681	-0.4614023
C	0.2852296	-6.6546189	-6.2476130
C	0.1282296	-5.6296189	-5.1546130
O	1.1392296	-5.1246189	-4.6176130
O	-1.0367704	-5.3066189	-4.8606130
H	-0.4014884	-6.4031365	-7.0622672
H	1.3144633	-6.7332930	-6.6176685
H	0.9938071	-4.6675642	-3.7446963
C	0.0322296	-3.3536189	5.4153870
P	-0.4257704	0.7193811	-0.7696130
P	1.6892296	-1.1226189	-0.2086130
P	1.6662296	-2.9876189	1.8683870
C	2.6022296	-1.3626189	5.8463870
C	2.9352296	-0.2446189	6.5363870
O	-0.4417704	2.2813811	-0.3936130
C	2.1972296	0.8753811	6.3873870
O	0.3362296	5.0633811	0.6273870
C	1.1472296	0.8623811	5.5363870
O	-2.6407704	5.1013811	1.7833870
C	0.7762296	-0.2726189	4.9013870
N	-0.4547704	-0.2046189	4.2593870
O	-2.7957704	5.7473811	-1.0376130
C	1.5252296	-1.4056189	5.0213870
C	-0.5067704	5.7813811	-0.2626130
N	1.0942296	9.2173811	-3.4816130
O	-0.5147704	0.6723811	-2.2496130
O	3.1422296	-1.0036189	-0.4876130
O	1.0262296	-3.8236189	0.8583870
C	0.5782296	9.3583811	-2.2196130
C	-1.7337704	4.9433811	-0.5206130
O	-1.4257704	-0.0056189	0.0363870
O	0.8602296	-1.9826189	-1.0776130
O	3.0422296	-3.2866189	2.2763870
C	-1.9307704	4.2883811	0.8363870
N	0.2832296	8.3463811	-1.4206130
O	1.0642296	0.3643811	-0.2226130

O	1.5342296	-1.4576189	1.3753870
O	0.7152296	-2.8696189	3.1543870
C	0.4892296	7.1653811	-2.0456130
C	-0.4817704	4.0783811	1.2673870
C	1.0032296	6.9313811	-3.2956130
C	-0.0387704	2.6683811	0.9153870
C	1.4322296	8.0233811	-4.0816130
O	2.1362296	8.0233811	-5.0906130
N	1.0462296	5.5623811	-3.5596130
C	0.5862296	5.0333811	-2.4596130
N	0.1632296	5.9533811	-1.5386130
C	1.1302296	-2.7676189	4.5133870
O	-0.8587704	-1.1776189	3.3423870
O	-1.2517704	0.9323811	4.4573870
H	-0.7664857	6.7842903	0.1116295
H	-0.8915245	-2.7677058	5.3336628
H	-0.1797104	-4.3845259	5.1010414
H	0.3681989	-3.3584279	6.4614401
H	-3.3203383	6.0115625	-0.2550157
H	2.4622087	1.7970864	6.9086057
H	-3.2497991	4.5202685	2.2643992
H	3.2060592	-2.2668125	5.9477167
H	0.5472606	1.7534842	5.3736488
H	1.4067455	10.0285723	-4.0199675
H	-1.5138946	4.1407989	-1.2347232
H	3.7978271	-0.2594375	7.2067728
H	0.4792358	3.9627156	-2.2699307
H	2.0346738	-3.3931478	4.6120651
H	-2.4337472	3.3178494	0.7198255
H	-0.3873838	4.2044461	2.3564862
H	1.0470184	2.5681360	1.0283052
H	-0.5129323	2.0189932	1.6599238
Mg	-0.4347704	-3.8346189	-0.9716130
O	-2.1387704	-2.3946189	-0.1996130
H	-1.8197065	-1.4019333	-0.1668718
H	-2.3122380	-2.5906671	0.7370953
O	1.4012296	-4.6756189	-2.0406130
H	1.9532121	-5.2644865	-1.4913709
H	1.7439934	-3.7573006	-1.8146778
O	0.8872296	0.6413811	2.3923870
H	1.6977490	0.1363797	2.2251470
H	0.2061869	0.0592452	2.0125872
H	-2.3365187	-7.2461845	1.1010377
H	-1.3608888	-8.4080203	0.1617086
H	-0.0202087	-7.6278255	-5.8383381
H	-1.0333656	-1.4433973	-2.8061770
N	0.3222296	10.6983811	-1.6306130
H	1.1710752	11.2838981	-1.5306336
H	-0.0453529	10.4795073	-0.6865715
H	-0.3901065	11.2437971	-2.1477798

1gvs (ΔΔE = –1.7 kcal/mol *vs* Ar-NO)

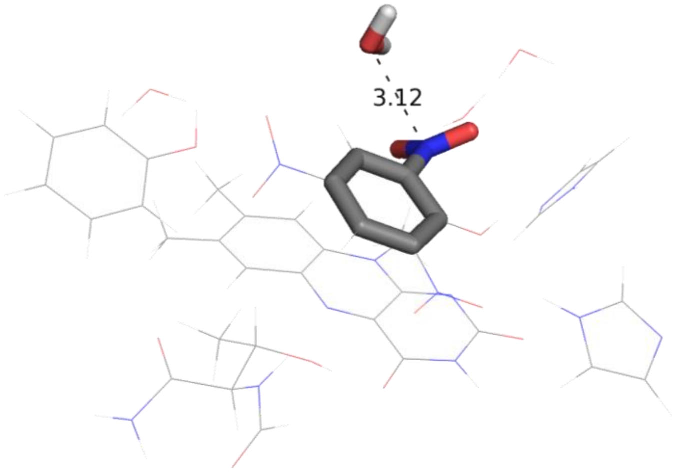

N	-2.5207100	3.2962888	-0.8651535
C	-2.4087100	3.4762888	-2.3101535
C	-3.7077100	2.9862888	-2.9611535
O	-4.1277100	1.8472888	-2.7291535
C	-1.2167100	2.6612888	-2.8531535
C	-1.2317100	2.6062888	-4.3791535
O	0.0002900	3.2602888	-2.4001535
H	-0.0307539	3.4259038	-1.4291811
H	-2.2325977	4.5383598	-2.5444889
H	-1.3096450	1.6255572	-2.4650056
H	-0.3209875	2.1073973	-4.7335778
H	-1.2664489	3.6179695	-4.8061146
H	-1.8399914	2.6682252	-0.4194866
H	-2.0968831	2.0343905	-4.7366875
C	6.5372900	4.7332888	2.2658465
C	5.5472900	4.2662888	1.4698465
N	6.8882900	3.6892888	3.0948465
C	6.1452900	2.6332888	2.8098465
N	5.3242900	2.9572888	1.8268465
H	4.6219116	2.3661240	1.3921974
H	4.9852467	4.7050192	0.6510605
H	6.1590023	1.6733295	3.3150416
C	5.2842900	-1.1627112	3.8718465
C	5.4032900	-1.6367112	5.1358465
N	4.0722900	-0.5047112	3.8248465
C	3.4852900	-0.5817112	5.0068465
N	4.2712900	-1.2637112	5.8198465
H	4.0781952	-1.4906261	6.7896070
H	6.1961896	-2.1865997	5.6318612
H	2.5321278	-0.1289795	5.2534570
C	-4.7577100	-1.8517112	-5.7631535
C	-3.9927100	-0.8887112	-5.1131535
C	-4.7567100	-3.1527112	-5.2541535
C	-3.2407100	-1.2097112	-3.9861535
C	-4.0077100	-3.4827112	-4.1261535
C	-3.2527100	-2.5067112	-3.4991535
O	-2.5107100	-2.8207112	-2.3811535
H	-2.3504060	-3.7796030	-2.3732573
H	-3.9762447	0.1404117	-5.4740004
H	-5.3435040	-3.9295344	-5.7472416
H	-2.6533633	-0.4449233	-3.4796627
H	-4.0124863	-4.5061050	-3.7435255
C	-0.5077100	-0.6337112	4.2178465
N	1.2432900	1.3302888	3.3848465
C	1.9552900	2.4892888	3.2168465
O	2.8572900	2.8352888	3.9478465
N	1.6772900	3.3022888	2.0558465
C	0.6472900	3.0952888	1.1838465
C	-0.1477100	1.8762888	1.4068465
O	0.4442900	3.8692888	0.2418465

C	-1.7447100	0.3242888	0.7368465
N	-0.9657100	1.4642888	0.4808465
C	-2.8847100	0.0242888	-0.1261535
C	-3.7857100	-0.9727112	0.1238465
C	-4.8937100	-1.1977112	-0.6781535
C	-3.6247100	-1.7947112	1.3468465
C	-4.6237100	-2.8677112	1.7188465
C	-2.5257100	-1.5377112	2.1668465
C	-1.6397100	-0.4487112	1.9638465
C	0.1792900	1.0822888	2.6238465
N	-0.6267100	-0.0107112	2.8808465
H	-2.3942482	-2.1476520	3.0617633
H	0.0761128	-1.5609566	4.1652851
H	-3.0250349	0.6577770	-1.0063545
H	-4.8998641	-2.2170646	-1.1040846
H	-4.3245678	-3.3951640	2.6323782
H	-4.7382700	-3.6006162	0.9058226
H	0.0069207	0.0767834	4.8696523
H	-4.9511494	-0.5090218	-1.5325739
H	-5.6181421	-2.4226980	1.8789966
H	-5.8456802	-1.1077837	-0.1254493
H	2.2687805	4.1226891	1.9218957
C	2.6452900	0.0002888	0.2868465
O	3.3142900	0.4352888	1.2408465
C	1.9762900	-1.3197112	0.2628465
N	2.1002900	-2.1957112	1.4258465
C	1.2192900	-1.8107112	-0.8271535
C	1.0812900	-1.0057112	-1.9641535
N	0.3122900	-1.4847112	-3.1341535
C	1.6682900	0.2552888	-2.0231535
C	2.4182900	0.7452888	-0.9491535
N	2.9912900	2.0952888	-1.1131535
O	1.0602900	-2.5817112	1.9888465
O	3.2082900	-2.4577112	1.8308465
O	0.2232900	-2.7097112	-3.3001535
O	-0.1027100	-0.6417112	-3.8971535
O	2.8182900	2.6662888	-2.1961535
O	3.6892900	2.5372888	-0.2441535
H	0.7860114	-2.8102022	-0.7695585
H	1.5231468	0.9122191	-2.8801376
H	3.4559618	-0.0428063	2.1722887
O	-0.9917100	-3.1027112	-6.3171535
H	-1.8836045	-2.7128827	-6.2900140
H	-0.6316736	-2.8846563	-5.4369300
O	1.5362900	-5.0097112	0.1958465
H	2.3141553	-5.5214371	-0.0821666
H	1.4278539	-5.2217996	1.1383069
O	1.7582900	-3.8367112	4.9118465
H	1.5234151	-3.4859069	4.0310972
H	2.6182152	-4.2661812	4.7388711
O	4.5652900	-4.5827112	2.9328465

H	5.2412865	-4.0910483	3.4295979
H	4.0018053	-3.8740742	2.5673362
C	-3.1267100	4.2962888	-0.0701535
H	-2.9439082	4.1698857	1.0185751
O	-3.8137100	5.1612888	-0.5821535
H	-5.3422190	-1.6014294	-6.6478452
H	-1.5123870	-0.8488068	4.5984895
N	-4.3927100	3.9242888	-3.8641535
H	-4.2776093	4.8998485	-3.5977149
H	-5.3523272	3.6451853	-4.0559921
H	5.9489762	-1.2320910	3.0195540
H	7.0165621	5.7042043	2.3121949

2y59 (ΔΔE = –1.0 kcal/mol *vs* Ar-NO)

C	1.4139103	-1.5307821	-4.6807564
C	2.4129103	-1.7747821	-3.5477564
O	3.4989103	-2.2937821	-3.7917564
H	0.5861206	-0.8608112	-4.4084212
N	2.0369103	-1.4317821	-2.3237564
C	2.9389103	-1.5027821	-1.1637564
C	2.2419103	-0.9157821	0.0892436
O	3.0859103	-0.8007821	1.2282436
H	1.3793070	-1.5535048	0.3154140
H	1.2477748	-0.7899377	-2.2260317
H	3.8706847	-0.9529614	-1.3757398
H	1.8523123	0.0828961	-0.1824640
C	3.3969103	0.8242179	4.3332436
O	3.6429103	0.2362179	3.0722436
H	2.5834659	1.5770430	4.3080452
H	4.3122941	1.3329953	4.6719999
H	3.1257236	0.0832551	5.1173055
C	3.8749103	-2.6247821	3.5422436
N	2.5459103	-2.0947821	3.2162436
H	1.9612110	-2.1260027	4.0609078
H	4.5034416	-2.5322799	2.6499380
H	2.0426503	-2.7052713	2.5383229
H	3.8104840	-3.6772687	3.8479297
H	4.3252183	-2.0255690	4.3405201
C	1.0619103	0.0912179	2.5432436
N	1.0059103	1.3632179	1.8152436
C	-0.0410897	1.8702179	1.1362436
O	-1.0820897	1.2432179	1.0452436
C	0.0589103	3.1972179	0.4122436
C	-0.1200897	4.3852179	1.1082436
C	-0.0450897	5.6012179	0.4352436
C	0.2139103	5.6362179	-0.9447564
C	0.3989103	4.4472179	-1.6407564
C	0.3149103	3.2292179	-0.9667564
N	0.5039103	2.0722179	-1.6737564
O	0.7849103	2.0782179	-3.0297564
O	0.4119103	0.8262179	-1.0737564
B	2.5369103	-0.5547821	2.5452436
H	0.6007564	4.4317581	-2.7096786
H	0.3360874	-0.5908424	2.0805993
H	1.8813909	1.8845631	1.7920042
H	-0.3095069	4.3511643	2.1810048
H	-0.1815031	6.5336419	0.9846412
H	0.2760709	6.5882708	-1.4705414
H	0.6684492	0.2571770	3.5657580
C	-0.9860897	-4.3047821	3.1672436
C	-0.4660897	-3.4987821	1.9672436
O	0.7339103	-3.4917821	1.6952436
H	-0.5494025	-3.8827510	4.0828254
H	-0.6268196	-5.3366396	3.0694346

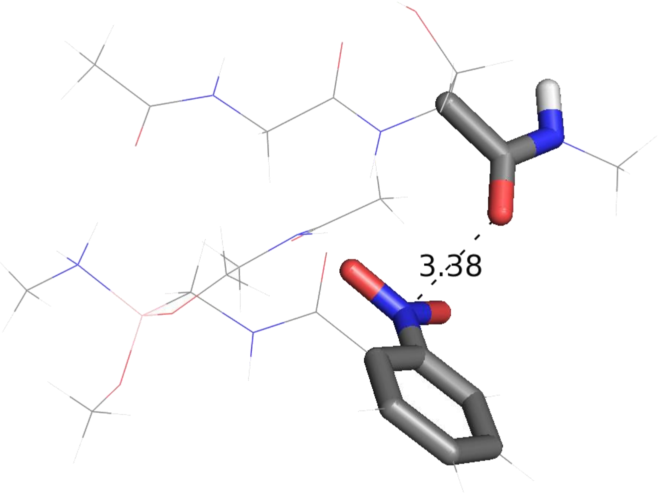

H	-2.0791574	-4.3052699	3.2646609
N	-1.3740897	-2.8157821	1.2662436
C	-0.9980897	-1.9947821	0.1222436
C	-2.1910897	-1.6737821	-0.7597564
O	-2.8180897	-2.5797821	-1.3317564
H	-0.2686573	-2.5458251	-0.4893736
H	-2.3612687	-2.9201735	1.4779399
H	-0.5321296	-1.0647266	0.4668125
N	-2.5080897	-0.3837821	-0.8697564
C	-3.6560897	0.0792179	-1.6357564
C	-3.3870897	1.3872179	-2.3797564
O	-2.8610897	2.3432179	-1.8227564
C	-4.8760897	0.3312179	-0.7267564
O	-5.3680897	-0.9087821	-0.2297564
H	-4.8934023	-1.6339060	-0.6783586
H	-1.9905993	0.3277774	-0.3306132
H	-3.9131092	-0.7283167	-2.3392215
H	-4.5793379	0.9821574	0.1106832
N	-3.7500897	1.3752179	-3.6557564
C	-3.9790897	2.5672179	-4.4637564
H	-3.4966971	2.4665683	-5.4451156
H	-4.1619900	0.5157241	-4.0069532
H	-3.5370271	3.4199978	-3.9363833
H	-5.0557749	2.7451598	-4.6042423
H	1.0028469	-2.5006044	-4.9939293
H	1.9624551	-1.1063503	-5.5295119
H	3.2220813	-2.5511684	-0.9880450
H	-5.6646731	0.8458570	-1.3078941

4qo7 (ΔΔE = –0.2 kcal/mol *vs* Ar-NO)

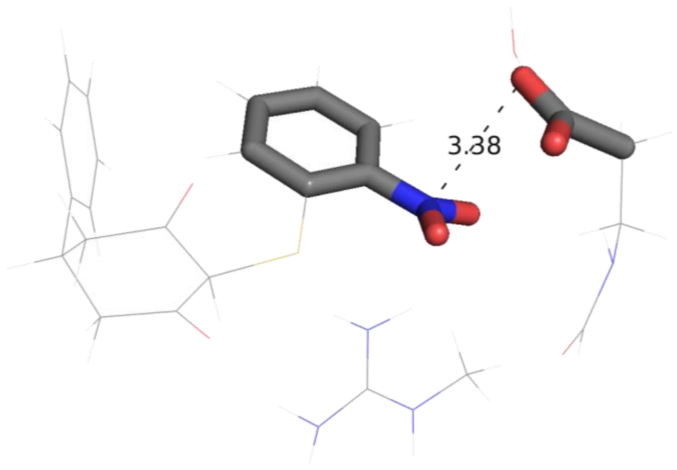

N	-4.3949710	-3.9491594	-0.5118406
C	-5.1779710	-2.8191594	-1.0258406
C	-5.0029710	-2.6551594	-2.5528406
C	-3.5319710	-2.6521594	-2.9958406
O	-3.0919710	-1.6621594	-3.6118406
O	-2.8109710	-3.6421594	-2.7488406
H	-5.4927496	-1.7240340	-2.8663904
H	-4.8521485	-1.9021763	-0.5138266
H	-5.4996698	-3.4944002	-3.0677584
H	-3.7822144	-4.3816937	-1.2263293
C	-3.9469710	0.1008406	3.2311594
N	-2.6959710	-0.0251594	3.9891594
C	-1.4729710	-0.1291594	3.4621594
N	-0.4249710	-0.2231594	4.2651594
N	-1.2759710	-0.1461594	2.1501594
H	-0.3395888	-0.3601310	1.7609957
H	-4.7493098	0.3247792	3.9404433
H	-2.7780306	-0.2240735	4.9797174
H	-0.4983026	0.1139674	5.2181635
H	-2.0714484	-0.4271777	1.5801890
H	-3.8681810	0.9303887	2.5156261
H	0.4960931	-0.1118288	3.8310737
C	4.0660290	3.9618406	-1.6348406
C	3.7000290	3.9598406	-0.2938406
C	4.0670290	2.9018406	0.5321594
C	4.8070290	1.8328406	0.0361594
C	5.1690290	1.8558406	-1.3128406
C	4.8000290	2.9038406	-2.1498406
C	5.2190290	0.6588406	0.9311594
C	4.3170290	0.5848406	2.1671594
C	2.8970290	0.1438406	1.8781594
O	1.9590290	0.5348406	2.5861594
C	2.7010290	-0.9251594	0.8371594
C	3.7780290	-1.1021594	-0.1958406
O	3.5830290	-1.7571594	-1.2228406
C	5.1880290	-0.6961594	0.2081594
S	0.9840290	-1.1211594	0.2431594
C	0.3590290	0.2508406	-0.8008406
C	1.2710290	1.2018406	-1.2678406
C	0.8220290	2.3108406	-1.9638406
C	-0.5319710	2.4738406	-2.2058406
C	-1.4429710	1.5338406	-1.7618406
C	-1.0019710	0.4258406	-1.0648406
N	-2.0069710	-0.5571594	-0.6118406
O	-3.1639710	-0.2111594	-0.4448406
O	-1.6829710	-1.7121594	-0.3948406
H	-2.5055489	1.5959513	-2.0183414
H	3.7689299	4.7898343	-2.2805212
H	3.1111559	4.7823217	0.1136838
H	3.7413618	2.9314650	1.5698661

H	5.7425166	1.0317487	-1.7373054
H	5.0874430	2.8862644	-3.2013932
H	6.2507835	0.8398409	1.2762894
H	4.2923053	1.5048599	2.7595661
H	2.7479576	-1.8771151	1.4094060
H	5.5540521	-1.4790586	0.8971372
H	2.3367098	1.0861345	-1.0733739
H	1.5465091	3.0416924	-2.3216023
H	-0.8917232	3.3306746	-2.7758059
H	5.8219837	-0.7388078	-0.6829509
H	4.7229362	-0.1921903	2.8457954
O	-4.2039710	0.7808406	-3.7628406
H	-4.0528115	0.9799804	-4.7003931
H	-3.8365936	-0.1596380	-3.6732304
C	-3.9579710	-3.9951594	0.8141594
H	-3.3440838	-4.8980513	1.0252655
O	-4.1749710	-3.1131594	1.6431594
H	-6.2415917	-2.9738236	-0.7797540
H	-4.1879146	-0.8298213	2.6888592

4nvh (ΔΔE = –1.9 kcal/mol *vs* Ar-NO)

C	6.3687467	0.4230533	2.9521200
C	5.9807467	0.4220533	1.4931200
O	5.4597467	-0.5649467	0.9371200
H	6.4909115	1.4294443	3.3767577
H	7.3230232	-0.1144298	3.0526782
H	5.6159044	-0.1278816	3.5269104
N	6.2907467	1.5240533	0.7861200
C	6.2267467	1.4970533	-0.6598800
C	4.8007467	1.3250533	-1.1728800
O	3.8087467	1.7330533	-0.5488800
H	4.7330277	0.2282018	-1.3907727
H	6.7861020	2.2825836	1.2422928
H	6.8373538	0.6735013	-1.0573419
H	6.6598975	2.4495616	-0.9998375
H	3.9769074	1.7979149	0.4130215
C	-0.5482533	2.4270533	-1.3918800
C	0.0347467	3.6700533	-0.7618800
O	0.0137467	4.7450533	-1.3498800
H	0.1345929	2.0766593	-2.1787770
H	-0.6980537	1.6026825	-0.6805467
N	0.5597467	3.5100533	0.4441200
C	1.0897467	4.6080533	1.2251200
H	0.1168939	2.7747314	0.9927041
H	1.5808550	4.2044994	2.1189112
H	0.3060810	5.3192310	1.5331394
N	-4.3802533	1.3530533	0.1571200
C	-4.9412533	0.1540533	-0.4328800
C	-4.4612533	-1.0569467	0.3521200
O	-3.4412533	-1.0069467	1.0311200
C	-4.5572533	0.0290533	-1.9108800
C	-3.0672533	-0.1369467	-2.1728800
S	-2.4872533	-1.8369467	-1.9968800
C	-3.2982533	-2.6289467	-3.3818800
H	-3.0336549	-2.1586410	-4.3410476
H	-3.3732923	1.3397279	0.3206778
H	-6.0339478	0.2493371	-0.3424675
H	-4.9117860	0.9462987	-2.4094504
H	-2.4635809	0.4425071	-1.4635125
H	-4.3933677	-2.6456654	-3.2785964
H	-5.1196043	-0.8004091	-2.3662446
H	-2.8051162	0.2268687	-3.1783057
H	-2.9444214	-3.6681197	-3.3967221
N	-5.2272533	-2.1319467	0.2651200
C	-4.8312533	-3.4439467	0.7821200
H	-4.9656995	-3.4973459	1.8723458
H	-6.0814527	-2.0741728	-0.2781778
H	-3.7701206	-3.6152518	0.5518454
H	-5.4439299	-4.2157511	0.3033294
C	2.1867467	-4.0279467	-1.0058800
C	2.8517467	-2.8109467	-1.0868800

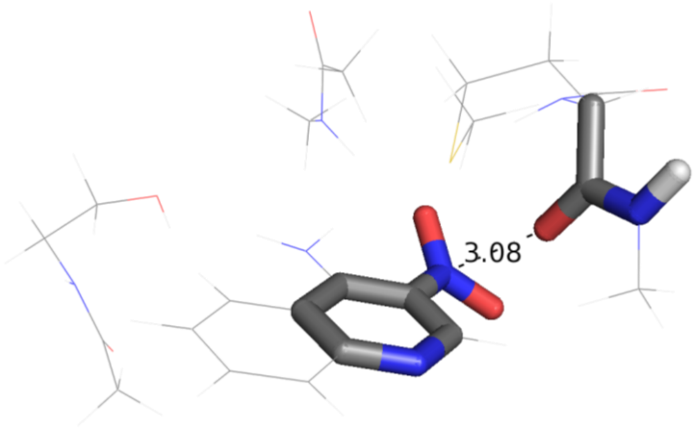

C	1.0347467	-4.1229467	-0.2458800
C	2.3817467	-1.6959467	-0.4108800
C	-1.0892533	-2.0819467	1.8541200
C	0.6977467	-0.7069467	1.0441200
C	-0.4612533	-0.8519467	1.7971200
C	0.5587467	-3.0129467	0.4281200
C	1.2237467	-1.7939467	0.3471200
N	1.3117467	0.4690533	0.9851200
N	-0.5572533	-3.1449467	1.1561200
N	-0.9752533	0.1730533	2.4591200
O	-0.9402533	1.3470533	1.9491200
O	-1.6232533	-0.0789467	3.5361200
H	0.8366188	1.2740536	1.3648911
H	2.5691762	-4.9004030	-1.5376043
H	3.7705931	-2.7275634	-1.6685683
H	0.4785270	-5.0555608	-0.1509266
H	2.9674408	-0.7795219	-0.4741779
H	-2.0047863	-2.2371398	2.4124033
H	2.0699819	0.6379203	0.3310585
H	1.8219671	5.1617528	0.6248771
H	4.8174104	1.7814138	-2.1906299
C	-5.1152533	2.1570533	1.0721200
H	-4.5058140	2.9702363	1.5161864
O	-6.2872533	1.9320533	1.3021200
H	-1.5000562	2.6888853	-1.8692750

Large binding pocket **4nvh**, H-positions optimized, SCS-MP2/def2-SVP

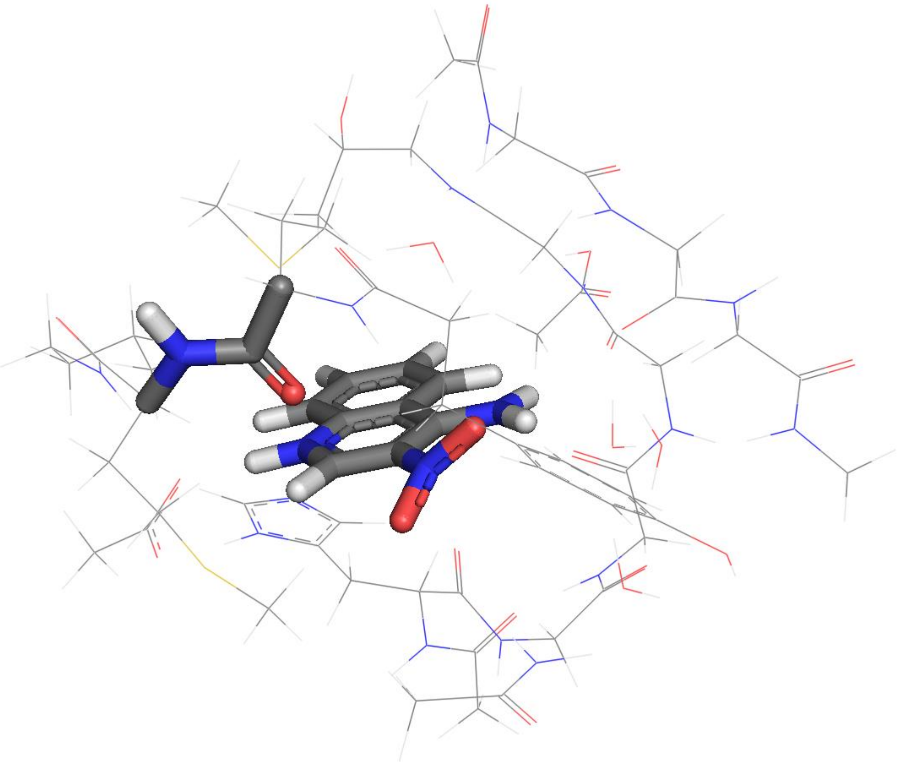

C	-4.066	5.662	5.731
C	-3.389	4.496	5.001
O	-2.538	3.824	5.567
N	-3.767	4.269	3.745
C	-3.164	3.209	2.956
C	-1.777	3.544	2.474
O	-1.164	2.772	1.721
C	-4.078	2.786	1.809
C	-5.125	1.774	2.217
C	-5.362	1.150	3.436
N	-6.053	1.308	1.367
C	-6.844	0.431	2.006
N	-6.418	0.331	3.271
N	-1.242	4.672	2.919
C	0.184	4.937	2.762
C	0.967	4.058	3.741
O	2.165	3.837	3.578
N	0.276	3.563	4.762
C	0.921	2.843	5.875
C	0.911	1.353	5.631
O	-0.083	0.754	5.175
N	2.013	0.674	5.999
C	1.979	-0.771	6.076
C	1.792	-1.420	4.708
O	2.194	-0.897	3.657
N	1.201	-2.612	4.723
C	1.136	-3.407	3.501
C	0.044	-4.431	3.599
O	-0.518	-4.684	4.667
N	-0.257	-5.019	2.448
C	-1.107	-6.176	2.399
C	-1.951	-6.221	1.084
C	-2.705	-4.925	0.840
O	-1.083	-6.428	-0.024
C	-2.151	-2.828	7.901
C	-0.805	-3.534	8.019
O	0.213	-3.085	7.491
O	-0.814	-4.604	8.744
H	-1.821	5.330	3.419
H	0.801	-3.010	5.579
H	0.977	-2.754	2.634
H	-2.987	2.347	3.623
H	0.532	4.693	1.748
H	-4.834	1.253	4.384
H	0.336	3.036	6.786
H	1.942	3.234	6.004
H	-0.734	3.691	4.854
H	-4.544	4.781	3.346
H	-3.431	2.354	1.029
H	-4.540	3.679	1.349

H	1.181	-1.116	6.750
H	2.938	-1.124	6.482
H	2.847	1.167	6.289
H	-7.684	-0.086	1.549
H	-3.288	6.386	6.015
H	-4.842	6.170	5.142
H	-4.505	5.273	6.660
H	-0.667	-7.292	0.065
H	0.179	-4.682	1.588
H	-2.666	-7.060	1.177
H	-2.016	-4.072	0.782
H	-3.264	-4.981	-0.104
H	-3.414	-4.731	1.657
H	-1.763	-6.150	3.281
H	-2.861	-3.471	7.356
H	-2.568	-2.615	8.898
H	-1.703	-4.807	9.056
H	-6.194	1.553	0.351
C	2.288	-6.477	-0.554
C	3.374	-5.902	-1.404
O	4.194	-6.627	-1.989
N	3.435	-4.581	-1.448
C	4.393	-3.823	-2.192
C	5.219	-2.993	-1.219
O	5.260	-3.319	-0.021
N	5.922	-1.996	-1.745
C	6.701	-1.082	-0.936
C	5.869	-0.540	0.191
O	4.702	-0.201	-0.012
N	6.476	-0.386	1.351
C	5.777	-0.065	2.595
C	6.570	0.965	3.384
O	7.781	0.977	3.358
C	6.414	2.792	4.981
H	2.777	-4.057	-0.879
H	2.569	-6.384	0.508
H	1.321	-5.967	-0.682
H	2.191	-7.542	-0.796
H	3.910	-3.191	-2.960
H	5.051	-4.544	-2.703
H	5.758	-1.742	-2.711
H	7.005	-0.234	-1.569
H	7.611	-1.574	-0.556
H	7.454	-0.633	1.459
H	4.771	0.266	2.343
H	6.356	3.797	4.529
H	5.882	2.816	5.945
H	7.473	2.559	5.158
O	2.956	-1.510	-6.375
N	1.743	-0.989	-4.559
C	0.537	-1.625	-5.049

C	-0.667	-0.791	-4.638
O	-0.605	-0.016	-3.689
C	0.409	-3.058	-4.522
C	0.255	-3.173	-3.013
S	-1.439	-2.933	-2.438
C	-2.244	-4.386	-3.104
N	-1.749	-0.946	-5.382
C	-3.055	-0.387	-5.027
C	-4.088	-1.474	-5.116
O	-4.117	-2.236	-6.102
C	-3.417	0.794	-5.971
C	-2.498	1.992	-5.821
S	-2.874	2.914	-4.303
C	-1.476	4.013	-4.284
N	-4.985	-1.548	-4.138
C	-6.138	-2.394	-4.234
C	-6.921	-2.381	-2.918
C	-6.106	-2.887	-1.714
C	-6.871	-2.463	-0.431
H	1.621	-0.375	-3.757
H	-1.813	-5.309	-2.686
H	-3.298	-4.315	-2.801
H	-2.215	-4.395	-4.203
H	-4.463	1.088	-5.790
H	-3.375	0.415	-7.005
H	0.619	-1.671	-6.146
H	1.328	-3.582	-4.834
H	-0.416	-3.574	-5.038
H	0.859	-2.407	-2.500
H	0.616	-4.149	-2.655
H	-1.783	-1.641	-6.123
H	-2.979	-0.009	-3.998
H	-1.481	4.692	-5.151
H	-1.538	4.614	-3.366
H	-0.532	3.450	-4.250
H	-5.810	-3.416	-4.490
H	-4.972	-0.826	-3.395
H	-2.616	2.671	-6.681
H	-1.443	1.673	-5.784
H	-5.103	-2.432	-1.712
H	-7.894	-2.869	-0.431
H	-6.373	-2.807	0.490
H	-6.939	-1.365	-0.392
H	-7.833	-2.987	-3.036
H	-7.253	-1.349	-2.713
C	-6.303	1.827	-3.582
C	-6.046	1.341	-2.206
O	-6.724	1.776	-1.247
O	-5.082	0.506	-2.082
H	-5.482	2.500	-3.877
H	-6.311	0.990	-4.296

H	-7.251	2.376	-3.644
C	-3.584	-1.481	2.136
C	-2.361	-1.503	2.795
C	-3.686	-0.836	0.916
C	-1.247	-0.881	2.252
C	-1.655	1.037	-1.419
C	-0.268	0.398	0.426
C	-0.420	1.035	-0.799
C	-2.577	-0.217	0.368
C	-1.353	-0.239	1.027
N	0.914	0.393	1.033
N	-2.716	0.400	-0.813
N	0.603	1.639	-1.384
O	1.775	1.129	-1.309
O	0.350	2.649	-2.131
H	-2.264	-2.001	3.763
H	1.029	0.030	1.974
H	1.743	0.831	0.612
H	-1.848	1.535	-2.373
H	-0.324	-0.874	2.831
H	-4.471	-1.934	2.582
H	-4.634	-0.789	0.378
H	-3.675	0.448	-1.288
H	-2.027	-1.891	7.346
H	-5.976	-3.981	-1.764
H	-6.782	-2.070	-5.072
H	0.400	5.994	2.969
H	5.687	-0.979	3.208
H	2.097	-3.922	3.320
H	-0.512	-7.108	2.465
C	1.877	6.400	-4.708
C	3.209	6.576	-3.969
N	3.305	6.016	-2.830
O	4.102	7.227	-4.463
H	2.084	5.900	-5.665
H	1.124	5.820	-4.154
H	4.184	6.041	-2.325
H	2.609	5.406	-2.426
C	3.952	-0.060	-4.737
C	2.843	-0.936	-5.293
C	3.599	1.413	-4.898
C	4.614	2.338	-4.302
C	5.518	3.042	-5.113
C	4.646	2.576	-2.908
C	5.576	3.437	-2.326
O	7.410	4.919	-2.543
H	4.872	-0.304	-5.287
H	4.110	-0.273	-3.664
H	2.623	1.605	-4.419
H	3.469	1.631	-5.970
H	5.494	2.907	-6.198

H	3.908	2.091	-2.262
H	5.551	3.645	-1.256
H	7.868	5.424	-3.224
H	1.478	7.397	-4.940
N	5.855	1.783	4.117
H	4.848	1.796	3.922
C	6.488	4.109	-3.145
C	6.487	3.903	-4.549
H	7.193	4.443	-5.186

Large binding pocket **4nvh**$^{\rightarrow Br}$, H-positions optimized, SCS-MP2/def2-SVP

C	-4.057	5.678	5.716
C	-3.380	4.512	4.986
O	-2.529	3.840	5.552
N	-3.758	4.285	3.730
C	-3.155	3.225	2.941
C	-1.768	3.560	2.459
O	-1.155	2.788	1.706
C	-4.069	2.802	1.794
C	-5.116	1.790	2.202
C	-5.353	1.166	3.421
N	-6.044	1.324	1.352
C	-6.835	0.447	1.991
N	-6.409	0.347	3.256
N	-1.233	4.688	2.904
C	0.193	4.953	2.747
C	0.976	4.074	3.726
O	2.174	3.853	3.563
N	0.285	3.579	4.747
C	0.930	2.859	5.860
C	0.920	1.369	5.616
O	-0.074	0.770	5.160
N	2.022	0.690	5.984
C	1.988	-0.755	6.061
C	1.801	-1.404	4.693
O	2.203	-0.881	3.642
N	1.210	-2.596	4.708
C	1.145	-3.391	3.486
C	0.053	-4.415	3.584
O	-0.509	-4.668	4.652
N	-0.248	-5.003	2.433
C	-1.098	-6.160	2.384
C	-1.942	-6.205	1.069
C	-2.696	-4.909	0.825
O	-1.074	-6.412	-0.039
C	-2.142	-2.812	7.886
C	-0.796	-3.518	8.004
O	0.222	-3.069	7.476
O	-0.805	-4.588	8.729
H	-1.812	5.345	3.407
H	0.807	-2.994	5.562
H	0.985	-2.737	2.620
H	-2.979	2.362	3.607
H	0.537	4.702	1.733
H	-4.824	1.267	4.368
H	0.344	3.052	6.771
H	1.950	3.250	5.990
H	-0.726	3.704	4.837
H	-4.539	4.793	3.332
H	-3.423	2.368	1.015
H	-4.532	3.695	1.334

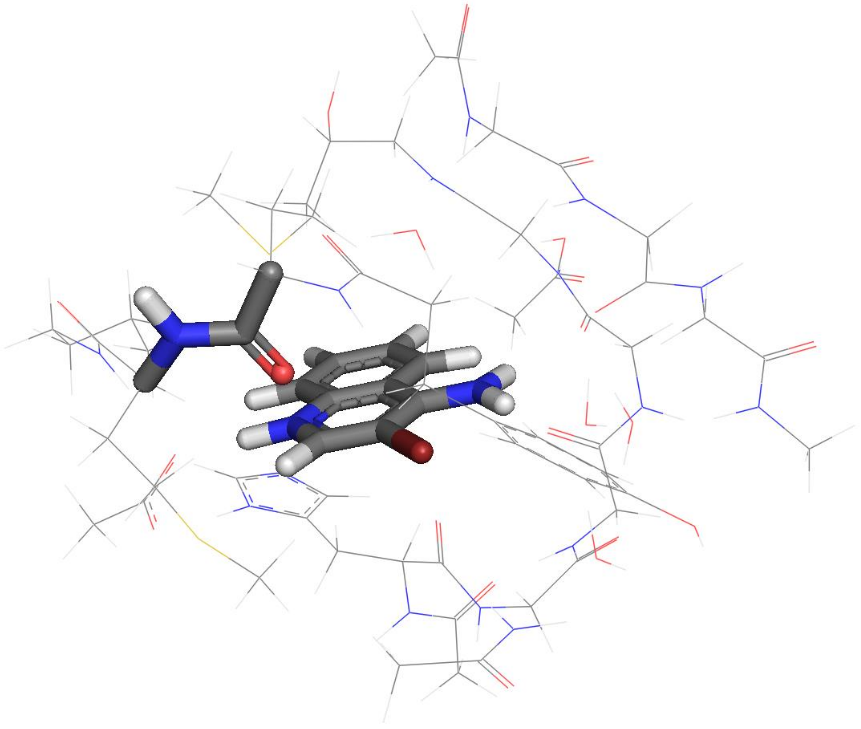

H	1.189	-1.100	6.735
H	2.947	-1.108	6.467
H	2.852	1.183	6.282
H	-7.674	-0.072	1.534
H	-3.279	6.398	6.008
H	-4.827	6.190	5.122
H	-4.506	5.288	6.640
H	-0.657	-7.275	0.051
H	0.181	-4.659	1.572
H	-2.658	-7.044	1.163
H	-2.007	-4.057	0.758
H	-3.263	-4.969	-0.115
H	-3.399	-4.710	1.646
H	-1.755	-6.134	3.265
H	-2.866	-3.468	7.376
H	-2.538	-2.565	8.884
H	-1.695	-4.792	9.038
H	-6.185	1.568	0.336
C	2.297	-6.461	-0.569
C	3.383	-5.886	-1.419
O	4.203	-6.611	-2.004
N	3.444	-4.565	-1.463
C	4.402	-3.807	-2.207
C	5.228	-2.977	-1.234
O	5.269	-3.303	-0.036
N	5.931	-1.980	-1.760
C	6.710	-1.066	-0.951
C	5.878	-0.524	0.176
O	4.711	-0.185	-0.027
N	6.485	-0.370	1.336
C	5.786	-0.049	2.580
C	6.579	0.981	3.369
O	7.790	0.993	3.343
N	5.864	1.799	4.102
C	6.423	2.808	4.966
H	2.782	-4.041	-0.899
H	2.633	-6.479	0.481
H	1.357	-5.889	-0.609
H	2.122	-7.496	-0.890
H	3.918	-3.176	-2.976
H	5.059	-4.530	-2.718
H	5.775	-1.734	-2.729
H	7.013	-0.218	-1.584
H	7.620	-1.558	-0.571
H	7.463	-0.616	1.444
H	4.779	0.282	2.329
H	4.857	1.813	3.905
H	6.451	3.799	4.481
H	5.831	2.892	5.890
H	7.454	2.528	5.221
C	1.886	6.416	-4.723

C	3.218	6.592	-3.984
N	3.314	6.032	-2.845
O	4.111	7.243	-4.478
H	2.089	5.899	-5.672
H	1.126	5.852	-4.162
H	4.189	6.068	-2.334
H	2.604	5.456	-2.415
C	3.961	-0.044	-4.752
C	2.852	-0.920	-5.308
O	2.965	-1.494	-6.390
C	3.608	1.429	-4.913
C	4.623	2.354	-4.317
C	5.527	3.058	-5.128
C	4.655	2.592	-2.923
C	6.496	3.919	-4.564
C	5.585	3.453	-2.341
C	6.497	4.125	-3.160
O	7.419	4.935	-2.558
N	1.752	-0.973	-4.574
C	0.546	-1.609	-5.064
C	-0.658	-0.775	-4.653
O	-0.596	0.000	-3.704
C	0.418	-3.042	-4.537
C	0.264	-3.157	-3.028
S	-1.430	-2.917	-2.453
C	-2.235	-4.370	-3.119
N	-1.740	-0.930	-5.397
C	-3.046	-0.371	-5.042
C	-4.079	-1.458	-5.131
O	-4.108	-2.220	-6.117
C	-3.408	0.810	-5.986
C	-2.489	2.008	-5.836
S	-2.865	2.930	-4.318
C	-1.467	4.029	-4.299
N	-4.976	-1.532	-4.153
C	-6.129	-2.378	-4.249
C	-6.912	-2.365	-2.933
C	-6.097	-2.871	-1.729
C	-6.862	-2.447	-0.446
H	4.880	-0.286	-5.303
H	4.120	-0.260	-3.680
H	2.626	1.617	-4.445
H	3.482	1.647	-5.986
H	5.503	2.921	-6.213
H	3.949	2.078	-2.263
H	1.616	-0.351	-3.779
H	7.202	4.457	-5.201
H	5.568	3.654	-1.269
H	-1.802	-5.293	-2.705
H	-3.289	-4.302	-2.812
H	-2.210	-4.379	-4.218

H	-4.454	1.104	-5.805
H	-3.366	0.432	-7.021
H	7.884	5.434	-3.238
H	0.628	-1.655	-6.162
H	1.335	-3.568	-4.851
H	-0.410	-3.556	-5.052
H	0.866	-2.393	-2.512
H	0.624	-4.134	-2.671
H	-1.777	-1.627	-6.137
H	-2.970	0.006	-4.013
H	-1.433	4.663	-5.199
H	-1.568	4.682	-3.420
H	-0.522	3.470	-4.207
H	-5.802	-3.400	-4.505
H	-4.963	-0.811	-3.409
H	-2.604	2.688	-6.695
H	-1.436	1.685	-5.797
H	-5.095	-2.414	-1.726
H	-7.883	-2.859	-0.441
H	-6.359	-2.784	0.474
H	-6.936	-1.350	-0.411
H	-7.825	-2.970	-3.052
H	-7.243	-1.333	-2.728
C	-6.294	1.843	-3.597
C	-6.037	1.357	-2.221
O	-6.715	1.792	-1.262
O	-5.073	0.522	-2.097
H	-5.477	2.518	-3.892
H	-6.300	1.006	-4.311
H	-7.244	2.389	-3.661
C	-3.575	-1.465	2.121
C	-2.352	-1.487	2.780
C	-3.677	-0.820	0.901
C	-1.238	-0.865	2.237
C	-1.646	1.053	-1.434
C	-0.259	0.414	0.411
C	-0.411	1.051	-0.814
C	-2.568	-0.201	0.353
C	-1.344	-0.223	1.012
N	0.923	0.409	1.018
N	-2.707	0.416	-0.828
H	-2.257	-1.984	3.749
H	1.060	0.015	1.942
H	1.722	0.927	0.640
H	-1.856	1.544	-2.386
H	-0.317	-0.850	2.818
H	-4.463	-1.919	2.567
H	-4.625	-0.774	0.363
H	-3.663	0.468	-1.293
O	3.350	1.577	3.069
H	2.871	0.773	3.373

H	2.813	2.368	3.300
O	3.707	4.691	-0.022
H	3.694	3.709	0.014
H	3.782	4.930	0.908
O	3.758	1.904	0.419
H	3.672	1.846	1.386
H	4.172	1.059	0.160
O	0.565	-3.216	0.191
H	0.769	-2.280	0.313
H	-0.210	-3.217	-0.404
Br	1.039	1.923	-1.662
H	1.494	7.412	-4.970
H	-2.025	-1.894	7.299
H	-5.967	-3.965	-1.779
H	-6.773	-2.055	-5.088
H	0.410	6.010	2.953
H	2.106	-3.906	3.305
H	5.695	-0.964	3.192
H	-0.504	-7.092	2.451

Large binding pocket **4nvi**, H-positions optimized, SCS-MP2/def2-SVP

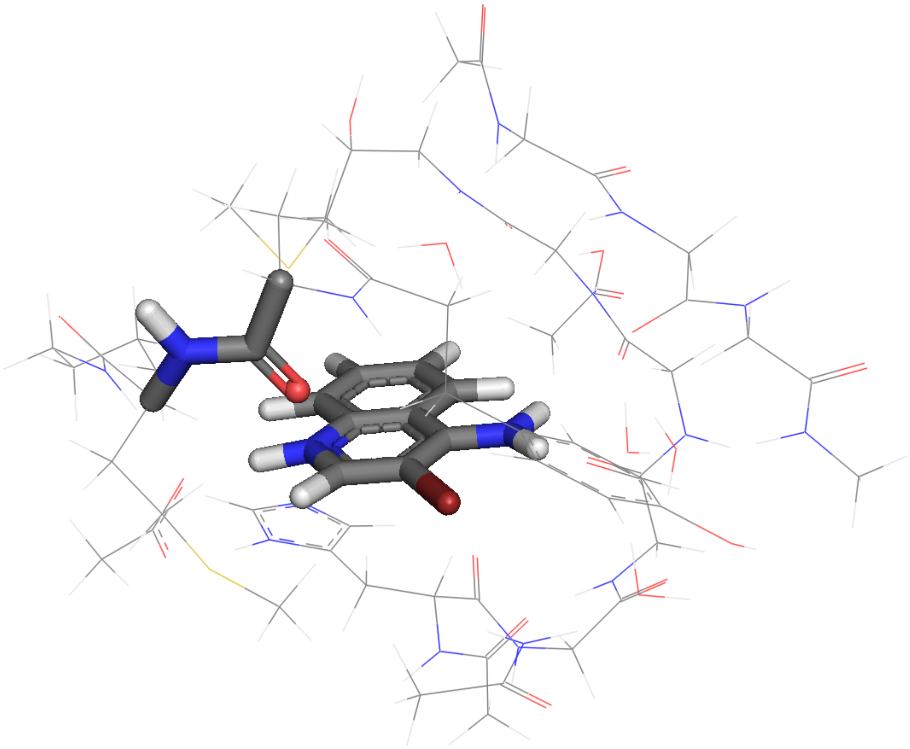

C -4.090 5.692 5.792
C -3.379 4.547 5.084
O -2.561 3.862 5.678
N -3.695 4.342 3.811
C -3.076 3.272 3.060
C -1.689 3.635 2.630
O -1.044 2.894 1.866
C -3.931 2.862 1.869
C -5.003 1.862 2.219
C -5.280 1.202 3.412
N -5.918 1.439 1.331
C -6.739 0.556 1.923
N -6.347 0.413 3.196
N -1.199 4.763 3.123
C 0.206 5.086 2.975
C 0.980 4.213 3.950
O 2.193 4.073 3.839
N 0.261 3.628 4.903
C 0.879 2.901 6.007
C 0.907 1.435 5.754
O -0.078 0.863 5.330
N 2.010 0.770 6.095
C 1.946 -0.661 6.095
C 1.791 -1.267 4.704
O 2.175 -0.690 3.664
N 1.190 -2.455 4.695
C 1.165 -3.307 3.507
C 0.023 -4.292 3.566
O -0.582 -4.518 4.617
N -0.237 -4.904 2.411
C -1.082 -6.066 2.338
C -1.897 -6.122 1.014
O -1.022 -6.307 -0.083
C -2.077 -2.784 7.982
C -0.747 -3.499 8.052
O 0.261 -3.028 7.493
O -0.734 -4.573 8.736
H 1.113 -1.023 6.732
H 2.883 -1.065 6.531
H -7.579 0.064 1.422
H -3.327 6.415 6.144
H -4.837 6.227 5.173
H -4.588 5.287 6.694
H 0.803 -2.858 5.567
H -0.764 3.726 4.970
H -4.474 4.844 3.382
H 0.260 3.065 6.913
H 1.890 3.326 6.186
H -4.772 1.271 4.383
H 0.576 4.860 1.955

H	-2.923	2.404	3.739
H	-3.240	2.421	1.117
H	-4.355	3.769	1.379
H	-1.827	5.414	3.591
H	1.099	-2.673	2.602
H	2.872	1.253	6.348
H	-0.582	-7.173	0.005
H	-2.617	-6.972	1.100
H	0.219	-4.558	1.549
H	-1.760	-6.050	3.214
H	-2.762	-3.346	7.312
H	-2.561	-2.705	8.978
H	-1.619	-4.788	9.087
H	-6.034	1.665	0.295
C	2.231	-6.382	-0.649
C	3.326	-5.839	-1.538
O	4.035	-6.574	-2.221
N	3.449	-4.522	-1.496
C	4.408	-3.754	-2.230
C	5.224	-2.927	-1.246
O	5.260	-3.261	-0.050
N	5.927	-1.923	-1.762
C	6.696	-1.011	-0.941
C	5.856	-0.480	0.185
O	4.688	-0.146	-0.023
N	6.454	-0.332	1.349
C	5.746	-0.021	2.592
C	6.529	1.006	3.392
O	7.741	1.024	3.373
N	5.807	1.816	4.127
C	6.356	2.822	5.001
H	4.727	0.291	2.336
H	6.196	3.847	4.601
H	5.890	2.775	6.008
H	7.446	2.656	5.101
H	2.062	-7.442	-0.912
H	2.544	-6.327	0.415
H	1.283	-5.814	-0.737
H	7.004	-0.154	-1.575
H	7.616	-1.505	-0.559
H	5.788	-1.674	-2.744
H	7.444	-0.569	1.467
H	2.829	-3.999	-0.867
H	4.785	1.813	3.939
H	3.925	-3.116	-3.008
H	5.070	-4.476	-2.758
C	1.733	6.201	-4.580
C	2.892	6.235	-3.663
N	2.741	5.486	-2.577
O	3.947	6.843	-3.938
H	1.797	5.298	-5.224

H	0.752	6.167	-4.062
H	3.451	5.406	-1.831
H	1.836	5.080	-2.339
C	4.063	-0.260	-5.147
C	2.864	-1.046	-5.651
O	2.879	-1.615	-6.764
C	3.698	1.224	-5.130
C	4.756	2.157	-4.587
C	5.523	2.913	-5.446
C	4.930	2.309	-3.218
C	6.506	3.780	-4.973
C	5.905	3.173	-2.717
C	6.686	3.903	-3.608
O	7.662	4.767	-3.154
N	1.821	-1.087	-4.839
C	0.563	-1.686	-5.269
C	-0.584	-0.798	-4.857
O	-0.466	-0.022	-3.927
C	0.336	-3.125	-4.722
C	0.253	-3.267	-3.218
S	-1.335	-2.872	-2.484
C	-2.218	-4.372	-2.931
N	-1.705	-0.961	-5.533
C	-3.001	-0.418	-5.111
C	-4.039	-1.513	-5.185
O	-4.107	-2.250	-6.158
C	-3.436	0.755	-6.002
C	-2.502	1.979	-5.869
S	-2.870	2.896	-4.341
C	-1.403	3.940	-4.237
N	-4.912	-1.572	-4.215
C	-6.162	-2.382	-4.280
C	-6.922	-2.302	-2.936
C	-6.109	-2.816	-1.772
C	-6.845	-2.452	-0.487
H	4.913	-0.462	-5.825
H	4.338	-0.586	-4.118
H	5.368	2.827	-6.534
H	5.982	3.330	-1.628
H	2.773	1.361	-4.528
H	3.428	1.525	-6.164
H	1.738	-0.466	-4.026
H	7.587	4.823	-2.184
H	0.627	-1.771	-6.375
H	4.299	1.753	-2.504
H	7.117	4.392	-5.652
H	-1.823	-5.249	-2.378
H	-3.279	-4.215	-2.657
H	-2.180	-4.543	-4.026
H	-1.311	4.617	-5.112
H	-1.502	4.557	-3.323

H	-0.484	3.324	-4.144
H	1.192	-3.731	-5.092
H	-0.565	-3.553	-5.211
H	-1.786	-1.669	-6.273
H	-4.479	1.039	-5.753
H	-3.447	0.405	-7.056
H	-4.898	-0.833	-3.470
H	-5.102	-2.348	-1.771
H	0.996	-2.601	-2.728
H	0.536	-4.295	-2.906
H	-2.878	-0.057	-4.070
H	-5.881	-3.428	-4.526
H	-7.878	-2.858	-0.479
H	-6.337	-2.828	0.426
H	-6.915	-1.349	-0.396
H	-2.629	2.660	-6.737
H	-1.442	1.651	-5.842
H	-7.870	-2.871	-3.035
H	-7.207	-1.244	-2.751
C	-6.277	1.921	-3.557
C	-6.029	1.325	-2.218
O	-6.639	1.746	-1.244
O	-5.097	0.460	-2.119
H	-5.469	2.650	-3.774
H	-6.237	1.154	-4.358
H	-7.247	2.449	-3.595
C	-3.477	-1.625	2.108
C	-2.286	-1.495	2.810
C	-3.623	-0.990	0.884
C	-1.244	-0.738	2.293
C	-1.729	1.145	-1.378
C	-0.530	1.291	-0.705
C	-0.377	0.654	0.519
C	-2.583	-0.234	0.364
C	-1.395	-0.108	1.068
N	0.764	0.777	1.186
N	-2.739	0.375	-0.818
Br	0.819	2.341	-1.471
H	-4.568	-1.043	0.326
H	-1.957	1.598	-2.359
H	-2.152	-1.991	3.783
H	-0.344	-0.618	2.909
H	0.932	0.325	2.086
H	1.570	1.303	0.807
H	-4.322	-2.190	2.527
H	-3.704	0.379	-1.309
O	3.377	1.621	3.136
H	2.858	0.815	3.447
H	2.861	2.434	3.394
O	3.743	4.759	0.070
H	4.503	5.002	0.628

H	3.752	3.759	0.114
O	3.809	1.970	0.492
H	4.113	1.069	0.214
H	3.712	1.899	1.470
O	0.655	-3.171	0.197
H	0.733	-2.213	0.379
H	-0.085	-3.208	-0.466
H	1.758	7.084	-5.246
H	-1.941	-1.774	7.557
H	-0.475	-7.000	2.423
H	0.392	6.154	3.199
H	-6.787	-2.021	-5.126
H	2.120	-3.873	3.414
H	-5.953	-3.915	-1.859
H	5.663	-0.949	3.202
C	-2.666	-4.817	0.812
H	-3.348	-4.634	1.665
H	-1.975	-3.955	0.743
H	-3.267	-4.851	-0.117

Large binding pocket **4nvi**$^{\rightarrow NO_2}$, H-positions optimized, SCS-MP2/def2-SVP

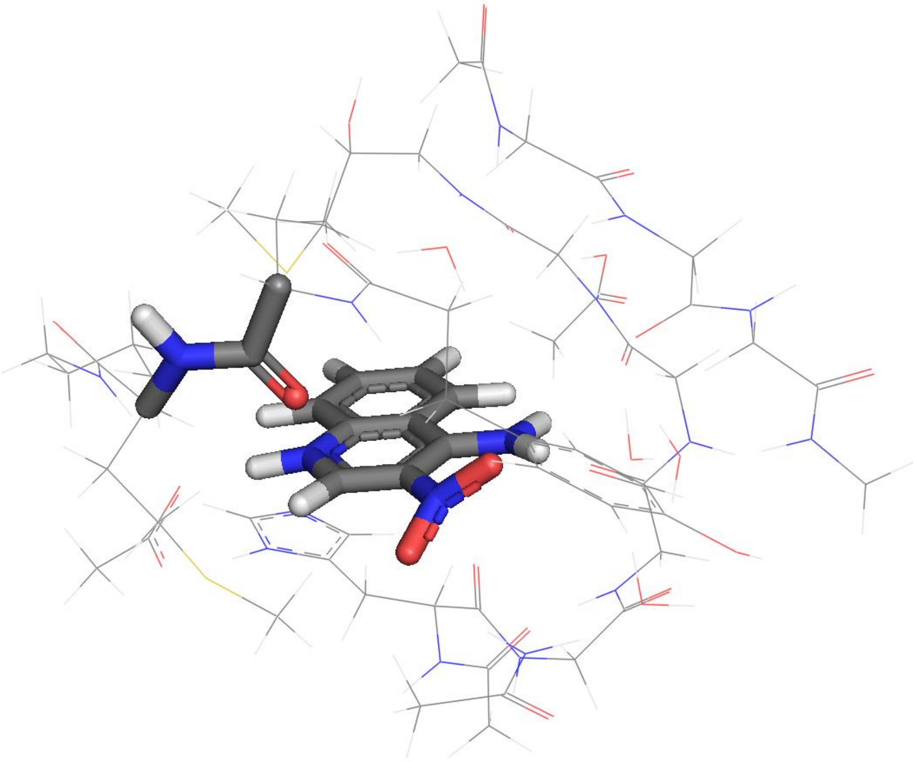

C	-4.096	5.673	5.806
C	-3.385	4.528	5.098
O	-2.567	3.843	5.692
N	-3.701	4.323	3.825
C	-3.082	3.253	3.074
C	-1.695	3.616	2.644
O	-1.050	2.875	1.880
C	-3.937	2.843	1.883
C	-5.009	1.843	2.233
C	-5.286	1.183	3.426
N	-5.924	1.420	1.345
C	-6.745	0.537	1.937
N	-6.353	0.394	3.210
N	-1.205	4.744	3.137
C	0.200	5.067	2.989
C	0.974	4.194	3.964
O	2.187	4.054	3.853
N	0.255	3.609	4.917
C	0.873	2.882	6.021
C	0.901	1.416	5.768
O	-0.084	0.844	5.344
N	2.004	0.751	6.109
C	1.940	-0.680	6.109
C	1.785	-1.286	4.718
O	2.169	-0.709	3.678
N	1.184	-2.474	4.709
C	1.159	-3.326	3.521
C	0.017	-4.311	3.580
O	-0.588	-4.537	4.631
N	-0.243	-4.923	2.425
C	-1.088	-6.085	2.352
C	-1.903	-6.138	1.031
C	-2.671	-4.834	0.832
O	-1.028	-6.321	-0.066
C	-2.083	-2.803	7.996
C	-0.753	-3.518	8.066
O	0.255	-3.047	7.507
O	-0.740	-4.592	8.750
H	1.107	-1.042	6.746
H	2.876	-1.085	6.545
H	-7.586	0.045	1.437
H	-3.333	6.394	6.159
H	-4.842	6.208	5.187
H	-4.596	5.267	6.707
H	0.799	-2.879	5.582
H	-0.770	3.709	4.986
H	-4.478	4.828	3.395
H	0.254	3.046	6.927
H	1.883	3.306	6.200
H	-4.780	1.253	4.397

H	0.571	4.847	1.968
H	-2.929	2.385	3.754
H	-3.247	2.403	1.130
H	-4.362	3.750	1.393
H	-1.834	5.394	3.605
H	1.092	-2.693	2.615
H	2.867	1.233	6.359
H	-0.589	-7.188	0.018
H	-2.624	-6.988	1.115
H	-1.980	-3.972	0.765
H	-3.272	-4.865	-0.098
H	-3.353	-4.655	1.685
H	0.219	-4.584	1.565
H	-1.765	-6.070	3.230
H	-2.801	-3.404	7.397
H	-2.522	-2.655	9.005
H	-1.625	-4.808	9.101
H	-6.043	1.647	0.309
C	2.225	-6.401	-0.635
C	3.320	-5.858	-1.524
O	4.029	-6.593	-2.207
N	3.443	-4.541	-1.482
C	4.402	-3.773	-2.216
C	5.218	-2.946	-1.232
O	5.254	-3.280	-0.036
N	5.921	-1.942	-1.748
C	6.690	-1.030	-0.927
C	5.850	-0.499	0.199
O	4.682	-0.165	-0.009
N	6.448	-0.351	1.363
C	5.740	-0.040	2.606
C	6.523	0.987	3.406
O	7.735	1.005	3.387
N	5.801	1.797	4.141
C	6.350	2.803	5.015
H	4.721	0.272	2.347
H	6.272	3.822	4.576
H	5.819	2.812	5.990
H	7.422	2.587	5.187
H	2.093	-7.475	-0.859
H	2.509	-6.293	0.433
H	1.263	-5.864	-0.767
H	6.997	-0.173	-1.561
H	7.609	-1.525	-0.545
H	5.776	-1.690	-2.728
H	7.437	-0.589	1.481
H	2.825	-4.020	-0.850
H	4.780	1.798	3.949
H	3.919	-3.135	-2.994
H	5.063	-4.495	-2.744
C	1.727	6.182	-4.566

C	2.886	6.216	-3.649
N	2.735	5.467	-2.563
O	3.941	6.824	-3.924
H	1.805	5.294	-5.229
H	0.749	6.119	-4.046
H	3.462	5.366	-1.839
H	1.900	4.897	-2.406
C	4.057	-0.279	-5.133
C	2.858	-1.065	-5.637
O	2.873	-1.634	-6.750
C	3.692	1.205	-5.116
C	4.750	2.138	-4.573
C	5.517	2.894	-5.432
C	4.924	2.290	-3.204
C	6.500	3.761	-4.959
C	5.899	3.154	-2.703
C	6.680	3.884	-3.594
O	7.656	4.748	-3.140
N	1.815	-1.106	-4.825
C	0.557	-1.705	-5.255
C	-0.590	-0.817	-4.843
O	-0.472	-0.041	-3.913
C	0.330	-3.144	-4.708
C	0.247	-3.286	-3.204
S	-1.341	-2.891	-2.470
C	-2.224	-4.391	-2.917
N	-1.711	-0.980	-5.519
C	-3.007	-0.437	-5.097
C	-4.045	-1.532	-5.171
O	-4.113	-2.269	-6.144
C	-3.442	0.736	-5.988
C	-2.508	1.960	-5.855
S	-2.876	2.877	-4.327
C	-1.409	3.921	-4.223
N	-4.918	-1.591	-4.201
C	-6.168	-2.401	-4.266
C	-6.928	-2.321	-2.922
C	-6.115	-2.835	-1.758
C	-6.851	-2.471	-0.473
H	4.907	-0.482	-5.810
H	4.331	-0.605	-4.103
H	5.360	2.809	-6.520
H	5.970	3.315	-1.615
H	2.768	1.344	-4.512
H	3.421	1.506	-6.150
H	1.736	-0.486	-4.011
H	7.575	4.809	-2.171
H	0.622	-1.790	-6.361
H	4.278	1.749	-2.492
H	7.110	4.374	-5.637
H	-1.830	-5.267	-2.362

H	-3.287	-4.234	-2.646
H	-2.183	-4.562	-4.011
H	-1.359	4.637	-5.070
H	-1.470	4.483	-3.274
H	-0.490	3.304	-4.193
H	1.186	-3.750	-5.077
H	-0.571	-3.573	-5.197
H	-1.791	-1.686	-6.262
H	-4.485	1.020	-5.739
H	-3.455	0.384	-7.041
H	-4.905	-0.852	-3.459
H	-5.109	-2.367	-1.758
H	0.990	-2.620	-2.716
H	0.529	-4.314	-2.890
H	-2.885	-0.074	-4.057
H	-5.888	-3.448	-4.512
H	-7.883	-2.880	-0.464
H	-6.343	-2.846	0.440
H	-6.925	-1.368	-0.383
H	-2.636	2.639	-6.724
H	-1.448	1.634	-5.828
H	-7.877	-2.891	-3.021
H	-7.214	-1.263	-2.737
C	-6.283	1.902	-3.543
C	-6.035	1.306	-2.204
O	-6.645	1.727	-1.230
O	-5.103	0.441	-2.105
H	-5.473	2.627	-3.761
H	-6.246	1.134	-4.344
H	-7.253	2.432	-3.579
C	-3.483	-1.644	2.122
C	-2.292	-1.514	2.824
C	-3.629	-1.009	0.898
C	-1.250	-0.757	2.307
C	-1.735	1.126	-1.364
C	-0.536	1.272	-0.691
C	-0.383	0.635	0.533
C	-2.589	-0.253	0.378
C	-1.401	-0.127	1.082
N	0.758	0.758	1.200
N	-2.745	0.356	-0.804
H	-4.574	-1.063	0.339
H	-1.948	1.588	-2.345
H	-2.157	-2.014	3.795
H	-0.346	-0.649	2.918
H	0.884	0.364	2.135
H	1.589	1.208	0.774
H	-4.328	-2.210	2.540
H	-3.715	0.356	-1.306
O	3.371	1.602	3.150
H	2.853	0.798	3.466

H	2.856	2.417	3.400
O	3.737	4.740	0.084
H	4.535	4.937	0.606
H	3.714	3.740	0.104
O	3.803	1.951	0.506
H	4.068	1.053	0.189
H	3.726	1.854	1.482
O	0.649	-3.190	0.211
H	0.822	-2.236	0.329
H	-0.093	-3.206	-0.451
N	0.441	2.001	-1.209
O	1.658	1.630	-1.070
O	0.114	2.988	-1.958
H	1.737	7.078	-5.215
H	-1.961	-1.823	7.503
H	-5.959	-3.934	-1.845
H	-6.794	-2.040	-5.112
H	0.384	6.134	3.215
H	5.656	-0.968	3.216
H	2.114	-3.893	3.428
H	-0.481	-7.020	2.436

Large binding pocket **4nvh**, H-positions optimized, SCS-MP2/def2-SVP (waters omitted)

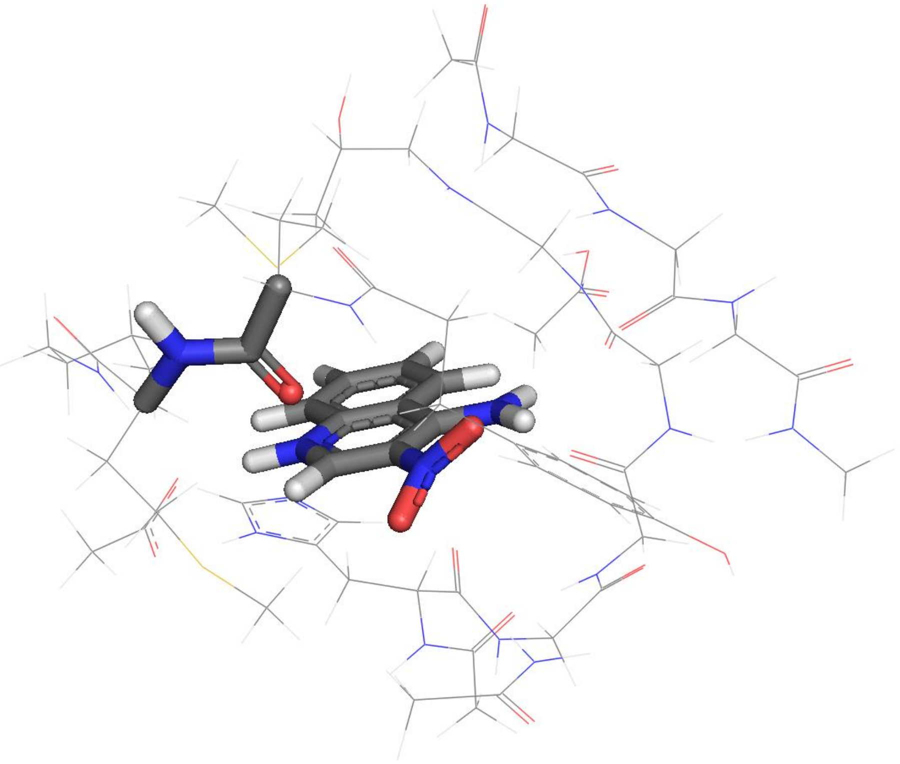

C	-4.066	5.662	5.731
C	-3.389	4.496	5.001
O	-2.538	3.824	5.567
N	-3.767	4.269	3.745
C	-3.164	3.209	2.956
C	-1.777	3.544	2.474
O	-1.164	2.772	1.721
C	-4.078	2.786	1.809
C	-5.125	1.774	2.217
C	-5.362	1.150	3.436
N	-6.053	1.308	1.367
C	-6.844	0.431	2.006
N	-6.418	0.331	3.271
N	-1.242	4.672	2.919
C	0.184	4.937	2.762
C	0.967	4.058	3.741
O	2.165	3.837	3.578
N	0.276	3.563	4.762
C	0.921	2.843	5.875
C	0.911	1.353	5.631
O	-0.083	0.754	5.175
N	2.013	0.674	5.999
C	1.979	-0.771	6.076
C	1.792	-1.420	4.708
O	2.194	-0.897	3.657
N	1.201	-2.612	4.723
C	1.136	-3.407	3.501
C	0.044	-4.431	3.599
O	-0.518	-4.684	4.667
N	-0.257	-5.019	2.448
C	-1.107	-6.176	2.399
C	-1.951	-6.221	1.084
C	-2.705	-4.925	0.840
O	-1.083	-6.428	-0.024
C	-2.151	-2.828	7.901
C	-0.805	-3.534	8.019
O	0.213	-3.085	7.491
O	-0.814	-4.604	8.744
H	-1.821	5.330	3.419
H	0.801	-3.010	5.579
H	0.977	-2.754	2.634
H	-2.987	2.347	3.623
H	0.532	4.693	1.748
H	-4.834	1.253	4.384
H	0.336	3.036	6.786
H	1.942	3.234	6.004
H	-0.734	3.691	4.854
H	-4.544	4.781	3.346
H	-3.431	2.354	1.029
H	-4.540	3.679	1.349

H	1.181	-1.116	6.750
H	2.938	-1.124	6.482
H	2.847	1.167	6.289
H	-7.684	-0.086	1.549
H	-3.288	6.386	6.015
H	-4.842	6.170	5.142
H	-4.505	5.273	6.660
H	-0.667	-7.292	0.065
H	0.179	-4.682	1.588
H	-2.666	-7.060	1.177
H	-2.016	-4.072	0.782
H	-3.264	-4.981	-0.104
H	-3.414	-4.731	1.657
H	-1.763	-6.150	3.281
H	-2.861	-3.471	7.356
H	-2.568	-2.615	8.898
H	-1.703	-4.807	9.056
H	-6.194	1.553	0.351
C	2.288	-6.477	-0.554
C	3.374	-5.902	-1.404
O	4.194	-6.627	-1.989
N	3.435	-4.581	-1.448
C	4.393	-3.823	-2.192
C	5.219	-2.993	-1.219
O	5.260	-3.319	-0.021
N	5.922	-1.996	-1.745
C	6.701	-1.082	-0.936
C	5.869	-0.540	0.191
O	4.702	-0.201	-0.012
N	6.476	-0.386	1.351
C	5.777	-0.065	2.595
C	6.570	0.965	3.384
O	7.781	0.977	3.358
C	6.414	2.792	4.981
H	2.777	-4.057	-0.879
H	2.569	-6.384	0.508
H	1.321	-5.967	-0.682
H	2.191	-7.542	-0.796
H	3.910	-3.191	-2.960
H	5.051	-4.544	-2.703
H	5.758	-1.742	-2.711
H	7.005	-0.234	-1.569
H	7.611	-1.574	-0.556
H	7.454	-0.633	1.459
H	4.771	0.266	2.343
H	6.356	3.797	4.529
H	5.882	2.816	5.945
H	7.473	2.559	5.158
O	2.956	-1.510	-6.375
N	1.743	-0.989	-4.559
C	0.537	-1.625	-5.049

C	-0.667	-0.791	-4.638
O	-0.605	-0.016	-3.689
C	0.409	-3.058	-4.522
C	0.255	-3.173	-3.013
S	-1.439	-2.933	-2.438
C	-2.244	-4.386	-3.104
N	-1.749	-0.946	-5.382
C	-3.055	-0.387	-5.027
C	-4.088	-1.474	-5.116
O	-4.117	-2.236	-6.102
C	-3.417	0.794	-5.971
C	-2.498	1.992	-5.821
S	-2.874	2.914	-4.303
C	-1.476	4.013	-4.284
N	-4.985	-1.548	-4.138
C	-6.138	-2.394	-4.234
C	-6.921	-2.381	-2.918
C	-6.106	-2.887	-1.714
C	-6.871	-2.463	-0.431
H	1.621	-0.375	-3.757
H	-1.813	-5.309	-2.686
H	-3.298	-4.315	-2.801
H	-2.215	-4.395	-4.203
H	-4.463	1.088	-5.790
H	-3.375	0.415	-7.005
H	0.619	-1.671	-6.146
H	1.328	-3.582	-4.834
H	-0.416	-3.574	-5.038
H	0.859	-2.407	-2.500
H	0.616	-4.149	-2.655
H	-1.783	-1.641	-6.123
H	-2.979	-0.009	-3.998
H	-1.481	4.692	-5.151
H	-1.538	4.614	-3.366
H	-0.532	3.450	-4.250
H	-5.810	-3.416	-4.490
H	-4.972	-0.826	-3.395
H	-2.616	2.671	-6.681
H	-1.443	1.673	-5.784
H	-5.103	-2.432	-1.712
H	-7.894	-2.869	-0.431
H	-6.373	-2.807	0.490
H	-6.939	-1.365	-0.392
H	-7.833	-2.987	-3.036
H	-7.253	-1.349	-2.713
C	-6.303	1.827	-3.582
C	-6.046	1.341	-2.206
O	-6.724	1.776	-1.247
O	-5.082	0.506	-2.082
H	-5.482	2.500	-3.877
H	-6.311	0.990	-4.296

H	-7.251	2.376	-3.644
C	-3.584	-1.481	2.136
C	-2.361	-1.503	2.795
C	-3.686	-0.836	0.916
C	-1.247	-0.881	2.252
C	-1.655	1.037	-1.419
C	-0.268	0.398	0.426
C	-0.420	1.035	-0.799
C	-2.577	-0.217	0.368
C	-1.353	-0.239	1.027
N	0.914	0.393	1.033
N	-2.716	0.400	-0.813
N	0.603	1.639	-1.384
O	1.775	1.129	-1.309
O	0.350	2.649	-2.131
H	-2.264	-2.001	3.763
H	1.029	0.030	1.974
H	1.743	0.831	0.612
H	-1.848	1.535	-2.373
H	-0.324	-0.874	2.831
H	-4.471	-1.934	2.582
H	-4.634	-0.789	0.378
H	-3.675	0.448	-1.288
H	-2.027	-1.891	7.346
H	-5.976	-3.981	-1.764
H	-6.782	-2.070	-5.072
H	0.400	5.994	2.969
H	5.687	-0.979	3.208
H	2.097	-3.922	3.320
H	-0.512	-7.108	2.465
O	3.341	1.561	3.084
H	2.863	0.761	3.396
H	2.805	2.357	3.298
O	3.698	4.675	-0.007
H	3.667	3.693	0.017
H	3.922	4.895	0.903
O	3.749	1.888	0.434
H	3.710	1.792	1.399
H	4.134	1.055	0.112
O	0.556	-3.232	0.206
H	0.825	-2.307	0.273
H	-0.214	-3.222	-0.395
C	1.877	6.400	-4.708
C	3.209	6.576	-3.969
N	3.305	6.016	-2.830
O	4.102	7.227	-4.463
H	2.084	5.900	-5.665
H	1.124	5.820	-4.154
H	4.184	6.041	-2.325
H	2.609	5.406	-2.426
C	3.952	-0.060	-4.737

C	2.843	-0.936	-5.293
C	3.599	1.413	-4.898
C	4.614	2.338	-4.302
C	5.518	3.042	-5.113
C	4.646	2.576	-2.908
C	5.576	3.437	-2.326
O	7.410	4.919	-2.543
H	4.872	-0.304	-5.287
H	4.110	-0.273	-3.664
H	2.623	1.605	-4.419
H	3.469	1.631	-5.970
H	5.494	2.907	-6.198
H	3.908	2.091	-2.262
H	5.551	3.645	-1.256
H	7.868	5.424	-3.224
H	1.478	7.397	-4.940
N	5.855	1.783	4.117
H	4.848	1.796	3.922
C	6.488	4.109	-3.145
C	6.487	3.903	-4.549
H	7.193	4.443	-5.186

Large binding pocket **4nvh**$^{\rightarrow Br}$, H-positions optimized, SCS-MP2/def2-SVP (waters omitted)

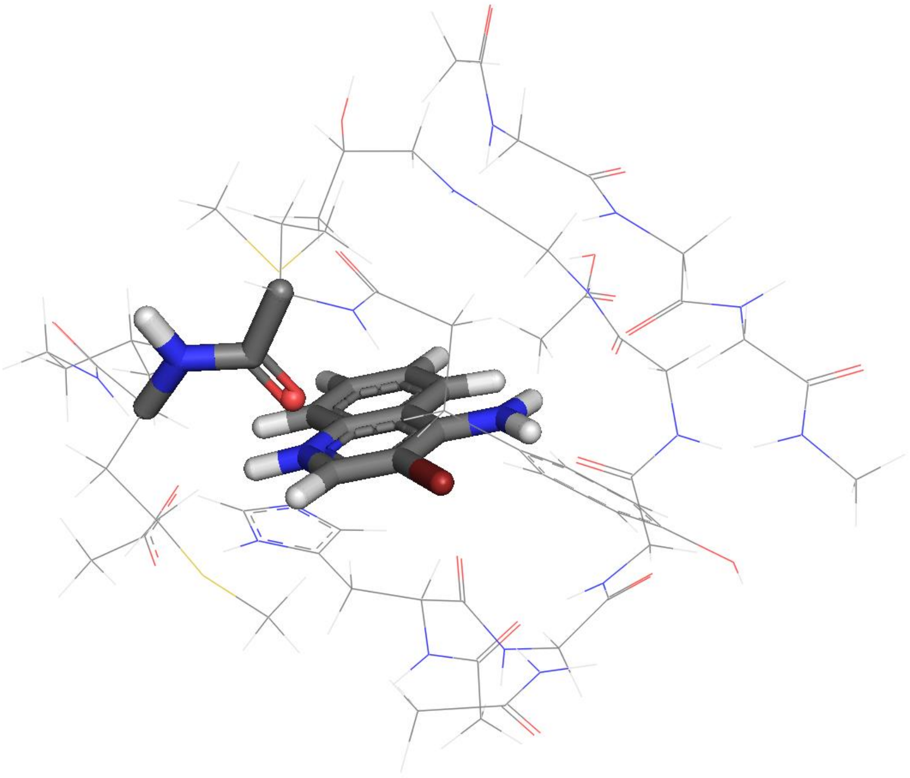

C	-4.057	5.678	5.716
C	-3.380	4.512	4.986
O	-2.529	3.840	5.552
N	-3.758	4.285	3.730
C	-3.155	3.225	2.941
C	-1.768	3.560	2.459
O	-1.155	2.788	1.706
C	-4.069	2.802	1.794
C	-5.116	1.790	2.202
C	-5.353	1.166	3.421
N	-6.044	1.324	1.352
C	-6.835	0.447	1.991
N	-6.409	0.347	3.256
N	-1.233	4.688	2.904
C	0.193	4.953	2.747
C	0.976	4.074	3.726
O	2.174	3.853	3.563
N	0.285	3.579	4.747
C	0.930	2.859	5.860
C	0.920	1.369	5.616
O	-0.074	0.770	5.160
N	2.022	0.690	5.984
C	1.988	-0.755	6.061
C	1.801	-1.404	4.693
O	2.203	-0.881	3.642
N	1.210	-2.596	4.708
C	1.145	-3.391	3.486
C	0.053	-4.415	3.584
O	-0.509	-4.668	4.652
N	-0.248	-5.003	2.433
C	-1.098	-6.160	2.384
C	-1.942	-6.205	1.069
C	-2.696	-4.909	0.825
O	-1.074	-6.412	-0.039
C	-2.142	-2.812	7.886
C	-0.796	-3.518	8.004
O	0.222	-3.069	7.476
O	-0.805	-4.588	8.729
H	-1.812	5.345	3.407
H	0.807	-2.994	5.562
H	0.985	-2.737	2.620
H	-2.979	2.362	3.607
H	0.537	4.702	1.733
H	-4.824	1.267	4.368
H	0.344	3.052	6.771
H	1.950	3.250	5.990
H	-0.726	3.704	4.837
H	-4.539	4.793	3.332
H	-3.423	2.368	1.015
H	-4.532	3.695	1.334

H	1.189	-1.100	6.735
H	2.947	-1.108	6.467
H	2.852	1.183	6.282
H	-7.674	-0.072	1.534
H	-3.279	6.398	6.008
H	-4.827	6.190	5.122
H	-4.506	5.288	6.640
H	-0.657	-7.275	0.051
H	0.181	-4.659	1.572
H	-2.658	-7.044	1.163
H	-2.007	-4.057	0.758
H	-3.263	-4.969	-0.115
H	-3.399	-4.710	1.646
H	-1.755	-6.134	3.265
H	-2.866	-3.468	7.376
H	-2.538	-2.565	8.884
H	-1.695	-4.792	9.038
H	-6.185	1.568	0.336
C	2.297	-6.461	-0.569
C	3.383	-5.886	-1.419
O	4.203	-6.611	-2.004
N	3.444	-4.565	-1.463
C	4.402	-3.807	-2.207
C	5.228	-2.977	-1.234
O	5.269	-3.303	-0.036
N	5.931	-1.980	-1.760
C	6.710	-1.066	-0.951
C	5.878	-0.524	0.176
O	4.711	-0.185	-0.027
N	6.485	-0.370	1.336
C	5.786	-0.049	2.580
C	6.579	0.981	3.369
O	7.790	0.993	3.343
N	5.864	1.799	4.102
C	6.423	2.808	4.966
H	2.782	-4.041	-0.899
H	2.633	-6.479	0.481
H	1.357	-5.889	-0.609
H	2.122	-7.496	-0.890
H	3.918	-3.176	-2.976
H	5.059	-4.530	-2.718
H	5.775	-1.734	-2.729
H	7.013	-0.218	-1.584
H	7.620	-1.558	-0.571
H	7.463	-0.616	1.444
H	4.779	0.282	2.329
H	4.857	1.813	3.905
H	6.451	3.799	4.481
H	5.831	2.892	5.890
H	7.454	2.528	5.221
C	1.886	6.416	-4.723

C	3.218	6.592	-3.984
N	3.314	6.032	-2.845
O	4.111	7.243	-4.478
H	2.089	5.899	-5.672
H	1.126	5.852	-4.162
H	4.189	6.068	-2.334
H	2.604	5.456	-2.415
C	3.961	-0.044	-4.752
C	2.852	-0.920	-5.308
O	2.965	-1.494	-6.390
C	3.608	1.429	-4.913
C	4.623	2.354	-4.317
C	5.527	3.058	-5.128
C	4.655	2.592	-2.923
C	6.496	3.919	-4.564
C	5.585	3.453	-2.341
C	6.497	4.125	-3.160
O	7.419	4.935	-2.558
N	1.752	-0.973	-4.574
C	0.546	-1.609	-5.064
C	-0.658	-0.775	-4.653
O	-0.596	0.000	-3.704
C	0.418	-3.042	-4.537
C	0.264	-3.157	-3.028
S	-1.430	-2.917	-2.453
C	-2.235	-4.370	-3.119
N	-1.740	-0.930	-5.397
C	-3.046	-0.371	-5.042
C	-4.079	-1.458	-5.131
O	-4.108	-2.220	-6.117
C	-3.408	0.810	-5.986
C	-2.489	2.008	-5.836
S	-2.865	2.930	-4.318
C	-1.467	4.029	-4.299
N	-4.976	-1.532	-4.153
C	-6.129	-2.378	-4.249
C	-6.912	-2.365	-2.933
C	-6.097	-2.871	-1.729
C	-6.862	-2.447	-0.446
H	4.880	-0.286	-5.303
H	4.120	-0.260	-3.680
H	2.626	1.617	-4.445
H	3.482	1.647	-5.986
H	5.503	2.921	-6.213
H	3.949	2.078	-2.263
H	1.616	-0.351	-3.779
H	7.202	4.457	-5.201
H	5.568	3.654	-1.269
H	-1.802	-5.293	-2.705
H	-3.289	-4.302	-2.812
H	-2.210	-4.379	-4.218

H	-4.454	1.104	-5.805
H	-3.366	0.432	-7.021
H	7.884	5.434	-3.238
H	0.628	-1.655	-6.162
H	1.335	-3.568	-4.851
H	-0.410	-3.556	-5.052
H	0.866	-2.393	-2.512
H	0.624	-4.134	-2.671
H	-1.777	-1.627	-6.137
H	-2.970	0.006	-4.013
H	-1.433	4.663	-5.199
H	-1.568	4.682	-3.420
H	-0.522	3.470	-4.207
H	-5.802	-3.400	-4.505
H	-4.963	-0.811	-3.409
H	-2.604	2.688	-6.695
H	-1.436	1.685	-5.797
H	-5.095	-2.414	-1.726
H	-7.883	-2.859	-0.441
H	-6.359	-2.784	0.474
H	-6.936	-1.350	-0.411
H	-7.825	-2.970	-3.052
H	-7.243	-1.333	-2.728
C	-6.294	1.843	-3.597
C	-6.037	1.357	-2.221
O	-6.715	1.792	-1.262
O	-5.073	0.522	-2.097
H	-5.477	2.518	-3.892
H	-6.300	1.006	-4.311
H	-7.244	2.389	-3.661
C	-3.575	-1.465	2.121
C	-2.352	-1.487	2.780
C	-3.677	-0.820	0.901
C	-1.238	-0.865	2.237
C	-1.646	1.053	-1.434
C	-0.259	0.414	0.411
C	-0.411	1.051	-0.814
C	-2.568	-0.201	0.353
C	-1.344	-0.223	1.012
N	0.923	0.409	1.018
N	-2.707	0.416	-0.828
H	-2.257	-1.984	3.749
H	1.060	0.015	1.942
H	1.722	0.927	0.640
H	-1.856	1.544	-2.386
H	-0.317	-0.850	2.818
H	-4.463	-1.919	2.567
H	-4.625	-0.774	0.363
H	-3.663	0.468	-1.293
Br	1.039	1.923	-1.662
H	1.494	7.412	-4.970

H	-2.025	-1.894	7.299
H	-5.967	-3.965	-1.779
H	-6.773	-2.055	-5.088
H	0.410	6.010	2.953
H	2.106	-3.906	3.305
H	5.695	-0.964	3.192
H	-0.504	-7.092	2.451

Large binding pocket **4nvi**, H-positions optimized, SCS-MP2/def2-SVP (waters omitted)

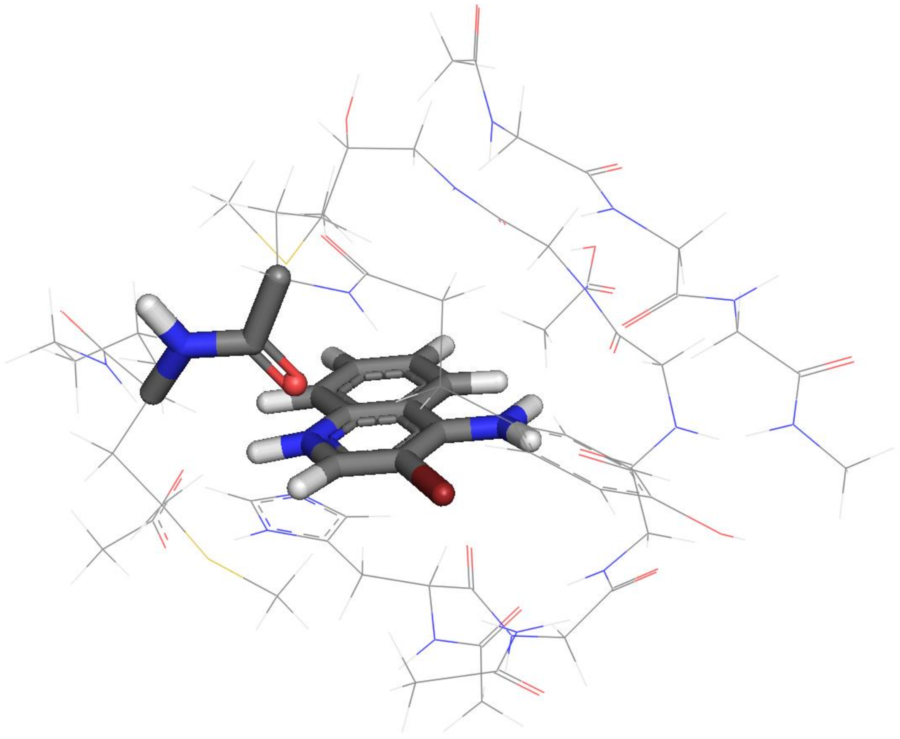

C	-4.090	5.692	5.792
C	-3.379	4.547	5.084
O	-2.561	3.862	5.678
N	-3.695	4.342	3.811
C	-3.076	3.272	3.060
C	-1.689	3.635	2.630
O	-1.044	2.894	1.866
C	-3.931	2.862	1.869
C	-5.003	1.862	2.219
C	-5.280	1.202	3.412
N	-5.918	1.439	1.331
C	-6.739	0.556	1.923
N	-6.347	0.413	3.196
N	-1.199	4.763	3.123
C	0.206	5.086	2.975
C	0.980	4.213	3.950
O	2.193	4.073	3.839
N	0.261	3.628	4.903
C	0.879	2.901	6.007
C	0.907	1.435	5.754
O	-0.078	0.863	5.330
N	2.010	0.770	6.095
C	1.946	-0.661	6.095
C	1.791	-1.267	4.704
O	2.175	-0.690	3.664
N	1.190	-2.455	4.695
C	1.165	-3.307	3.507
C	0.023	-4.292	3.566
O	-0.582	-4.518	4.617
N	-0.237	-4.904	2.411
C	-1.082	-6.066	2.338
C	-1.897	-6.122	1.014
O	-1.022	-6.307	-0.083
C	-2.077	-2.784	7.982
C	-0.747	-3.499	8.052
O	0.261	-3.028	7.493
O	-0.734	-4.573	8.736
H	1.113	-1.023	6.732
H	2.883	-1.065	6.531
H	-7.579	0.064	1.422
H	-3.327	6.415	6.144
H	-4.837	6.227	5.173
H	-4.588	5.287	6.694
H	0.803	-2.858	5.567
H	-0.764	3.726	4.970
H	-4.474	4.844	3.382
H	0.260	3.065	6.913
H	1.890	3.326	6.186
H	-4.772	1.271	4.383
H	0.576	4.860	1.955

H	-2.923	2.404	3.739
H	-3.240	2.421	1.117
H	-4.355	3.769	1.379
H	-1.827	5.414	3.591
H	1.099	-2.673	2.602
H	2.872	1.253	6.348
H	-0.582	-7.173	0.005
H	-2.617	-6.972	1.100
H	0.219	-4.558	1.549
H	-1.760	-6.050	3.214
H	-2.762	-3.346	7.312
H	-2.561	-2.705	8.978
H	-1.619	-4.788	9.087
H	-6.034	1.665	0.295
C	2.231	-6.382	-0.649
C	3.326	-5.839	-1.538
O	4.035	-6.574	-2.221
N	3.449	-4.522	-1.496
C	4.408	-3.754	-2.230
C	5.224	-2.927	-1.246
O	5.260	-3.261	-0.050
N	5.927	-1.923	-1.762
C	6.696	-1.011	-0.941
C	5.856	-0.480	0.185
O	4.688	-0.146	-0.023
N	6.454	-0.332	1.349
C	5.746	-0.021	2.592
C	6.529	1.006	3.392
O	7.741	1.024	3.373
N	5.807	1.816	4.127
C	6.356	2.822	5.001
H	4.727	0.291	2.336
H	6.196	3.847	4.601
H	5.890	2.775	6.008
H	7.446	2.656	5.101
H	2.062	-7.442	-0.912
H	2.544	-6.327	0.415
H	1.283	-5.814	-0.737
H	7.004	-0.154	-1.575
H	7.616	-1.505	-0.559
H	5.788	-1.674	-2.744
H	7.444	-0.569	1.467
H	2.829	-3.999	-0.867
H	4.785	1.813	3.939
H	3.925	-3.116	-3.008
H	5.070	-4.476	-2.758
C	1.733	6.201	-4.580
C	2.892	6.235	-3.663
N	2.741	5.486	-2.577
O	3.947	6.843	-3.938
H	1.797	5.298	-5.224

H	0.752	6.167	-4.062
H	3.451	5.406	-1.831
H	1.836	5.080	-2.339
C	4.063	-0.260	-5.147
C	2.864	-1.046	-5.651
O	2.879	-1.615	-6.764
C	3.698	1.224	-5.130
C	4.756	2.157	-4.587
C	5.523	2.913	-5.446
C	4.930	2.309	-3.218
C	6.506	3.780	-4.973
C	5.905	3.173	-2.717
C	6.686	3.903	-3.608
O	7.662	4.767	-3.154
N	1.821	-1.087	-4.839
C	0.563	-1.686	-5.269
C	-0.584	-0.798	-4.857
O	-0.466	-0.022	-3.927
C	0.336	-3.125	-4.722
C	0.253	-3.267	-3.218
S	-1.335	-2.872	-2.484
C	-2.218	-4.372	-2.931
N	-1.705	-0.961	-5.533
C	-3.001	-0.418	-5.111
C	-4.039	-1.513	-5.185
O	-4.107	-2.250	-6.158
C	-3.436	0.755	-6.002
C	-2.502	1.979	-5.869
S	-2.870	2.896	-4.341
C	-1.403	3.940	-4.237
N	-4.912	-1.572	-4.215
C	-6.162	-2.382	-4.280
C	-6.922	-2.302	-2.936
C	-6.109	-2.816	-1.772
C	-6.845	-2.452	-0.487
H	4.913	-0.462	-5.825
H	4.338	-0.586	-4.118
H	5.368	2.827	-6.534
H	5.982	3.330	-1.628
H	2.773	1.361	-4.528
H	3.428	1.525	-6.164
H	1.738	-0.466	-4.026
H	7.587	4.823	-2.184
H	0.627	-1.771	-6.375
H	4.299	1.753	-2.504
H	7.117	4.392	-5.652
H	-1.823	-5.249	-2.378
H	-3.279	-4.215	-2.657
H	-2.180	-4.543	-4.026
H	-1.311	4.617	-5.112
H	-1.502	4.557	-3.323

H	-0.484	3.324	-4.144
H	1.192	-3.731	-5.092
H	-0.565	-3.553	-5.211
H	-1.786	-1.669	-6.273
H	-4.479	1.039	-5.753
H	-3.447	0.405	-7.056
H	-4.898	-0.833	-3.470
H	-5.102	-2.348	-1.771
H	0.996	-2.601	-2.728
H	0.536	-4.295	-2.906
H	-2.878	-0.057	-4.070
H	-5.881	-3.428	-4.526
H	-7.878	-2.858	-0.479
H	-6.337	-2.828	0.426
H	-6.915	-1.349	-0.396
H	-2.629	2.660	-6.737
H	-1.442	1.651	-5.842
H	-7.870	-2.871	-3.035
H	-7.207	-1.244	-2.751
C	-6.277	1.921	-3.557
C	-6.029	1.325	-2.218
O	-6.639	1.746	-1.244
O	-5.097	0.460	-2.119
H	-5.469	2.650	-3.774
H	-6.237	1.154	-4.358
H	-7.247	2.449	-3.595
C	-3.477	-1.625	2.108
C	-2.286	-1.495	2.810
C	-3.623	-0.990	0.884
C	-1.244	-0.738	2.293
C	-1.729	1.145	-1.378
C	-0.530	1.291	-0.705
C	-0.377	0.654	0.519
C	-2.583	-0.234	0.364
C	-1.395	-0.108	1.068
N	0.764	0.777	1.186
N	-2.739	0.375	-0.818
Br	0.819	2.341	-1.471
H	-4.568	-1.043	0.326
H	-1.957	1.598	-2.359
H	-2.152	-1.991	3.783
H	-0.344	-0.618	2.909
H	0.932	0.325	2.086
H	1.570	1.303	0.807
H	-4.322	-2.190	2.527
H	-3.704	0.379	-1.309
H	1.758	7.084	-5.246
H	-1.941	-1.774	7.557
H	-0.475	-7.000	2.423
H	0.392	6.154	3.199
H	-6.787	-2.021	-5.126

H	2.120	-3.873	3.414
H	-5.953	-3.915	-1.859
H	5.663	-0.949	3.202
C	-2.666	-4.817	0.812
H	-3.348	-4.634	1.665
H	-1.975	-3.955	0.743
H	-3.267	-4.851	-0.117

Large binding pocket **4nvi**$^{\rightarrow NO_2}$, H-positions optimized, SCS-MP2/def2-SVP (waters omitted)

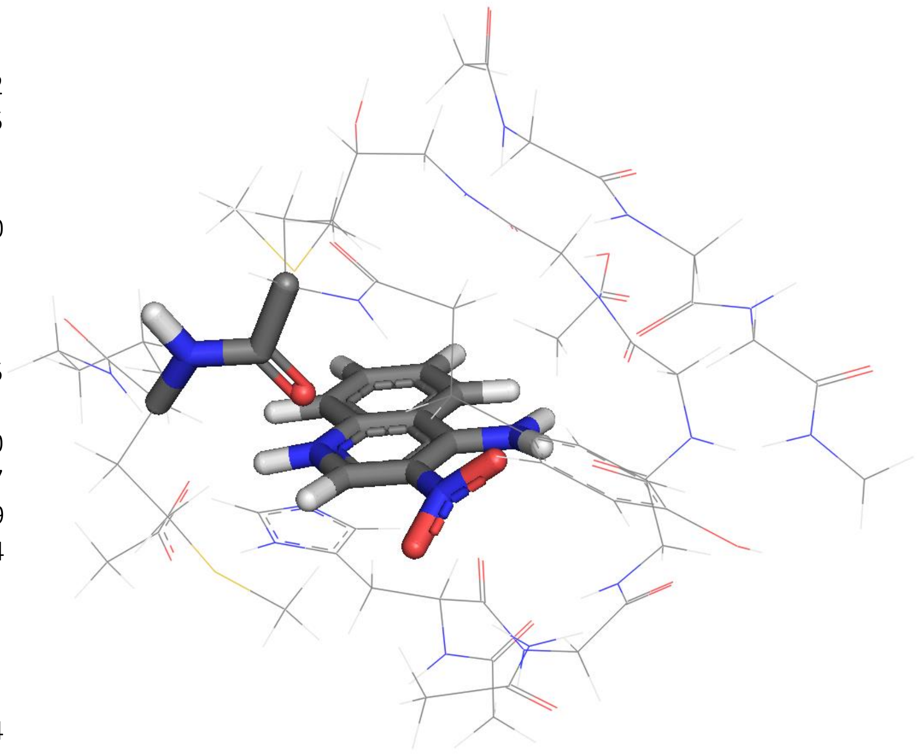

C -4.096 5.673 5.806
C -3.385 4.528 5.098
O -2.567 3.843 5.692
N -3.701 4.323 3.825
C -3.082 3.253 3.074
C -1.695 3.616 2.644
O -1.050 2.875 1.880
C -3.937 2.843 1.883
C -5.009 1.843 2.233
C -5.286 1.183 3.426
N -5.924 1.420 1.345
C -6.745 0.537 1.937
N -6.353 0.394 3.210
N -1.205 4.744 3.137
C 0.200 5.067 2.989
C 0.974 4.194 3.964
O 2.187 4.054 3.853
N 0.255 3.609 4.917
C 0.873 2.882 6.021
C 0.901 1.416 5.768
O -0.084 0.844 5.344
N 2.004 0.751 6.109
C 1.940 -0.680 6.109
C 1.785 -1.286 4.718
O 2.169 -0.709 3.678
N 1.184 -2.474 4.709
C 1.159 -3.326 3.521
C 0.017 -4.311 3.580
O -0.588 -4.537 4.631
N -0.243 -4.923 2.425
C -1.088 -6.085 2.352
C -1.903 -6.138 1.031
C -2.671 -4.834 0.832
O -1.028 -6.321 -0.066
C -2.083 -2.803 7.996
C -0.753 -3.518 8.066
O 0.255 -3.047 7.507
O -0.740 -4.592 8.750
H 1.107 -1.042 6.746
H 2.876 -1.085 6.545
H -7.586 0.045 1.437
H -3.333 6.394 6.159
H -4.842 6.208 5.187
H -4.596 5.267 6.707
H 0.799 -2.879 5.582
H -0.770 3.709 4.986
H -4.478 4.828 3.395
H 0.254 3.046 6.927
H 1.883 3.306 6.200
H -4.780 1.253 4.397

H	0.571	4.847	1.968
H	-2.929	2.385	3.754
H	-3.247	2.403	1.130
H	-4.362	3.750	1.393
H	-1.834	5.394	3.605
H	1.092	-2.693	2.615
H	2.867	1.233	6.359
H	-0.589	-7.188	0.018
H	-2.624	-6.988	1.115
H	-1.980	-3.972	0.765
H	-3.272	-4.865	-0.098
H	-3.353	-4.655	1.685
H	0.219	-4.584	1.565
H	-1.765	-6.070	3.230
H	-2.801	-3.404	7.397
H	-2.522	-2.655	9.005
H	-1.625	-4.808	9.101
H	-6.043	1.647	0.309
C	2.225	-6.401	-0.635
C	3.320	-5.858	-1.524
O	4.029	-6.593	-2.207
N	3.443	-4.541	-1.482
C	4.402	-3.773	-2.216
C	5.218	-2.946	-1.232
O	5.254	-3.280	-0.036
N	5.921	-1.942	-1.748
C	6.690	-1.030	-0.927
C	5.850	-0.499	0.199
O	4.682	-0.165	-0.009
N	6.448	-0.351	1.363
C	5.740	-0.040	2.606
C	6.523	0.987	3.406
O	7.735	1.005	3.387
N	5.801	1.797	4.141
C	6.350	2.803	5.015
H	4.721	0.272	2.347
H	6.272	3.822	4.576
H	5.819	2.812	5.990
H	7.422	2.587	5.187
H	2.093	-7.475	-0.859
H	2.509	-6.293	0.433
H	1.263	-5.864	-0.767
H	6.997	-0.173	-1.561
H	7.609	-1.525	-0.545
H	5.776	-1.690	-2.728
H	7.437	-0.589	1.481
H	2.825	-4.020	-0.850
H	4.780	1.798	3.949
H	3.919	-3.135	-2.994
H	5.063	-4.495	-2.744
C	1.727	6.182	-4.566

C	2.886	6.216	-3.649
N	2.735	5.467	-2.563
O	3.941	6.824	-3.924
H	1.805	5.294	-5.229
H	0.749	6.119	-4.046
H	3.462	5.366	-1.839
H	1.900	4.897	-2.406
C	4.057	-0.279	-5.133
C	2.858	-1.065	-5.637
O	2.873	-1.634	-6.750
C	3.692	1.205	-5.116
C	4.750	2.138	-4.573
C	5.517	2.894	-5.432
C	4.924	2.290	-3.204
C	6.500	3.761	-4.959
C	5.899	3.154	-2.703
C	6.680	3.884	-3.594
O	7.656	4.748	-3.140
N	1.815	-1.106	-4.825
C	0.557	-1.705	-5.255
C	-0.590	-0.817	-4.843
O	-0.472	-0.041	-3.913
C	0.330	-3.144	-4.708
C	0.247	-3.286	-3.204
S	-1.341	-2.891	-2.470
C	-2.224	-4.391	-2.917
N	-1.711	-0.980	-5.519
C	-3.007	-0.437	-5.097
C	-4.045	-1.532	-5.171
O	-4.113	-2.269	-6.144
C	-3.442	0.736	-5.988
C	-2.508	1.960	-5.855
S	-2.876	2.877	-4.327
C	-1.409	3.921	-4.223
N	-4.918	-1.591	-4.201
C	-6.168	-2.401	-4.266
C	-6.928	-2.321	-2.922
C	-6.115	-2.835	-1.758
C	-6.851	-2.471	-0.473
H	4.907	-0.482	-5.810
H	4.331	-0.605	-4.103
H	5.360	2.809	-6.520
H	5.970	3.315	-1.615
H	2.768	1.344	-4.512
H	3.421	1.506	-6.150
H	1.736	-0.486	-4.011
H	7.575	4.809	-2.171
H	0.622	-1.790	-6.361
H	4.278	1.749	-2.492
H	7.110	4.374	-5.637
H	-1.830	-5.267	-2.362

H	-3.287	-4.234	-2.646
H	-2.183	-4.562	-4.011
H	-1.359	4.637	-5.070
H	-1.470	4.483	-3.274
H	-0.490	3.304	-4.193
H	1.186	-3.750	-5.077
H	-0.571	-3.573	-5.197
H	-1.791	-1.686	-6.262
H	-4.485	1.020	-5.739
H	-3.455	0.384	-7.041
H	-4.905	-0.852	-3.459
H	-5.109	-2.367	-1.758
H	0.990	-2.620	-2.716
H	0.529	-4.314	-2.890
H	-2.885	-0.074	-4.057
H	-5.888	-3.448	-4.512
H	-7.883	-2.880	-0.464
H	-6.343	-2.846	0.440
H	-6.925	-1.368	-0.383
H	-2.636	2.639	-6.724
H	-1.448	1.634	-5.828
H	-7.877	-2.891	-3.021
H	-7.214	-1.263	-2.737
C	-6.283	1.902	-3.543
C	-6.035	1.306	-2.204
O	-6.645	1.727	-1.230
O	-5.103	0.441	-2.105
H	-5.473	2.627	-3.761
H	-6.246	1.134	-4.344
H	-7.253	2.432	-3.579
C	-3.483	-1.644	2.122
C	-2.292	-1.514	2.824
C	-3.629	-1.009	0.898
C	-1.250	-0.757	2.307
C	-1.735	1.126	-1.364
C	-0.536	1.272	-0.691
C	-0.383	0.635	0.533
C	-2.589	-0.253	0.378
C	-1.401	-0.127	1.082
N	0.758	0.758	1.200
N	-2.745	0.356	-0.804
H	-4.574	-1.063	0.339
H	-1.948	1.588	-2.345
H	-2.157	-2.014	3.795
H	-0.346	-0.649	2.918
H	0.884	0.364	2.135
H	1.589	1.208	0.774
H	-4.328	-2.210	2.540
H	-3.715	0.356	-1.306
N	0.441	2.001	-1.209
O	1.658	1.630	-1.070

O	0.114	2.988	-1.958
H	1.737	7.078	-5.215
H	-1.961	-1.823	7.503
H	-5.959	-3.934	-1.845
H	-6.794	-2.040	-5.112
H	0.384	6.134	3.215
H	5.656	-0.968	3.216
H	2.114	-3.893	3.428
H	-0.481	-7.020	2.436

4r6s (ΔΔE = –4.4 kcal/mol *vs* Ar-NO)

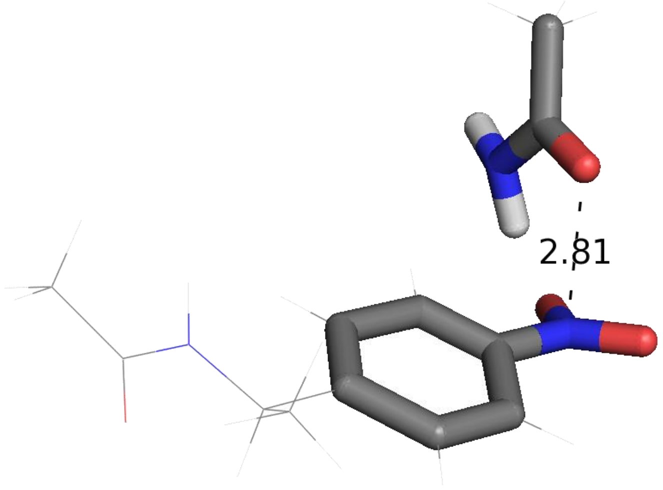

C	-5.0068889	0.9189722	-0.0358056
C	-3.9378889	-0.0080278	-0.5568056
N	-3.6318889	-1.0410278	0.2231944
O	-3.3908889	0.1929722	-1.6458056
H	-4.2163836	-1.1918234	1.0407657
H	-4.9107007	1.8956731	-0.5215747
H	-3.2403082	-1.8966276	-0.1924424
H	-5.9946876	0.5054919	-0.2919190
C	3.0491111	0.3499722	2.4161944
O	3.4641111	-0.8030278	2.3861944
N	2.7001111	1.0919722	1.2151944
C	2.8151111	0.5159722	-0.1068056
C	1.5491111	-0.1970278	-0.5788056
C	3.1621111	1.6149722	-1.0808056
C	1.4111111	-0.5480278	-2.0248056
C	0.2891111	-1.1870278	-2.4678056
C	-0.7848889	-1.5300278	-1.4978056
C	-0.6568889	-1.2120278	-0.1768056
C	0.5731111	-0.5060278	0.3081944
N	-1.9708889	-2.2050278	-1.9688056
O	-2.3278889	-2.0840278	-3.1378056
O	-2.6548889	-2.9310278	-1.1478056
H	0.6495351	-0.2659946	1.3649451
H	2.2560091	1.9987286	1.3186295
H	3.6258460	-0.2302071	-0.0486471
H	2.3221420	2.3177414	-1.1936347
H	2.2171262	-0.3164233	-2.7164822
H	0.1361326	-1.4559506	-3.5115058
H	-1.4217820	-1.4654169	0.5470985
H	3.4033347	1.2314332	-2.0783088
H	4.0387026	2.1773313	-0.7289886
H	-4.9705260	1.0472234	1.0564037
C	2.8931111	1.1339722	3.6951944
H	3.6268062	1.9526391	3.7324765
H	3.0626985	0.4628082	4.5424719
H	1.8915759	1.5813847	3.7780117

2w7x (ΔΔE = –2.7 kcal/mol *vs* Ar-NO; –4.4 kcal/mol *vs* C(O)NH_2 → H)

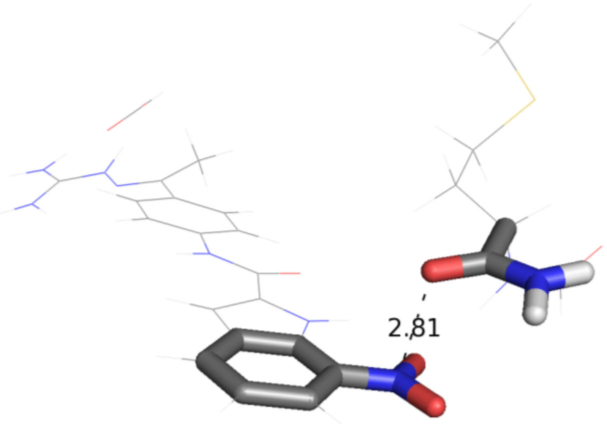

C	0.13615040	5.21807820	-10.21824990
O	-0.38084940	4.81707830	-9.15225020
O	0.26315040	4.50307840	-11.25524960
C	2.76114970	0.94007940	5.81474560
O	2.62014970	1.88707910	6.58674530
N	1.74615000	0.19307960	5.41674570
C	0.40315030	0.42407950	5.89774550
C	-0.03084950	-0.91992010	6.39874540
O	-0.62484940	-1.69491990	5.67374560
C	-0.51384940	0.93107940	4.77874580
C	-0.18484950	2.35307900	4.29674600
S	-0.41584940	3.64307860	5.55074560
C	-2.22384890	3.67607860	5.55374560
N	0.29415040	-1.17192000	7.66374500
O	1.90214990	-2.29491970	3.83374610
N	1.27115010	-3.25091940	4.29474600
O	1.54015000	-3.64591930	5.42874570
C	0.29415040	-3.90291920	3.53374620
C	-0.36384940	-5.04891890	3.97974610
C	-0.01284950	-3.40091940	2.28074660
N	0.43715030	-2.32991970	1.59474670
C	-0.19884950	-2.22191970	0.38874710
C	-1.07184920	-3.28791940	0.26674710
C	-0.94484930	-4.01091920	1.48474680
C	-1.60784910	-5.16191890	1.90474670
C	-1.31484920	-5.67591870	3.17074630
C	0.09315040	-1.19592000	-0.62525260
O	1.00315020	-0.40192020	-0.39925270
N	-0.63884940	-1.20892000	-1.73825230
C	-0.46184940	-0.37592020	-2.84925200
C	-1.28984920	-0.60192020	-3.94825170
C	-1.17784920	0.13107960	-5.09725140
C	-0.22384950	1.12707930	-5.19025140
C	0.63115030	1.34307930	-4.10725170
C	0.51315030	0.59807950	-2.93425200
C	-0.10384950	1.91207910	-6.45625100
C	0.53315030	3.27007870	-6.47425100
N	-0.50284940	1.38807930	-7.54325070
N	-0.43884940	2.00007910	-8.64925040
C	-0.86384930	1.31707930	-9.70925010
N	-0.85184930	1.81907910	-10.92124970
N	-1.21484920	0.12007960	-9.56325010
H	-1.56695970	0.87407570	5.12633310
H	-0.45032980	0.21814260	3.92859760
H	0.88549330	2.41339410	4.00781410
H	-0.77851520	2.60837390	3.39303320
H	-2.54080890	4.46003990	6.26945360
H	-2.66532540	2.71352340	5.88557340
H	-2.63011040	3.93389900	4.55249590
H	0.47583850	1.19106070	6.69700440

H	1.90079470	-0.64073180	4.83138350
H	0.82223370	-0.45244040	8.16293920
H	-0.10868940	-5.42594150	4.97859650
H	1.15976710	-1.68918650	1.93476190
H	-1.73023460	-3.54743040	-0.56841090
H	-2.35243530	-5.64746570	1.25681930
H	-1.82431080	-6.57793050	3.53904260
H	-2.06315750	-1.38705820	-3.88139770
H	-1.85823650	-0.04807940	-5.94201670
H	1.41791650	2.10872940	-4.16644780
H	1.18717080	0.77444200	-2.09183050
H	0.63776560	3.70338390	-5.46335010
H	-0.02291470	4.00167210	-7.09914160
H	1.54852160	3.21080290	-6.92526870
H	-0.47635100	2.82112600	-11.08515580
H	-1.19481400	-0.23745570	-8.59831400
H	-1.47636090	-0.48685970	-10.33423380
H	-0.34225790	3.08781780	-8.80277550
H	-1.20213780	1.28612500	-11.71263680
H	0.49031960	6.29223610	-10.26858970
H	-1.40441890	-1.88046030	-1.80775040
H	3.72153079	0.69755892	5.41008281
H	0.23482504	-2.07904587	8.08040211

5del (ΔΔE = –1.3 kcal/mol *vs* Ar-NO)

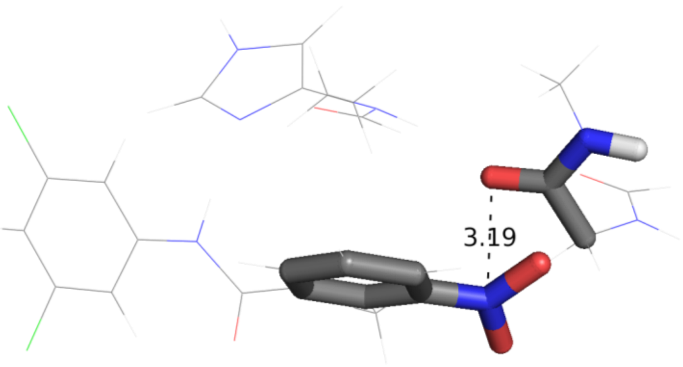

N	3.2999091	1.3189242	-5.4910303
C	3.0619091	0.8059242	-4.1560303
C	2.0729091	1.6139242	-3.3390303
O	1.1269091	1.0589242	-2.7760303
H	4.2484639	1.5111529	-5.7961385
H	4.0101741	0.7600579	-3.6052180
H	2.6524870	-0.2117854	-4.2044526
N	-1.6710909	0.8319242	-1.8190303
C	-2.0000909	1.0299242	-0.4140303
C	-0.8530909	1.7379242	0.3079697
C	-1.1130909	1.9849242	1.7609697
C	-1.0600909	3.1199242	2.4979697
N	-1.4760909	0.9809242	2.6329697
C	-1.6360909	1.4859242	3.8409697
N	-1.3910909	2.7809242	3.7879697
H	-1.4485371	3.4402914	4.5575531
H	-0.7209463	1.0495024	-2.1379817
H	-2.2079796	0.0575506	0.0629039
H	-0.6385292	2.7001938	-0.1828097
H	-0.8299337	4.1465718	2.2320037
H	-1.9270003	0.9411676	4.7332511
H	0.0621428	1.1291088	0.2052723
Cl	-3.2830909	-6.7060758	3.3769697
Cl	-6.1180909	-2.1120758	3.9589697
C	-3.4040909	-4.9720758	3.2699697
N	-1.2890909	-2.0560758	2.3319697
O	0.1749091	-3.6650758	1.5449697
C	-2.3050909	-4.2480758	2.8379697
N	3.4619091	-0.0580758	-0.9070303
O	3.8949091	1.2169242	-1.1470303
C	-2.3850909	-2.8690758	2.7189697
O	3.8259091	-1.0630758	-1.7600303
C	-3.5540909	-2.2240758	3.0909697
C	-4.6520909	-2.9480758	3.5279697
C	-4.5800909	-4.3270758	3.6159697
C	-0.0590909	-2.4980758	1.7789697
C	1.0009091	-1.4500758	1.5259697
C	1.7089091	-1.3060758	0.3349697
C	2.6779091	-0.3020758	0.2519697
C	2.9279091	0.5259242	1.3329697
C	2.2269091	0.3739242	2.5099697
C	1.2719091	-0.6170758	2.6019697
C	1.4319091	-2.2170758	-0.8480303
H	2.2258757	-2.9700269	-0.9331919
H	-1.4304639	-1.0277119	2.3835774
H	-1.3897891	-4.7641093	2.5669817
H	-3.6226016	-1.1378475	3.0095610
H	-5.4433154	-4.9004813	3.9515506
H	3.6889151	1.2973650	1.2294882
H	2.4438175	1.0213819	3.3591841

H	0.7120261	-0.7453641	3.5280908
H	1.4228479	-1.6432993	-1.7841250
H	0.4769149	-2.7357858	-0.7258039
C	-2.4060909	-0.0150758	-2.6100303
H	-1.9227673	-0.1628355	-3.6037622
O	-3.4670909	-0.5310758	-2.2980303
N	2.3299091	2.9629242	-3.2700303
H	3.1558309	3.3092056	-3.7440695
C	1.5399091	3.8489242	-2.4590303
H	0.4830464	3.5582320	-2.5277200
H	1.6455986	4.8795993	-2.8191515
H	1.8390022	3.8051072	-1.3987031
C	2.3269091	1.2189242	-6.4530303
H	2.7003758	1.5516276	-7.4513138
O	1.1849091	0.8279242	-6.2700303
H	-2.9274028	1.6189690	-0.3243633

4lq3 (ΔΔE = –1.0 kcal/mol *vs* Ar-NO)

C	1.1041750	-4.7609000	1.4228250
C	1.3741750	-3.2599000	1.2388250
O	0.5471750	-2.4229000	1.5818250
N	2.5421750	-2.9329000	0.7028250
C	2.9261750	-1.5519000	0.4128250
H	2.6241090	-0.9117087	1.2508049
H	0.2475279	-5.0190020	0.7853048
H	3.1470430	-3.6725097	0.3648045
C	-1.0438250	3.6391000	1.2658250
C	-0.5338250	-0.2229000	-1.9511750
C	-0.3378250	3.3751000	-1.0291750
C	-1.2518250	0.0641000	0.3338250
C	-0.5918250	4.1931000	0.0748250
O	-0.8348250	0.6771000	3.4968250
O	-2.0278250	2.8181000	3.8708250
O	0.4211750	0.6411000	-4.6461750
O	-0.6338250	2.8811000	-4.2281750
O	-2.0998250	-2.5659000	0.4138250
O	-0.6398250	-3.0449000	-1.4961750
O	-3.1838250	0.9201000	2.7338250
O	1.7021750	2.3991000	-3.4791750
C	-1.0058250	-0.7729000	-0.7531750
C	-1.2728250	2.2711000	1.3568250
C	-0.3098250	1.1551000	-2.0631750
C	-0.5588250	1.9951000	-0.9581750
C	-1.0298250	1.4431000	0.2468250
N	-1.2408250	-2.0839000	-0.6201750
S	-1.8558250	1.6491000	2.9048250
S	0.3111750	1.8041000	-3.6581750
H	-1.2804174	2.4561674	-4.8335192
H	-1.2260868	4.2563695	2.1451941
H	-1.6898198	-0.4021209	1.2120201
H	-0.3528588	-0.8749126	-2.8056733
H	-0.9622571	-0.2509094	3.1969052
H	0.0651607	3.8226006	-1.9363828
H	1.9568986	-5.4050558	1.1695210
H	0.8114799	-4.9340004	2.4656921
H	2.4362569	-1.1808856	-0.5010989
H	4.0131618	-1.4990287	0.2849739
H	-0.4113143	5.2656055	0.0086078

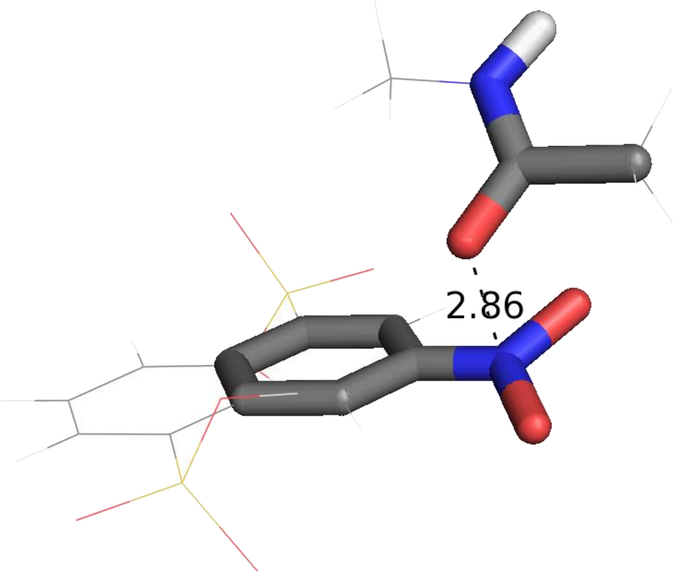

2w5u (ΔΔE = –0.6 kcal/mol *vs* Ar-NO)

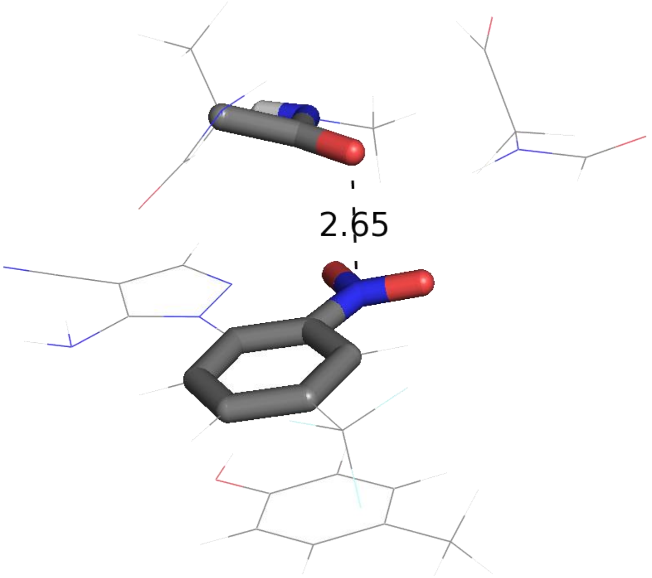

N	-2.5100685	-0.2810548	2.7501370
C	-1.1240685	-0.3070548	3.2411370
C	-0.2380685	-1.3780548	2.5561370
O	-0.6310685	-1.9880548	1.5411370
C	-1.1100685	-0.4980548	4.7351370
H	-0.1132391	-0.3377967	5.1682025
H	-0.7032890	0.6814900	2.9887879
H	-1.4375010	-1.5096609	5.0123237
H	-2.9486469	-1.1883341	2.6011573
H	-1.7851455	0.2251694	5.2118431
N	-0.9010685	-4.4980548	-0.1728630
C	-2.3370685	-4.3890548	-0.3648630
C	-3.1370685	-4.6080548	0.9121370
O	-4.3640685	-4.8060548	0.8681370
H	-2.5669982	-4.5876666	1.8729975
H	-2.6672278	-5.1370918	-1.1000773
H	-2.5935929	-3.3883081	-0.7508345
H	-0.3995504	-3.6425008	0.0730585
C	0.9019315	0.1849452	-5.2168630
C	1.6009315	0.9999452	-4.1498630
C	2.6709315	0.4699452	-3.4228630
C	1.2039315	2.3029452	-3.8808630
C	3.3179315	1.2159452	-2.4418630
C	1.8429315	3.0539452	-2.9128630
C	2.8919315	2.5069452	-2.1928630
O	3.4979315	3.2709452	-1.2278630
H	4.1507249	2.7218656	-0.7606924
H	0.4049131	-0.6978849	-4.7843572
H	2.9976901	-0.5531874	-3.6193502
H	0.3705356	2.7388324	-4.4348722
H	4.1408546	0.7819861	-1.8694524
H	1.5310842	4.0750485	-2.6896354
H	0.1365948	0.7770930	-5.7329675
C	0.1089315	1.5939452	-0.1528630
N	0.9239315	1.9189452	0.8941370
O	0.4389315	-1.8520548	-0.9388630
C	-0.0550685	0.3339452	-0.7788630
N	1.8849315	1.0619452	1.4611370
O	1.5559315	-0.6910548	0.4341370
C	-0.9380685	0.0839452	-1.8348630
N	0.6849315	-0.7770548	-0.4098630
C	-1.7390685	1.0989452	-2.3178630
N	2.4309315	4.6849452	4.4381370
C	-1.6420685	2.3309452	-1.7318630
C	-0.7460685	2.5439452	-0.6928630
C	-2.7170685	0.8989452	-3.4188630
C	2.4979315	1.6579452	2.5451370
C	1.9029315	2.8999452	2.6691370
C	0.8999315	3.0449452	1.6211370
C	2.1869315	3.8839452	3.6341370

F	-3.8000685	1.5789452	-3.1218630
F	-3.0060685	-0.3790548	-3.4538630
F	-2.2810685	1.3049452	-4.5988630
H	-0.9626753	-0.9205693	-2.2411014
H	-2.2472537	3.1693838	-2.0734391
H	-0.6664102	3.5660971	-0.3482586
H	3.2883293	1.1436130	3.0768702
N	0.1149315	4.2109452	1.3911370
H	-0.8855818	4.0085725	1.5010311
H	0.3806733	4.9256708	2.0726824
C	-0.1540685	-5.5650548	-0.7198630
H	0.9420626	-5.3937490	-0.6694815
O	-0.7020685	-6.5660548	-1.1438630
N	0.9319315	-1.7970548	3.2171370
H	1.3245157	-1.1807567	3.9198656
C	1.8009315	-2.8500548	2.6651370
H	2.4553711	-2.4576971	1.8755454
H	1.1639555	-3.6291799	2.2309547
H	2.3923209	-3.2753703	3.4847395
C	-3.0580685	0.8019452	2.0421370
H	-4.0610063	0.5945200	1.6190811
O	-2.4640685	1.8669452	1.9761370
H	1.6138648	-0.1844104	-5.9696740

1y2k (ΔΔE = –1.2 kcal/mol *vs* Ar-NO)

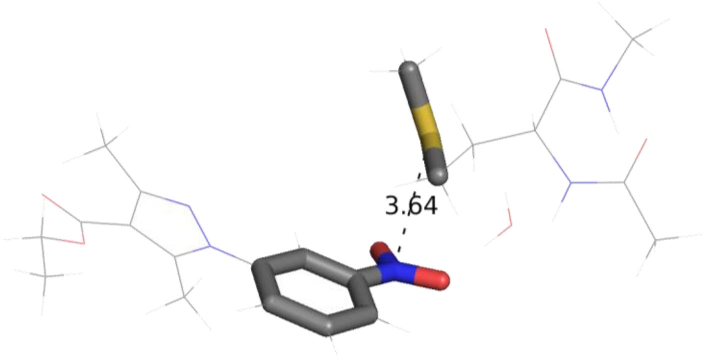

C	2.2357350	1.9032944	-6.7778102
C	2.0877350	0.4632944	-6.2798102
O	2.8337350	-0.4187056	-6.7028102
N	1.1407350	0.2332944	-5.3698102
C	0.9627350	-1.0817056	-4.7548102
C	0.6257350	-2.1987056	-5.7348102
O	0.8727350	-3.3647056	-5.4438102
C	-0.1012650	-1.0157056	-3.6518102
C	0.3877350	-0.3567056	-2.3698102
S	1.8057350	-1.1667056	-1.5748102
C	1.3057350	-2.8537056	-1.5598102
N	0.0747350	-1.8587056	-6.8948102
C	-0.2302650	-2.8767056	-7.8988102
O	2.7487350	1.6562944	0.8881898
N	1.9107350	2.0602944	0.1011898
O	2.1917350	2.4502944	-1.0178102
C	0.5907350	2.0122944	0.4501898
C	-0.3732650	2.6952944	-0.3128102
C	0.1507350	1.2772944	1.5721898
C	-1.2012650	1.2412944	1.9281898
C	-2.1422650	1.9272944	1.1571898
C	-1.7252650	2.6532944	0.0391898
N	-1.6292650	0.5242944	2.9991898
C	-1.3982650	0.7332944	4.2761898
C	-0.5702650	1.8872944	4.8131898
C	-2.0132650	-0.2337056	4.9961898
C	-2.6612650	-1.0857056	3.9911898
N	-2.3902650	-0.5777056	2.8671898
C	-3.5012650	-2.3237056	4.1421898
C	-2.0592650	-0.4507056	6.4551898
O	-2.7932650	-1.3177056	6.9061898
O	-1.1702650	0.2112944	7.2011898
C	-1.1052650	-0.0727056	8.6191898
C	-0.3802650	1.0792944	9.3491898
O	4.9587350	1.6132944	-1.8628102
H	1.3939616	2.5774026	-6.5177405
H	-0.4357808	-2.0505107	-3.4401490
H	-1.0014281	-0.4823428	-4.0334499
H	-0.4503252	-0.2845828	-1.6411791
H	0.7463451	0.6811553	-2.5414430
H	2.0870466	-3.4120794	-1.0068840
H	0.3410011	-3.0105962	-1.0304387
H	1.2414266	-3.2891936	-2.5810739
H	1.9286530	-1.4003585	-4.3055507
H	0.5827959	1.0120429	-5.0154357
H	-0.6072752	-3.7828507	-7.3886116
H	0.0116199	-0.8711681	-7.1427046
H	-0.0385027	3.2715139	-1.1858612
H	0.8902757	0.7179837	2.1612065
H	-3.2025893	1.8741213	1.4426577

H	-2.4590081	3.2029961	-0.5680057
H	-0.9273238	2.1745476	5.8164466
H	-0.6121809	2.7655910	4.1386411
H	0.4946746	1.5932728	4.9174099
H	-4.3919148	-2.1105864	4.7647481
H	-3.8087341	-2.6876015	3.1442674
H	-2.9368209	-3.1139926	4.6755123
H	-2.1353276	-0.2230031	9.0040022
H	-0.5684102	-1.0369874	8.7508311
H	-0.3059452	0.8376686	10.4277977
H	-0.9393598	2.0295728	9.2386709
H	0.6431436	1.2224457	8.9491839
H	2.3618026	1.8778170	-7.8763525
H	3.1684011	2.3221861	-6.3492047
H	-0.9962653	-2.4936426	-8.5998448
H	0.6766252	-3.1647119	-8.4732413
H	4.6604192	0.6924172	-1.9869585
H	4.1993295	1.9921639	-1.3755729

4. Overview of (approved) drugs containing a nitro aromatic.

Table S5. Entries in this table were collected from reference 22 in the paper (unmarked) and from a search in the Reays© database for nitroaromatic molecules that were drug approved (marked with an '*').

Entry	Name	General structure	R_1	R_2	R_3
1[a]	Chloramphenicol*	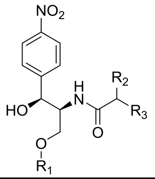	H	Cl	Cl
2	Chloramphenicol Palmitate*		$C(O)C_{15}H_{31}$		
3	Chloramphenicol Succinate sodium*		$C(O)C_2H_4CO_2Na$		
4	Azidamphenicol		H	H	N_3
5	Nefidipine (Adalat)*	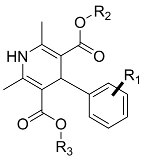	*o*-NO_2	Me	Me
6	Nisoldipine*				CH_2-*i*-Pr
7[b]	Nimodipine*		*m*-NO_2	CH_2-*i*-Pr	C_2H_4OMe
8	Nitrendipine*			Me	Et
9	Aranidipine				$CH_2C(O)Et$
10	Falnidipine				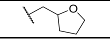
11	Nicardipine hydrochloride*				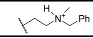
12	Iercanidipine*				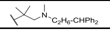
13	Barnipidine				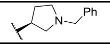
14	Manidipine*				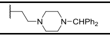
15	Benidipine*				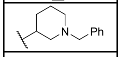
16	Pranidipine				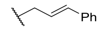
17	Cylnidipine			C_2H_4OMe	
18	Efonidipine			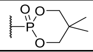	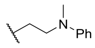
19	Nilvadipine	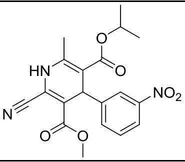			
20	Clonazepam*	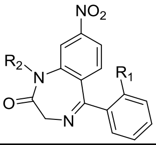	Cl	H	
21	Nitrazepam*		H		
22	Nimetazepam			Me	
23	Flunitrazepam*		F		
24	Loprazolam*	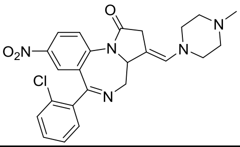			
25[c]	Flutamide*	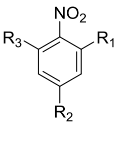	H	NHC(O)-iPr	CF_3
26	Nilutamide*			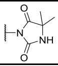	
27	Netobimin			SC_3H_7	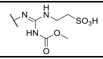
28	Pyrrolnitrin*		Cl	H	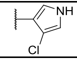
29	Nitromide	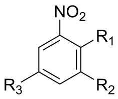	H	$C(O)NH_2$	NO_2
30	Niclofolan*			homo-coupled	Cl
31[d]	Nifursol		OH	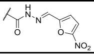	NO_2
32	Nitisinone*		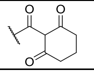	H	CF_3
33[e]	Venetoclax*		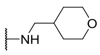		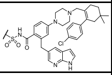
34	Entacapone*		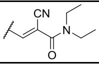		

35[f]	Tolcapone*	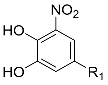	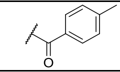		
36	Opicapone*		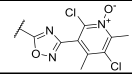		
37	Nisuflazole*	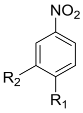	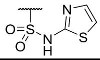		
38	Nitrefazole		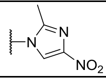		
39	Nitrodan		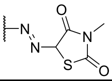		
40	Acenocoumarol*		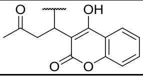	H	
41	Dandrolene* (Dantrolene sodium or Dantrium; N–H = N^-Na^+)		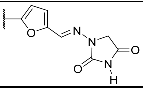		
42	Clefamide		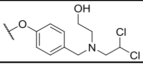		
43	Etofamide		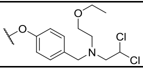		
44	Amocarzina*		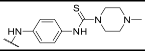		
45[g]	Nimesulide*		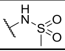	OPh	
46	Niclosamide*		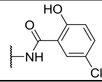	Cl	
47	Nitrefazone		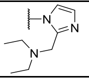	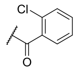	
48	Nitromersol	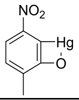			
49	Nitroxynil	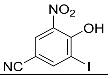			
50	Disophenol	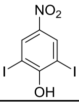			
51	Nitracrine	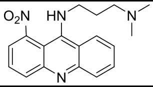			
52[h]	Nitroxoline	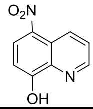			
53	Rubitecan*	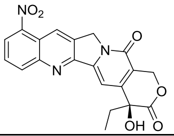			
54[i]	Oxamniquine*	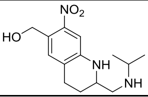			

a) PDB codes that containing a Chloramphenicol ligand are: 1CLA, 1K01, 1NJI, 1QHS, 1QHY, 1USQ, 2JKJ, 2JKN, 2JKN, 2UXP, 2W5P, 2XAT, 3CLA, 3JBV, 3U9F, 4BN6 , 4CLA, 4EJV , 4OAD , 4OA , 4V7T , 4V7W , 4ZOW, 6IEY and 6ND5. Other ligands with a very similar nitro aromatic part include: 1GRR, 2DQT, 2DQU, 6CFJ, 6CFK and 6CFL (see also Figure S37 and Figure S38); b) Nimodipine in found only in structure 5KMF (see also Figure S39a); c) Flutamide itself is not encountered in the PDB, but its active metabolite, hydroxy Flutamide was found in: 2AX6, 4OGH, 4OH5, 4OH6, 4OHA, 4OIL, 4OIU and 4OJ9 (see also Figure S39b); d) Nifursol was not found in the DBP, but two structures containing the same 2,4-dinitrophenol fragment are 1KFY and 1NEN; e) A somewhat similar ligand to Venetoclax is found in 4MAN (see also Figure S39c); f) Tolcapone is found as ligand in structures: 3S68, 4D7B, 4PYL, 5A6I (see also Figure S39d); g) PDB structures that contain a Nimesulide ligand are: 1ZWP, 2OTH, 3E9X, 3N8X, 3QL6 and 4EIX (see also Figure S40); h) A Nitroxoline ligand is present in structures 3AI8 and 5Y1Y (see also Figure S41a); i) Structures 5BYJ and 5TIX contain an Oxamniquine ligand (see also Figure S41b).

The PDB was inspected for all the drug molecules collected in Table S5. Only nine of these drugs (or very similar molecules) were present as a ligand in a total of 57 PDB structures: Chloramphenicol (and related ligands),[a] Nimodipine,[b] Hydroxy Flutamide (the active metabolite of Flutamide),[c] Nifursol-like ligands,[d] a Venetoclax-like ligand,[e] Tolcapone,[f] Nimesulide,[g] Nitroxoline[h] and Oxamniquine[i] (see also notes a-i in Table S5). All structures were inspected manually, in particular the 5 Å envelope surrounding the NO_2 moiety. In 32 of the 57 structures (56%), there is at least one electron rich moieties near the nitro group (i.e., atoms with lone-pair electrons and/or aromatic rings). These 32 structures included all of the nine abovementioned drugs –except the Finurisol-like ligands– and are summarized in Figures S37 – S41 below. The structures were the nitro aromatic ligand in not near an electron rich atom/residue are briefly described below, grouped by ligand and (where relevant) similar protein structures:

Chloramphenicol(-like): In transferases _1cla, 3cla, 4cla and 3u9f_, the nitro moiety is pointing into a hydrophobic pocket consisting out of Leu-29, Tyr-168, Val-162 and Ile-172 (for 1cla, 3cla and 4cla) and out of Ala-29, Phe-144, Leu-158, Val-160 and Phe-166 (for 3u9f); In transferases _1qhs, 1qhy and 1qrr_, the nitro moiety is nested into a hydrophobic pocket consisting out of Val-36, Ile-64, Phe-68, Asp-93, Val-94 and Leu-96; Within adhesins _1usq, 2jkj, 2jkl, 2jkn and 2w5p_, the nitro moiety is stuck onto a hydrophobic side of the protein consisting out of Phe-43 and Ile-111; In transcription protein _2uxp_, transferase _2xat_, transport protein _4zow_ and hydrolase _6iey_ there are no lone-pair bearing atoms or aromatic residues nearby the nitro moiety. **Nifursol-like:** In _1kfy_ (chains B and N), a Finursol-like ligand is bound in an aromatic rich pocket (Phe-17 and Phe-206), but the aromatic residues adopt a more T-shaped geometry; in _1nen_ the nitro-aromatic moiety of a Finursol-like ligand is in between two aliphatic residues (Pro-160 and Ile-28). **Tolcapone:** The nitro groups of the Tolcapone ligand in _4d7b_ (chains A and B) and in _5a6i_ are not very close to any residue. **Nimesulide:** In both _2oth and 4eix_, only Ile-19 is nearby the nitro moiety of the Nimesulide ligand. **Nitroxoline:** In _5yiy_, the nitro group of the Nitroxoline ligand is in between aliphatic residues Pro-82 and Leu-92. **Oxamniquine**: In _5byj_, Val-128 and Thr-157 are nearest the nitro moiety of the Oxamniquine ligand (Met-38 seems close, but the S-atom is pointing away from N).

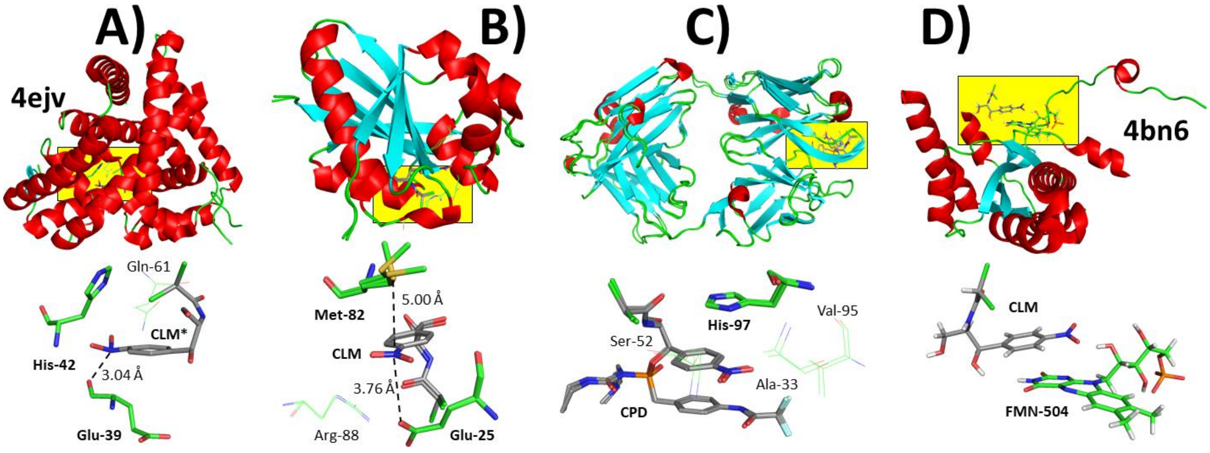

Figure S37. Illustrations of proteins structures containing *Chloramphenicol(-like)* ligands (CLM) where the nitro moiety is nearest a lone-pair bearing atom and/or an aromatic ring. **A)** Transcription regulator 4ejv has the nitro group stacked with His-42 and the amide-carbonyl of Glu-39; **B)** In transferases 4oad and 4oae, the nitro moiety is closest to Met-82 (shortest $N^{NO2} – S^{Met-82}$ = 5.00 Å) and a salt-bridge between Glu-25 and Arg-88. The shortest $N^{NO2} – O^{Glu-25}$ distance is 3.76 Å; **C)** Within immune system proteins 2dqt and 2dqu, the nitro moiety is surrounded by residues Ala-33, Ser-52, Val-95 and His-97 (which is π-stacked with the nitro arene). The nitro group might also be interacting with the amide of the CPD ligand; **D)** In Oxidoreductase 4nb6, the nitro aromatic is stacked on top of ligand FMN-504. See also entry 1 and note a) in Table S5.

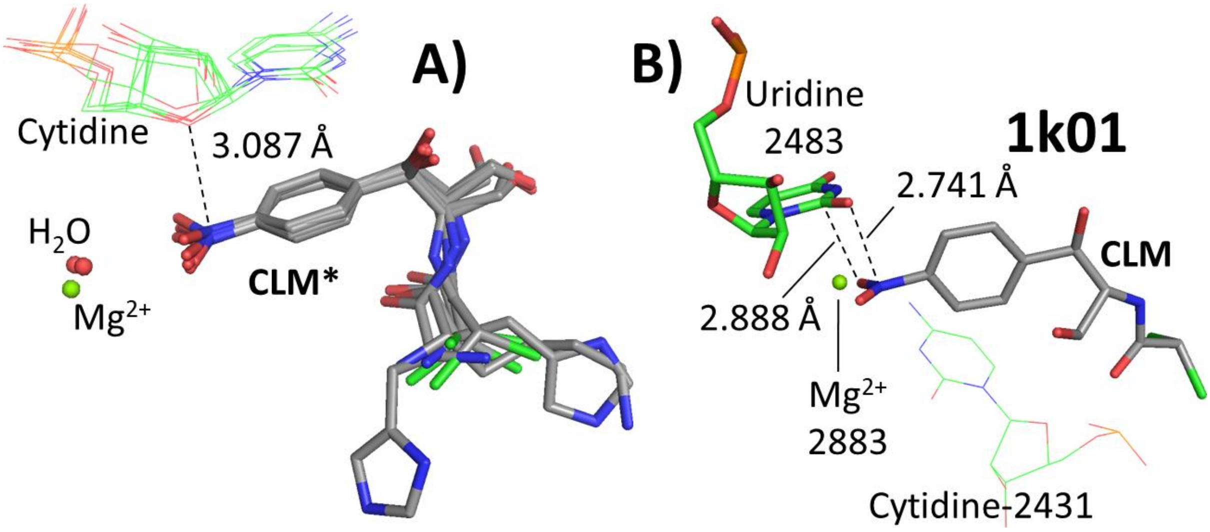

Figure S38. Illustrations of ribosome structures containing *Chloramphenicol(-like)* ligands (CLM) where the nitro moiety is nearest a lone-pair bearing atom. **A)** Overlay with shortest interatomic N-O distance of CLM(-like) ligands and a cytidine nucleoside as found within 4v7t (with C-2452, 3.591 Å), 4v7w (with C-2452, 3.216 Å), 6cfj (with C-2464, 3.125 Å, nitro O's also close to Mg^{2+}-109), 6cfk (with C-2464, 3.107 Å, nitro O's also close to H_2O-5766), 6cfl (with C-2464, 3.178 Å) and 6nd5 (with C-2452, 3.087 Å, nitro O's also close to H_2O-201). Additionally (not shown), within structures 1jni and 3jbv the nucleosides are roughly similarly position (i.e., near the nitro moiety), but the intermolecular distances are larger than those shown in A): the distance between the ether-like O of the nucleoside and the NO_2 N-atom are 3.916 Å in 1nji (with adenosine-2100) and 3.408 Å in 3jbv (with cytidine-2452); **B)** In ribosome structure 1k01 the NO_2 fragment of a chlorampenicol ligand is coordinated to Mg^{2+}-2833 with Mg–O distance of 2.503 Å. Concurrently, one of the carbonyls in uridine-2483 is very close to the nitro group with interatomic N–O and O–C distances of respectively 2.741 Å and 2.888 Å. See also entry 1 and note a) in Table S5.

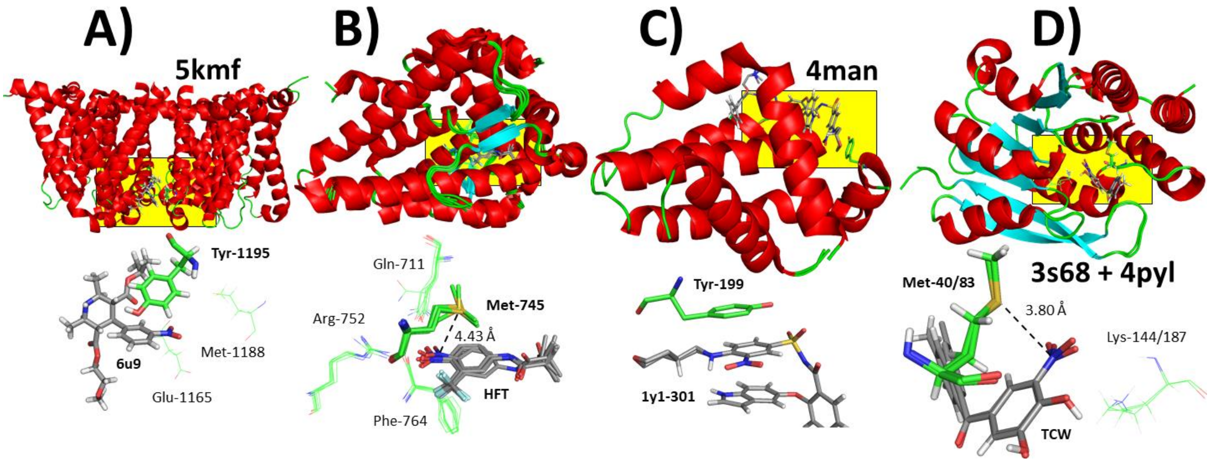

Figure S39. Illustrations of proteins structures containing _Nimodipine_ (A), _Hydroxy Flutamide_ (B), _Venetoclax_ (C) and _Tolcapone_ (D) where the nitro moiety is nearest a lone-pair bearing atom and/or an aromatic ring. **A)** In the transport protein tetramer 5kmf the NO_2 fragment of a Nimodipine ligand is in closest proximity to Glu-1165 (N – O = 5.31 Å), Met-1188 (N – S = 5.55 Å) and Try-1195 (N – centroid = 4.84 Å); **B)** Hormone receptors 2ax6, 4ogh, 4oh5, 4oh6, 4oha, 4oil, 4oiu and 4 oj9 contain hydroxyl flutamide (HFT); in all cases, Met-745 is in close proximity of the nitro moiety (N – $S^{Met\text{-}745}$ = 4.43 Å on average); **C)** Apoptosis regulator / inhibitor 4man has its nitro aromatic moiety stacked in between an aromatic part of the ligand (1y1) and Tyr-199 (average centroid – N distance = 3.52 Å); **D)** The Tolcapone ligand within transferases 3s68 and 4pyl is in close proximity to the S-atom of Met-40 (3s68) or Met-83 (4pyl) with a shortest N – S distance of 3.7952 Å in 4pyl. See also entries 7 (note b), 25 (note c), 33 (note e) and 35 (note f) in Table S5.

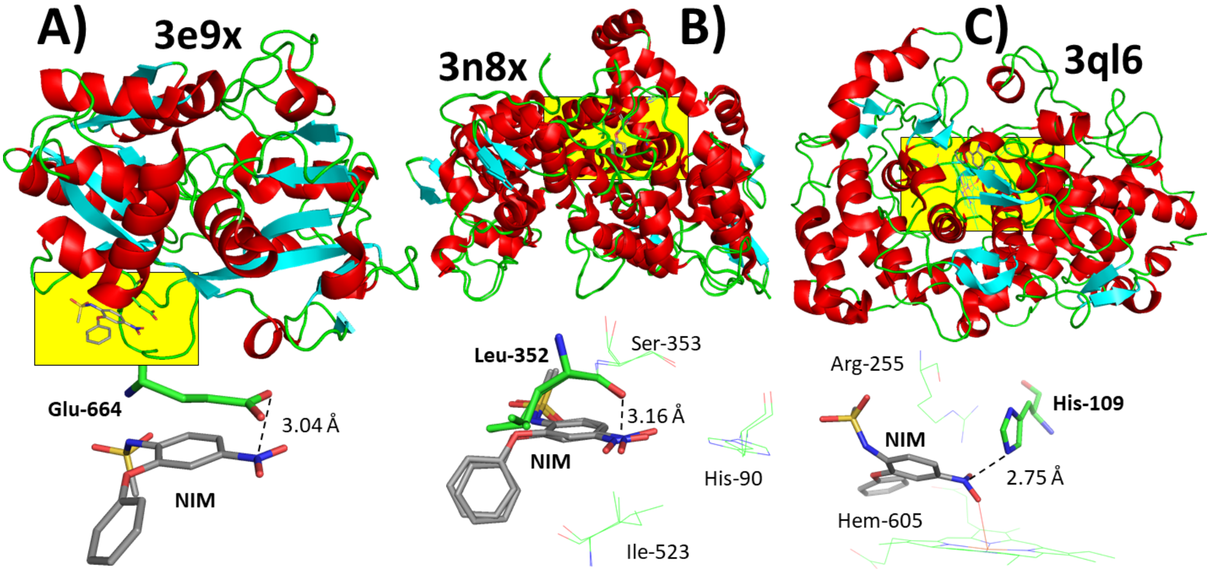

Figure S40. Illustrations of proteins structures containing _*Nimesulide*_ (NIM) where the nitro moiety is nearest a lone-pair bearing atom and/or an aromatic ring. **A)** In metal binding protein 3e9x a Nimesulide ligand is near Glu-664 with a short $O^{Glu\text{-}664} – N^{NO2}$ distance of 3.044 Å (and $O^{NO2} – C^{Glu\text{-}664}$ = 3.081 Å); **B)** In oxidoreductase 3n8x a Nimesulide ligand is near the amide O-atom of Leu-352 with a short $O^{Leu\text{-}352} – N^{NO2}$ distance of 3.155 Å (in chain A) and 3.206 Å (in chain B); **C)** In oxidoredictase 3ql6 a Nimesulide ligand is coordinated to Hem-605 and has the N-atom of His-109 very close to the nitro's N-atom with an N – N distance of 2.748 Å. See also entry 45 and note g) in Table S5.

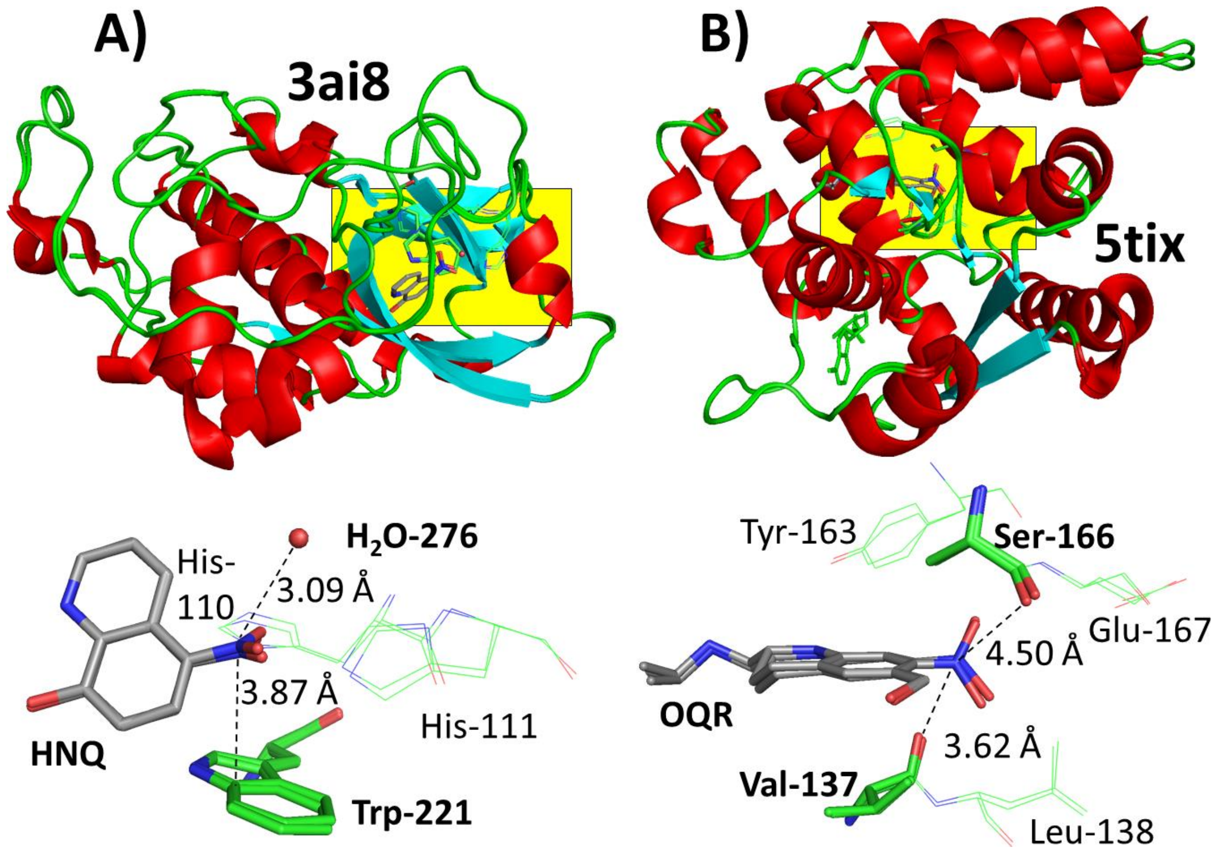

Figure S41. Illustrations of proteins structures containing *Nitroxoline* (A) and *Oxamniquine* (B) ligands where the nitro moiety is nearest a lone-pair bearing atom and/or an aromatic ring. **A)** In chains A and B of hydrolase 3ai8, the nitro group of a Nitroxoline ligand is in closest proximity to Trp-211 (shortest N^{NO2} – $C^{Trp\text{-}211}$ = 3.87Å) and water molecule 276 (N^{NO2} – $O^{H2O\text{-}276}$ = 3.089 Å); **B)** In chains A and B of transferase 5tix, the nitro group of a Oxamniquine ligand is in closest proximity to the amide O-atoms of Ser-166 (average N^{NO2} – $O^{Ser\text{-}166}$ = 4.50 Å) and Val-137 (average N^{NO2} – $O^{val\text{-}137}$ = 3.62 Å). See also entries 52 (note h) and 54 (note i) in Table S5.

References:

[1] a) C. T. Lee, W. T. Yang, R. G. Parr, *Phys. Rev. B* **1988**, *37*, 785-789; b) A. D. Becke, *Phys. Rev. A* **1988**, *38*, 3098-3100.

[2] a) G. A. Petersson, A. Bennett, T. G. Tensfeldt*, et al.*, *J. Chem. Phys.* **1988**, *89*, 2193-2218; b) G. A. Petersson, M. A. Allaham, *J. Chem. Phys.* **1991**, *94*, 6081-6090.

[3] M. J. Frisch, M. Headgordon, J. A. Pople, *Chem. Phys. Lett.* **1990**, *166*, 275-280.

[4] T. H. Dunning, *J. Chem. Phys.* **1989**, *90*, 1007-1023.

[5] S. Grimme, *J. Chem. Phys.* **2003**, *118*, 9095-9102.

[6] a) F. Weigend, R. Ahlrichs, *Phys. Chem. Chem. Phys.* **2005**, *7*, 3297-3305; b) F. Weigend, *Phys. Chem. Chem. Phys.* **2006**, *8*, 1057-1065.

[7] A. Bondi, *J. Phys. Chem.* **1964**, *68*, 441-452.

[8] R. F. W. Bader, *Acc. Chem. Res.* **1985**, *18*, 9-15.

[9] G. te Velde, F. M. Bickelhaupt, E. J. Baerends*, et al.*, *J. Comput. Chem.* **2001**, *22*, 931-967.

[10] C. F. Macrae, I. J. Bruno, J. A. Chisholm*, et al.*, *J. Appl. Crystallogr.* **2008**, *41*, 466-470.

[11] A. C. Wallace, R. A. Laskowski, J. M. Thornton, *Protein Engineering* **1995**, *8*, 127-134.

[12] S. Grimme, J. Antony, S. Ehrlich*, et al.*, *J. Chem. Phys.* **2010**, *132*, 19.

[13] W. Qiu, R. Lam, O. Voytyuk*, et al.*, *Acta crystallographica. Section D, Biological crystallography* **2014**, *70*, 2740-2753.

[14] K. E. Zahn, H. Belrhali, S. S. Wallace*, et al.*, *Biochemistry* **2007**, *46*, 10551-10561.

[15] C. S. Raman, H. Li, P. Martasek*, et al.*, *Biochemistry* **2001**, *40*, 13448-13455.

[16] K. D'Ambrosio, R. M. Vitale, J. M. Dogne*, et al.*, *Journal of medicinal chemistry* **2008**, *51*, 3230-3237.

[17] W. A. Offen, D. L. Zechel, S. G. Withers*, et al.*, *Chemical communications (Cambridge, England)* **2009**, 2484-2486.

[18] T. Roret, A. Thuillier, F. Favier*, et al.*, *Fungal genetics and biology : FG & B* **2015**, *83*, 103-112.

[19] X. Deng, D. Matthews, P. K. Rathod*, et al.*, *Acta crystallographica. Section F, Structural biology communications* **2015**, *71*, 553-559.

[20] J. Huang, Z. Xu, D. Wang*, et al.*, *Glycobiology* **2010**, *20*, 1643-1653.

[21] A. M. Gulick, C. B. Bauer, J. B. Thoden*, et al.*, *The Journal of biological chemistry* **2000**, *275*, 398-408.

[22] E. Schonbrunn, A. Becker, S. Betzi*, et al.*, *To be bublished* **2012**.

[23] P. A. Wright, R. C. Wilmouth, I. J. Clifton*, et al.*, *The Biochemical journal* **2000**, *351 Pt 2*, 335-340.

[24] J. Sanz-Aparicio, J. A. Hermoso, M. Martinez-Ripoll*, et al.*, *Proteins* **1998**, *33*, 567-576.

[25] E. Fan, E. A. Merritt, Z. Zhang*, et al.*, *Acta crystallographica. Section D, Biological crystallography* **2001**, *57*, 201-212.

[26] P. A. Patten, N. S. Gray, P. L. Yang*, et al.*, *Science* **1996**, *271*, 1086-1091.

[27] M. Konno, M. Ito, T. Hayano*, et al.*, *Journal of molecular biology* **1996**, *256*, 897-908.

[28] R. J. Rosenfeld, E. D. Garcin, K. Panda*, et al.*, *Biochemistry* **2002**, *41*, 13915-13925.

[29] A. J. Scheidig, S. M. Franken, J. E. Corrie*, et al.*, *Journal of molecular biology* **1995**, *253*, 132-150.

[30] C. Schiene, U. Reimer, M. Schutkowski*, et al.*, *FEBS letters* **1998**, *432*, 202-206.

[31] H. Khan, R. J. Harris, T. Barna*, et al.*, *The Journal of biological chemistry* **2002**, *277*, 21906-21912.

[32] G. Kontopidis, M. J. Andrews, C. McInnes*, et al.*, *ChemMedChem* **2009**, *4*, 1120-1128.

[33] D. H. Juers, T. D. Heightman, A. Vasella*, et al.*, *Biochemistry* **2001**, *40*, 14781-14794.

[34] R. Rai, A. Kolesnikov, P. A. Sprengeler*, et al.*, *Bioorganic & medicinal chemistry letters* **2006**, *16*, 2270-2273.

[35] J. C. Pickens, E. A. Merritt, M. Ahn*, et al.*, *Chem. Biol.* **2002**, *9*, 215-224.

[36] M. Prizant, Y. Eisenberg-Domovich, V. P. Hytonen*, et al.*, *Journal of molecular biology* **2006**, *358*, 754-763.

[37] N. Cremades, A. Velazquez-Campoy, M. Martinez-Julvez*, et al.*, *ACS chemical biology* **2009**, *4*, 928-938.

[38] A. G. Jobson, G. T. Lountos, P. L. Lorenzi*, et al.*, *The Journal of pharmacology and experimental therapeutics* **2009**, *331*, 816-826.

[39] G. T. Lountos, A. G. Jobson, J. E. Tropea*, et al.*, *Journal of structural biology* **2011**, *176*, 292-301.

[40] E. Schonbrunn, S. Betzi, R. Alam*, et al.*, *Journal of medicinal chemistry* **2013**, *56*, 3768-3782.

[41] A. Zervosen, R. Herman, F. Kerff*, et al.*, *Journal of the American Chemical Society* **2011**, *133*, 10839-10848.

[42] G. T. Lountos, A. G. Jobson, J. E. Tropea*, et al.*, *FEBS letters* **2011**, *585*, 3245-3249.

[43] M. Michikawa, H. Ichinose, M. Momma*, et al.*, *The Journal of biological chemistry* **2012**, *287*, 14069-14077.

[44] M. Nagae, N. Nishi, T. Murata*, et al.*, *Glycobiology* **2009**, *19*, 112-117.

[45] E. A. Merritt, Z. Zhang, J. C. Pickens, *et al.*, *Journal of the American Chemical Society* **2002**, *124*, 8818-8824.
[46] Y. S. Choi, H. Zhang, J. S. Brunzelle, *et al.*, *Proceedings of the National Academy of Sciences of the United States of America* **2008**, *105*, 6858-6863.
[47] J. Schiebel, A. Chang, B. Merget, *et al.*, *Biochemistry* **2015**.
[48] A. B. Burgin, O. T. Magnusson, J. Singh, *et al.*, *Nature biotechnology* **2010**, *28*, 63-70.
[49] *Crystal Structure of the complex of three phase partition treated lipase from Thermomyces lanuginosa with Lauric acid and P-nitrobenzaldehyde (PNB) at 2.1 resolution*, http://www.rcsb.org/pdb/explore/explore.do?structureId=4KJX.
[50] E. A. Merritt, S. Sarfaty, I. K. Feil, *et al.*, *Structure (London, England : 1993)* **1997**, *5*, 1485-1499.
[51] Y. Nakatani, D. S. Larsen, S. M. Cutfield, *et al.*, *Biochemistry* **2014**, *53*, 3318-3326.
[52] S. J. Stachel, C. A. Coburn, D. Rush, *et al.*, *Bioorganic & medicinal chemistry letters* **2009**, *19*, 2977-2980.
[53] L. Feng, H. Sun, Y. Zhang, *et al.*, *FASEB journal : official publication of the Federation of American Societies for Experimental Biology* **2010**, *24*, 3861-3868.
[54] D. D. Mitchell, J. C. Pickens, K. Korotkov, *et al.*, *Bioorganic & medicinal chemistry* **2004**, *12*, 907-920.
[55] B. Stec, A. Cheltsov, J. L. Millan, *Acta crystallographica. Section F, Structural biology and crystallization communications* **2010**, *66*, 866-870.
[56] W. Zhu, Y. Zhang, W. Sinko, *et al.*, *Proceedings of the National Academy of Sciences of the United States of America* **2013**, *110*, 123-128.
[57] *To be published*, http://www.rcsb.org/pdb/explore/explore.do?structureId=4MIK.
[58] *The crystal structure of a 2-fluoroxylotriosyl complex of the Vibrio sp. AX-4 beta-1,3-xylanase at 1.2 A resolution*, http://www.rcsb.org/pdb/explore/explore.do?structureId=3VPL.
[59] *Crystal Structure of the ternary complex of lipase from Thermomyces lanuginosa with Ethylacetoacetate and P-nitrobenzaldehyde at 2.3 A resolution*, http://www.rcsb.org/pdb/explore/explore.do?structureId=4N8S.
[60] *Crystal structure of the cdk2 in complex with thiazolylpyrimidine inhibitor*, http://www.rcsb.org/pdb/explore/explore.do?structureId=4EK8.
[61] J. C. Pickens, D. D. Mitchell, J. Liu, *et al.*, *Chemistry & biology* **2004**, *11*, 1205-1215.
[62] O. Simak, P. Pachl, M. Fabry, *et al.*, *Organic & biomolecular chemistry* **2014**, *12*, 7971-7982.
[63] R. Alag, A. M. Balakrishna, S. Rajan, *et al.*, *Eukaryotic cell* **2013**, *12*, 627-634.
[64] R. Croci, D. Tarantino, M. Milani, *et al.*, *FEBS letters* **2014**, *588*, 1720-1725.
[65] N. I. Howard, M. V. Dias, F. Peyrot, *et al.*, *ChemMedChem* **2015**, *10*, 116-133.
[66] Y. Kim, M. Makowska-Grzyska, S. K. Gorla, *et al.*, *Acta crystallographica. Section F, Structural biology communications* **2015**, *71*, 531-538.
[67] *Structure of three phase partition - treated lipase from Thermomyces lanuginosa in complex with lauric acid at 2.1 A resolution*, http://www.rcsb.org/pdb/explore/explore.do?structureId=4S0X.
[68] E. Papadopoulos, S. Jenni, E. Kabha, *et al.*, *Proceedings of the National Academy of Sciences of the United States of America* **2014**, *111*, E3187-3195.
[69] G. T. Lountos, S. Cherry, J. E. Tropea, *et al.*, *Acta crystallographica. Section F, Structural biology communications* **2015**, *71*, 199-205.
[70] D. Tarantino, M. Pezzullo, E. Mastrangelo, *et al.*, *Antiviral research* **2014**, *102*, 23-28.
[71] *Deciphering the role of 2-O-sulfotransferase in regulating heparan sulfate biosynthesis*, http://www.rcsb.org/pdb/explore/explore.do?structureId=4NDZ.
[72] M. Fischer, R. G. Coleman, J. S. Fraser, *et al.*, *Nature chemistry* **2014**, *6*, 575-583.
[73] G. L. Card, L. Blasdel, B. P. England, *et al.*, *Nature biotechnology* **2005**, *23*, 201-207.
[74] *Crystal structure of a GNAT superfamily acetyltransferase PA4794 in complex with chloramphenicol*, http://www.rcsb.org/pdb/explore/explore.do?structureId=4OAD.
[75] H. J. Li, C. T. Lai, P. Pan, *et al.*, *ACS chemical biology* **2014**, *9*, 986-993.
[76] *Mutated B-Glucosidase a from Paenibacillus Polymyxa Showing Increased Stability*, http://www.rcsb.org/pdb/explore/explore.do?structureId=1UYQ.
[77] B. U. Klink, R. S. Goody, A. J. Scheidig, *Biophysical journal* **2006**, *91*, 981-992.
[78] P. S. Dragovich, B. P. Fauber, J. Boggs, *et al.*, *Bioorganic & medicinal chemistry letters* **2014**, *24*, 3764-3771.
[79] T. Teramoto, Y. Sakakibara, M. C. Liu, *et al.*, *Biochemical and biophysical research communications* **2009**, *379*, 76-80.

[80] *Crystal Structure of 5-CARBOXYVANILLATE Decarboxylase from Novosphingobium Aromaticivorans*, http://www.rcsb.org/pdb/explore/explore.do?structureId=4QS5.
[81] T. Teramoto, Y. Sakakibara, M. C. Liu*, et al.*, *Biochemical and biophysical research communications* **2009**, *383*, 83-87.
[82] *Crystal Structure of Ligw2 Decarboxylase from Novosphingobium Aromaticivorans*, http://www.rcsb.org/pdb/explore/explore.do?structureId=4QS6.
[83] *The atomic resolution Crystal structure of the Phospholipase A2 (PLA2) complex with Nimesulide reveals its weaker binding to PLA2*, http://www.rcsb.org/pdb/explore/explore.do?structureId=1ZWP.
[84] D. P. Marciano, D. S. Kuruvilla, S. V. Boregowda*, et al.*, *Nature communications* **2015**, *6*, Art. Nr. 7443.
[85] P. Hothi, A. Roujeinikova, K. A. Khadra*, et al.*, *Biochemistry* **2007**, *46*, 9250-9259.
[86] T. H. Scheuermann, D. R. Tomchick, M. Machius*, et al.*, *Proceedings of the National Academy of Sciences of the United States of America* **2009**, *106*, 450-455.
[87] J. Key, T. H. Scheuermann, P. C. Anderson*, et al.*, *Journal of the American Chemical Society* **2009**, *131*, 17647-17654.
